# Organocatalytic Asymmetric Approach to γ,δ-Functionalization of 3-Cyano-4-styrylcoumarins via Bifunctional Catalysis

**DOI:** 10.1021/acs.orglett.2c02836

**Published:** 2022-10-17

**Authors:** Marta Romaniszyn, Anna Skrzyńska, Joanna Dybowska, Łukasz Albrecht

**Affiliations:** † Institute of Organic Chemistry, Lodz University of Technology, Żeromskiego 116, 90-924 Łódź, Poland

## Abstract

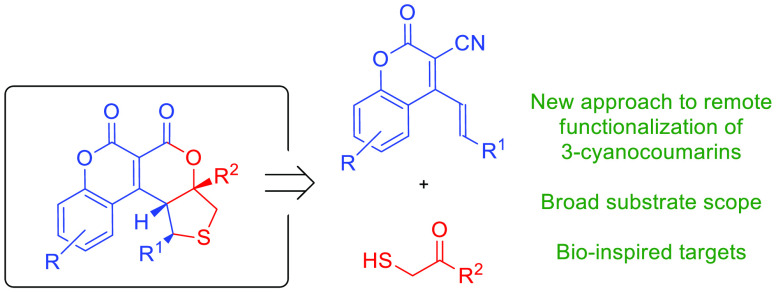

A novel organocatalytic reaction cascade between 3-cyano-4-styrylcoumarins and 2-mercaptoacetophenones is described. It is based on stereocontrolled functionalization of cyanocoumarins proceeding in a sequence of *thia*-Michael/aldol/annulation reactions. This highly diastereo- and enantioselective reaction is realized by employing enantioselective bifunctional catalysis and exhibits a broad substrate scope and excellent functional group tolerance. The synthetic application involves the transformation of the imidoester group, thus opening access to biologically relevant coumarin and δ-lactone-fused products.

Compounds bearing a poly(hetero)cyclic fused ring system are identified as an important group of molecules in the contemporary organic and medicinal chemistry. Due to their wide biological and synthetic relevance, they constitute an inspiration for the chemical community.^1^ Among various privileged heterocyclic structures, δ-lactone^2^ and coumarin^3^ rings are considered key units found in many optically active natural products that exhibit a large spectrum of biological activities (Scheme 1, top). Similarly, the substituted tetrahydrothiophene derivatives attract much attention owing to their significant value as building blocks and synthetic targets.^4^ Therefore, the combination of such biorelevant frameworks leads to new types of products with promising properties. In this context, the organocatalytic cascade reactions have been recognized as diverse strategies providing access to carbo- and heterocyclic scaffolds in an asymmetric fashion.^5^ 2-Mercaptocarbonyl compounds constitute an interesting group of reactants in synthesis of tetrahydrothiophenes with the organocatalytic cascades involving these systems initiated by the *thia*-Michael addition followed by the intramolecular aldol reaction (Scheme 1, center).^6^ The formation of a nucleophilic tertiary alcohol in such a cascade allows for subsequent intramolecular reactions leading to the construction of unique poly(hetero)cyclic fused ring systems.^7^ However, such synthetic strategies still remain a challenge, particularly in the field of stereocontrolled remote functionalizations involving vinylogous Michael acceptors.^8^ In recent years, 4-methyl-3-cyanocoumarin and its derivatives have been recognized as highly attractive pronucleophilic vinylogous reactants in enantioselective reactions (Scheme 1, bottom).^9^ The incorporation of an olefin moiety in their structure provides access to a new class of vinylogous Michael acceptors that participate in the catalytic asymmetric remote γ,δ-functionalizations.

**Scheme 1:**
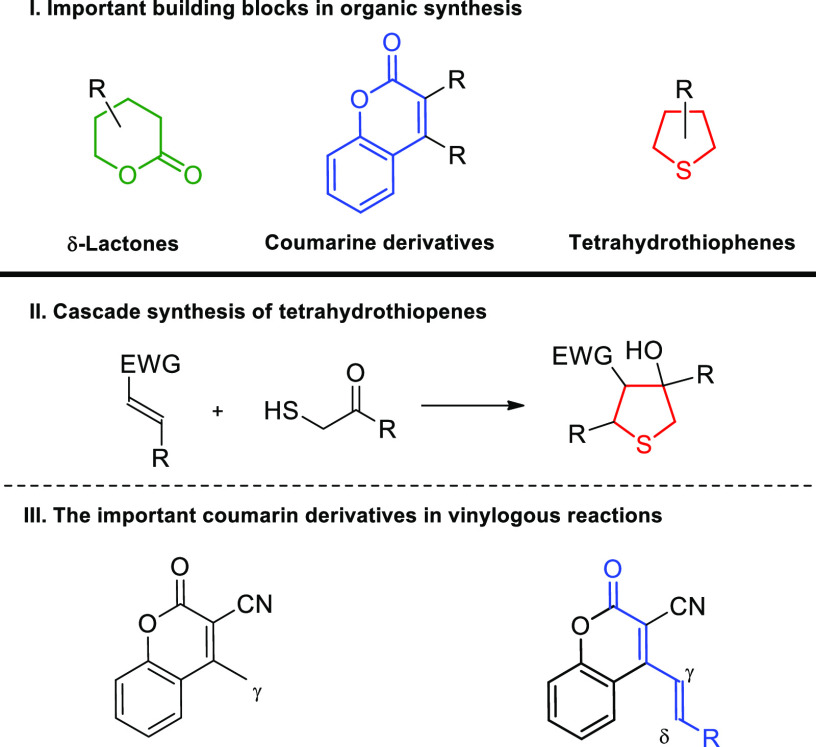
Important Structural Motifs and Building Blocks in Organic Chemistry

Notably, there are only a few examples of organocatalytic transformations involving these systems (Scheme 2, top). In 2019, the Yuan group described the enantioselective domino 1,6-addition/annulation reaction of 3-cyano-4-styrylcoumarins with isatin-derived Morita–Baylis–Hillman carbonates catalyzed by a chiral Brønsted base, in which the final product bears an additional cyclopentene framework.^10^ Moreover, Wang and Chen established an efficient NHC-catalyzed oxidative γ,δ-functionalization leading to the chiral derivatives containing a cyclohexanone unit.^11^ Very recently, we have demonstrated that 3-cyano-4-styrylcoumarins readily participate in the doubly vinylogous 1,6-addition with dienolates derived from 5-substituted-furan-2(3*H*)-ones.^12^ Despite these achievements, the remote transformations of this class of compounds remain limited.

**Scheme 2:**
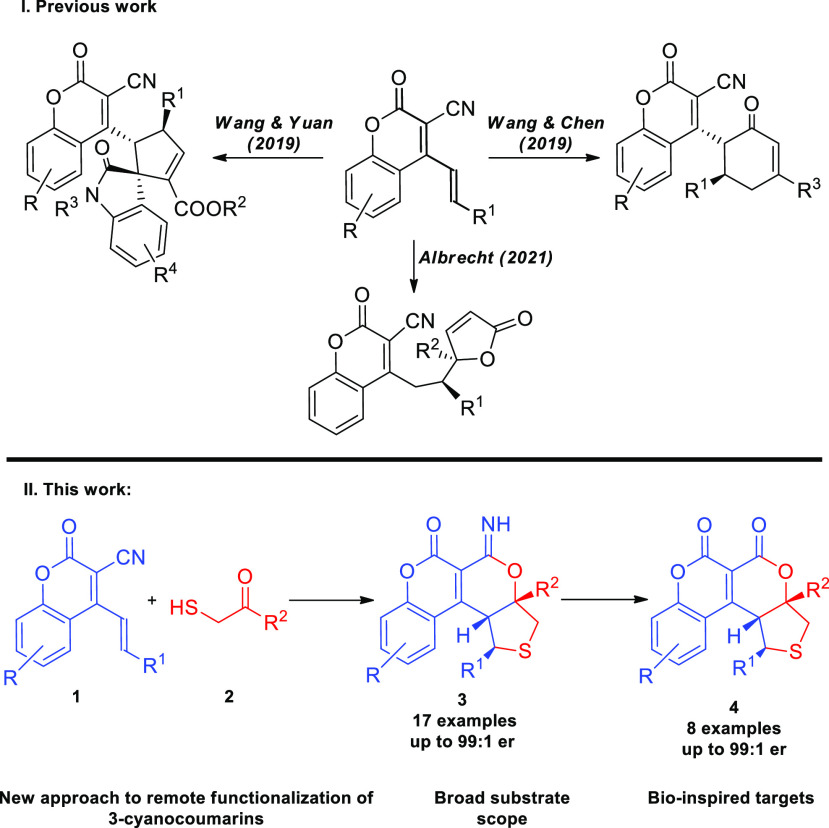
Remote Functionalization of 3-Cyano-4-Styrylcoumarins and the Synthetic Objectives of Our Study

To the best of our knowledge, the reaction between coumarins **1** and 2-mercaptocarbonyl compounds **2** promoted by a modified cinchona alkaloid catalyst has never been achieved. As part of our continuing efforts on organocatalytic asymmetric construction of polycyclic frameworks containing δ-lactone and coumarin motifs,^12,13^ a new approach for the remote γ,δ-functionalization of **1** based on a stereocontrolled cascade involving *thia*-Michael/aldol/annulation reactions was devised (Scheme 2, bottom). As a consequence, the unique polycyclic chiral products **3** containing coumarin, 2*H*-pyran-2-imine and tetrahydrothiophene units were obtained. Furthermore, the efficient transformation of the imidoester group of **3** opened access to biologically important δ-lactone derivatives **4**. At the outset of our studies, the reaction between 2-oxo-4-styryl-2*H*-chromene-3-carbonitrile **1a** and 2-mercaptoacetophenone **2a** was utilized as a model transformation. It was satisfying to find that the envisaged cascade was viable and chiral product **3a** was afforded with variable amounts of *thia*-Michael reaction adduct **5a** (Table 1, entries 1–3). In order to improve the enantioselectivity, a series of cinchona alkaloid catalysts **6** were screened, showing that **6c** bearing a squaramide moiety was the best (Table 1, entry 3 vs 1–2). Moreover, extending the reaction time to 72 h allowed **3a** to be obtained as the sole product of the cascade (Table 1, entry 3 vs 4) and the addition of molecular sieves limited the formation of the product of hydrolysis of **3a** (Table 1, entry 4 vs 5). The evaluation of different solvents indicated that CDCl_3_ was superior in terms of chemoselectivity and yield of the cascade reaction (Table 1, entry 5 vs 6–8). Further optimization studies were focused on the evaluation of concentration effect (Table 1, entries 9–10) and reducing the catalyst loading to 5 mol % (Table 1, entry 11). Neither of these changes led to improvement of the results. Finally, modulating the molar ratio of the reactants led to a slightly higher yield of the process and the product **3a** was obtained with excellent selectivity (Table 1, entry 12). Similar results of the reaction carried out in freshly distilled chloroform were observed (Table 1, entry 12 vs 13), and these conditions were found to be optimal.

**Table 1:**
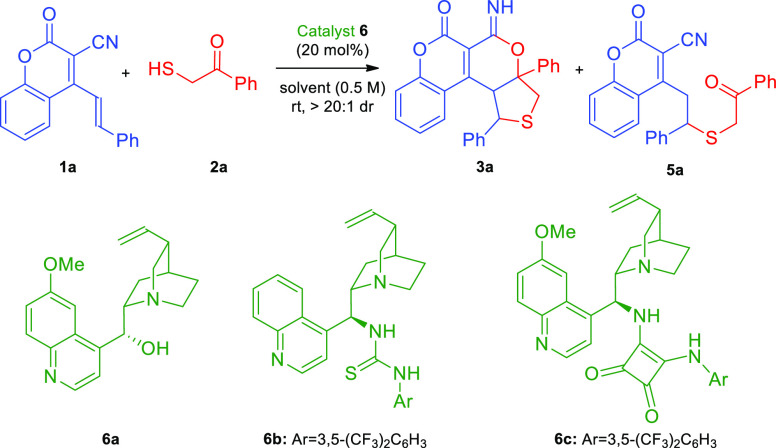
Stereocontrolled γ,δ-Functionalization of 3-Cyano-4-styrylcoumarins *via* Bifunctional Catalysis: Optimization Studies^a^

	Cat.	Solvent	Conv. (yield) [%]^b^	**3a**:**5a**^c^	er^d^
1	**6a**	CDCl_3_	>95	1:0.6	28:72
2	**6b**	CDCl_3_	>95	1:0.6	52:48
3	**6c**	CDCl_3_	>95	1:1.4	98:2
4^e^	**6c**	CDCl_3_	>95 (60)	1:0	98:2
5^e^^,^^f^	**6c**	CDCl_3_	88 (65)	1:0	98:2
6^e^^,^^f^	**6c**	CH_2_Cl_2_	83	1:0.9	n.d.
7^e^^,^^f^	**6c**	CH_3_CN	52	1:2.6	n.d.
8^e^^,^^f^	**6c**	Toluene	83	1:1.3	n.d.
9^e^^,^^f^^,^^g^	**6c**	CDCl_3_	>95 (70)	1:0	98:2
10^e^^,^^f^^,^^h^	**6c**	CDCl_3_	95 (70)	1:0	97.5:2.5
11^e^,^f^,^i^	**6c**	CDCl_3_	>95	1:0.5	n.d.
12^e^^,^^f^^,^^j^	**6c**	CDCl_3_	>95 (76)	1:0	98:2
13^e^^,^^f^^,^^j^^,^^k^	**6c**	CHCl_3_	>95 (76)	1:0	98:2

a Reactions performed on a 0.05 mmol scale using **1a** (1 equiv) and **2a** (2 equiv) in 0.1 mL of the solvent for 24 h.

b Conversion as determined by ^1^H NMR of a crude reaction mixture. In parentheses yield of isolated product **3a** after column chromatography is given.

c Determined by ^1^H NMR of a crude reaction mixture.

d Determined by a chiral stationary phase UPC^2^ for product **3a**.

e Reaction performed for 72 h.

f Reaction performed with sieves 3A.

g Reaction performed in 0.05 mL of the solvent.

h Reaction performed in 0.2 mL of the solvent.

i Reaction performed using 5 mol % of catalyst.

j Reaction performed using **1a** (1 equiv) and **2a** (1.2 equiv).

k Freshly distilled over P_2_O_5_ chloroform was used as a solvent.

Having identified the optimal conditions, the scope of the cascade was explored (Table 2). To our delight, various 2-mercaptocarbonyl compounds **2** with different steric and electronic character of substituents on the aromatic ring underwent an organocatalytic cascade smoothly. In almost all cases, substrates bearing electron-donating or electron-withdrawing groups on the phenyl ring provided optically active products **3b**–**g** with good to high yields and excellent stereoselectivity (compare entries **3b**–**d** vs **3e**–**g**). The position of the substituents in **2** had no significant effect on the selectivity of the cascade. However, in the case of **3d**,**f,g**, lower yields were observed. Additionally, the bulky 2-naphthyl-group in **2h** was also well-tolerated. In the course of further scope studies, the possibility of modifying the structure of 2-oxo-4-styryl-2*H*-chromene-3-carbonitrile **1** was attempted as outlined in Table 2. In general, it was determined that the position and electron properties of the substituents at phenyl ring in **1b**–**h** had no significant effect on both the enantioselectivity and the efficiency of the reaction leading to the corresponding **3i**–**o**. Only in the reaction between **1g** bearing a nitro group in the *para*-position of the phenyl ring and **2a**, the product **3n** was obtained in moderate yield. Notably, when **1i**–**j** bearing electron-donating or electron-withdrawing groups on the chromene aromatic ring were tested, the products **3p**–**q** were obtained with excellent enantioselectivities and moderate yields.

**Table 2:**
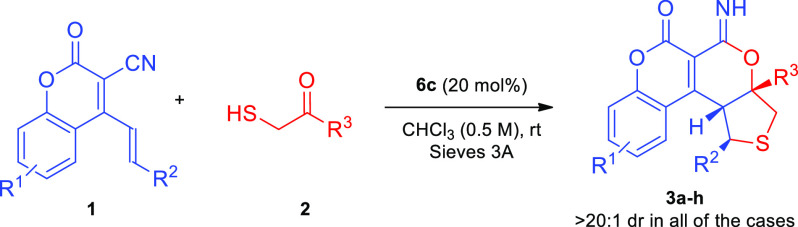
Stereocontrolled γ,δ-Functionalization of 3-Cyano-4-styrylcoumarins *via* Bifunctional Catalysis: Substrate Scope for 3-Cyano-4-alkenyl-2*H*-chromen-2-ones **1** and 2-Mercaptocarbonyl Compounds **2**^a^

**3**	R^1^	R^2^	R^3^	Yield [%]	er^b^
**3a**	H	Ph	Ph	76	98:2
**3b**	H	Ph	2-MeOC_6_H_4_	91	99:1
**3c**	H	Ph	3-MeOC_6_H_4_	82	97:3
**3d**	H	Ph	4-MeC_6_H_4_	59	99:1
**3e**	H	Ph	2-FC_6_H_4_	83	97:3
**3f**	H	Ph	4-FC_6_H_4_	74	96:4
**3g**	H	Ph	4-CF_3_C_6_H_4_	62	97:3
**3h**	H	Ph	2-Naphthyl	51	98:2
**3i**	H	4-MeOC_6_H_4_	Ph	84	99:1
**3j**	H	3-MeC_6_H_4_	Ph	73	98:2
**3k**	H	4-MeC_6_H_4_	Ph	76	98:2
**3l**	H	3-ClC_6_H_4_	Ph	90	97:3
**3m**	H	4-ClC_6_H_4_	Ph	80	99:1
**3n**	H	4-NO_2_C_6_H_4_	Ph	46	99:1
**3o**	H	4-CF_3_C_6_H_4_	Ph	89	95:5
**3p**	9-MeO	Ph	Ph	58	97:3
**3q**	10-Br	Ph	Ph	53	95:5

a Reactions performed on a 0.1 mmol scale using **1a** (1 equiv) and **2** (1.2 equiv) in 0.4 mL of the solvent for 20 h.

b Determined by a chiral stationary phase UPC^2^.

To further demonstrate the synthetic utility of the developed cascade, the transformation of imidoester **3** into lactone **4** was performed (Table 3). Therefore, chiral compounds **3** were hydrolyzed to **4** under acidic conditions and the scope of the method was tested. It was found that all reactions proceeded with satisfactory results providing **4a**–**4h** in moderate to high yields and with the preservation of optical purity introduced in the organocatalytic step. Subsequently, a 1 mmol scale experiment between **1a** and **2a** was carried out under the optimized conditions (Scheme 3). Moreover, the synthesis of compound **4a** was performed in a one-pot fashion, obtaining the corresponding product with a very good outcome. Additionally, the tetrahydro-pyran-2-ylideneamine derivative **3** was subjected to selective Boc protection leading to *N*-protected product **7**.

**Table 3:**
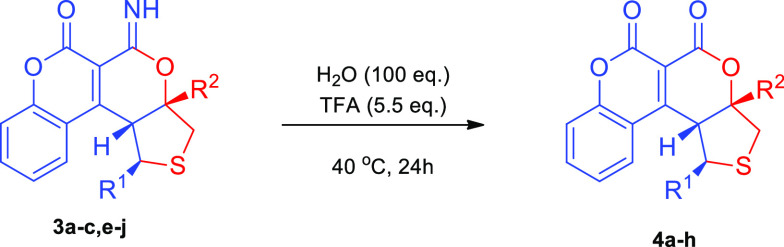
Stereocontrolled γ,δ-Functionalization of 3-Cyano-4-styrylcoumarins *via* Bifunctional Catalysis: Derivatization of Products **3**^a^

**4**	R^1^	R^2^	Yield [%]	er^b^
**4a**	Ph	Ph	70	98:2
**4b**	Ph	2-MeOC_6_H_4_	90	99:1
**4c**	Ph	3-MeOC_6_H_4_	74	97:3
**4d**	Ph	2-FC_6_H_4_	88	97:3
**4e**	Ph	4-CF_3_C_6_H_4_	76	97:3
**4f**	Ph	2-Naphthyl	84	97:3
**4g**	4-MeOC_6_H_4_	Ph	57	99:1
**4h**	3-MeC_6_H_4_	Ph	61	99:1

a Reactions performed on a 0.1 mmol scale using **1a** (1 equiv) and **2** (1.2 equiv) in 0.4 mL of the solvent for 24 h.

b Determined by a chiral stationary phase UPC^2^.

**Scheme 3:**
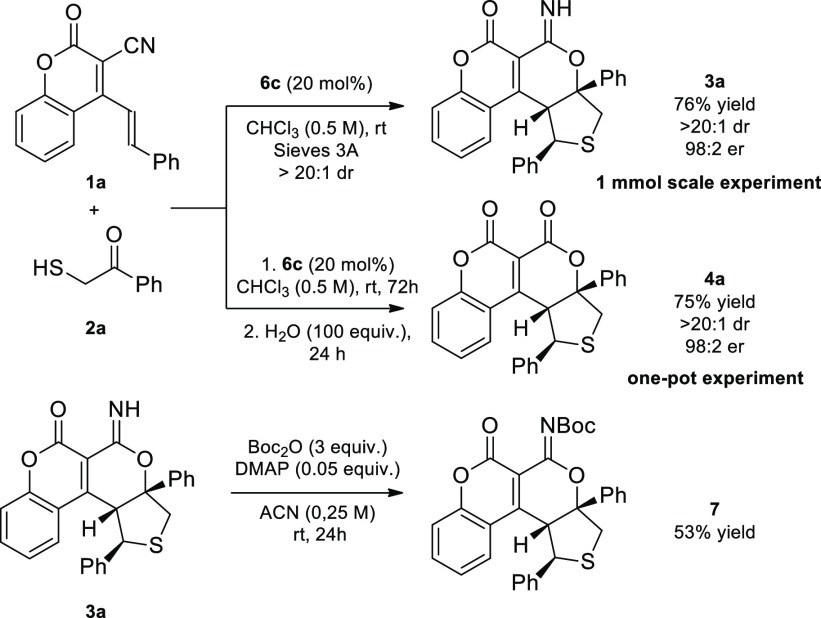
Stereocontrolled γ,δ-Functionalization of 3-Cyano-4-styrylcoumarins *via* Bifunctional Catalysis: Transformation of Product **3a**

The absolute configuration of product **4b** was determined by the single-crystal X-ray analysis (see the Supporting Information for details). Because all products were synthesized under the catalysis of **6c**, the absolute configurations of **3** and **4** were assigned by analogy. Based on the absolute stereochemistry of the final products **3** and **4**, a plausible mechanism of a cascade was proposed (Scheme 4). The reaction is promoted by the bifunctional catalyst **6c**, which is responsible for the independent activation of 2-oxo-4-styryl-2*H*-chromene-3-carbonitrile **1** and 2-mercaptocarbonyl compound **2**. Such a recognition profile by H-bonding and ion-pairing interactions of both reaction partners allows the stereoselectivity of the cascade to be controlled. In the second stage of the cascade, an intramolecular vinylogous aldol reaction takes place, leading to the formation of chiral alkoxide anion **9**, which reacts with the carbon atom of the nitrile group to give enantioenriched product **3**.

**Scheme 4:**
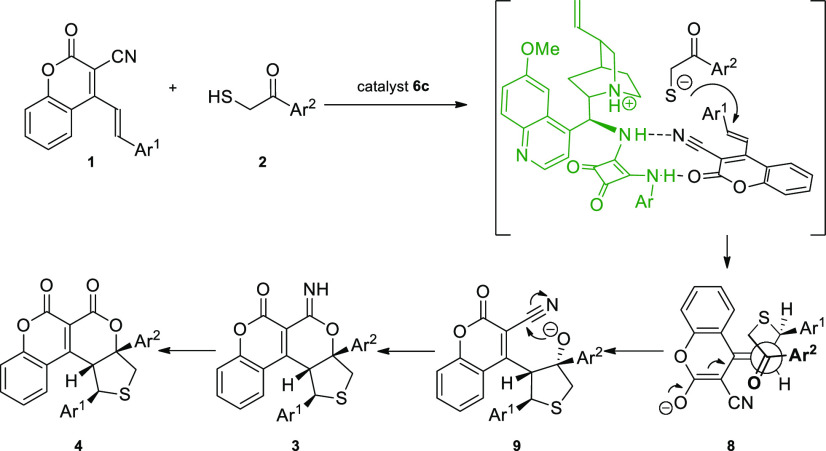
Stereocontrolled γ,δ-Functionalization of 3-Cyano-4-styrylcoumarins *via* Bifunctional Catalysis: Mechanistic Considerations

In conclusion, the stereocontrolled γ,δ-functionalization of 2-oxo-4-styryl-2*H*-chromene-3-carbonitriles with 2-mercaptocarbonyl compounds was established employing bifunctional catalysis. Their reaction proceeded in a vinylogous fashion, providing polycyclic products in a sequence of reaction involving *thia*-Michael addition, followed by the intramolecular aldol and annulation. The developed cascade enables the introduction of 3,4-dihydrocoumarin, δ-lactone, and tetrahydrothiophene scaffolds in one structure, resulting in biologically important products in high yields and stereoselectivities with a broad scope.

## Supporting Information Available

Experimental procedures, characterization of the products, NMR data, and HPLC traces (PDF)
