# Supplementary material for: Organocatalytic Asymmetric Approach to γ,δ-Functionalization of 3-Cyano-4-styrylcoumarins via Bifunctional Catalysis
Source: Org Lett. 2022 Oct 17;24(42):7722–6. doi: 10.1021/acs.orglett.2c02836 (PMC9623593; doi:10.1021/acs.orglett.2c02836)
Supplement: Supplementary file 1 — ol2c02836_si_001.pdf [file ol2c02836_si_001.pdf]

Organocatalytic asymmetric approach to γ,δ-functionalization of 3-cyano-4-styrylcoumarins via bifunctional catalysis

Marta Romaniszyn[a,‡], Anna Skrzyńska[a,‡], Joanna Dybowska[a], Łukasz Albrecht*[a]

Institute of Organic Chemistry
Department of Chemistry,
Lodz University of Technology
Żeromskiego 116, 90-924 Łódź, Poland
e-mail: lukasz.albrecht@p.lodz.pl
http://www.a-teamlab.p.lodz.pl

Contents

1. General methods S2

2. Synthesis of 3-cyano-4-styrylcoumarins **1a-j** S3

3. Synthesis of mercaptocarbonyl compounds **2a-h** S5

4. Enantioselective synthesis of polycyclic coumarin derivatives **3** – general procedure S7

5. Enantioselective synthesis of (1*R*,3a*R*,11c*R*)-5-imino-1,3a-diphenyl-1,3,3a,11c-tetrahydrothieno[3',4':5,6]pyrano[3,4-c]chromen-6(5*H*)-one **3a** on a 1 mmol scale S16

6. Enantioselective synthesis of δ-lactones **4** - general procedure S17

7. Enantioselective synthesis of **4a** – one-pot procedure S22

8. Transformation of **3a** –synthesis of (*Z*)-*tert*-butyl-((1*R*,3a*R*,11c*R*)-6-oxo-1,3a-diphenyl-1,3,3a,11c-tetrahydrothieno[3',4':5,6]pyrano[3,4-c]chromen-5(6*H*)-ylidene)carbamate **7** S23

9. Crystal and X-ray data for (1*R*,3a*R*,11c*R*)-3a-(2-methoxyphenyl)-1-phenyl-1,3,3a,11c-tetrahydrothieno[3',4':5,6]pyrano[3,4-c]chromene-5,6-dione **4b** S24

10. NMR data S26

11. UPC^2 traces S61

1. General methods

NMR spectra were acquired on a Bruker Ultra Shield 700 instrument, running at 700 MHz for ^{1}H and 176 MHz for ^{13}C, respectively. Chemical shifts (δ) are reported in ppm relative to residual solvent signals ($CDCl_3$: 7.26 ppm for ^{1}H NMR, 77.16 ppm for ^{13}C NMR). Mass spectra were recorded on a Bruker Maxis Impact spectrometer using electrospray (ES+) ionization referenced to the mass of the charged species. Optical rotations were measured on a Perkin-Elmer 241 polarimeter and $[a]_D$ values are given in deg•cm•g^{-1}•dm^{-1}; concentration *c* is listed in g•(100 mL)$^{-1}$. Analytical thin layer chromatography (TLC) was performed using pre-coated aluminum-backed plates (Merck Kieselgel 60 F254) and visualized by ultraviolet irradiation or potassium manganate (VII) stain. The enantiomeric ratio (er) of the products was determined by chiral stationary phase UPC^2 (Daicel Chiralpak IA, IB and IG column). Unless otherwise noted, analytical grade solvents and commercially available reagents were used without further purification. For flash chromatography (FC) silica gel (60, 35-70 μm, Merck KGaA).

2. Synthesis of 3-cyano-4-styrylcoumarins 1a-j

3-Cyano-4-styrylcoumarins **1a-j** were synthesized by the reported procedure.[1]

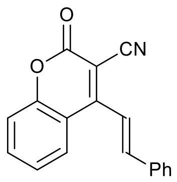

1a

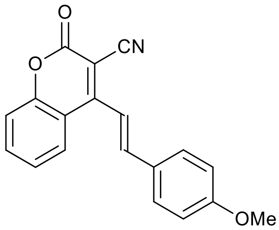

1b

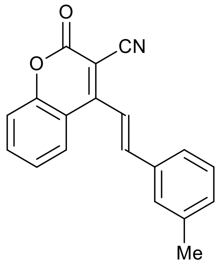

1c

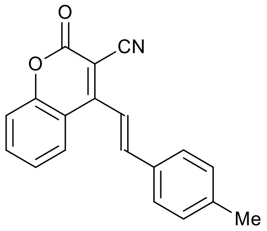

1d

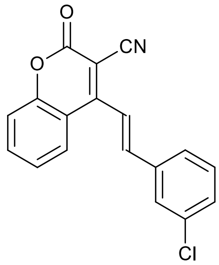

1e

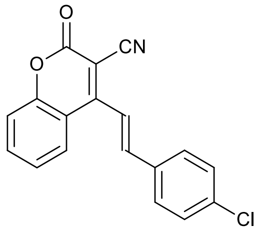

1f

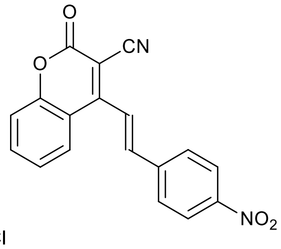

1g

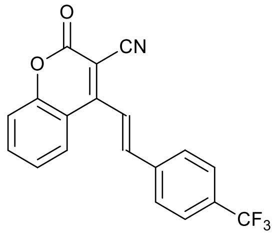

1h

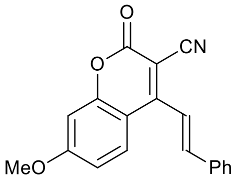

1i

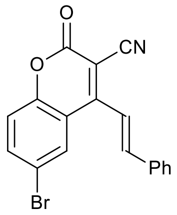

1j

2-Oxo-4-styryl-2*H*-chromene-3-carbonitrile 1a: ^{1}H NMR (700 MHz, $CDCl_3$) δ 7.93 – 7.90 (m, 1H), 7.74 – 7.70 (m, 1H), 7.67 (d, *J* = 8.0 Hz, 1H). 7.67 – 7.64 (m, 2H), 7.49 – 7.45 (m, 3H), 7.45 – 7.43 (m, 1H), 7.43 – 7.39 (m, 1H), 7.36 (d, *J* = 16.4 Hz, 1H).

(*E*)-4-(4-Methoxystyryl)-2-oxo-2*H*-chromene-3-carbonitrile 1b: ^{1}H NMR (700 MHz, $CDCl_3$) δ 7.94 – 7.92 (m, 1H)), 7.72 – 7.67 (m, 1H), 7.69 (d, *J* = 16.3 Hz, 1H), 7.64 – 7.59 (m, 2H), 7.43 – 7.41 (m, 1H), 7.41 – 7.39 (m, 1H), 7.24 (d, *J* = 16.3 Hz, 1H), 7.00 – 6.96 (m, 2H), 3.88 (s, 3H).

(*E*)-4-(3-Methylstyryl)-2-oxo-2*H*-chromene-3-carbonitrile 1c: ^{1}H NMR (700 MHz, $CDCl_3$) δ 7.94 – 7.90 (m, 1H), 7.74 – 7.69 (m, 1H), 7.65 (d, *J* = 16.4 Hz, 1H), 7.48 – 7.45 (m, 2H), 7.44

[1] Romaniszyn, M.; Gronowska, K.; Albrecht, Ł. *Adv. Synth. Catal.* **2021**, *363*, 5116-5121.

– 7.43 (m, 1H), 7.42 – 7.40 (m, 1H), 7.37 – 7.34 (m, 1H), 7.34 (d, *J* = 16.4 Hz, 1H), 7.29 – 7.27 (m, 1H), 2.43 (s, 3H).

(*E*)-4-(4-Methylstyryl)-2-oxo-2*H*-chromene-3-carbonitrile 1d: ^{1}H NMR (700 MHz, $CDCl_3$) δ 7.94-7.90 (m, 1H), 7.77-7.69 (m, 1H), 7.67 (d, *J* = 16.3 Hz, 1H), 7.57 – 7.54 (m, 2H), 7.44-7.42 (m, 1H), 7.43 – 7.38 (m, 1H), 7.32 (d, *J* = 16.3 Hz, 1H), 7.28 – 7.26 (m, 2H), 2.42 (s, 3H).

(*E*)-4-(3-Chlorostyryl)-2-oxo-2*H*-chromene-3-carbonitrile 1e: ^{1}H NMR (700 MHz, $CDCl_3$) δ 7.88 (dd, *J* = 8.0, 1.5 Hz, 1H), 7.74-7.72 (m, 1H), 7.65 – 7.63 (m, 1H), 7.57 (d, *J* = 16.3 Hz, 1H), 7.53 – 7.50 (m, 1H), 7.45 – 7.39 (m, 4H), 7.34 (d, *J* = 16.3 Hz, 1H).

(*E*)-4-(4-Chlorostyryl)-2-oxo-2*H*-chromene-3-carbonitrile 1f: ^{1}H NMR (700 MHz, $CDCl_3$) δ 7.89 – 7.87 (m, 1H), 7.74 – 7.70 (m, 1H), 7.61 (d, *J* = 16.2 Hz, 1H), 7.60 – 7.57 (m, 2H), 7.46 – 7.43 (m, 3H), 7.43 – 7.39 (m, 1H), 7.32 (d, *J* = 16.2 Hz, 1H).

(*E*)-4-(4-Nitrostyryl)-2-oxo-2*H*-chromene-3-carbonitrile 1g: ^{1}H NMR (700 MHz, DMSO-d_6) δ 8.36 – 8.31 (m, 2H), 8.14 (dd, *J* = 8.1, 1.5 Hz, 1H), 8.13 – 8.11 (m, 2H), 7.97 (d, *J* = 16.4 Hz, 1H), 7.85 – 783 (m, 1H), 7.73 (d, *J* = 16.4 Hz, 1H), 7.56 (dd, *J* = 8.4, 1.0 Hz, 1H), 7.52 – 7.49 (m, 1H).

(*E*)-2-Oxo-4-(4-(trifluoromethyl)styryl)-2*H*-chromene-3-carbonitrile 1h: ^{1}H NMR (700 MHz, CD_2Cl_2) δ 7.91-7.87 (m, 1H), 7.83 – 7.78 (m, 2H), 7.78 – 7.73 (m, 3H), 7.63 (d, *J* = 16.4 Hz, 1H), 7.47 – 7.44 (m, 2H), 7.44 (d, *J* = 16.4 Hz, 1H).

(*E*)-7-Methoxy-2-oxo-4-styryl-2*H*-chromene-3-carbonitrile 1i: ^{1}H NMR (700 MHz, $CDCl_3$) δ 7.80 (d, *J* = 9.0 Hz, 1H), 7.65 – 7.63 (m, 2H), 7.60 (d, *J* = 16.4 Hz, 1H), 7.49 – 7.43 (m, 3H), 7.30 (d, *J* = 16.4 Hz, 1H), 6.95 (dd, *J* = 9.0, 2.5 Hz, 1H), 6.87 (d, *J* = 2.5 Hz, 1H).

(*E*)-6-Bromo-2-oxo-4-styryl-2*H*-chromene-3-carbonitrile 1j: ^{1}H NMR (700 MHz, DMSO-d_6) δ 8.38 (d, *J* = 2.3 Hz, 1H), 7.97 (dd, *J* = 8.8, 2.3 Hz, 1H), 7.90 – 7.85 (m, 2H), 7.78 (d, *J* = 16.2 Hz, 1H), 7.69 (d, *J* = 16.2 Hz, 1H), 7.52 – 7.48 (m, 4H).

3. Synthesis of mercaptocarbonyl compounds 2a-h

The marcaptocarbonyl compounds **2a-h** were synthesized by the reported procedure.[2]

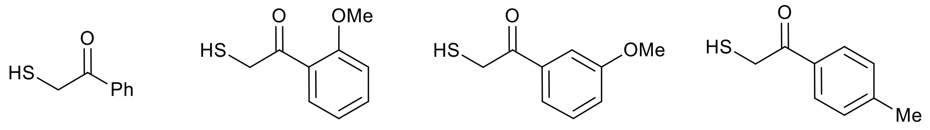

2a **2b** **2c** **2d**

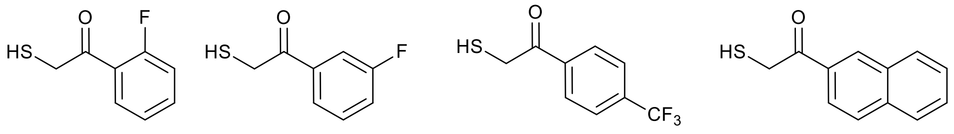

2e **2f** **2g** **2h**

2-Mercapto-1-phenylethanone 2a: ^{1}H NMR (700 MHz, $CDCl_3$) δ 8.07 – 7.75 (m, 2H), 7.61 – 7.58 (m, 1H), 7.55 – 7.42 (m, 2H), 3.96 (d, *J* = 7.4 Hz, 2H), 2.13 (t, *J* = 7.4 Hz, 1H).

2-Mercapto-1-(2-methoxyphenyl)ethenone 2b: ^{1}H NMR (700 MHz, $CDCl_3$) δ 7.82 – 7.81(m, 1H), 7.51 – 7.48 (m,1H), 7.05 – 7.02 (m, 1H), 7.00 – 6.98 (m, 1H), 3.96 (d, *J* = 7.7 Hz, 2H), 3.93 (s, 3H), 2.02 (t, *J* = 7.7 Hz, 1H).

2-Mercapto-1-(3-methoxyphenyl)ethenone 2c: ^{1}H NMR (700 MHz, $CDCl_3$) δ 7.52 – 7.50 (m, 1H), 7.50 – 7.48 (m, 1H), 7.40 – 7.38 (m, 1H), 7.15 – 7.13 (m, 1H), 3.94 (d, *J* = 7.3, 0.5 Hz, 2H), 3.86 (s, 3H), 2.12 (td, *J* = 7.4, 0.5 Hz, 1H).

2-Mercapto-1-(p-tolyl)ethenone 2d: ^{1}H NMR (700 MHz, $CDCl_3$) δ 7.87 – 7.85 (m, 2H), 7.32 – 7.27 (m, 2H), 3.93 (d, *J* = 7.3 Hz, 2H), 2.42 (s, 3H), 2.13 (t, *J* = 7.3 Hz, 1H).

1-(2-Fluorophenyl)-2-mercaptoethanone 2e: ^{1}H NMR (700 MHz, $CDCl_3$) δ 7.95 – 7.92 (m, 1H), 7.58 – 7.54 (m, 1H), 7.31 – 7.22 (m, 1H), 7.18 – 7.14 (m, 1H), 3.94 (dd, *J* = 7.7, 2.3 Hz, 2H), 2.05 (td, *J* = 7.7, 0.9 Hz, 1H).

1-(3-Fluorophenyl)-2-mercaptoethanone 2f: ^{1}H NMR (700 MHz, $CDCl_3$) ^{1}H NMR (700 MHz, $CDCl_3$) δ 7.74 – 7.72 (m, 1H), 7.66 – 7.63 (m, 1H), 7.52 – 7.44 (m, 1H), 7.31 – 7.28 (m, 1H), 4.07 – 3.72 (d, *J* = 7.5 Hz, 2H), 2.12 (t, *J* = 7.5 Hz, 1H).

2-Mercapto-1-(4-(trifluoromethyl)phenyl)ethenone 2g: ^{1}H NMR (700 MHz, $CDCl_3$) δ 8.08 – 8.06 (m, *J* =2H), 7.77 – 7.75 (m, 2H), 3.97 (d, *J* = 7.5 Hz, 2H), 2.12 (t, *J* = 7.5 Hz, 1H).

2-Mercapto-1-(naphthalen-2-yl)ethenone 2h: ^{1}H NMR (700 MHz, $CDCl_3$) δ 8.48 (m, 1H), 8.03 (dd, *J* = 8.6, 1.8 Hz, 1H), 7.98 (dq, *J* = 8.1, 0.7 Hz, 1H), 7.93 (dq, *J* = 8.6, 0.7 Hz, 1H),

[2] Przydacz, A.; Kowalczyk, R.; Albrecht, Ł. *Org. Biomol. Chem.* **2017**, *15*, 9566-9569.

7.89 (dq, *J* = 8.2, 0.6 Hz, 1H), 7.63 (ddd, *J* = 8.2, 6.8, 1.3 Hz, 1H), 7.58 (ddd, *J* = 8.1, 6.8, 1.3 Hz, 1H), 4.09 (d, *J* = 7.4 Hz, 2H), 2.20 (t, *J* = 7.4 Hz, 1H).

4. Enantioselective synthesis of polycyclic coumarin derivatives 3 – general procedure

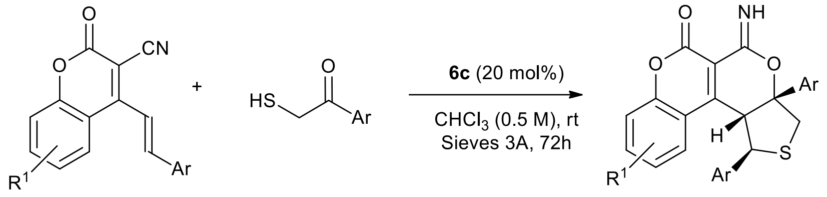

In an ordinary 4 mL glass vial, equipped with a magnetic stirring bar, screw cap, and molecular sieves (3Å), corresponding mercaptocarbonyl compound **2** (1.2 equiv., 0.12 mmol) was dissolved in $CHCl_3$ (0.2 mL). Next, 3-cyano-4-styrylcoumarine **1** (1 equiv. 0.1 mmol) and catalyst **6c** (0.2 equiv., 0.02 mmol, 12.6 mg) were added. The reaction mixture was stirred for 72 h at ambient temperature. After full conversion of the starting material **1** (as confirmed by ^{1}H NMR of a crude reaction mixture), the reaction mixture was directly subjected to flash chromatography on silica gel (eluent: dichlorometane then hexanes/ethyl acetate 4:1) to obtain pure product **3**. The enantiomeric ratio (er) of the products was determined by chiral stationary phase UPC^2. The racemic samples of products **3** for chiral UPC^2 separation studies were prepared using catalyst **6b.**

(1*R*,3a*R*,11c*R*)-5-Imino-1,3a-diphenyl-1,3,3a,11c-tetrahydrothieno[3',4':5,6]pyrano[3,4-c]chromen-6(5*H*)-one 3a

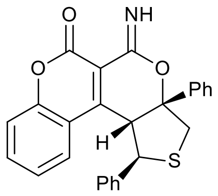

Following the general procedure product **3a** (>20:1 dr in a crude reaction mixture) was isolated (eluent: dichlorometane then hexanes/ethyl acetate 4:1) in 76% (32.4 mg) yield as white solid (mp = 160-162 ^{o}C). **^{1}H NMR** (700MHz, $CDCl_3$) δ 10.50 (bs, 1H), 7.44 – 7.40 (m, 5H), 7.29 – 7.26 (m, 2H), 7.25 – 7.23 (m, 1H), 7.22 – 7.20 (m, 3H), 7.19 – 7.17 (m, 1H), 6.83 – 6.80 (m, 1H), 6.69 – 6.67 (m, 1H), 4.84 (d, *J* = 10.3 Hz, 1H), 4.31 (d, *J* = 10.3 Hz, 1H), 3.77 (d, *J* = 12.4 Hz, 1H), 3.73 (d, *J* = 12.4 Hz, 1H). **^{13}C NMR** (176 MHz, $CDCl_3$) δ 158.4, 158.3, 158.2, 153.4, 152.0, 139.0, 137.8, 134.1, 129.4 (2C), 129.1 (2C), 128.8 (2C), 128.6 (2C), 125.6, 124.9, 124.5, 117.3, 117.1, 109.4, 92.0, 57.8, 54.6, 46.1. The er was determined by UPC^2 using a chiral Chiralpack IA column gradient from 100% CO_2 up to 40%; *i*-PrOH, 2.5 mL/min; τ_{major} = 4.48 min, τ_{minor} = 4.66 min, (98:2 er). $[\alpha]_D^{21}$ = -21.8 (c = 1.0, $CHCl_3$). HRMS (ESI): *m/z* calcd for $C_{26}H_{20}NO_3S$ $[M+H]^+$ 426.1164, found 426.1167.

(1*R*,3a*R*,11c*R*)-5-Imino-3a-(2-methoxyphenyl)-1-phenyl-1,3,3a,11c-tetrahydrothieno[3',4':5,6]pyrano[3,4-c]chromen-6(5*H*)-one 3b

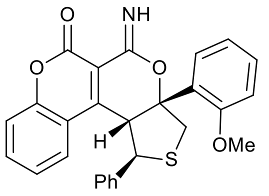

Following the general procedure product **3b** (>20:1 dr in a crude reaction mixture) was isolated (eluent: dichlorometane then hexanes/ethyl acetate 4:1) in 91% (41.4 mg) yield as light-yellow solid (mp =148-150 ^{o}C). **^{1}H NMR** (700MHz, $CDCl_3$) δ 10.44 (bs, 1H), 7.64 – 7.62 (m, 1H), 7.41 – 7.39 (m, 2H), 7.38 – 7.35 (m, 1H), 7.22 – 7.16 (m, 4H), 7.16 – 7.13 (m, 1H), 6.95 – 6.92 (m, 1H), 6.85 – 6.76 (m, 2H), 6.62 – 6.60 (m, 1H), 5.18 (d, *J* = 10.4 Hz, 1H), 4.73 (d, *J* = 10.4 Hz, 1H), 4.13 (d, *J* = 11.9 Hz, 1H), 3.85 (s, 3H), 3.56 (d, *J* = 11.9 Hz, 1H). **^{13}C NMR** (176 MHz, $CDCl_3$) δ 158.7, 158.4, 155.3, 153.5, 153.2, 138.7, 133.8, 130.1, 128.9 (2C), 128.6, 128.5, 128.4 (2C), 126.3, 125.7, 124.2, 121.8, 117.5, 116.9, 111.7, 108.9, 91.9, 58.5, 55.7, 52.4, 43.5. The er was determined by UPC^2 using a chiral Chiralpack IA column gradient from 100% CO_2 up to 40%; *i*-PrOH, 2.5 mL/min; τ_{major} = 4.37 min, τ_{minor} = 4.49 min, (99:1 er); $[\alpha]_D^{21}$ = -53.0 (c = 1.0, $CHCl_3$). HRMS (ESI): *m/z* calcd for $C_{27}H_{22}NO_4S$ $[M+H]^+$ 456.1270, found 456.1273.

(1*R*,3a*R*,11c*R*)-5-Imino-3a-(3-methoxyphenyl)-1-phenyl-1,3,3a,11c-tetrahydrothieno[3',4':5,6]pyrano[3,4-c]chromen-6(5*H*)-one 3c

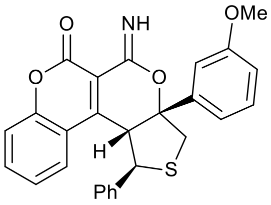

Following the general procedure product **3c** (>20:1 dr in a crude reaction mixture) was isolated (eluent: dichlorometane then hexanes/ethyl acetate 4:1) in 82% (37.4 mg) yield as light-yellow solid (mp =170-172 ^{o}C). **^{1}H NMR** (700MHz, $CDCl_3$) δ 10.45 (bs, 1H), 7.43 – 7.39 (m, 3H), 7.22 – 7.13 (m, 5H), 7.00 – 6.97 (m, 1H), 6.93 – 6.91 (m, 1H), 6.83 – 6.80 (m, 1H), 6.76-6.73 (m, 1H), 6.68 – 6.66 (m, 1H), 4.82 (d, *J* = 10.3 Hz, 1H), 4.28 (d, *J* = 10.3 Hz, 1H), δ 3.76 (d, *J* = 12.5 Hz, 1H), 3.72 (d, *J* = 12.5 Hz, 1H), 3.71 (s, 3H). **^{13}C NMR** (176 MHz, $CDCl_3$) δ 160.3, 158.3, 158.2, 153.4, 152.1, 140.6, 137.8, 134.1, 130.4, 129.0 (2C), 128.8, 128.5 (2C), 125.6, 124.5, 117.3, 117.1, 116.8, 114.1, 111.3, 109.3, 91.9, 57.8, 55.4, 54.6, 46.0. The er was determined by UPC^2 using a chiral Chiralpack IB column gradient from 100% CO_2 up to 40%; *i*-PrOH, 2.5 mL/min; τ_{major}= 4.42 min, τ_{minor}= 4.51 min, (97:3 er); $[\alpha]_D^{21}$ = -27.8 (c = 1.0, $CHCl_3$). HRMS (ESI): *m/z* calcd for $C_{27}H_{22}NO_4S$ $[M+H]^+$ 456.1270, found 456.1273.

(1*R*,3a*R*,11c*R*)-5-Imino-1-phenyl-3a-(*p*-tolyl)-1,3,3a,11c-tetrahydrothieno[3',4':5,6]pyrano[3,4-c]chromen-6(5*H*)-one 3d

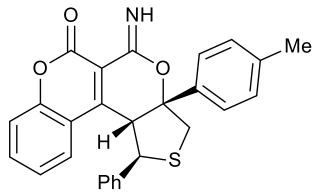

Following the general procedure product **3d** (>20:1 dr in a crude reaction mixture) was isolated in (eluent: dichlorometane then hexanes/ethyl acetate 4:1) 59% (25.8 mg) yield as light-yellow solid (mp =204-206 °C). **^{1}H NMR** (700 MHz, $CDCl_3$) δ 10.43 (bs, 1H), 7.43 – 7.40 (m, 3H), 7.30 – 7.27 (m, 2H), 7.22 – 7.19 (m, 3H), 7.18 – 7.16 (m, 1H), 7.07 – 7.05 (m, 2H), 6.83 – 6.80 (m, 1H), 6.68 – 6.66 (m, 1H), 4.82 (d, *J* = 10.3 Hz, 1H), 4.28 (d, *J* = 10.3 Hz, 1H), 3.75 (d, *J* = 12.4 Hz, 1H), 3.71 (d, *J* = 12.4 Hz, 1H), 2.24 (s, 3H). **^{13}C NMR** (176 MHz, $CDCl_3$) δ 158.4, 158.3, 153.4, 152.2, 138.8, 137.9, 135.9, 134.1, 130.0 (2C), 129.0 (2C), 128.8, 128.6 (2C), 125.6, 124.8 (2C), 124.5, 117.3, 117.1, 109.4, 92.1, 57.8, 54.6, 46.1, 21.1. The er was determined by UPC^2 using a chiral Chiralpack IA column gradient from 100% CO_2 up to 40%; *i*-PrOH, 2.5 mL/min; τ_{major}= 4.56 min, τ_{minor}= 4.72 min, (99:1 er); $[\alpha]_D^{21}$ = -17.4 (c = 1.0, $CHCl_3$). HRMS (ESI): *m/z* calcd for $C_{27}H_{22}NO_3S$ $[M+H]^+$ 440.1320, found 440.1325.

(1*R*,3a*R*,11c*R*)-3a-(2-Fluorophenyl)-5-imino-1-phenyl-1,3,3a,11c-tetrahydrothieno[3',4':5,6]pyrano[3,4-c]chromen-6(5*H*)-one 3e

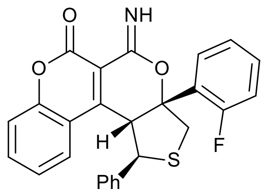

Following the general procedure product **3e** (>20:1 dr in a crude reaction mixture) was isolated (eluent: dichlorometane then hexanes/ethyl acetate 4:1) in 83% (36.6 mg) yield as light-yellow solid (mp =194-196 °C). **^{1}H NMR** (700 MHz, $CDCl_3$) δ 10.49 (bs, 1H), 7.70 – 7.66 (m, 1H), 7.42 – 7.39 (m, 3H), 7.25 – 7.21 (m, 1H), 7.21 – 7.19 (m, 3H), 7.18 – 7.16 (m, 1H), 7.14 – 7.11 (m, 1H), 6.96 – 6.92 (m, 1H), 6.82 – 6.79 (m, 1H), 6.68 – 6.65 (m, 1H), 4.77 (dd, *J* = 10.3, 1.8 Hz, 1H), 4.71 (d, *J* = 10.4 Hz, 1H), 4.00 (d, *J* = 12.3 Hz, 1H), 3.69 (dd, *J* = 12.3, 1.3 Hz, 1H). **^{13}C NMR** (176 MHz, $CDCl_3$) δ 158.8 (d, *J* = 247.1 Hz), 158.3, 158.2, 153.4, 152.6, 137.9, 134.2, 130.8 (d, *J* = 8.6 Hz), 129.0 (2C), 128.9 (d, *J* = 3.1 Hz), 128.8, 128.5 (2C), 125.9, 125.8, (d, *J* = 11 Hz), 125.4 (d, *J* = 3.3 Hz), 124.5, 117.2, 116.9, 116.7 (d, *J* = 22.8 Hz), 108.7, 90.3 (d, *J* = 5.0 Hz), 58.4, 53.6 (d, *J* = 9.5 Hz), 44.2.The er was determined by UPC^2 using a chiral Chiralpack IA column gradient from 100% CO_2 up to 40%; *i*-PrOH, 2.5 mL/min; flow rate 1.0 mL/min; τ_{major}= 4.06 min, τ_{minor}= 4.21 min, (97:3

er); $[\alpha]_D^{21}$ = -11.7 (c = 1.0, $CHCl_3$). HRMS (ESI): *m/z* calcd for $C_{26}H_{19}FNO_3S$ $[M+H]^+$ 444.1070, found 444.1072.

(1*R*,3a*R*,11c*R*)-3a-(4-Fluorophenyl)-5-imino-1-phenyl-1,3,3a,11c-tetrahydrothieno[3',4':5,6]pyrano[3,4-c]chromen-6(5*H*)-one 3f

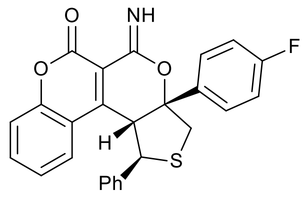

Following the general procedure product **3f** (>20:1 dr in a crude reaction mixture) was isolated (eluent: dichlorometane then hexanes/ethyl acetate 4:1) in 74% (32.8 mg) yield as light-yellow solid (mp =201-203 ^oC). **^{1}H NMR** (700MHz, $CDCl_3$) δ 10.48 (bs, 1H), 7.46 – 7.37 (m, 5H), 7.23 – 7.18 (m, 4H), 6.97 – 6.94 (m, 2H), 6.87 – 6.78 (m, 1H), 6.69 – 6.61 (m, 1H), 4.83 (d, *J* = 10.4 Hz, 1H), 4.26 (d, *J* = 10.4 Hz, 1H), 3.76 (d, *J* = 12.5 Hz, 1H), 3.69 (d, *J* = 12.5 Hz, 1H). **^{13}C NMR** (176 MHz, $CDCl_3$) δ 162.8 (d, *J* = 248.6 Hz), 158.2, 157.9, 153.4, 151.9, 137.7, 134.9 (d, *J* = 3.3 Hz), 134.2, 129.2, 129.1 (2C), 128.9, 128.6 (2C), 126.9 (d, *J* = 8.3 Hz, 2C), 125.5, 124.6, 117.2, 116.4 (d, *J* = 21.7 Hz, 2C), 109.4, 91.6, 57.8, 54.6, 46.1. The er was determined by UPC^2 using a chiral Chiralpack IA column gradient from 100% CO_2 up to 40%; *i*-PrOH, 2.5 mL/min; τ_{major}= 4.25 min, τ_{minor}= 4.64 min, (96:4 er); $[\alpha]_D^{21}$ = -27.4 (c = 1.0, $CHCl_3$). HRMS (ESI): *m/z* calcd for $C_{26}H_{19}FNO_3S$ $[M+H]^+$ 444.1070, found 444.1071.

(1*R*,3a*R*,11c*R*)-5-Imino-1-phenyl-3a-(4-(trifluoromethyl)phenyl)-1,3,3a,11c-tetrahydrothieno[3',4':5,6]pyrano[3,4-c]chromen-6(5*H*)-one 3g

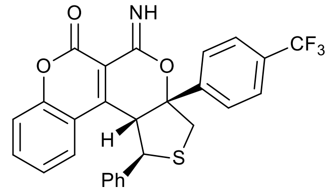

Following the general procedure product **3g** (>20:1 dr in a crude reaction mixture) was isolated (eluent: dichlorometane then hexanes/ethyl acetate 4:1) in 62% (30.4 mg) yield as light-yellow solid (mp =163-165 ^oC). **^{1}H NMR** (700MHz, $CDCl_3$) δ 10.57 (bs, 1H), 7.58 – 7.52 (m, 4H), 7.46 – 7.41 (m, 3H), 7.25 – 7.18 (m, 4H), 6.85 – 6.82 (m, 1H), 6.67 – 6.65 (m, 1H), 4.86 (d, *J* = 10.2 Hz, 1H), 4.31 (d, *J* = 10.2 Hz, 1H), 3.77 (d, *J* = 12.5 Hz, 1H), 3.70 (d, *J* = 12.5 Hz, 1H). **^{13}C NMR** (176 MHz, $CDCl_3$) δ 158.1, 157.8, 153.4, 151.6, 143.0, 137.3, 134.4, 131.2 (q, *J* = 32.8 Hz), 129.1 (2C), 129.0, 128.5 (2C), 126.5 (q, *J* = 3.6 Hz, 2C), 125.5, 125.4 (2C), 124.6, 123.5 (q, *J* = 272.3 Hz), 117.2, 117.0, 109.2, 91.4, 57.9, 54.6, 45.9. The er was determined by UPC^2 using a chiral Chiralpack IA column gradient from 100% CO_2 up to 40%; *i*-PrOH, 2.5 mL/min; τ_{major}= 3.91 min, τ_{minor}= 4.23 min,

(97:3 er); $[\alpha]_D^{21}$ = -24.4 (c = 1.0, $CHCl_3$). HRMS (ESI): *m/z* calcd for $C_{27}H_{19}F_3NO_3S$ $[M+H]^+$ 494.1038, found 494.1037.

(1*R*,3a*R*,11c*R*)-5-Imino-3a-(naphthalen-2-yl)-1-phenyl-1,3,3a,11c-tetrahydrothieno[3',4':5,6]pyrano[3,4-c]chromen-6(5*H*)-one 3h

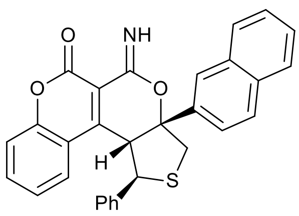

Following the general procedure product **3h** (>20:1 dr in a crude reaction mixture) was isolated (eluent: dichlorometane then hexanes/ethyl acetate 4:1) in 51% (24.2 mg) yield as light-yellow solid (mp =186-188 ^oC). **^{1}H NMR** (700MHz, $CDCl_3$) δ 10.56 (bs, 1H), 7.93 – 7.91 (m, 1H), 7.81 – 7.70 (m, 3H), 7.50 – 7.42 (m, 5H), 7.44 – 7.36 (m, 1H), 7.27 – 7.20 (m, 3H), 7.15 – 7.13 (m, 1H), 6.85 – 6.82 (m, 1H), 6.75 – 6.73 (m, 1H), 4.89 (d, *J* = 10.3 Hz, 1H), 4.44 (d, *J* = 10.3 Hz, 1H), 3.85 – 3.81 (m, 2H). **^{13}C NMR** (176 MHz, $CDCl_3$) δ 158.2, 153.4, 151.9, 137.7, 136.2, 134.1, 133.2, 133.1, 129.4 (2C), 129.1 (2C), 128.9, 128.6 (2C), 128.5, 127.6, 127.1, 127.0, 125.5, 124.8, 124.5, 122.0, 117.3, 117.1, 109.3, 92.1, 58.0, 54.8, 46.0. The er was determined by UPC^2 using a chiral Chiralpack IA column gradient from 100% CO_2 up to 40%; *i*-PrOH, 2.5 mL/min; τ_{major}= 5.04 min, τ_{minor}= 5.28 min, (98:2 er); $[\alpha]_D^{21}$ = -28.6 (c = 1.0, $CHCl_3$). HRMS (ESI): *m/z* calcd for $C_{30}H_{22}NO_3S$ $[M+H]^+$ 476.1320, found 476.1321.

(1*R*,3a*R*,11c*R*)-5-Imino-1-(4-methoxyphenyl)-3a-phenyl-1,3,3a,11c-tetrahydrothieno[3',4':5,6]pyrano[3,4-c]chromen-6(5*H*)-one 3i

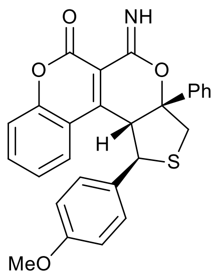

Following the general procedure product **3i** (>20:1 dr in a crude reaction mixture) was isolated (eluent: dichlorometane then hexanes/ethyl acetate 4:1) in 84% (38.4 mg) yield as light-yellow solid (mp =196-198 ^oC). **^{1}H NMR** (700MHz, $CDCl_3$) δ 10.48 (bs, 1H), 7.46 – 7.42 (m, 1H), 7.42 – 7.39 (m, 2H), 7.35 – 7.32 (m, 2H), 7.28 – 7.25 (m, 2H), 7.24 – 7.20 (m, 1H), 7.19 – 7.17 (m, 1H), 6.90 – 6.87 (m, 1H), 6.78 – 6.76 (m, 1H), 6.75 – 6.72 (m, 2H), 4.82 (d, *J* = 10.3 Hz, 1H), 4.28 (d, *J* = 10.3 Hz, 1H), 3.75 (d, *J* = 12.4 Hz, 1H), 3.73 (s, 3H), 3.70 (d, *J* = 12.4 Hz, 1H). **^{13}C NMR** (176 MHz, $CDCl_3$) δ 160.1, 158.3, 158.1, 153.4, 152.2, 139.1, 134.1, 129.6 (2C), 129.4, 129.3 (2C), 128.8, 125.8, 124.9 (2C), 124.5, 117.3, 117.1, 114.4 (2C), 109.4, 91.9, 57.4, 55.6, 54.6, 46.0. The er was determined by UPC^2 using a chiral Chiralpack IA column gradient from 100% CO_2 up to 40%; *i*-PrOH, 2.5 mL/min; τ_{major}=

4.69 min, τ_{minor}= 4.88 min, (99:1 er); $[\alpha]_D^{21}$ = -103.8 (c = 1.0, $CHCl_3$). HRMS (ESI): *m/z* calcd for $C_{27}H_{22}NO_4S$ $[M+H]^+$ 456.1270, found 456.1276.

(1*R*,3a*R*,11c*R*)-5-Imino-3a-phenyl-1-(*m*-tolyl)-1,3,3a,11c-tetrahydrothieno[3',4':5,6]pyrano[3,4-c]chromen-6(5*H*)-one 3j

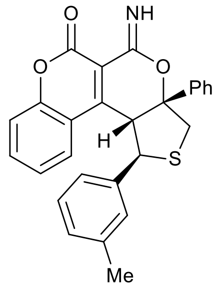

Following the general procedure product **3j** (>20:1 dr in a crude reaction mixture) was isolated (eluent: dichlorometane then hexanes/ethyl acetate 4:1) in 73% (32 mg) yield as light-yellow solid (mp =182-184 °C). **^{1}H NMR** (700MHz, $CDCl_3$) δ 10.49 (bs, 1H), 7.44 – 7.41 (m, 3H), 7.28 – 7.25 (m, 2H), 7.25 – 7.21 (m, 2H), 7.20 – 7.16 (m, 2H), 7.11 – 7.08 (m, 1H), 7.03 – 7.00 (m, 1H), 6.85 – 6.82 (m, 1H), 6.70 – 6.68 (m, 1H), 4.80 (d, *J* = 10.3 Hz, 1H), 4.30 (d, *J* = 10.3 Hz, 1H), 3.76 (d, *J* = 12.4 Hz, 1H), 3.72 (d, *J* = 12.4 Hz, 1H), 2.20 (s, 3H). **^{13}C NMR** (176 MHz, $CDCl_3$) δ 158.3, 158.2, 153.4, 152.1, 139.0, 138.8, 137.7, 134.0, 129.5, 129.3 (2C), 129.2, 129.0, 128.8, 125.8, 125.5, 124.9 (2C), 124.4, 117.4, 117.0, 109.3, 92.0, 57.8, 54.5, 46.1, 21.3. The er was determined by UPC^2 using a chiral Chiralpack IA column gradient from 100% CO_2 up to 40%; *i*-PrOH, 2.5 mL/min; τ_{major}= 4.22 min, τ_{minor}= 4.34 min, (98:2 er); $[\alpha]_D^{21}$ = -36.6 (c = 1.0, $CHCl_3$). HRMS (ESI): *m/z* calcd for $C_{27}H_{22}NO_3S$ $[M+H]^+$ 440.1320, found 440.1322.

(1*R*,3a*R*,11c*R*)-5-Imino-3a-phenyl-1-(*p*-tolyl)-1,3,3a,11c-tetrahydrothieno[3',4':5,6]pyrano[3,4-c]chromen-6(5*H*)-one 3k

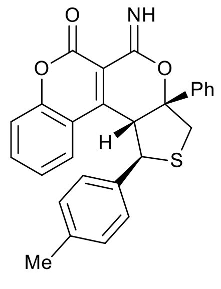

Following the general procedure product **3k** (>20:1 dr in a crude reaction mixture) was isolated (eluent: dichlorometane then hexanes/ethyl acetate 4:1) in 76% (33.4 mg) yield as light-yellow solid (mp =174-176 °C). **^{1}H NMR** (700MHz, $CDCl_3$) δ 10.48 (bs, 1H), 7.45 – 7.39 (m, 3H), 7.31 – 7.25 (m, 4H), 7.24 – 7.21 (m, 1H), 7.19 – 7.17 (m, 1H), 7.03 – 7.00 (m, 2H), 6.85 – 6.82 (m, 1H), 6.73 – 6.71 (m, 1H), 4.82 (d, *J* = 10.3 Hz, 1H), 4.29 (d, *J* = 10.3 Hz, 1H), 3.76 (d, *J* = 12.5 Hz, 1H), 3.71 (d, *J* = 12.5 Hz, 1H), 2.28 (s, 3H). **^{13}C NMR** (176 MHz, $CDCl_3$) δ 158.4, 158.1, 153.4, 152.2, 139.1, 138.8, 134.6, 134.0, 129.6 (2C), 129.3 (2C), 128.8, 128.4 (2C), 125.8, 124.9 (2C), 124.4, 117.3, 117.0, 110.5, 92.0, 57.6, 54.6, 46.1, 21.2. The er was determined by UPC^2 using a chiral Chiralpack IA column gradient from 100% CO_2 up to 40%; *i*-PrOH, 2.5 mL/min; τ_{major}= 4.50 min, τ_{minor}= 4.60 min, (98:2 er);

$[\alpha]_D^{21}$ = -66.4 (c = 1.0, $CHCl_3$). HRMS (ESI): *m/z* calcd for $C_{27}H_{22}NO_4S$ $[M+H]^+$ 440.1320, found 440.1323.

(1*R*,3a*R*,11c*R*)-1-(3-Chlorophenyl)-5-imino-3a-phenyl-1,3,3a,11c-tetrahydrothieno[3',4':5,6]pyrano[3,4-c]chromen-6(5*H*)-one 3l

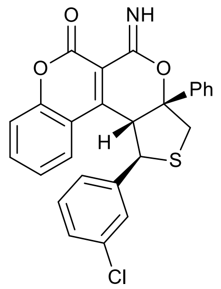

Following the general procedure product **3l** (>20:1 dr in a crude reaction mixture) was isolated (eluent: dichlorometane then hexanes/ethyl acetate 4:1) in 90% (41.4 mg) yield as light-yellow solid (mp =188-190 °C). **^{1}H NMR** (700MHz, $CDCl_3$) δ 10.52 (bs, 1H), 7.57 – 7.54 (m, 1H), 7.47 – 7.44 (m, 1H), 7.44 – 7.39 (m, 2H), 7.30 – 7.25 (m, 2H), 7.26 – 7.16 (m, 4H), 7.12 – 7.07 (m, 1H), 6.93 – 6.90 (m, 1H), 6.74 – 6.72 (m, 1H), 4.79 (d, *J* = 10.3 Hz, 1H), 4.29 (d, *J* = 10.3, 1H), 3.77 (d, *J* = 12.4 Hz, 1H), 3.72 (d, *J* = 12.4 Hz, 1H). **^{13}C NMR** (176 MHz, $CDCl_3$) 158.2, 153.5, 151.5, 145.7, 140.1, 138.7, 135.1, 134.3, 130.2, 129.4 (2C), 129.0, 129.0, 128.5, 126.9, 125.3, 124.9 (2C), 124.6, 117.3, 117.1, 109.5, 91.9, 57.2, 54.3, 46.1. The er was determined by UPC^2 using a chiral Chiralpack IG column gradient from 100% CO_2 up to 40%; *i*-PrOH, 2.5 mL/min; τ_{major}= 5.90 min, τ_{minor}= 6.11 min, (97:3 er); $[\alpha]_D^{21}$ = -52.1 (c = 1.0, $CHCl_3$). HRMS (ESI): *m/z* calcd for $C_{26}H_{19}ClNO_3S$ $[M+H]^+$ 460.0696, found 460.0770.

(1*R*,3a*R*,11c*R*)-1-(4-Chlorophenyl)-5-imino-3a-phenyl-1,3,3a,11c-tetrahydrothieno[3',4':5,6]pyrano[3,4-c]chromen-6(5*H*)-one 3m

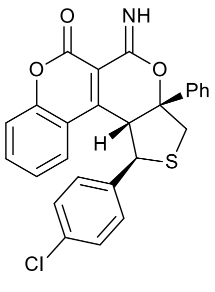

Following the general procedure product **3m** (>20:1 dr in a crude reaction mixture) was isolated (eluent: dichlorometane then hexanes/ethyl acetate 4:1) in 80% (36.6 mg) yield as light-yellow solid (mp =190-192 °C). **^{1}H NMR** (700MHz, $CDCl_3$) δ 10.51 (bs, 1H), 7.49 – 7.46 (m, 1H), 7.42 – 7.39 (m, 2H), 7.39 – 7.36 (m, 2H), 7.29 – 7.26 (m, 2H), 7.25 – 7.23 (m, 1H), 7.22 – 7.20 (m, 1H), 7.21 – 7.18 (m, 2H), 6.93 – 6.91 (m, 1H), 6.72 – 6.70 (m, 1H), 4.81 (d, *J* = 10.3 Hz, 1H), 4.27 (d, *J* = 10.3 Hz, 1H), 3.77 (d, *J* = 12.4 Hz, 1H), 3.71 (d, *J* = 12.4 Hz, 1H). **^{13}C NMR** (176 MHz, $CDCl_3$) δ 161.9, 158.1, 153.5, 151.5, 138.8, 136.4, 134.7, 134.4, 129.9 (2C), 129.4 (2C), 129.2 (2C), 128.9, 125.4, 124.9 (2C), 124.6, 117.3, 117.1, 109.4, 91.9, 57.1, 54.4, 46.0. The er was determined by UPC^2 using a chiral Chiralpack IA column gradient from 100% CO_2 up to 40%; *i*-PrOH, 2.5 mL/min; τ_{major}= 4.82 min, τ_{minor}= 4.96 min,

(99:1 er); $[\alpha]_D^{21}$ = -79.7 (c = 1.0, $CHCl_3$). HRMS (ESI): *m/z* calcd for $C_{26}H_{19}ClNO_3S$ $[M+H]^+$ 460.0696, found 460.0775.

(1*R*,3a*R*,11c*R*)-5-Imino-1-(4-nitrophenyl)-3a-phenyl-1,3,3a,11c-tetrahydrothieno[3',4':5,6]pyrano[3,4-c]chromen-6(5*H*)-one 3n

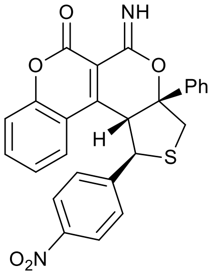

Following the general procedure product **3n** (>20:1 dr in a crude reaction mixture) was isolated (eluent: dichlorometane then hexanes/ethyl acetate 4:1) in 46% (22.2 mg) yield as light-yellow solid (mp =166-168 ^oC). **^{1}H NMR** (700MHz, $CDCl_3$) δ 10.57 (bs, 1H), 8.11 – 8.06 (m, 2H), 7.66 – 7.62 (m, 2H), 7.48 – 7.45 (m, 1H), 7.44 – 7.39 (m, 2H), 7.31 – 7.28 (m, 2H), 7.27 – 7.25 (m, 1H), 7.24 – 7.22 (m, 1H), 6.86 – 6.64 (m, 1H), 6.68 – 6.66 (m, 1H), 4.92 (d, *J* = 10.3 Hz, 1H), 4.33 (d, *J* = 10.3, 1H), 3.82 (d, *J* = 12.5 Hz, 1H), 3.76 (d, *J* = 12.5 Hz, 1H). **^{13}C NMR** (176 MHz, $CDCl_3$) δ 153.5, 150.8, 148.1, 145.6, 144.6, 143.9, 138.3, 134.7, 129.7 (2C), 129.5 (2C), 129.1, 124.9 (2C), 124.7, 124.7, 124.2 (2C), 117.6, 116.8, 109.6, 92.0, 57.0, 54.0, 46.2. The er was determined by UPC^2 using a chiral Chiralpack IB column gradient from 100% CO_2 up to 40%; *i*-PrOH, 2.5 mL/min; τ_{major}= 5.23 min, τ_{minor}= 4.94 min, (99:1 er); $[\alpha]_D^{21}$ = -86.8 (c = 1.0, $CHCl_3$). HRMS (ESI): *m/z* calcd for $C_{26}H_{19}N_2O_5S$ $[M+H]^+$ 471.1015, found 471.1011.

(1*R*,3a*R*,11c*R*)-5-Imino-3a-phenyl-1-(4-(trifluoromethyl)phenyl)-1,3,3a,11c-tetrahydrothieno[3',4':5,6]pyrano[3,4-c]chromen-6(5*H*)-one 3o

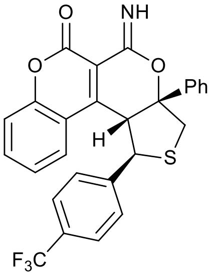

Following the general procedure product **3o** (>20:1 dr in a crude reaction mixture) was isolated (eluent: dichlorometane then hexanes/ethyl acetate 4:1) in 89% (43.8 mg) yield as light-yellow solid (mp =157-159 ^oC). **^{1}H NMR** (700MHz, $CDCl_3$) δ 10.54 (bs, 1H), 7.58 – 7.55 (m, 2H), 7.49 – 7.46 (m, 2H), 7.46 – 7.44 (m, 1H), 7.42 – 7.39 (m, 2H), 7.29 – 7.26 (m, 2H), 7.25 – 7.23 (m, 1H), 7.22 – 7.20 (m, 1H), 6.83 – 6.80 (m, 1H), 6.59 – 6.57 (m, 1H), 4.88 (d, *J* = 10.3 Hz, 1H), 4.28 (d, *J* = 10.3 Hz, 1H), 3.81 (d, *J* = 12.4 Hz, 1H), 3.75 (d, *J* = 12.4 Hz, 1H). **^{13}C NMR** (176 MHz, $CDCl_3$) δ 158.1, 157.9, 153.4, 151.2, 142.3, 138.5, 134.4, 131.2 (q, *J* = 32.8 Hz), 129.4 (2C), 129.1 (2C), 129.0, 126.0 (q, *J* = 3.7 Hz, 2C), 125.0, 124.9 (2C), 124.5, 123.8 (q, *J* = 272.2 Hz), 117.4, 117.0, 109.5, 91.9, 57.2, 54.4, 46.1. The er was determined by UPC^2 using a chiral Chiralpack IB column gradient from 100% CO_2 up to 40%;

i-PrOH, 2.5 mL/min; τ_{major}= 3.88 min, τ_{minor}= 3.78 min, (95:5 er); $[\alpha]_D^{21}$ = -21.1 (c = 1.0, $CHCl_3$). HRMS (ESI): *m/z* calcd for $C_{27}H_{19}F_3NO_3S$ $[M+H]^+$ 494.1038, found 494.1037.

(1*R*,3a*R*,11c*R*)-5-Imino-9-methoxy-1,3a-diphenyl-1,3,3a,11c-tetrahydrothieno[3',4':5,6]pyrano[3,4-c]chromen-6(5*H*)-one 3p

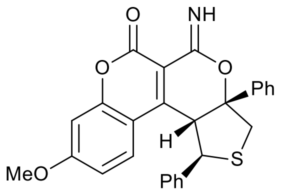

Following the general procedure product **3p** (>20:1 dr in a crude reaction mixture) was isolated (eluent: dichlorometane then hexanes/ethyl acetate 4:1) in 58% (26.4 mg) yield as light-yellow solid (mp =186-188 °C). **^{1}H NMR** (700MHz, $CDCl_3$) δ 10.32 (bs, 1H), 7.44 – 7.39 (m, 4H), 7.28 – 7.25 (m, 2H), 7.24 – 7.21 (m, 4H), 6.63 – 6.61 (m, 1H), 6.55 – 6.63 (m, 1H), 6.38 – 6.36 (m, 1H), 4.82 (d, *J* = 10.3 Hz, 1H), 4.22 (d, *J* = 10.3 Hz, 1H), 3.79 (s, 3H), 3.75 (d, *J* = 12.4 Hz, 1H), 3.70 (d, *J* = 12.4 Hz, 1H). **^{13}C NMR** (176 MHz, $CDCl_3$) δ 164.7, 158.8, 158.5, 155.6, 151.9, 139.1, 138.0, 129.3 (2C), 129.1 (2C), 128.8, 128.8, 128.6 (2C), 126.9, 124.9 (2C), 113.5, 110.9, 106.0, 100.4, 91.8, 57.7, 56.1, 54.8, 46.1. The er was determined by UPC^2 using a chiral Chiralpack IA column gradient from 100% CO_2 up to 40%; *i*-PrOH, 2.5 mL/min; τ_{major}= 4.72 min, τ_{minor}= 4.96 min, (97:3 er); $[\alpha]_D^{21}$ = -36.3 (c = 1.0, $CHCl_3$). HRMS (ESI): *m/z* calcd for $C_{27}H_{22}NO_4S$ $[M+H]^+$ 456.1270, found 456.1272.

(1*R*,3a*R*,11c*R*)-10-Bromo-5-imino-1,3a-diphenyl-1,3,3a,11c-tetrahydrothieno[3',4':5,6]pyrano[3,4-c]chromen-6(5*H*)-one 3q

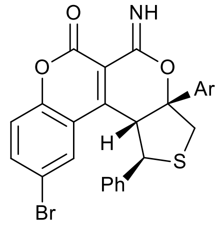

Following the general procedure product **3q** (>20:1 dr in a crude reaction mixture) was isolated (eluent: dichlorometane then hexanes/ethyl acetate 4:1) in 53% (26.2 mg) yield as light-yellow solid (mp =220-222 °C). **^{1}H NMR** (700MHz, $CDCl_3$) δ 10.53 (bs, 1H), 7.50 – 7.48 (m 1H), 7.47 – 7.43 (m, 2H), 7.43 – 7.40 (m, 2H), 7.33 – 7.26 (m, 5H), 7.27 – 7.24 (m, 1H), 7.06 – 7.04 (m, 1H), 6.75 – 6.73 (m, 1H), 4.82 (d, *J* = 10.3 Hz, 1H), 4.20 (d, *J* = 10.3, 1H), 3.78 (d, *J* = 12.6 Hz, 1H), 3.73 (d, *J* = 12.6 Hz, 1H). **^{13}C NMR** (176 MHz, $CDCl_3$) δ 157.6, 152.1, 151.0, 138.8, 137.5, 136.9, 129.5 (2C), 129.5, 129.3 (2C), 129.0, 128.3 (2C), 128.3, 124.9 (2C), 124.7, 119.0, 118.7, 118.7, 117.7, 92.0, 57.8, 55.0, 46.1. The er was determined by UPC^2 using a chiral Chiralpack IA column gradient from 100% CO_2 up to 40%; *i*-PrOH, 2.5 mL/min; τ_{major}= 4.54 min, τ_{minor}= 4.77 min, (95:5 er); $[\alpha]_D^{21}$ = -54.5 (c = 1.0, $CHCl_3$). HRMS (ESI): *m/z* calcd for $C_{26}H_{19}BrNO_3S$ $[M+H]^+$ 504.0269, found 504.0273.

5. Enantioselective synthesis of (1*R*,3a*R*,11c*R*)-5-imino-1,3a-diphenyl-1,3,3a,11c-tetrahydrothieno[3',4':5,6]pyrano[3,4-c]chromen-6(5*H*)-one 3a on a 1 mmol scale

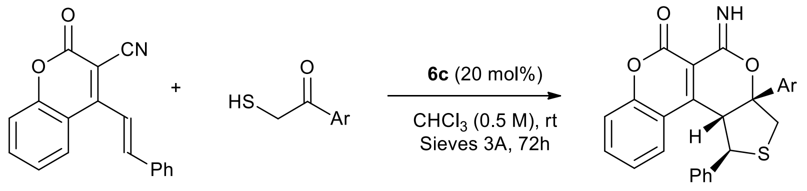

1a **2a** **3a**

In an ordinary 4 mL glass vial, equipped with a magnetic stirring bar, screw cap, and molecular sieves (3Å), 2-mercapto-1-phenylethanone **2a** (1.2 equiv., 1.2 mmol, 182.7 mg) was dissolved in $CHCl_3$ (2 mL). Subsequently, (*E*)-2-oxo-4-styryl-2*H*-chromene-3-carbonitrile **1a** (1 equiv. 1 mmol, 273.3 mg) and catalyst **6c** (0.2 equiv., 0.2 mmol, 126 mg) were added. The reaction mixture was stirred for 72 h at ambient temperature. After full conversion of the starting material **1a** (as confirmed by ^{1}H NMR of a crude reaction mixture), the reaction mixture was directly subjected to flash chromatography on silica gel (eluent: dichlorometane then hexanes/ethyl acetate 4:1) to obtain pure product **3a** in 76% (323.4 mg) yield as white solid (mp.=160-162 ^{o}C). Spectral data were in accordance with the data reported above. The er was determined by UPC^2 using a chiral Chiralpack IA column gradient from 100% CO_2 up to 40%; *i*-PrOH, 2.5 mL/min; τ_{major} = 4.48 min, τ_{minor} = 4.66 min, (98:2 er). $[\alpha]_D^{21}$ = -21.8 (c = 1.0, $CHCl_3$). HRMS (ESI): *m/z* calcd for $C_{26}H_{20}NO_3S$ $[M+H]^+$ 426.1164, found 426.1167.

6. Enantioselective synthesis of δ-lactones 4 - general procedure

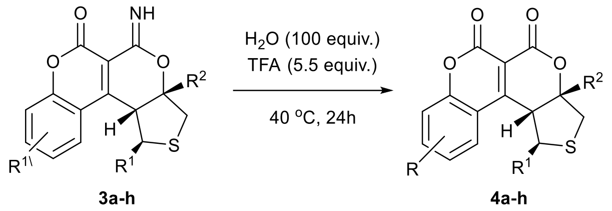

An ordinary 4 mL glass vial equipped with a magnetic stirring bar and screw cap was charged with **3** (0.1 mmol), H_2O (0.2 mL, 100 equiv.) and TFA (40 μL, 5.4 equiv.) and stirred for 24 h at 40 °C in a heating block. Reaction mixture was then transferred to a separatory funnel, diluted with water (5 mL), and extracted with $CHCl_3$ (3 x 10 mL). Organic layer was dried with anhydrous magnesium sulfate, filtered, evaporated with small amount of silica gel to dryness and subjected to flash chromatography on silica gel (eluent: hexanes/ethyl acetate 4:1) to obtain pure product **4**.

(1*R*,3a*R*,11c*R*)-1,3a-Diphenyl-1,3,3a,11c-tetrahydrothieno[3',4':5,6]pyrano[3,4-c]chromene-5,6-dione 4a

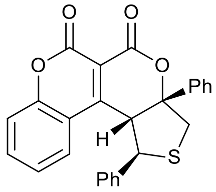

Following the general procedure product **4a** (>20:1 dr in a crude reaction mixture) was isolated (eluent: hexanes/ethyl acetate 4:1) in 70% (30 mg) yield as light-yellow solid (mp =158-160 °C). **^{1}H NMR** (700MHz, $CDCl_3$) δ 7.48 – 7.45 (m, 1H), 7.44 – 7.41 (m, 2H), 7.38 – 7.36 (m, 2H), 7.32 – 7.28 (m, 2H), 7.28 – 7.26 (m, 1H), 7.25 – 7.22 (m, 3H), 7.18 – 7.16 (m, 1H), 6.82 – 6.80 (m, 1H), 6.64 – 6.62 (m, 1H), 4.81 (d, *J* = 10.3 Hz, 1H), 4.32 (dd, *J* = 10.3, 0.7 Hz, 1H), 3.80 – 3.75 (m, 2H). **^{13}C NMR** (176 MHz, $CDCl_3$) δ 158.5, 158.3, 155.0, 154.9, 138.0, 137.4, 135.4, 129.5 (2C), 129.3, 129.2 (2C), 129.1, 128.4 (2C), 125.8, 124.6 (2C), 124.4, 117.4, 116.5, 110.6, 94.7, 58.2, 54.8, 46.2.The er was determined by UPC^2 using a chiral Chiralpack IB column gradient from 100% CO_2 up to 40%; *i*-PrOH, 2.5 mL/min; τ_{major}= 4.57 min, τ_{minor}= 4.88 min, (98:2 er); $[\alpha]_D^{21}$ = -83.4 (c = 1.0, $CHCl_3$). HRMS (ESI): *m/z* calcd for $C_{26}H_{19}O_4S$ $[M+H]^+$ 427.1004, found 427.1012.

(1*R*,3a*R*,11c*R*)-3a-(2-Methoxyphenyl)-1-phenyl-1,3,3a,11c-tetrahydrothieno[3',4':5,6]pyrano[3,4-c]chromene-5,6-dione 4b

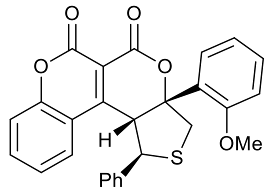

Following the general procedure product **4b** (>20:1 dr in a crude reaction mixture) was isolated (eluent: hexanes/ethyl acetate 4:1) in 90% (41.0 mg) yield as light-yellow solid (mp =135-137 °C). **^{1}H NMR** (700MHz, $CDCl_3$) δ 7.48 – 7.46 (m, 1H), 7.43 – 7.39 (m, 3H), 7.26 – 7.24 (m, 1H), 7.24 – 7.21 (m, 3H), 7.15 – 7.13 (m, 1H), 6.98 – 6.94 (m, 1H), 6.84 – 6.82 (m, 1H), 6.78 – 6.75 (m, 1H), 6.55 – 6.53 (m, 1H), 5.17 (dd, *J* = 10.4, 0.5 Hz, 1H), 4.72 (d, *J* = 10.4 Hz, 1H), 4.20 (d, *J* = 12.1 Hz, 1H), 3.87 (s, 3H), 3.55 (d, *J* = 12.1 Hz, 1H). **^{13}C NMR** (176 MHz, $CDCl_3$) δ 159.5, 159.0, 155.4, 155.1, 154.9, 138.4, 135.0, 130.5, 129.1 (2C), 128.7, 128.4 (2C), 128.0, 125.9, 125.1, 124.1, 122.0, 117.3, 116.8, 111.9, 110.2, 94.7, 58.8, 55.8, 52.5, 43.5. The er was determined by UPC^2 using a chiral Chiralpack IA column gradient from 100% CO_2 up to 40%; *i*-PrOH, 2.5 mL/min; τ_{major}= 4.65 min, τ_{minor}= 4.77 min, (99:1 er); $[\alpha]_D^{21}$ = -10.7 (c = 1.0, $CHCl_3$). HRMS (ESI): *m/z* calcd for $C_{27}H_{21}O_5S$ $[M+H]^+$ 457.1110, found 457.1108.

(1*R*,3a*R*,11c*R*)-3a-(3-Methoxyphenyl)-1-phenyl-1,3,3a,11c-tetrahydrothieno[3',4':5,6]pyrano[3,4-c]chromene-5,6-dione 4c

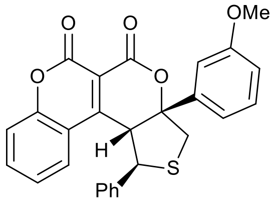

Following the general procedure product **4c** (>20:1 dr in a crude reaction mixture) was isolated (eluent: hexanes/ethyl acetate 4:1) in 74% (33.8 mg) yield as light-yellow solid (mp =169-171 °C). **^{1}H NMR** (700MHz, $CDCl_3$) δ 7.48 – 7.44 (m, 1H), 7.43 – 7.40 (m, 2H), 7.25 – 7.21 (m, 3H), 7.20 – 7.16 (m, 2H), 6.93 – 6.91 (m, 1H), 6.90 – 6.87 (m, 1H), 6.82 – 6.80 (m, 1H), 6.79 – 6.77 (m, 1H), 6.64 – 6.62 (m, 1H), 4.79 (d, *J* = 10.3 Hz, 1H), 4.30 (d, *J* = 10.3, 1H), 3.78 (d, *J* = 12.7 Hz, 1H),3.75 (d, *J* = 12.7 Hz, 1H), 3.72 (s, 3H). **^{13}C NMR** (176 MHz, $CDCl_3$) δ 160.4, 158.5, 158.3, 155.0, 154.8, 139.6, 137.4, 135.3, 130.6, 129.2 (2C), 129.1, 128.4 (2C), 125.8, 124.4, 117.4, 116.6, 116.5, 114.3, 111.2, 110.6, 94.5, 58.1, 55.5, 54.7, 46.1. The er was determined by UPC^2 using a chiral Chiralpack IB column gradient from 100% CO_2 up to 40%; *i*-PrOH, 2.5 mL/min; τ_{major}= 4.61 min, τ_{minor}= 4.78 min, (97:3 er); $[\alpha]_D^{21}$ = -108.5 (c = 1.0, $CHCl_3$). HRMS (ESI): *m/z* calcd for for $C_{27}H_{21}O_5S$ $[M+H]^+$ 457.1110, found 457.1111.

(1*R*,3a*R*,11c*R*)-3a-(2-Fluorophenyl)-1-phenyl-1,3,3a,11c-tetrahydrothieno[3',4':5,6]pyrano[3,4-c]chromene-5,6-dione 4d

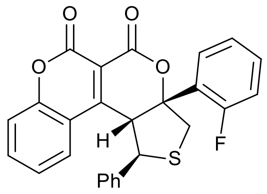

Following the general procedure product **4d** (>20:1 dr in a crude reaction mixture) was isolated (eluent: hexanes/ethyl acetate 4:1) in 88% (40.5 mg) yield as light-yellow solid (mp =182-184 °C). **^{1}H NMR** (700MHz, $CDCl_3$) δ 7.56 – 7.51 (m, 1H), 7.46 – 7.43 (m, 1H), 7.43 – 7.40 (m, 2H), 7.31 – 7.27 (m, 1H), 7.24 – 7.21 (m, 3H), 7.19 – 7.15 (m, 2H), 7.00 – 6.97 (m, 1H), 6.80 – 6.77 (m, 1H), 6.61 – 6.51 (m, 1H), 4.77 (dd, *J* = 10.3, 1.5 Hz, 1H), 4.70 (d, *J* = 10.3 Hz, 1H), 4.07 (d, *J* = 12.5 Hz, 1H), 3.69 (dd, *J* = 12.5, 1.5 Hz, 1H). **^{13}C NMR** (176 MHz, $CDCl_3$) δ 158.9, 158.8 (d, *J* = 245.9 Hz), 158.3, 155.0, 154.9, 137.5, 135.4, 131.3 (d, *J* = 8.9 Hz), 129.2 (2C), 129.0, 128.5 (2C), 128.3 (d, *J* = 2.7 Hz), 126.1, 125.7 (d, *J* = 3.2 Hz), 124.9 (d, *J* = 10.5 Hz), 124.3, 117.3, 117.0 (d, *J* = 23.0 Hz), 116.4, 110.0, 92.9 (d, *J* = 5.1 Hz), 58.8, 53.6 (d, *J* = 9.3 Hz), 44.2 (d, *J* = 2.6 Hz). The er was determined by UPC^2 using a chiral Chiralpack IA column gradient from 100% CO_2 up to 40%; ACN, 2.5 mL/min; τ_{major}= 3.93 min, τ_{minor}= 4.09 min, (97:3 er); $[\alpha]_D^{21}$ = -53.8 (c = 1.0, $CHCl_3$). HRMS (ESI): *m/z* calcd for for $C_{26}H_{18}FO_4S$ $[M+H]^+$ 445.0904, found 445.0913.

(1*R*,3a*R*,11c*R*)-1-Phenyl-3a-(4-(trifluoromethyl)phenyl)-1,3,3a,11c-tetrahydrothieno[3',4':5,6]pyrano[3,4-c]chromene-5,6-dione 4e

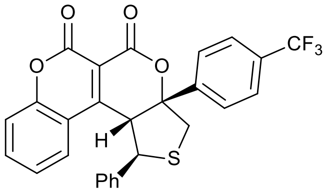

Following the general procedure product **4e** (>20:1 dr in a crude reaction mixture) was isolated (eluent: hexanes/ethyl acetate 4:1) in 76% (37.6 mg) yield as light-yellow solid (mp =146-148 °C). **^{1}H NMR** (700MHz, $CDCl_3$) δ 7.60 – 7.56 (m, 2H), 7.54 – 7.51 (m, 2H), 7.49 – 7.46 (m, 1H), 7.45 – 7.41 (m, 2H), 7.27 – 7.22 (m, 3H), 7.20 – 7.18 (m, 1H), 6.83 – 6.81 (m, 1H), 6.63 – 6.61 (m, 1H), 4.84 (d, *J* = 10.2 Hz, 1H), 4.32 (d, *J* = 10.2 Hz, 1H), 3.79 – 3.75 (m, 2H). **^{13}C NMR** (176 MHz, $CDCl_3$) δ 158.1, 157.9, 154.9, 154.7, 142.0, 136.9, 135.7, 131.6 (q, *J* = 33.0 Hz), 129.3 (2C), 129.2, 128.4 (2C), 126.7 (q, *J* = 3.7 Hz, 2C), 125.7, 125.2 (2C), 124.5, 123.6 (q, *J* = 272.7 Hz), 117.6, 116.3, 110.4, 94.0, 58.2, 54.8, 46.1.The er was determined by UPC^2 using a chiral Chiralpack IA column gradient from 100% CO_2 up to 40%; *i*-PrOH, 2.5 mL/min; τ_{major}= 4.16 min, τ_{minor}= 4.46 min, (97:3 er); $[\alpha]_D^{21}$ =-70.5 (c = 1.0, $CHCl_3$). HRMS (ESI): *m/z* calcd for for $C_{27}H_{18}F_3O_4S$ $[M+H]^+$ 495.0878, found 495.0881.

(1*R*,3a*R*,11c*R*)-3a-(Naphthalen-2-yl)-1-phenyl-1,3,3a,11c-tetrahydrothieno[3',4':5,6]pyrano[3,4-c]chromene-5,6-dione 4f

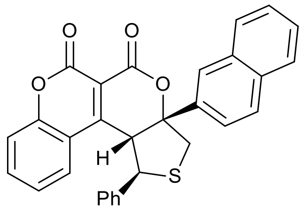

Following the general procedure product **4f** (>20:1 dr in a crude reaction mixture) was isolated (eluent: hexanes/ethyl acetate 4:1) in 84% (32 mg) yield as light-yellow solid (mp =168 - 170 °C). **^{1}H NMR** (700MHz, $CDCl_3$) δ 7.86 – 7.85 (m, 1H), 7.79 – 7.77 (m, 1H), 7.77 – 7.74 (m, 2H), 7.51 – 7.45 (m, 5H), 7.45 – 7.40 (m, 2H), 7.26 – 7.24 (m, 2H), 7.17 – 7.13 (m, 1H), 6.83 – 6.81 (m, 1H), 6.68 – 6.66 (m, 1H), 4.87 (d, *J* = 10.3 Hz, 1H), 4.43 (d, *J* = 10.3 Hz, 1H), 3.90 (d, *J* = 12.8 Hz, 1H), 3.84 (d, *J* = 12.8 Hz, 1H). **^{13}C NMR** (176 MHz, $CDCl_3$) δ 158.4, 158.3, 154.9, 154.8, 137.4, 135.3, 135.2, 133.2, 133.1, 129.8, 129.2 (2C), 129.1, 128.6, 128.5 (2C), 127.7, 127.4, 127.3, 125.7, 124.6, 124.3, 121.5, 117.4, 116.5, 110.7, 94.8, 58.4, 55.0, 46.2. The er was determined by UPC^2 using a chiral Chiralpack IB column gradient from 100% CO_2 up to 40%; *i*-PrOH, 2.5 mL/min; τ_{major}= 5.59 min, τ_{minor}= 6.08 min, (97:3 er); $[\alpha]_D^{21}$ = -121.5 (c = 1.0, $CHCl_3$). HRMS (ESI): *m/z* calcd for for $C_{30}H_{21}O_4S$ $[M+H]^+$ 477.1161, found 477.1166.

(1*R*,3a*R*,11c*R*)-1-(4-Methoxyphenyl)-3a-phenyl-1,3,3a,11c-tetrahydrothieno[3',4':5,6]pyrano[3,4-c]chromene-5,6-dione 4g

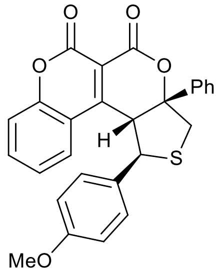

Following the general procedure product **4g** (>20:1 dr in a crude reaction mixture) was isolated (eluent: hexanes/ethyl acetate 4:1) in 57% (26 mg) yield as light-yellow solid (mp =163-165 °C). **^{1}H NMR** (700MHz, $CDCl_3$) δ 7.49 –7.46 (m, 1H), 7.38 – 7.35 (m, 2H), 7.35 – 7.32 (m, 2H), 7.32 – 7.28 (m, 2H), 7.28 – 7.26 (m, 1H), 7.19 -7.17 (m, 1H), 6.89 – 6.86 (m, 1H), 6.78 – 6.74 (m, 2H), 6.73 – 6.71 (m, 1H), 4.80 (d, *J* = 10.3 Hz, 1H), 4.29 (d, *J* = 10.3 Hz, 1H), 3.76 (d, *J* = 2.2 Hz, 2H), 3.75 (s, 3H). **^{13}C NMR** (176 MHz, $CDCl_3$) δ 160.3, 158.6, 158.3, 154.9, 154.9, 138.2, 135.3, 129.5 (4C), 129.2, 129.0, 126.0, 124.7 (2C), 124.4, 117.4, 116.6, 114.6 (2C), 110.7, 94.7, 57.8, 55.6, 54.8, 46.1. The er was determined by UPC^2 using a chiral Chiralpack IA column gradient from 100% CO_2 up to 40%; *i*-PrOH, 2.5 mL/min; τ_{major}= 5.12 min, τ_{minor}= 5.28 min, (99:1 er); $[\alpha]_D^{21}$ = -94,7 (c = 1.0, $CHCl_3$). HRMS (ESI): *m/z* calcd for for $C_{27}H_{21}O_5S$ $[M+H]^+$ 457.1110, found 457.1108.

(1*R*,3a*R*,11c*R*)-3a-Phenyl-1-(*m*-tolyl)-1,3,3a,11c-tetrahydrothieno[3',4':5,6]pyrano[3,4-c]chromene-5,6-dione 4h

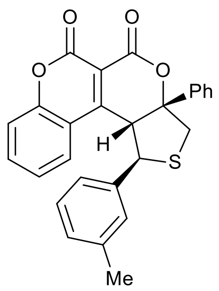

Following the general procedure product **4h** (>20:1 dr in a crude reaction mixture) was isolated (eluent: hexanes/ethyl acetate 4:1) in 61% (26.8 mg) yield as light-yellow solid (mp =172-174 °C). **^{1}H NMR** (700MHz, $CDCl_3$) δ 7.48-7.46 (m, 1H), 7.38 – 7.36 (m, 2H), 7.32 – 7.28 (m, 2H), 7.28 – 7.26 (m, 1H), 7.25 – 7.23 (m, 1H), 7.19 – 7.17 (m, 2H), 7.14-7.11 (m, 1H), 7.06 – 7.04 (m, 1H), 6.84-6.81 (m, 1H), 6.65-6.63 (m, 1H), 4.77 (d, *J* = 10.3 Hz, 1H), 4.30 (d, *J* = 10.3 Hz, 1H), 3.81 – 3.74 (m, 2H), 2.22 (s, 3H). **^{13}C NMR** (176 MHz, $CDCl_3$) δ 158.5, 158.3, 154.9, 154.9, 139.0, 138.1, 137.3, 135.3, 129.7, 129.5 (2C), 129.2, 129.1, 129.1, 126.0, 125.4 (2C), 124.7, 124.2, 117.4, 116.6, 110.7, 94.7, 58.2, 54.8, 46.2, 21.3. The er was determined by UPC^2 using a chiral Chiralpack IA column gradient from 100% CO_2 up to 40%; *i*-PrOH, 2.5 mL/min; τ_{major}= 4.62 min, τ_{minor}= 4.74 min, (99:1 er); $[\alpha]_D^{21}$ = -119.7 (c = 1.0, $CHCl_3$). HRMS (ESI): *m/z* calcd for $C_{27}H_{21}O_4S$ $[M+H]^+$ 441.1161, found 441.1162.

7. Enantioselective synthesis of 4a – one-pot procedure

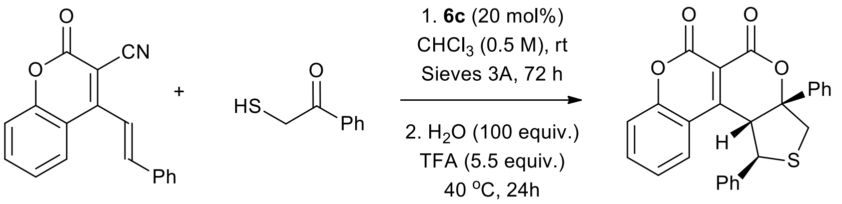

1a **2a** **4a**

In an ordinary 4 mL glass vial, equipped with a magnetic stirring bar, screw cap, and molecular sieves (3Å), corresponding mercaptocarbonyl compound **2a** (1.2 equiv., 0.12 mmol, 18.3 mg) was dissolved in $CHCl_3$ (0.2 mL). Next, 3-cyano-4-styrylcoumarine **1a** (1 equiv. 0.1 mmol, 27.3 mg) and catalyst **6c** (0.2 equiv., 0.02 mmol, 12.6 mg) were added. The reaction mixture was stirred for 72 h at ambient temperature. Subsequently, H_2O (0.2 mL, 100 equiv.) and CF_3CO_2H (40 μL, 5.4 equiv.) were added directly to the reaction mixture and the resulting mixture was stirred for 24 h at 40 ^oC in a heating block. Reaction mixture was then transferred to a separatory funnel, diluted with water (5 mL), and extracted with $CHCl_3$ (3 x 10 mL). Organic layer was dried with anhydrous magnesium sulfate, filtered, evaporated with small amount of silica gel to dryness and subjected to flash chromatography on silica gel (eluent: hexanes/ethyl acetate 4:1) to obtain pure product **4a** (>20:1 dr in a crude reaction mixture) was isolated in 75% (31.9 mg) yield as white solid (mp =158-160 ^oC). Spectroscopic data were in accordance to the reported above. The er was determined by UPC^2 using a chiral Chiralpack IB column gradient from 100% CO_2 up to 40%; *i*-PrOH, 2.5 mL/min; τ_{major}= 4.57 min, τ_{minor}= 4.88 min, (98:2 er); $[\alpha]_D^{21}$ = -83.4 (c = 1.0, $CHCl_3$). HRMS (ESI): *m/z* calcd for $C_{26}H_{19}O_4S$ $[M+H]^+$ 427.1004, found 427.1012.

8. Transformation of 3a– synthesis of (*Z*)-*tert*-butyl-((1*R*,3a*R*,11c*R*)-6-oxo-1,3a-diphenyl-1,3,3a,11c-tetrahydrothieno[3',4':5,6]pyrano[3,4-c]chromen-5(6*H*)-ylidene)carbamate 7

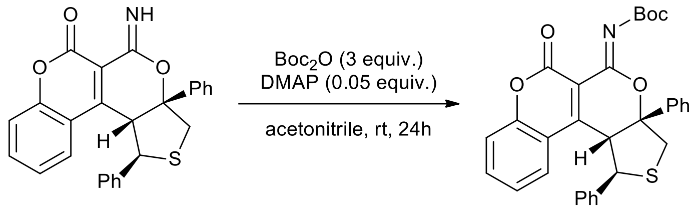

3a **7**

In an ordinary 4 mL glass vial, equipped with a magnetic stirring bar and a screw cap, Boc_2O (3 equiv., 0.3 mmol, 66 mg), DMAP (0.05 equiv., 0.015 mmol, 1.2 mg) were dissolved in 0.4 ml acetonitrile and compound **3a** (1 equiv., 0.1 mmol, 22,6 mg) was added. Reaction was stirred at 25 °C for 24 h, evaporated and directly subjected to flash chromatography on silica gel (eluent: hexanes/ethyl acetate 6:1) to obtain pure product **7** with 53% yield (28 mg) as yellow oil. **^{1}H NMR** (700MHz, $CDCl_3$) δ 7.47 – 7.42 (m, 4H), 7.40 (ddd, *J* = 8.3, 7.3, 1.5 Hz, 1H), 7.32 – 7.29 (m, 2H), 7.27-7.25 (m, 1H), 7.25 – 7.21 (m, 3H), 7.16 (dd, *J* = 8.3, 1.1 Hz, 1H), 6.77 (ddd, *J* = 8.3, 7.3, 1.2 Hz, 1H), 6.59 (dd, *J* = 8.2, 1.5 Hz, 1H), 4.85 (d, *J* = 10.2 Hz, 1H), 4.32 (dd, *J* = 10.3, 0.6 Hz, 1H), 3.76 (d, *J* = 12.7 Hz, 1H), 3.61 (d, *J* = 12.7 Hz, 1H), 1.66 (s, 9H). **^{13}C NMR** (176 MHz, $CDCl_3$) δ 159.1, 154.7, 154.4, 154.2, 137.9, 137.6, 134.4, 129.6 (2C), 129.1 (2C), 128.9, 128.5 (2C), 128.5, 125.1, 124.7, 124.5 (2C), 124.1, 117.2, 116.8, 111.6, 93.4, 82.4, 57.3, 54.9, 45.9, 28.4 (3C). $[\alpha]_D^{21}$ = -35,6 (c = 1.0, $CHCl_3$). HRMS (ESI): *m/z* calcd for $C_{31}H_{28}NO_5S$ $[M+H]^+$ 526.1688, found 526.1696.

9. Crystal and X-ray data for (1*R*,3a*R*,11c*R*)-3a-(2-Methoxyphenyl)-1-phenyl-1,3,3a,11c-tetrahydrothieno[3',4':5,6]pyrano[3,4-c]chromene-5,6-dione 4b

Single crystal of **4b** was obtained from dichlormethane at 5 °C. The crystal structure of the compound **4b**, ($C_{27}H_{20}O_5S \cdot 0.25\ CH_2Cl_2$), was established by single-crystal X-ray diffraction at 100 K. The compound crystallizes in the non-centrosymmetric tetragonal space group $P4_2$ (Z = 2) and the crystal structure consists of two crystallographically independent formula units of the main molecule and a half of the dichloromethane solvent molecule in the unit cell (Figure 1).

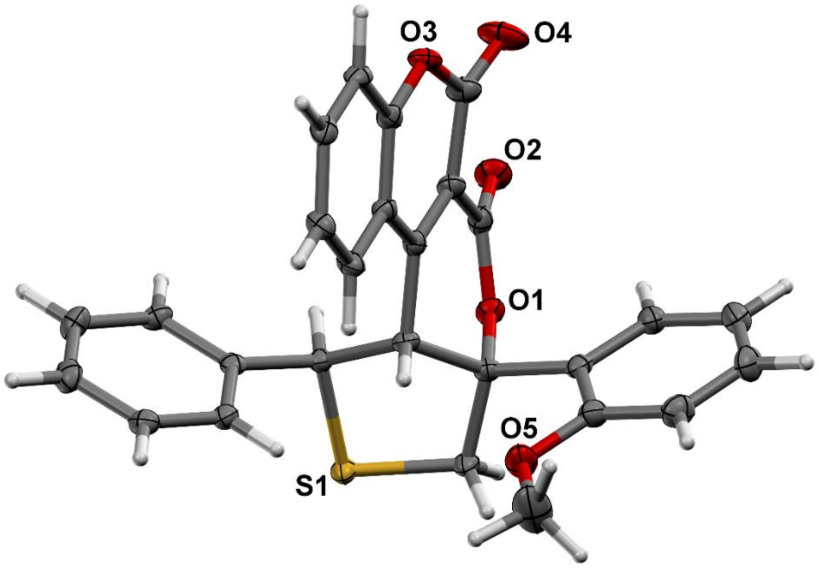

Figure 1. The molecular structure of the main molecule of the compound JD-5B at 100 K, with the heteroatom labeling scheme, showing 50% probability displacement ellipsoids. Hydrogen atoms are drawn with an arbitrary radius.

Single crystal X-ray diffraction data were collected at 100 K by the ω-scan technique using a RIGAKU XtaLAB Synergy, Dualflex, Pilatus 300K diffractometer[3] with PhotonJet micro-focus X-ray Source Cu-Kα (λ = 1.54184 Å). Data collection, cell refinement, data reduction and absorption correction were performed using CrysAlis PRO software.[3] The crystal structure was solved by using direct methods with the SHELXT 2018/2 program.[4] Atomic scattering factors were taken from the International Tables for X-ray Crystallography. Positional parameters of non-H-atoms were refined by a full-matrix least-squares method on F^2 with anisotropic thermal parameters by using the SHELXL 2018/3 program.[5] All hydrogen atoms were found from the difference Fourier maps and for further calculations they were positioned geometrically in calculated positions (C–H = 0.95–1.00 Å) and constrained to ride on their

parent atoms with isotropic displacement parameters set to 1.2-1.5 times the U_{eq} of the parent atom.

4b: Formula $C_{109}H_{82}Cl_2O_{20}S_4$, tetragonal, space group $P4_2$, Z = 2, unit cell constants a = 22.7866(5), c = 8.7584(4) Å, V = 4547.6(3) $Å^3$. The integration of the data yielded a total of 165438 reflections with θ angles in the range of 3.88 to 66.59°, of which 7821 were unique (R_{int} = 3.44%) which were used in all calculations. The final anisotropic full-matrix least-squares refinement on F^2 with 611 parameters. The final R_1 was 0.0330 (for I > 2σ(I)) and wR_2 was 0.0969 (all data). The largest peak in the final difference electron density synthesis was 0.95 $eÅ^{-3}$ and the largest hole was -0.71 $eÅ^{-3}$. The goodness-of-fit was 1.049. The absolute configuration was unambiguously established from anomalous scattering, by calculating the *x* Flack parameter[6] of -0.001(8) using 3406 quotients.

CCDC 2168554 contains the supplementary crystallographic data for this paper. These data can be obtained free of charge from The Cambridge Crystallographic Data Centre *via* www.ccdc.cam.ac.uk/structures.

[3] Rigaku OD. CrysAlis PRO. Rigaku Oxford Diffraction Ltd, Yarnton, Oxfordshire, England, **2019**.

[4] Sheldrick, G.M. *Acta Cryst.* **2015**, *A71*, 3-8.

[5] Sheldrick, G.M. *Acta Cryst.* **2015**, *C71*, 3-8.

[6] Parsons, S.; Flack, H.D.; Wagner, T. *Acta Cryst.* **2013**, *B69*, 249-259.

10. NMR data

2-Oxo-4-styryl-2*H*-chromene-3-carbonitrile 1a
^{1}H NMR

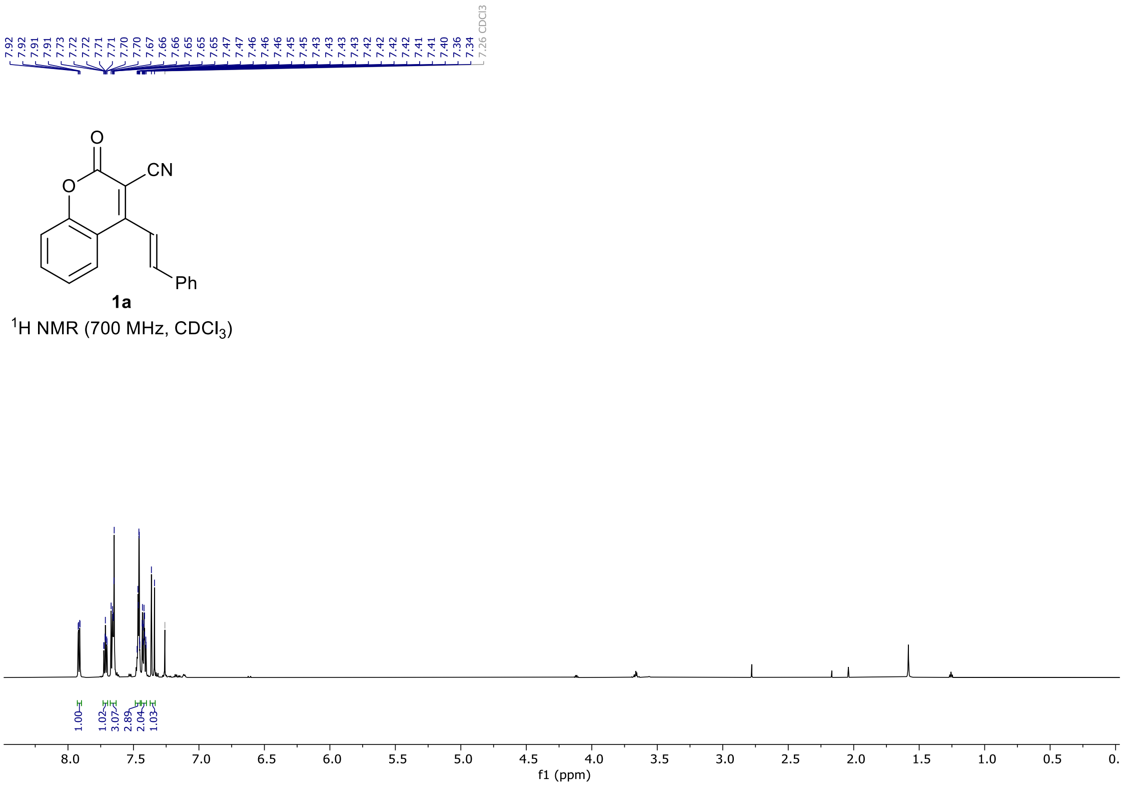

(*E*)-4-(4-Methoxystyryl)-2-oxo-2*H*-chromene-3-carbonitrile 1b
^{1}H NMR

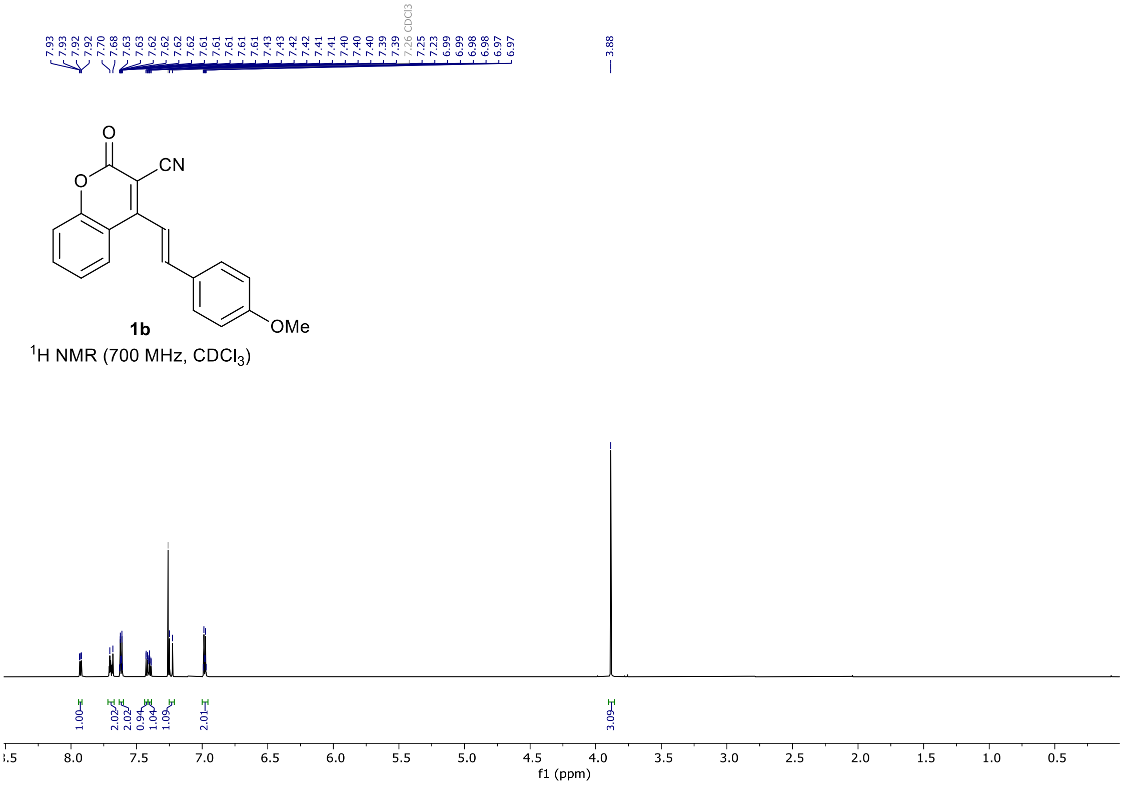

(E)-4-(3-Methylstyryl)-2-oxo-2H-chromene-3-carbonitrile 1c
^{1}H NMR

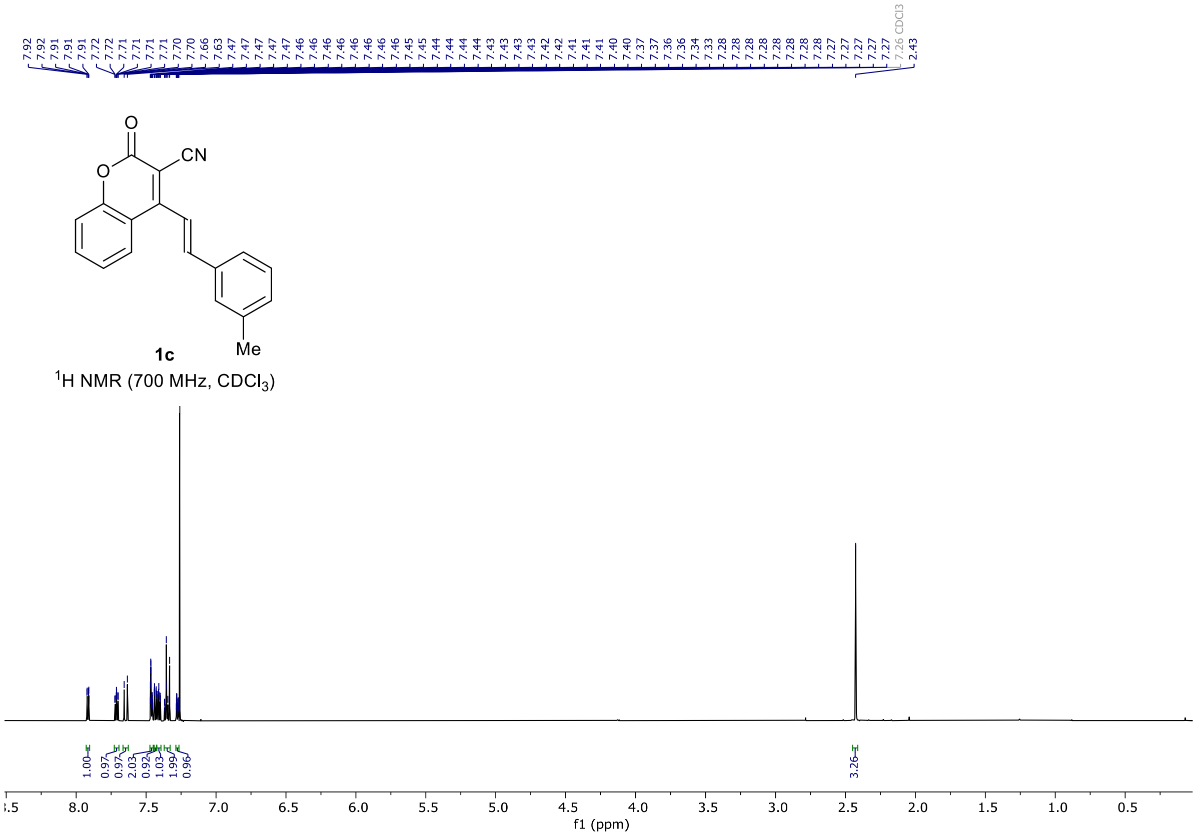

(E)-4-(4-Methylstyryl)-2-oxo-2H-chromene-3-carbonitrile 1d
^{1}H NMR

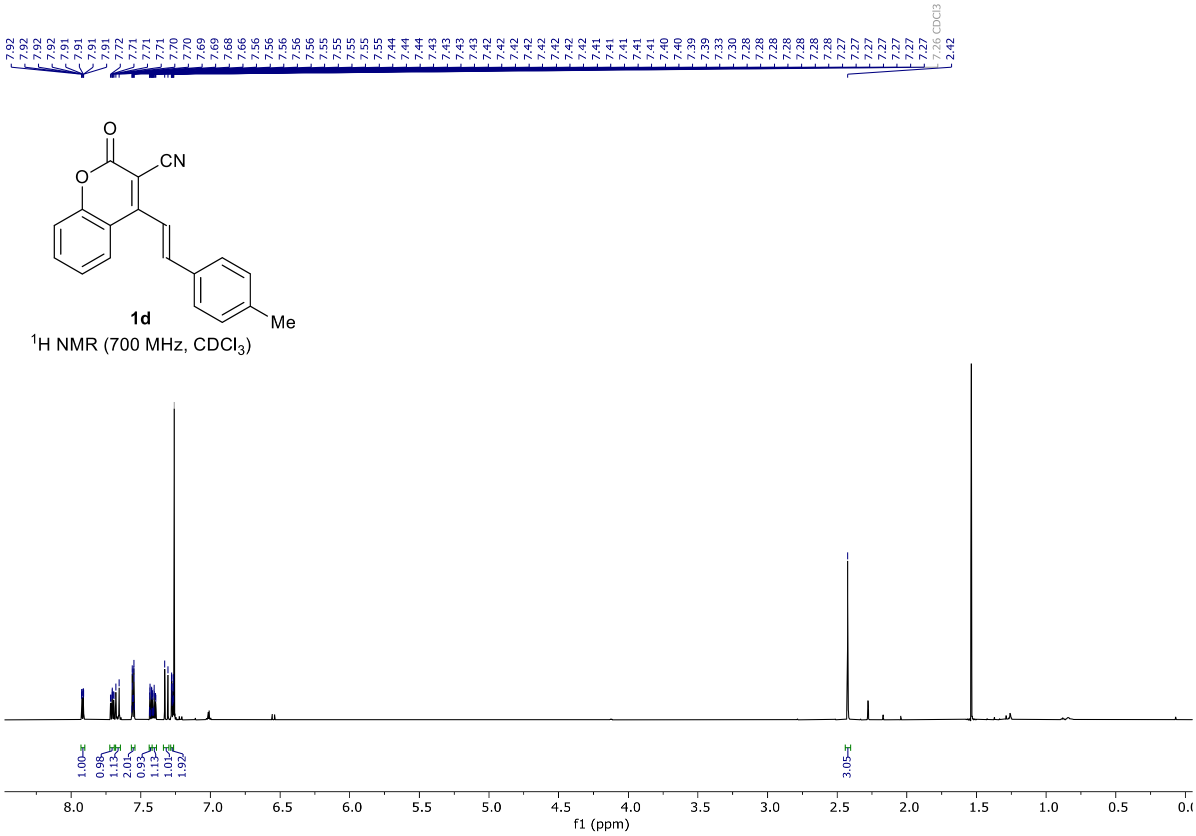

(*E*)-4-(3-Chlorostyryl)-2-oxo-2*H*-chromene-3-carbonitrile 1e
^{1}H NMR

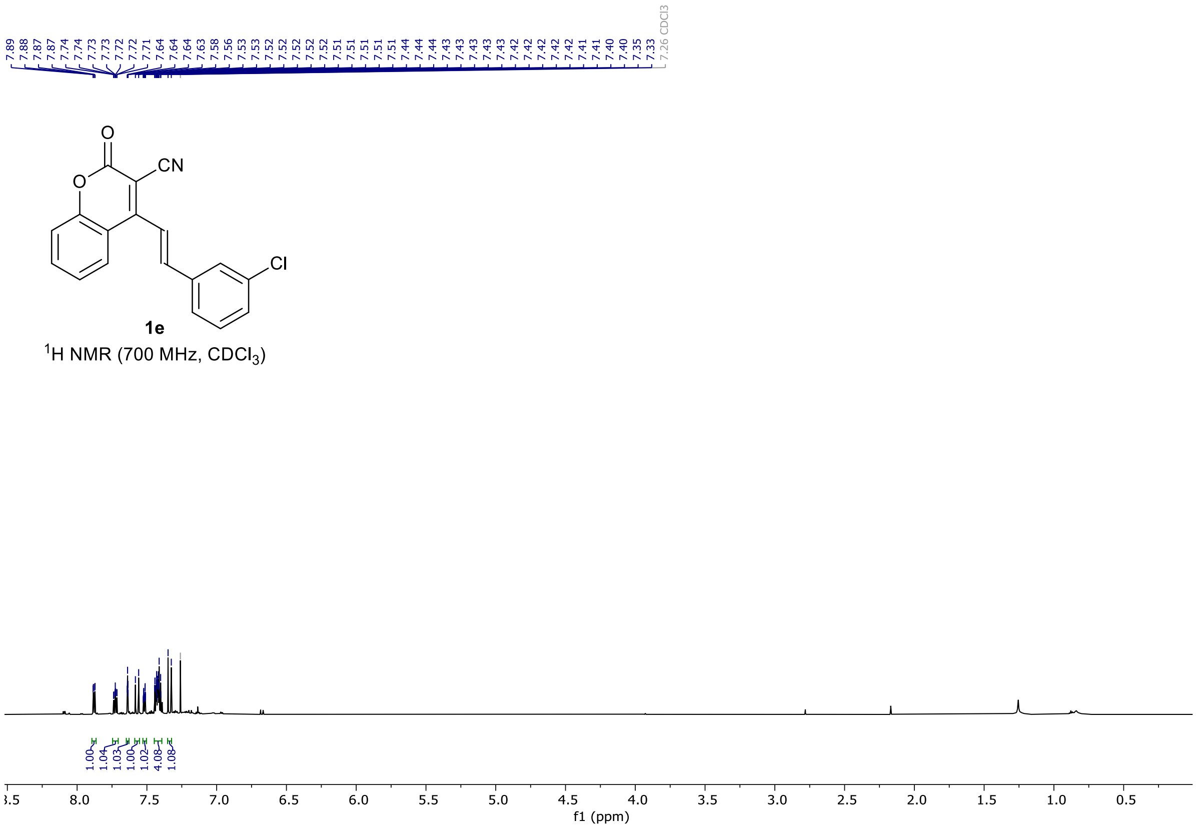

(*E*)-4-(4-Chlorostyryl)-2-oxo-2*H*-chromene-3-carbonitrile 1f
^{1}H NMR

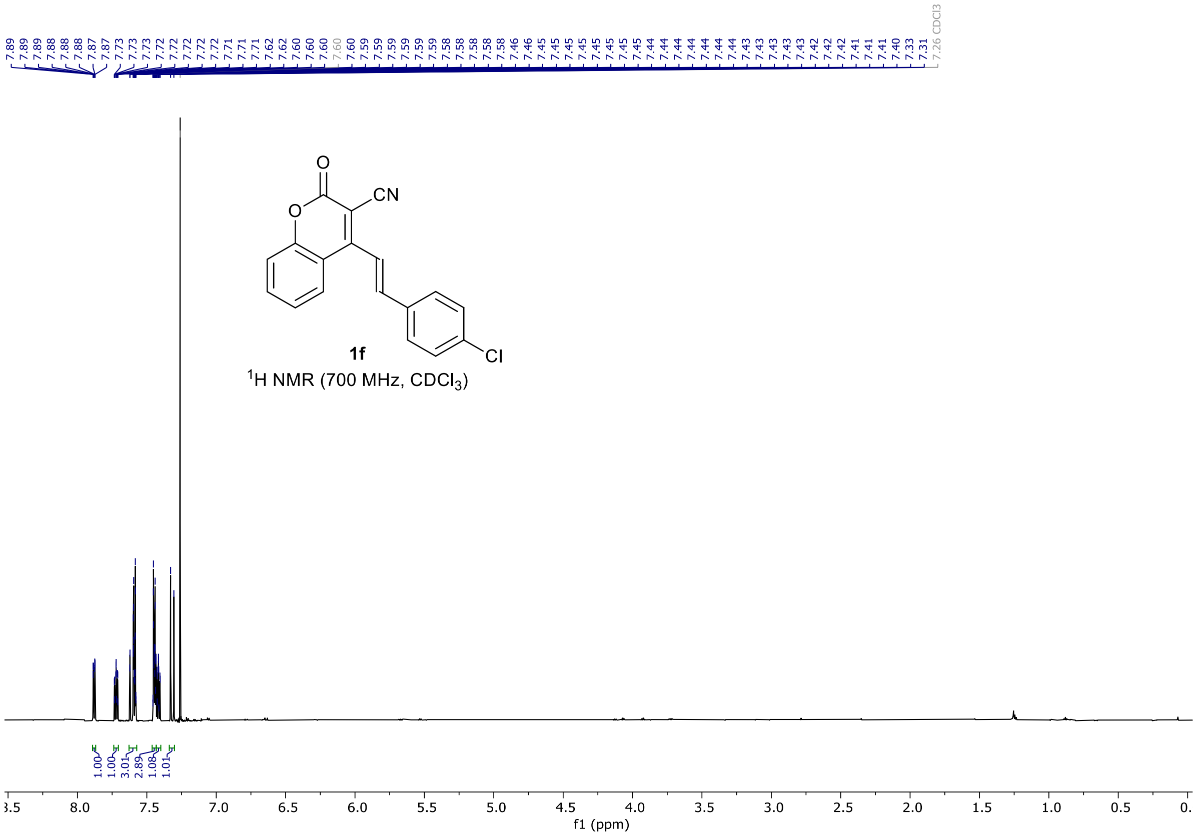

(*E*)-4-(4-Nitrostyryl)-2-oxo-2*H*-chromene-3-carbonitrile 1g
^{1}H NMR

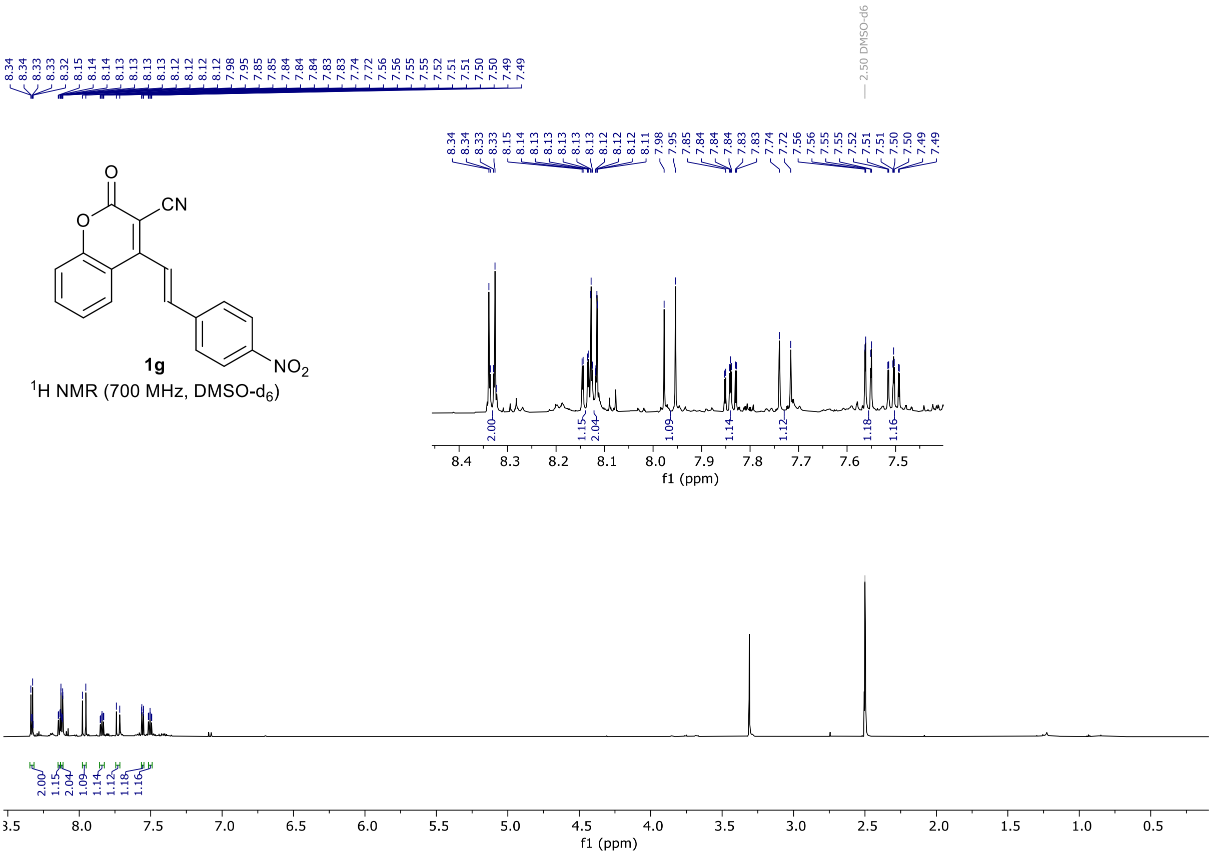

(*E*)-2-Oxo-4-(4-(trifluoromethyl)styryl)-2*H*-chromene-3-carbonitrile 1h
^{1}H NMR

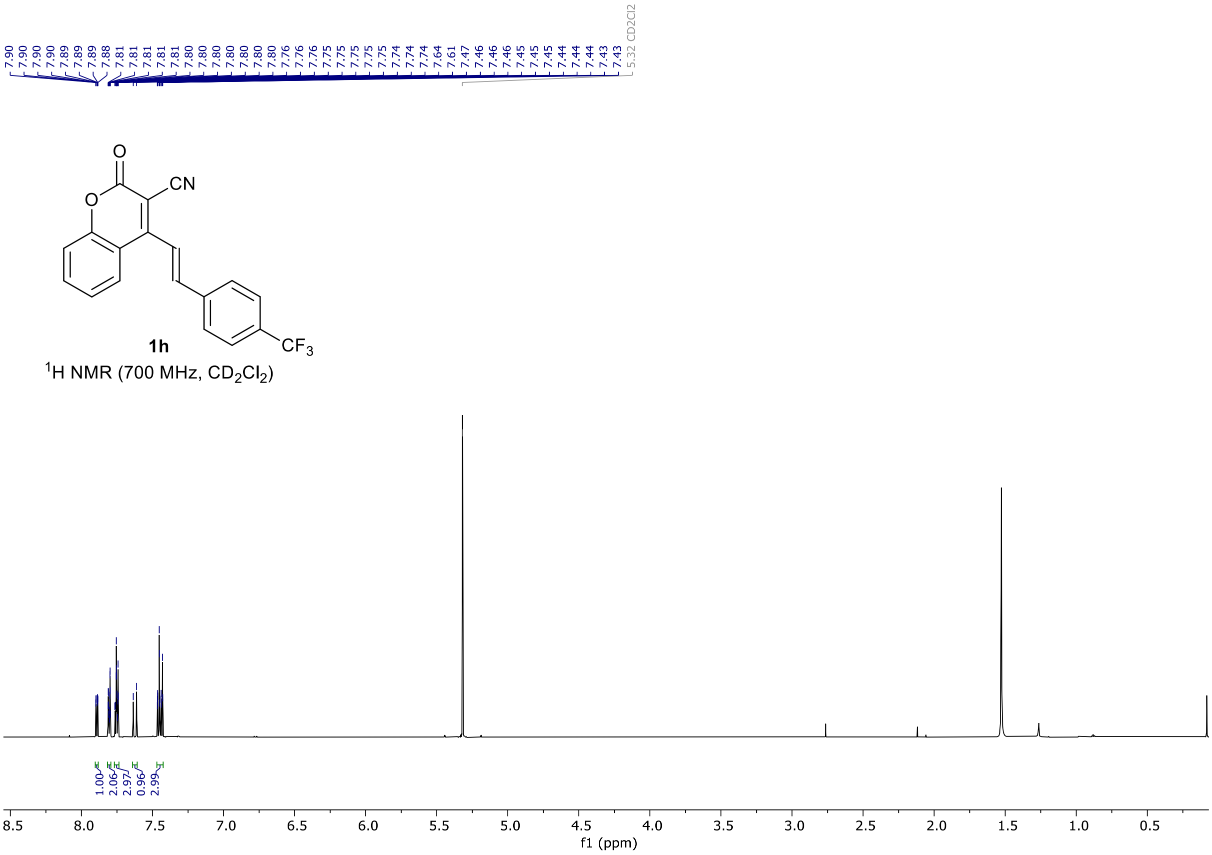

(*E*)-7-Methoxy-2-oxo-4-styryl-2*H*-chromene-3-carbonitrile 1i
^{1}H NMR

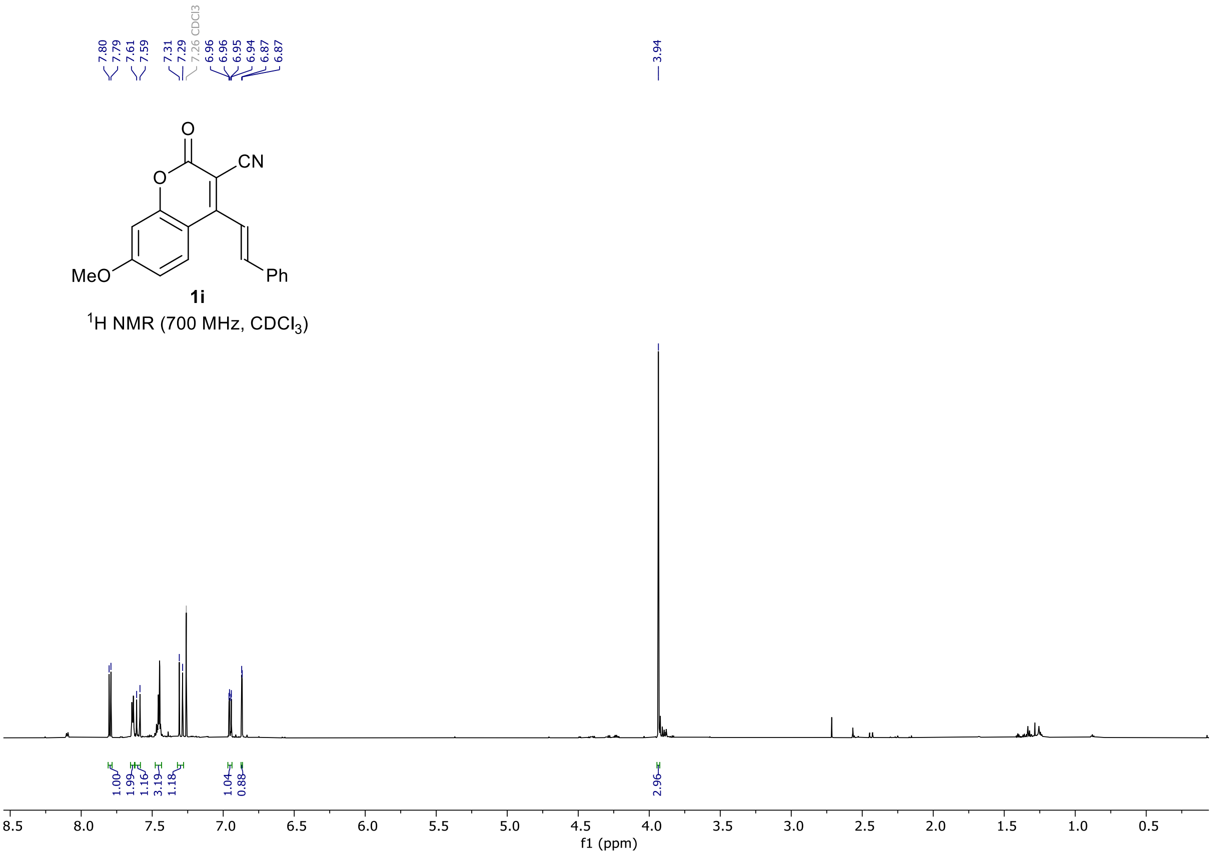

(*E*)-6-Bromo-2-oxo-4-styryl-2*H*-chromene-3-carbonitrile 1j
^{1}H NMR

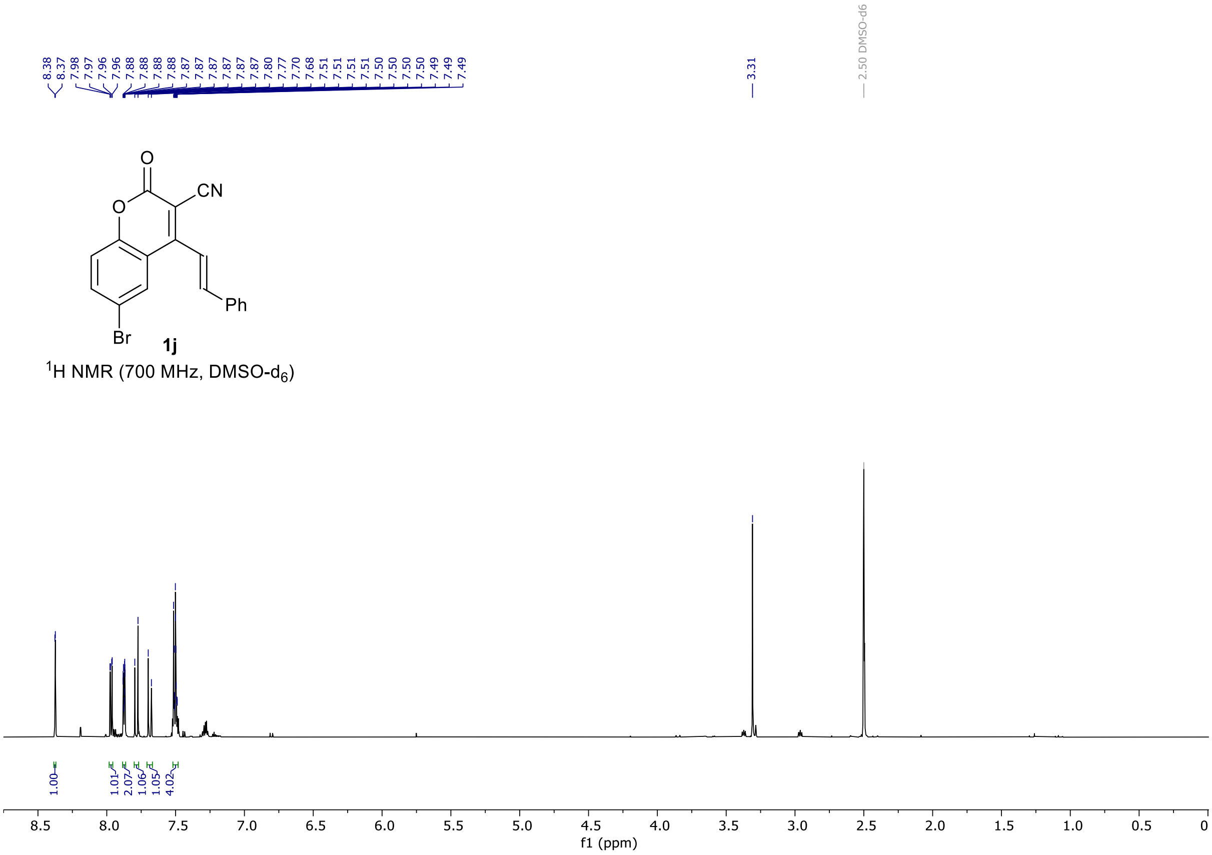

2-Mercapto-1-phenylethanone 2a

^{1}H NMR

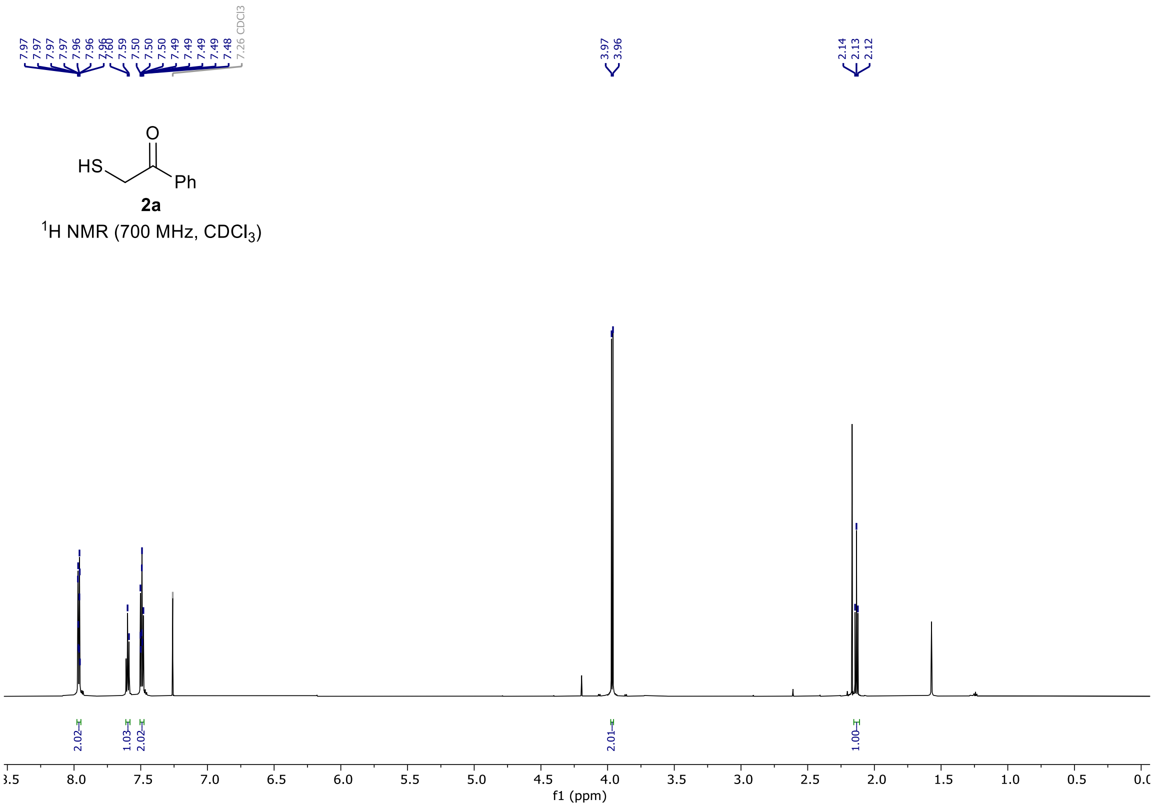

2-Mercapto-1-(2-methoxyphenyl)ethenone 2b

^{1}H NMR

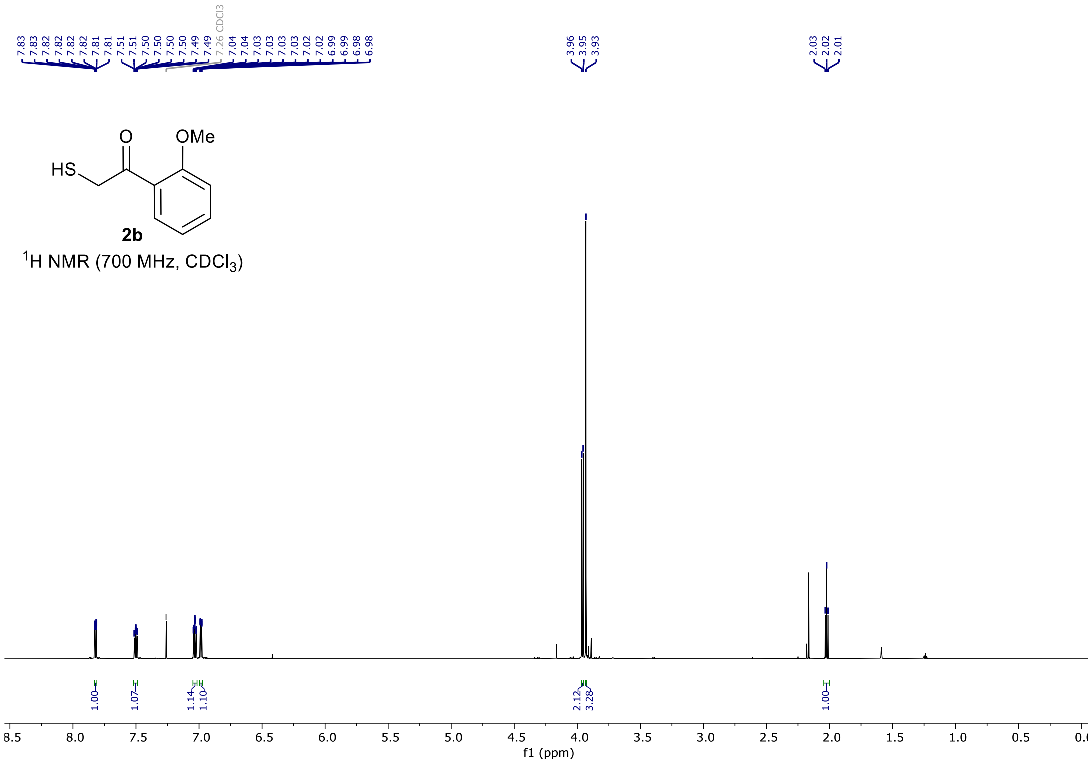

2-Mercapto-1-(3-methoxyphenyl)ethenone 2c

^{1}H NMR

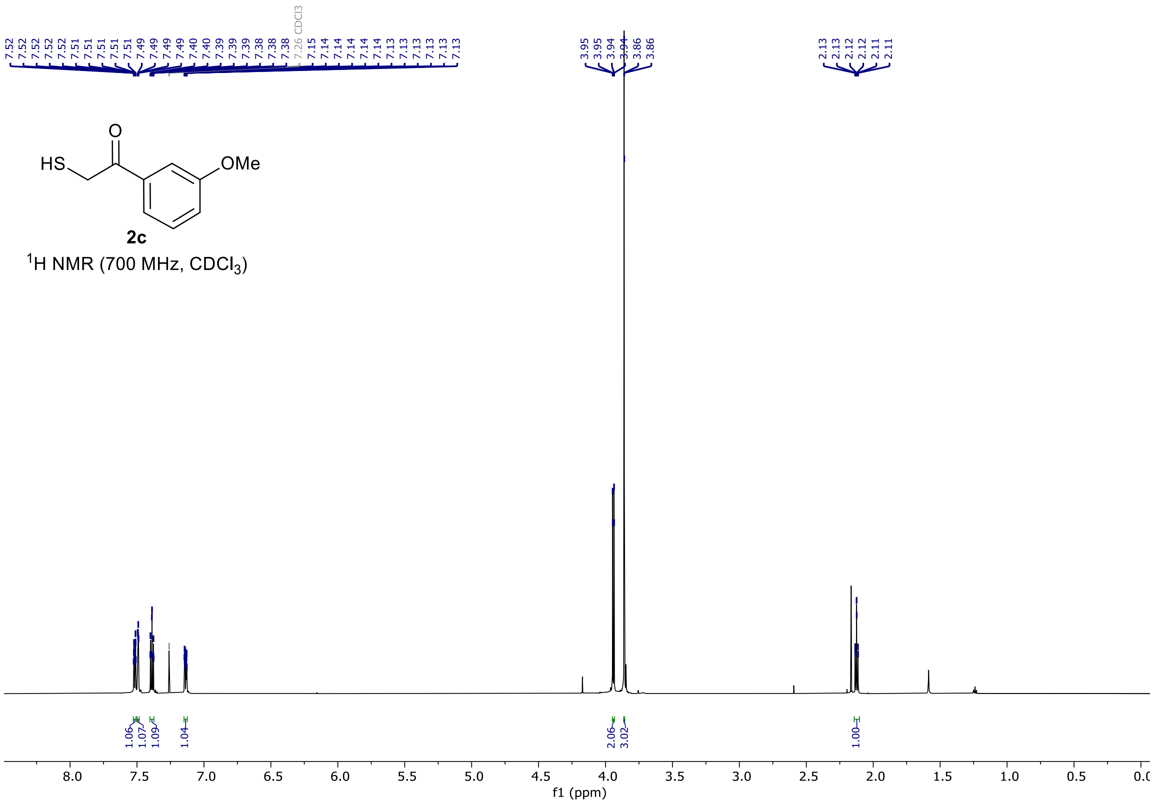

2-Mercapto-1-(p-tolyl)ethenone 2d

^{1}H NMR

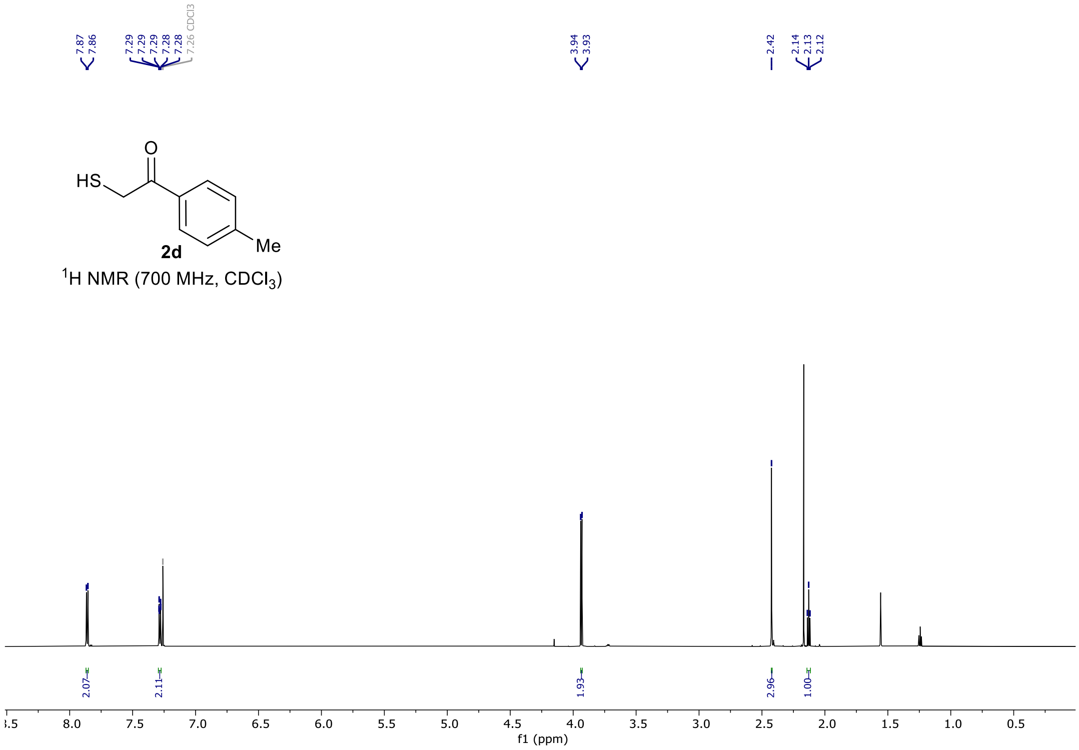

1-(2-Fluorophenyl)-2-mercaptoethanone 2e

^{1}H NMR

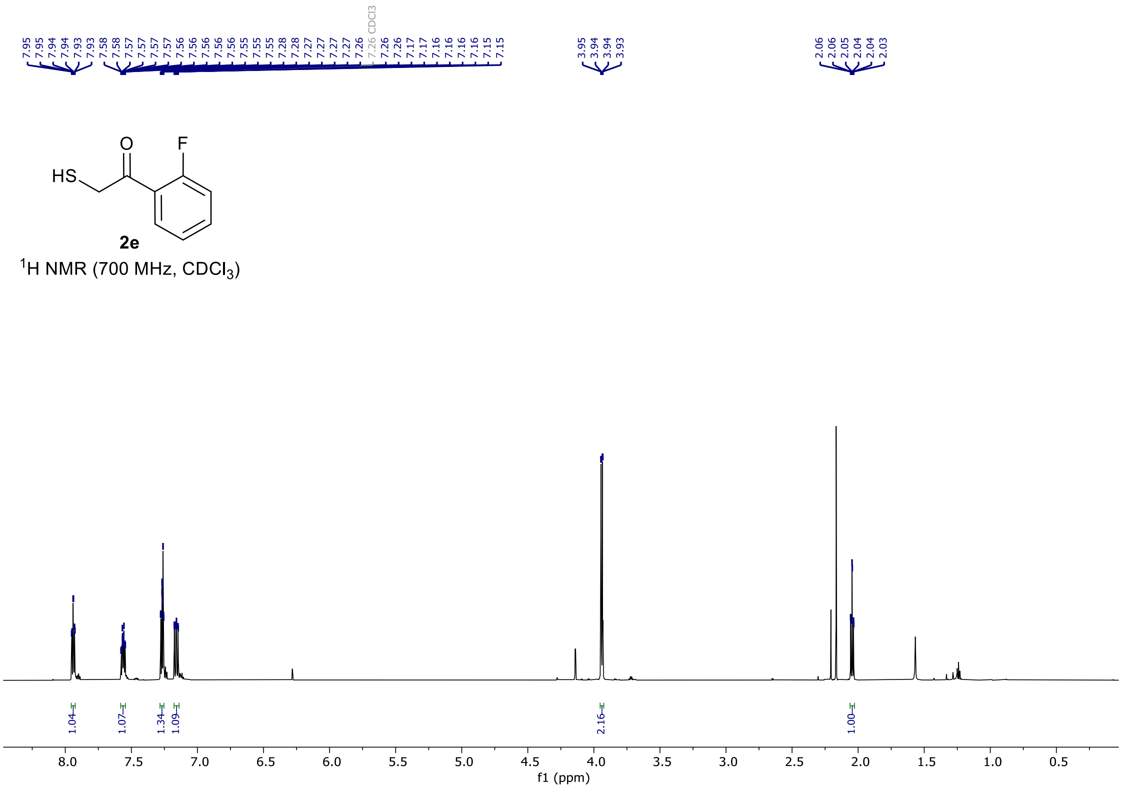

1-(3-Fluorophenyl)-2-mercaptoethanone 2f

^{1}H NMR

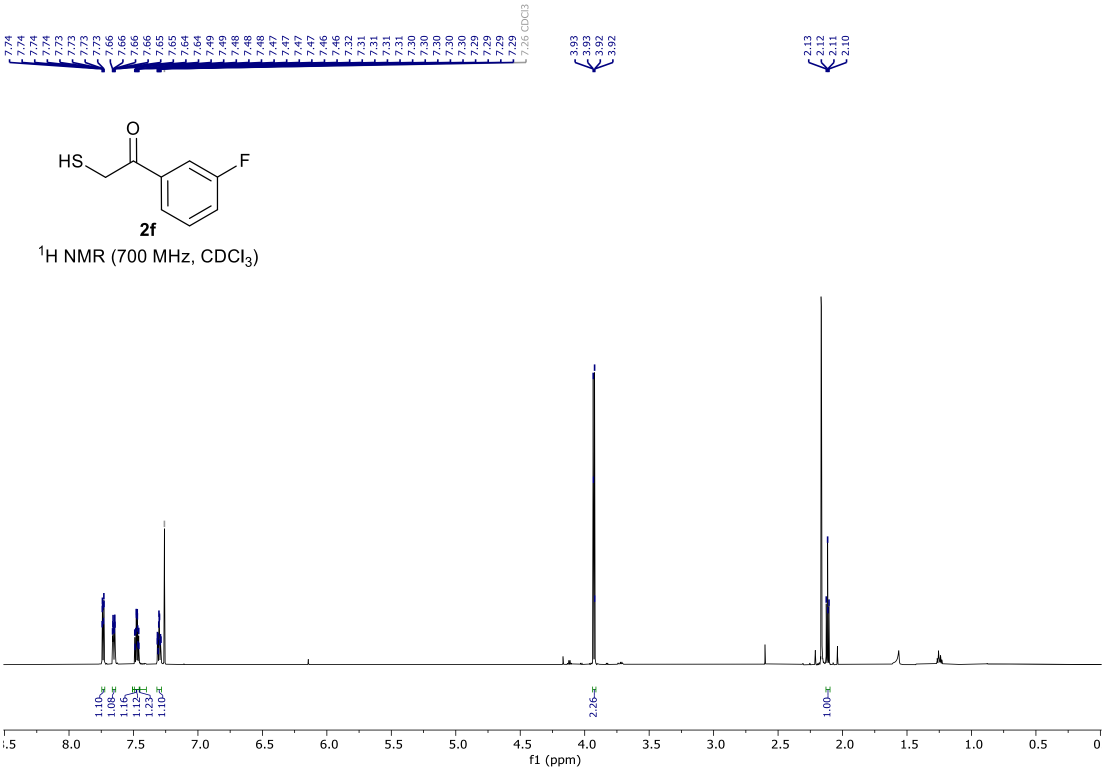

2-Mercapto-1-(4-(trifluoromethyl)phenyl)ethenone 2g

^{1}H NMR

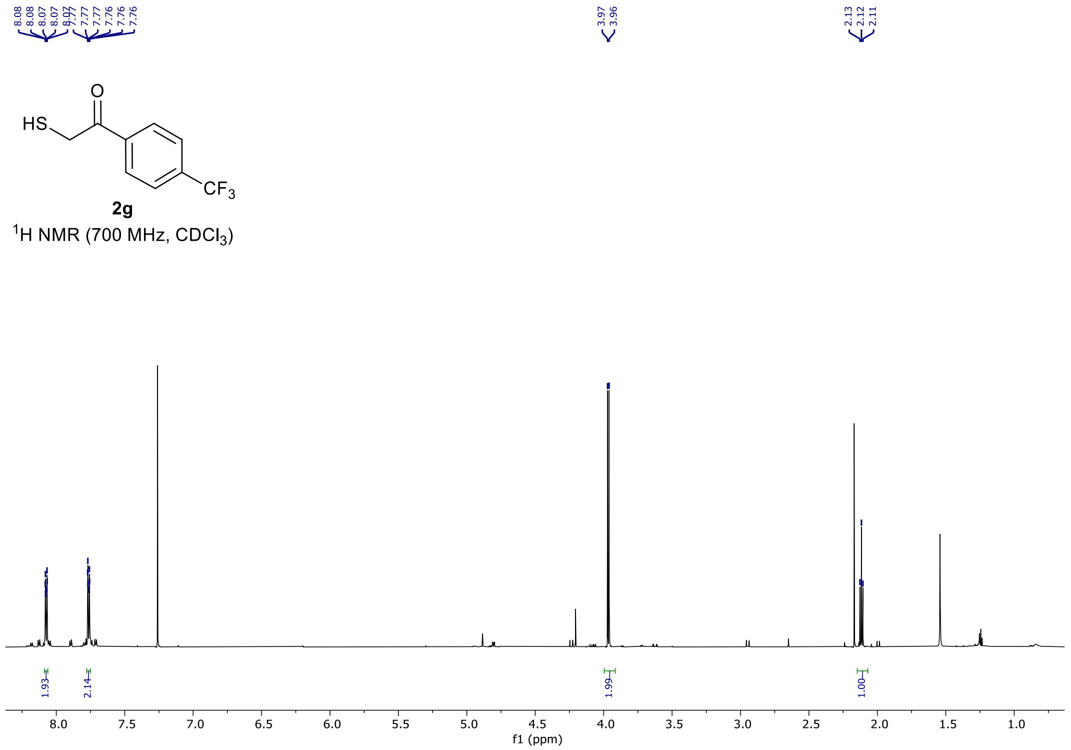

2-Mercapto-1-(naphthalen-2-yl)ethenone 2h

^{1}H NMR

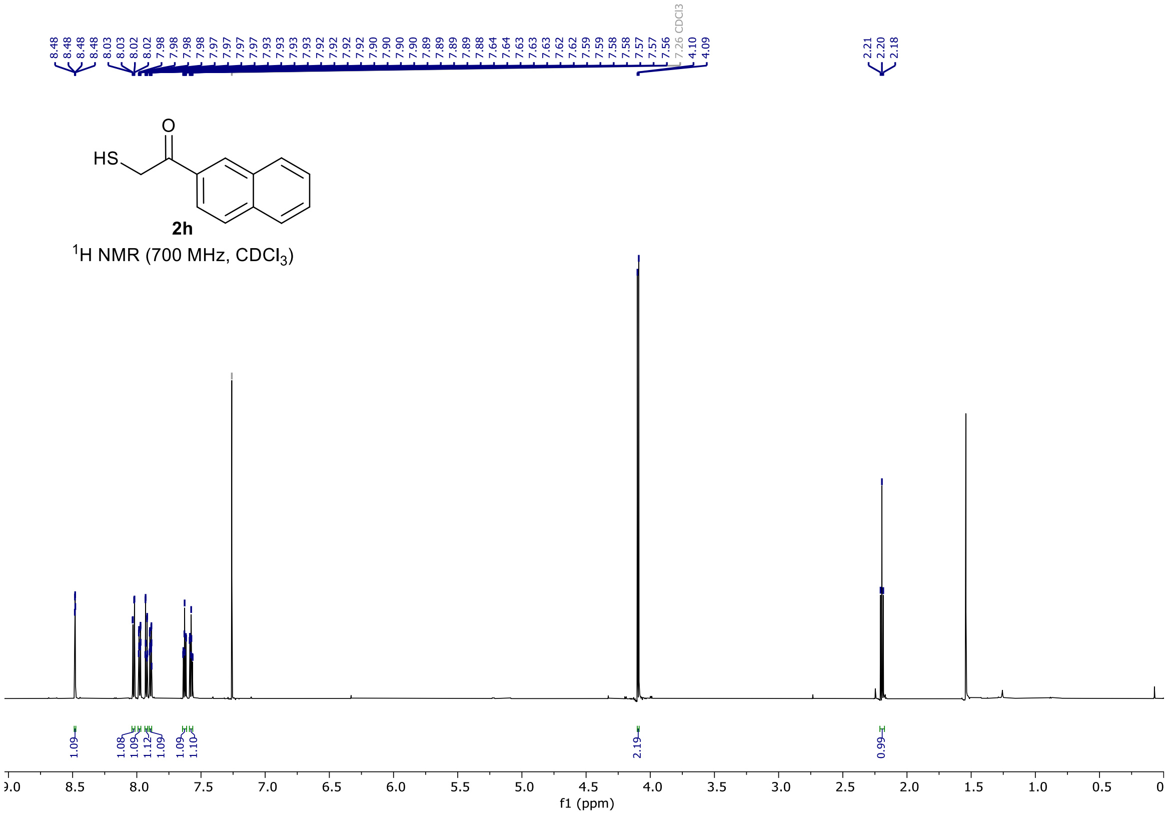

(1*R*,3a*R*,11c*R*)-5-Imino-1,3a-diphenyl-1,3,3a,11c-tetrahydrothieno[3',4':5,6]pyrano[3,4-c]chromen-6(5*H*)-one 3a

^{1}H NMR

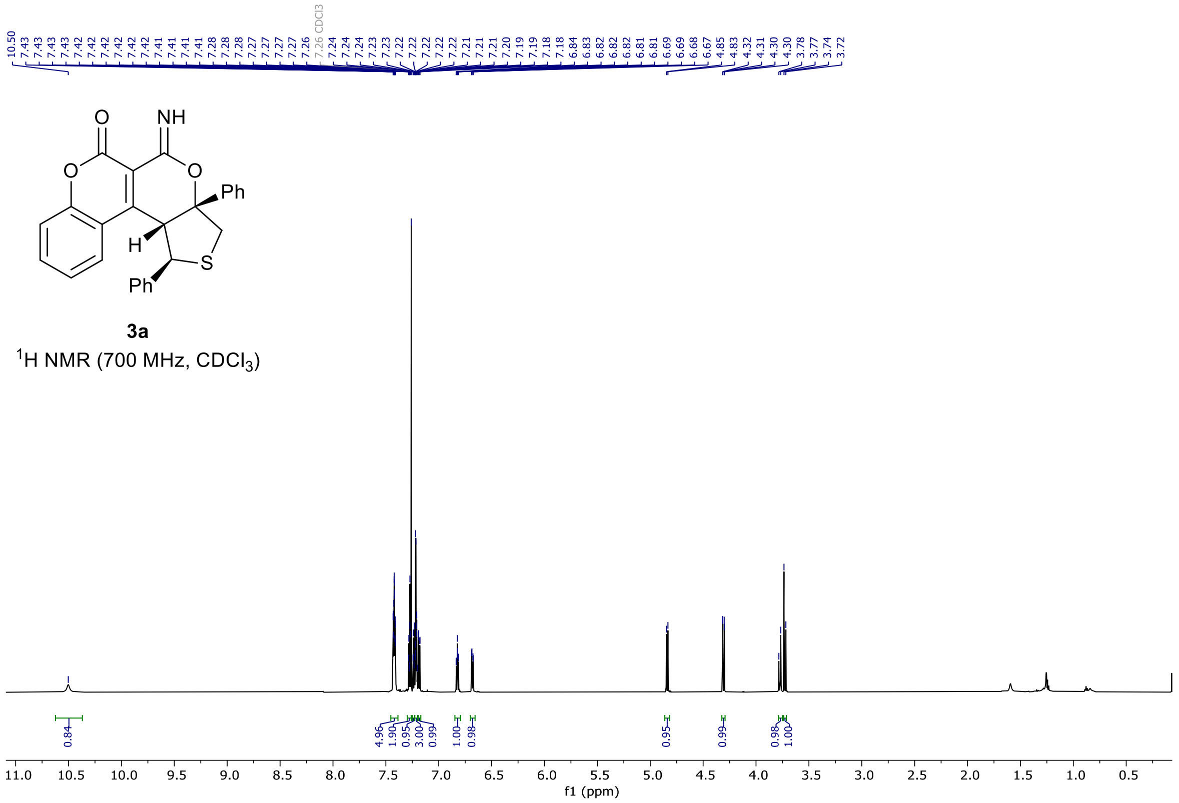

^{13}C NMR

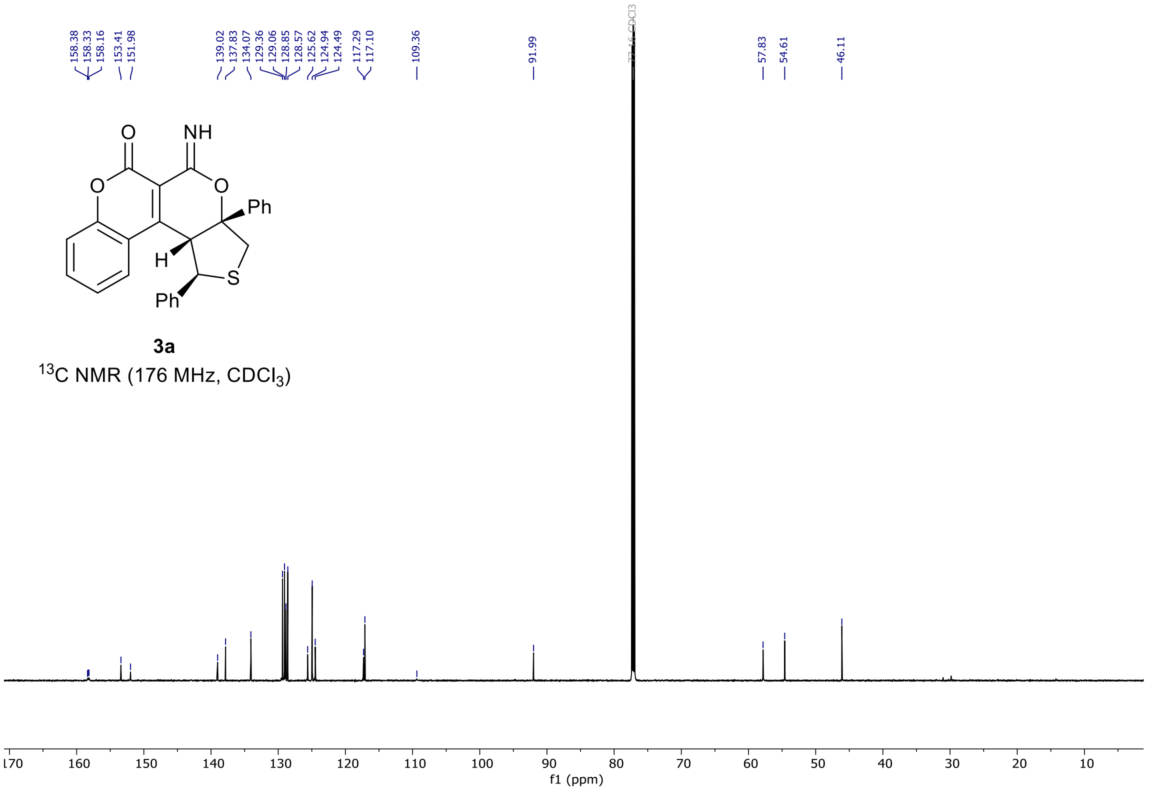

(1*R*,3a*R*,11c*R*)-5-Imino-3a-(2-methoxyphenyl)-1-phenyl-1,3,3a,11c-tetrahydrothieno[3',4':5,6]pyrano[3,4-c]chromen-6(5*H*)-one 3b

^{1}H NMR

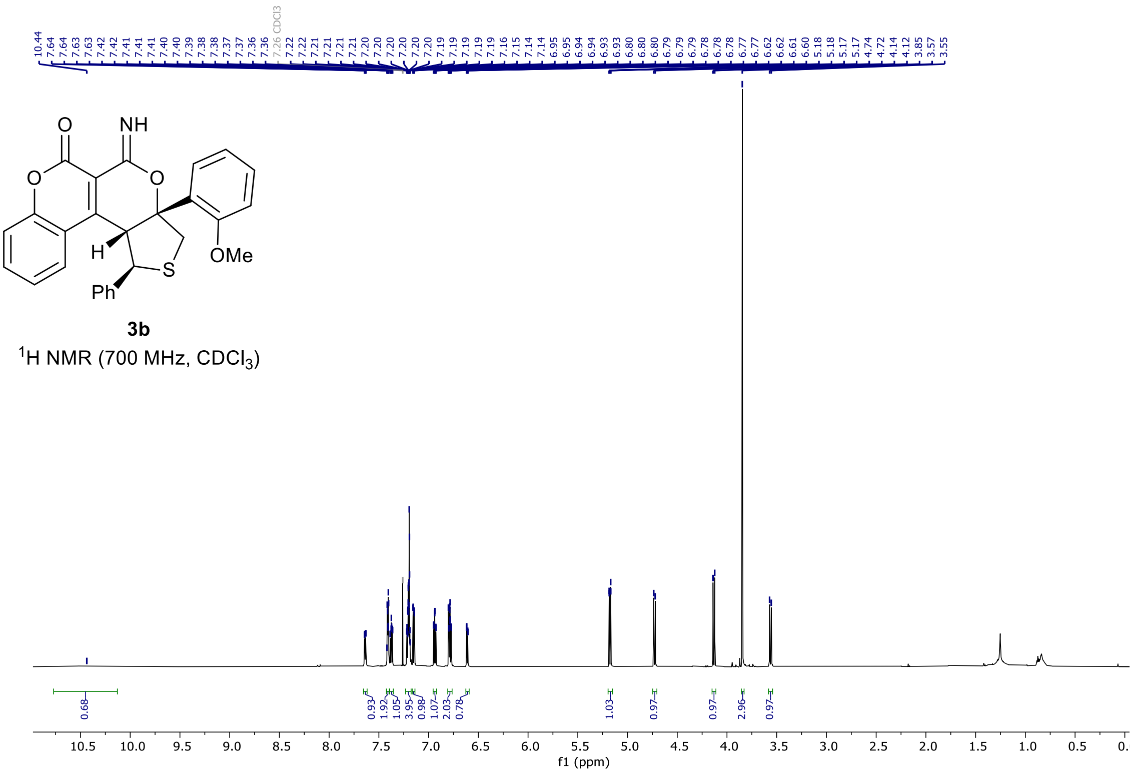

^{13}C NMR

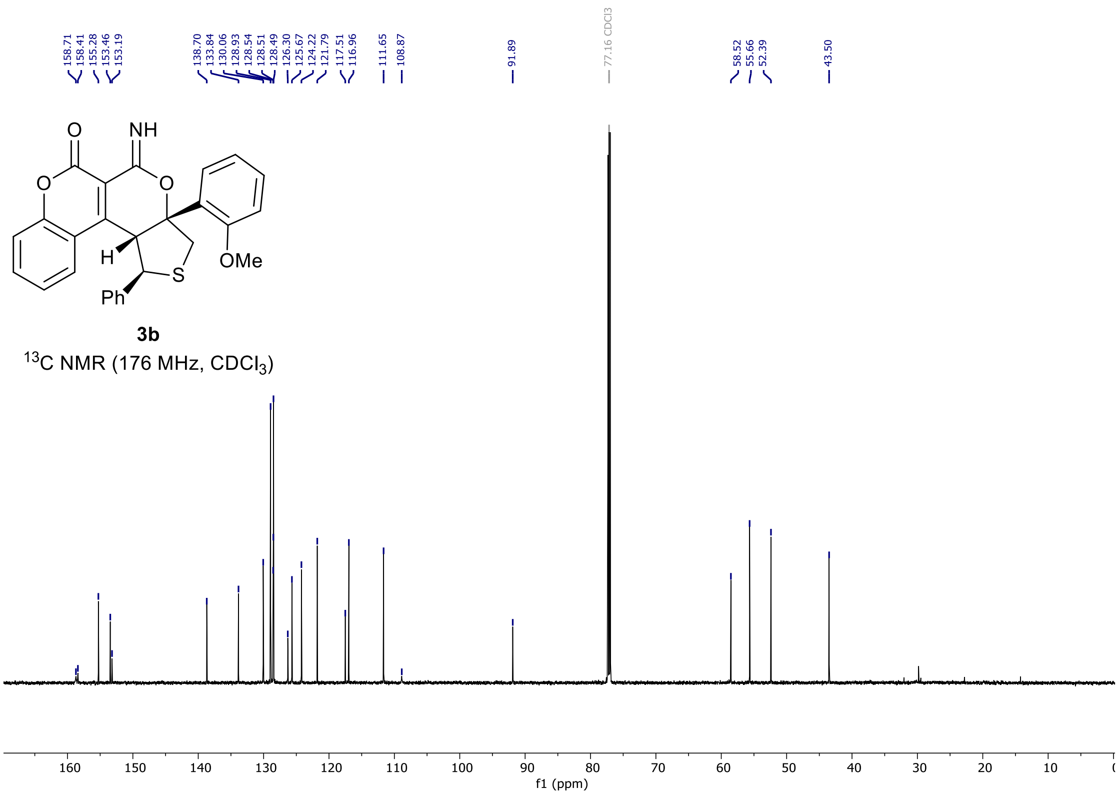

(1*R*,3a*R*,11c*R*)-5-Imino-3a-(3-methoxyphenyl)-1-phenyl-1,3,3a,11c-tetrahydrothieno[3',4':5,6]pyrano[3,4-c]chromen-6(5*H*)-one 3c

^{1}H NMR

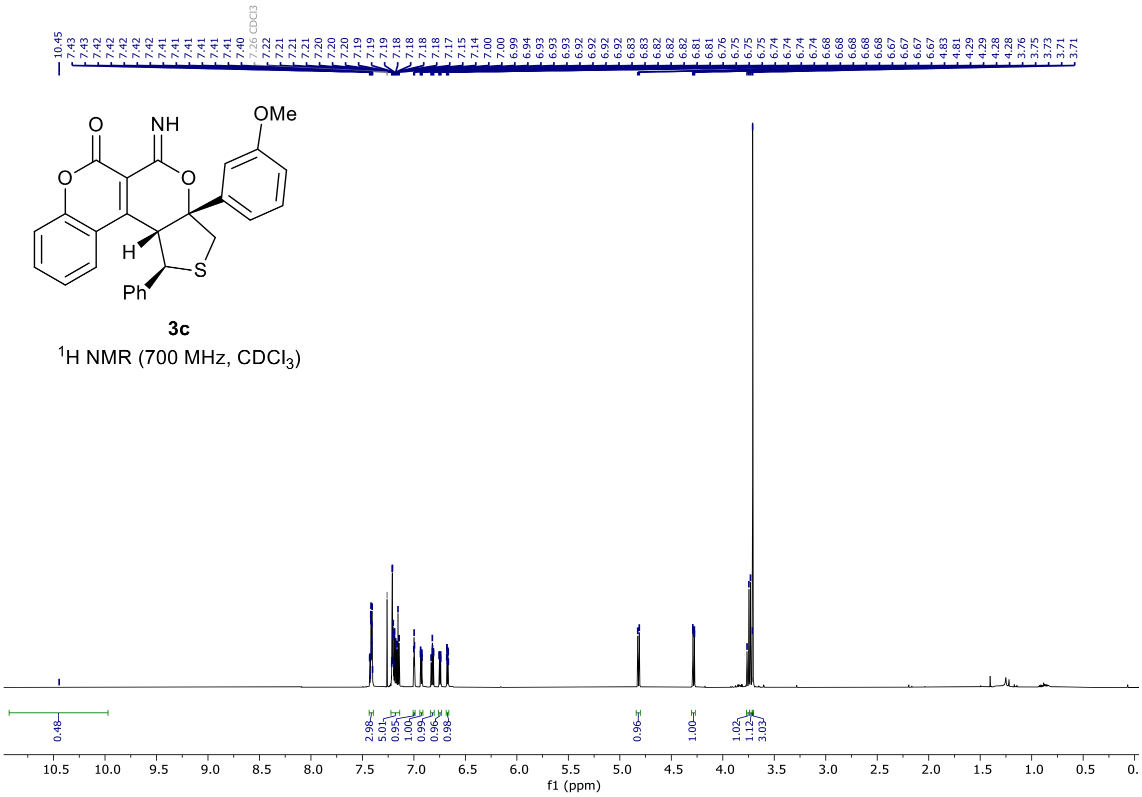

^{13}C NMR

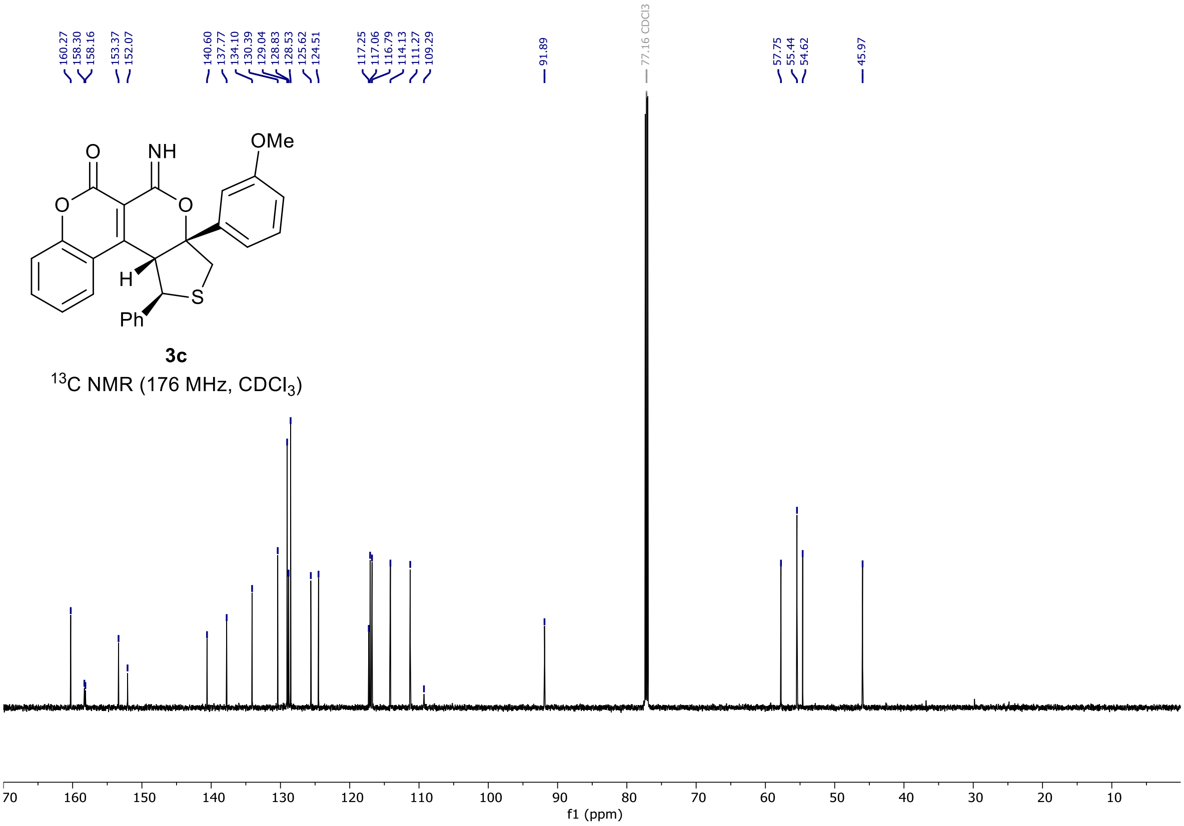

(1*R*,3a*R*,11c*R*)-5-Imino-1-phenyl-3a-(*p*-tolyl)-1,3,3a,11c-tetrahydrothieno[3',4':5,6]pyrano[3,4-c]chromen-6(5*H*)-one 3d

^{1}H NMR

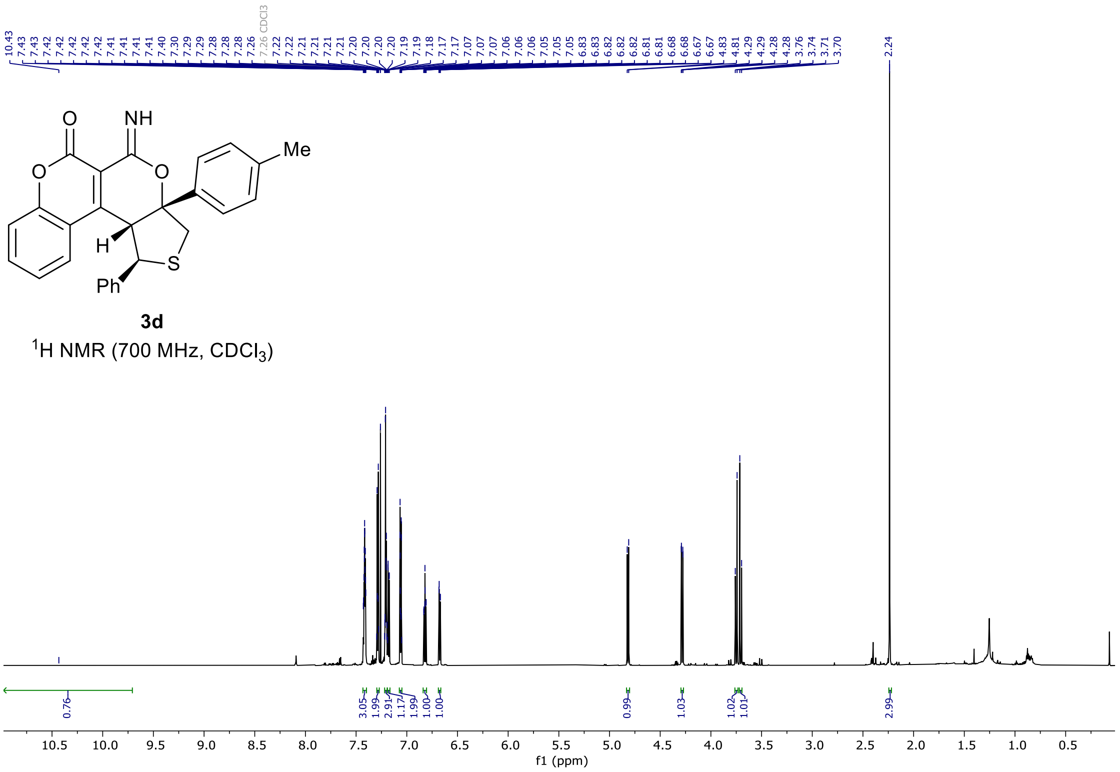

^{13}C NMR

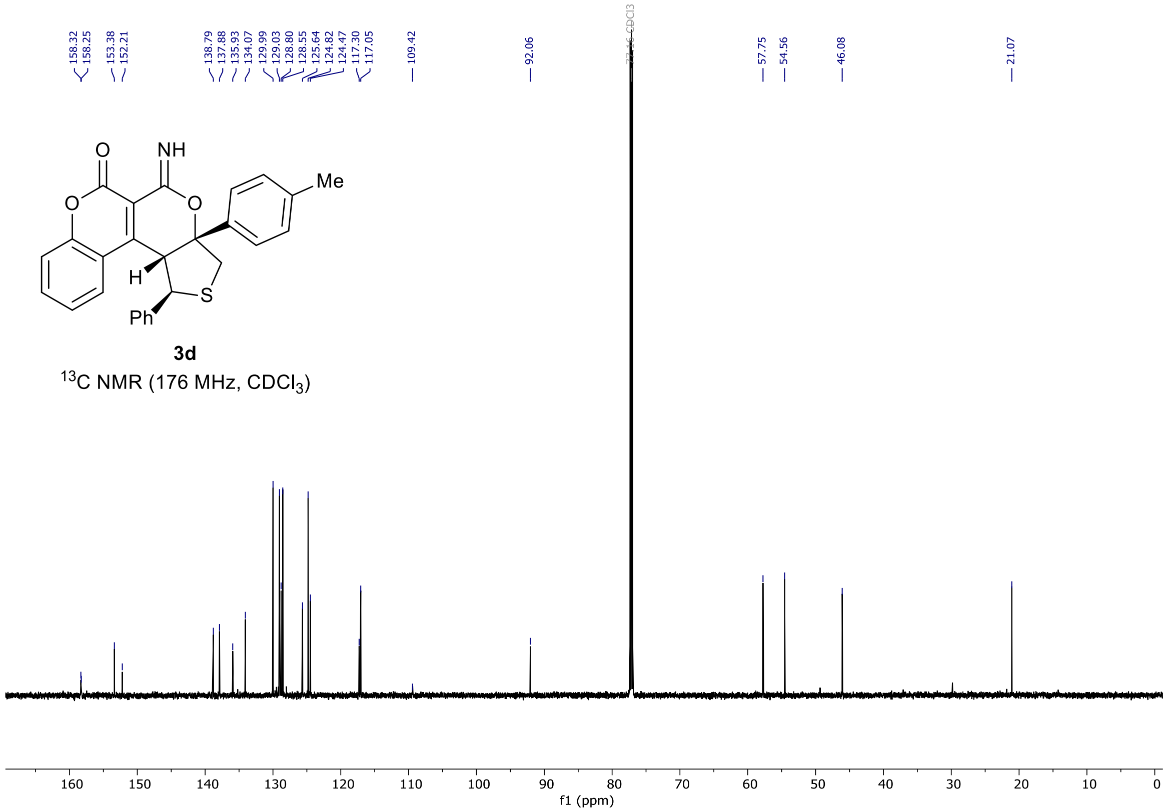

(1*R*,3a*R*,11c*R*)-3a-(2-Fluorophenyl)-5-imino-1-phenyl-1,3,3a,11c-tetrahydrothieno[3',4':5,6]pyrano[3,4-c]chromen-6(5*H*)-one 3e

^{1}H NMR

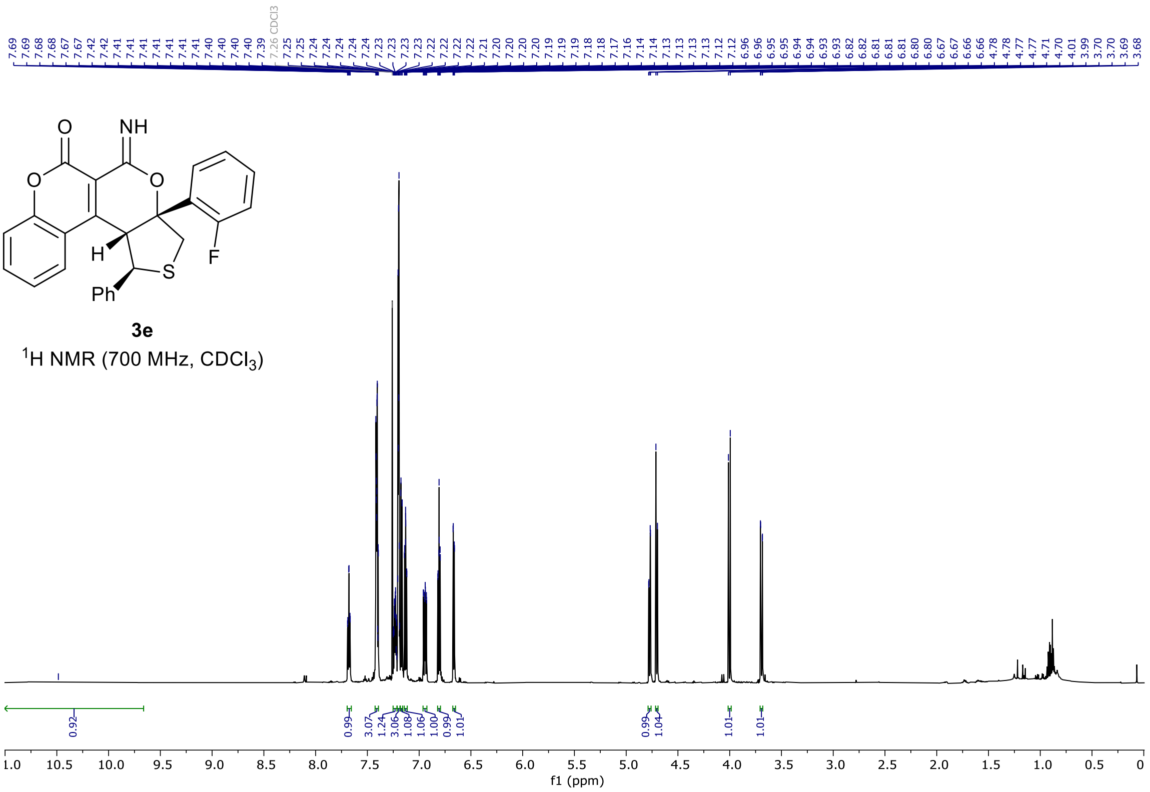

^{13}C NMR

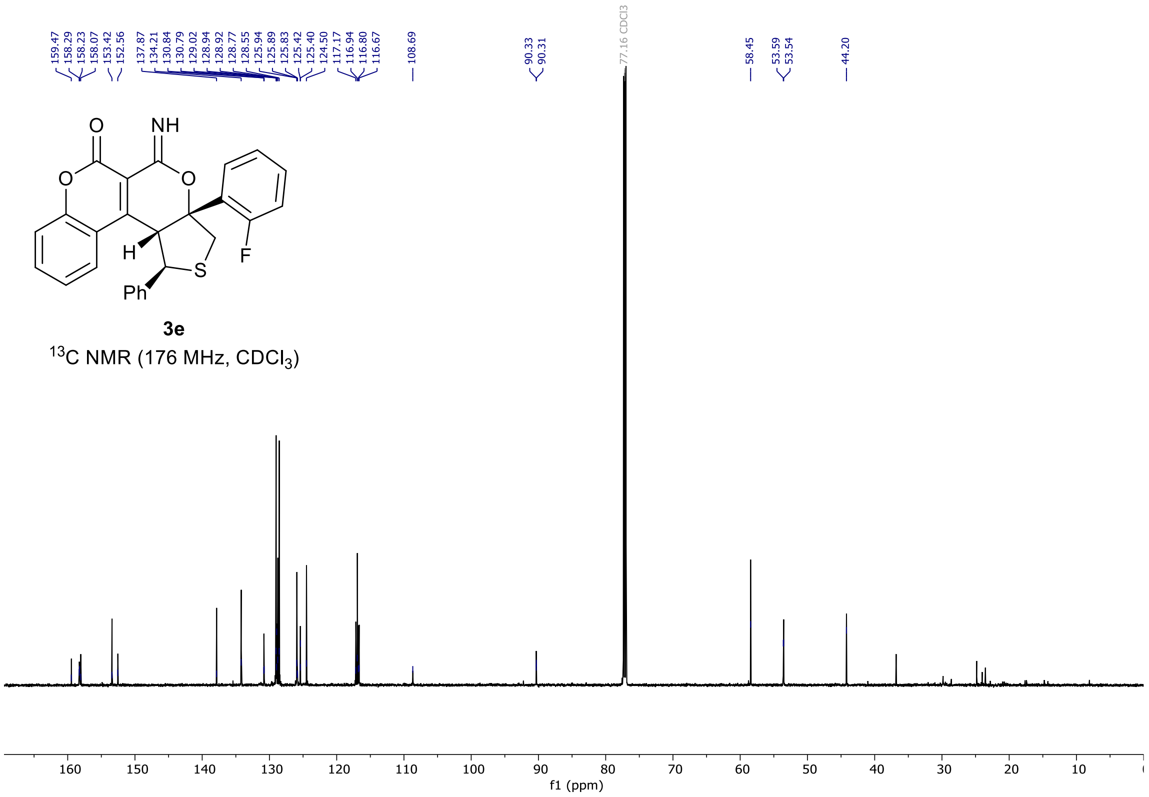

(1*R*,3a*R*,11c*R*)-3a-(4-Fluorophenyl)-5-imino-1-phenyl-1,3,3a,11c-tetrahydrothieno[3',4':5,6]pyrano[3,4-c]chromen-6(5*H*)-one 3f

^{1}H NMR

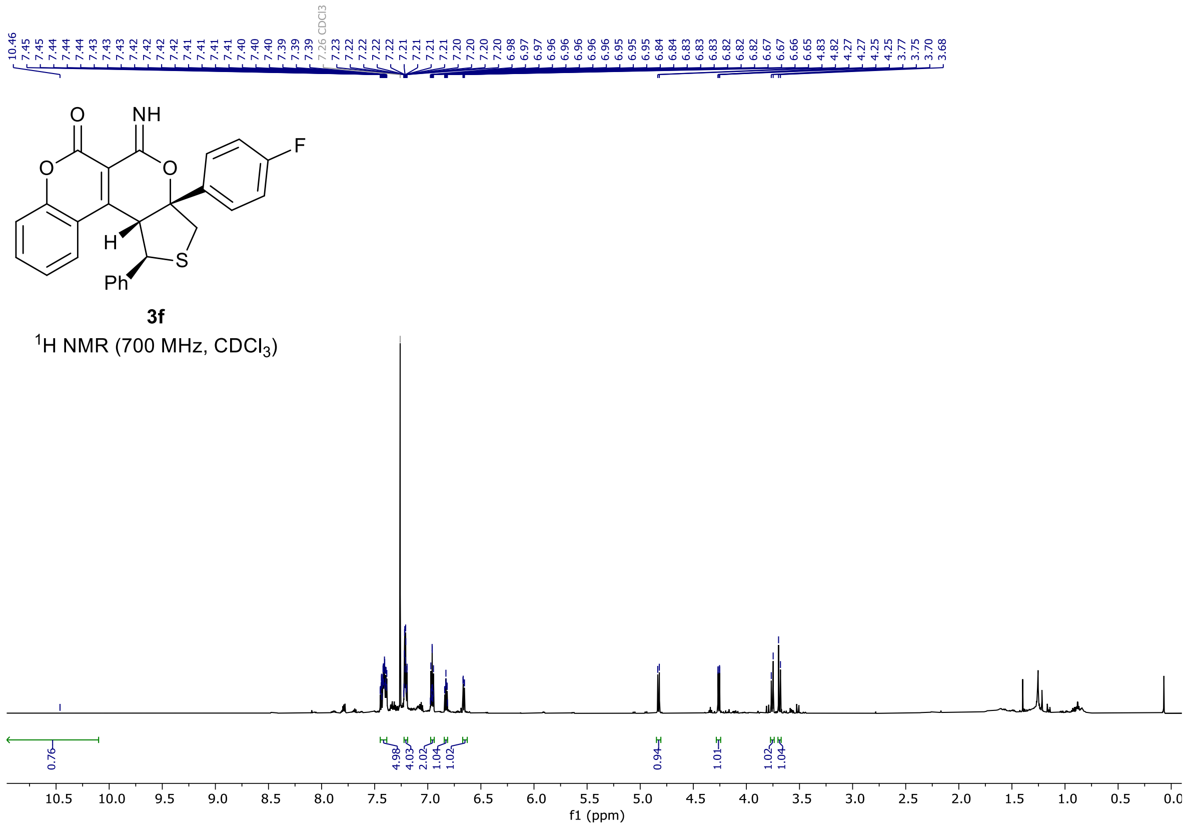

^{13}C NMR

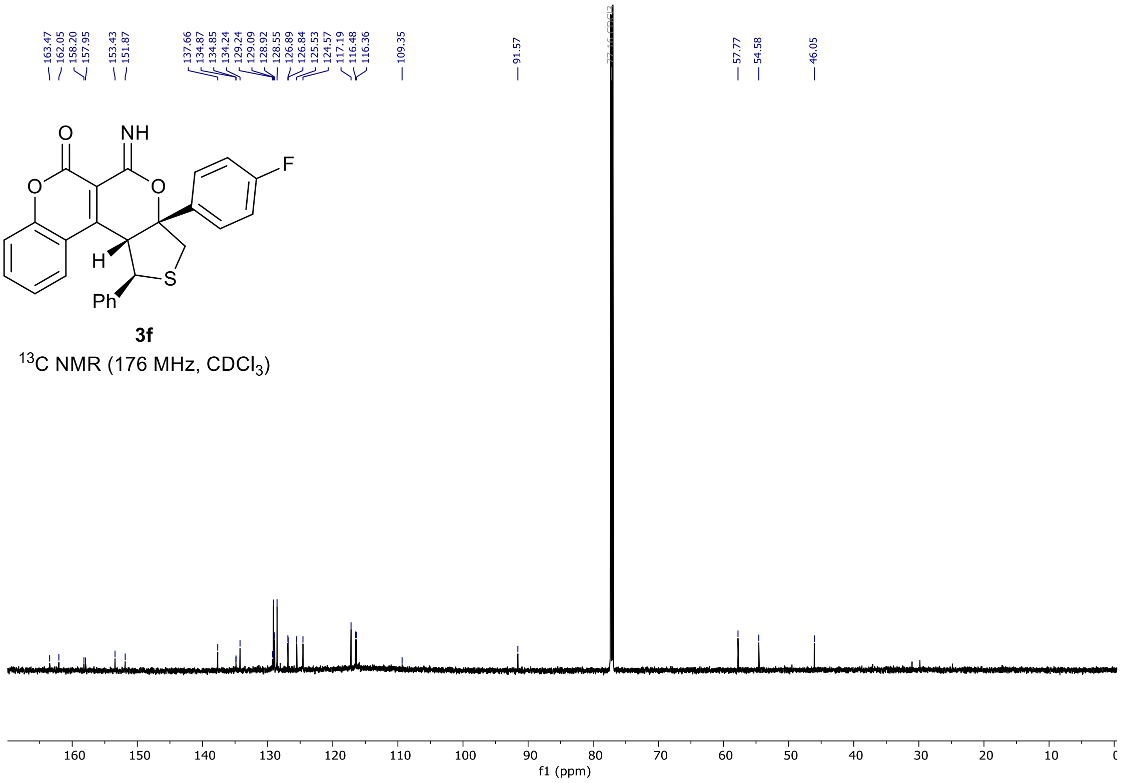

(1*R*,3a*R*,11c*R*)-5-Imino-1-phenyl-3a-(4-(trifluoromethyl)phenyl)-1,3,3a,11c-tetrahydrothieno[3',4':5,6]pyrano[3,4-c]chromen-6(5*H*)-one 3g

^{1}H NMR

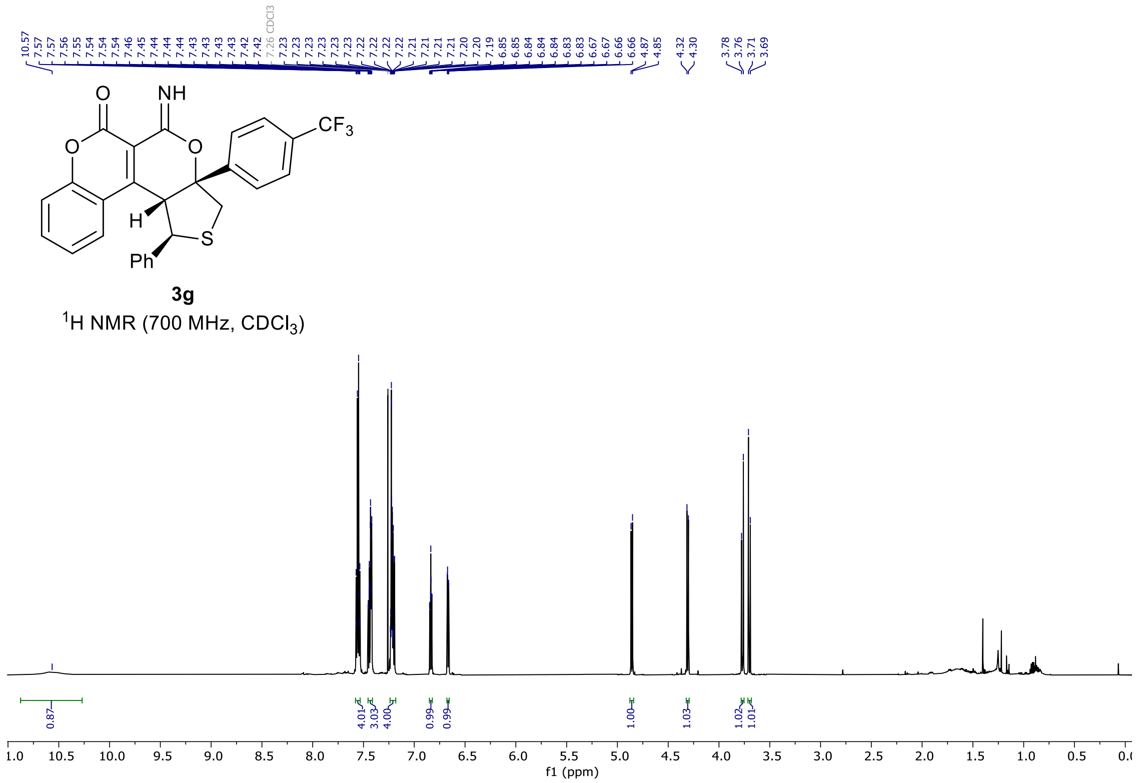

^{13}C NMR

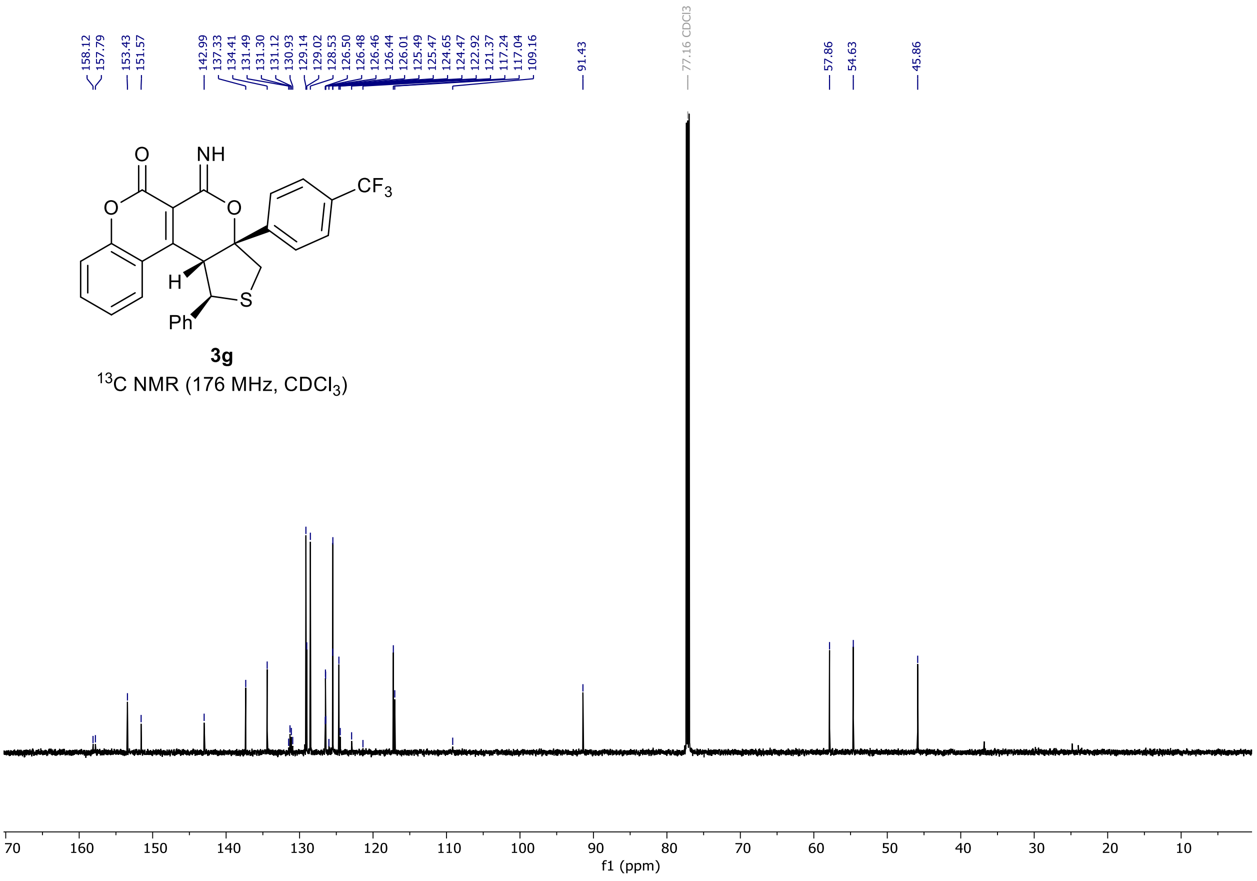

(1*R*,3a*R*,11c*R*)-5-Imino-3a-(naphthalen-2-yl)-1-phenyl-1,3,3a,11c-tetrahydrothieno[3',4':5,6]pyrano[3,4-c]chromen-6(5*H*)-one 3h

^{1}H NMR

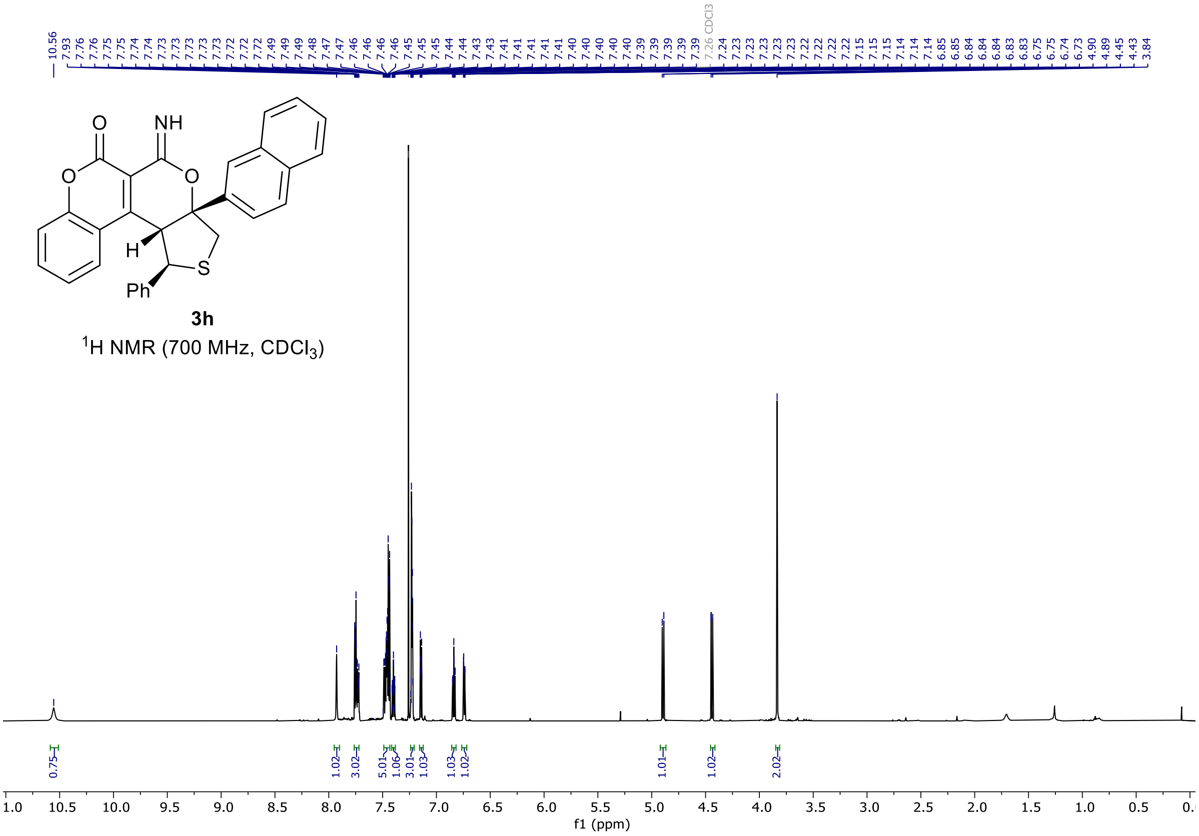

^{13}C NMR

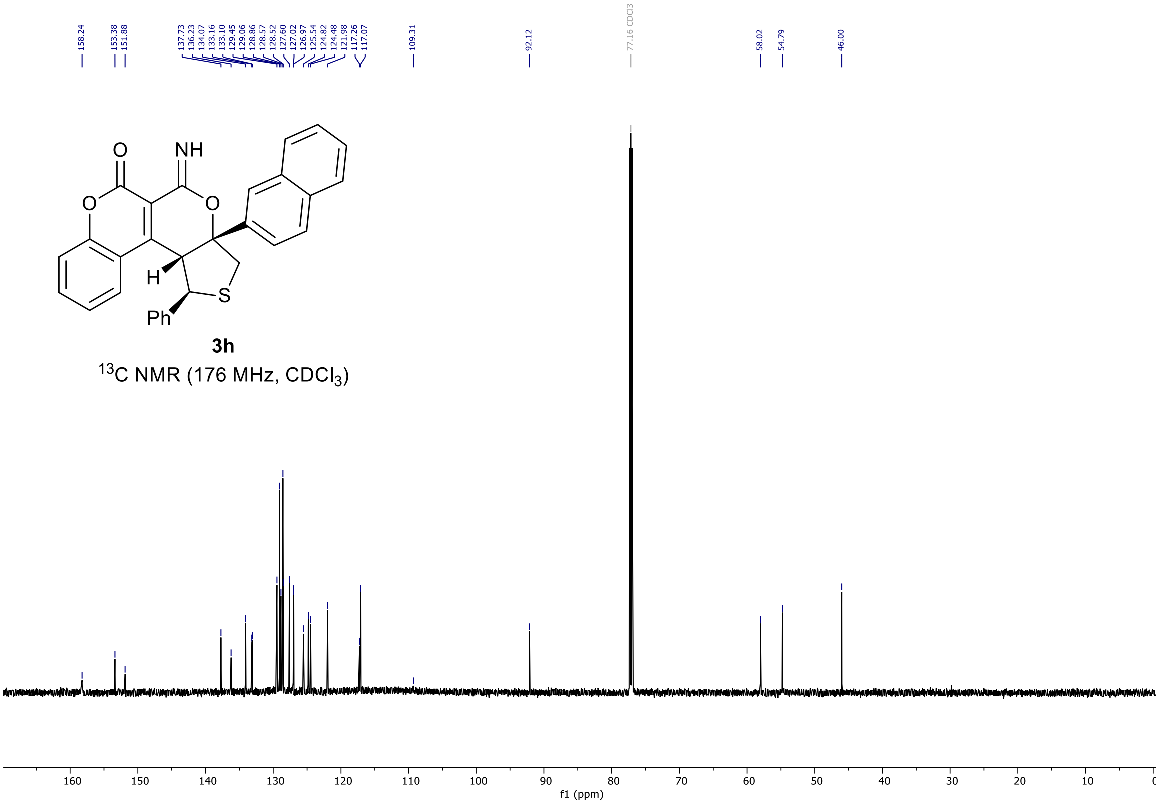

(1*R*,3a*R*,11c*R*)-5-Imino-1-(4-methoxyphenyl)-3a-phenyl-1,3,3a,11c-tetrahydrothieno[3',4':5,6]pyrano[3,4-c]chromen-6(5*H*)-one 3i

^{1}H NMR

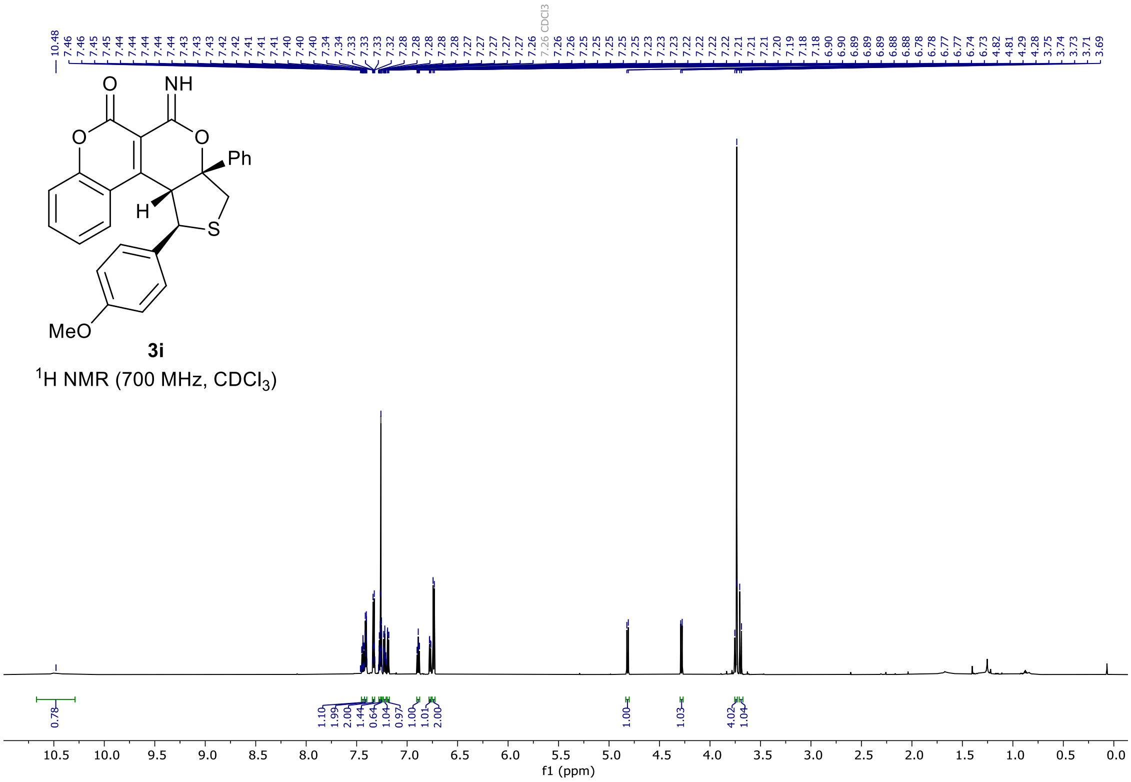

^{13}C NMR

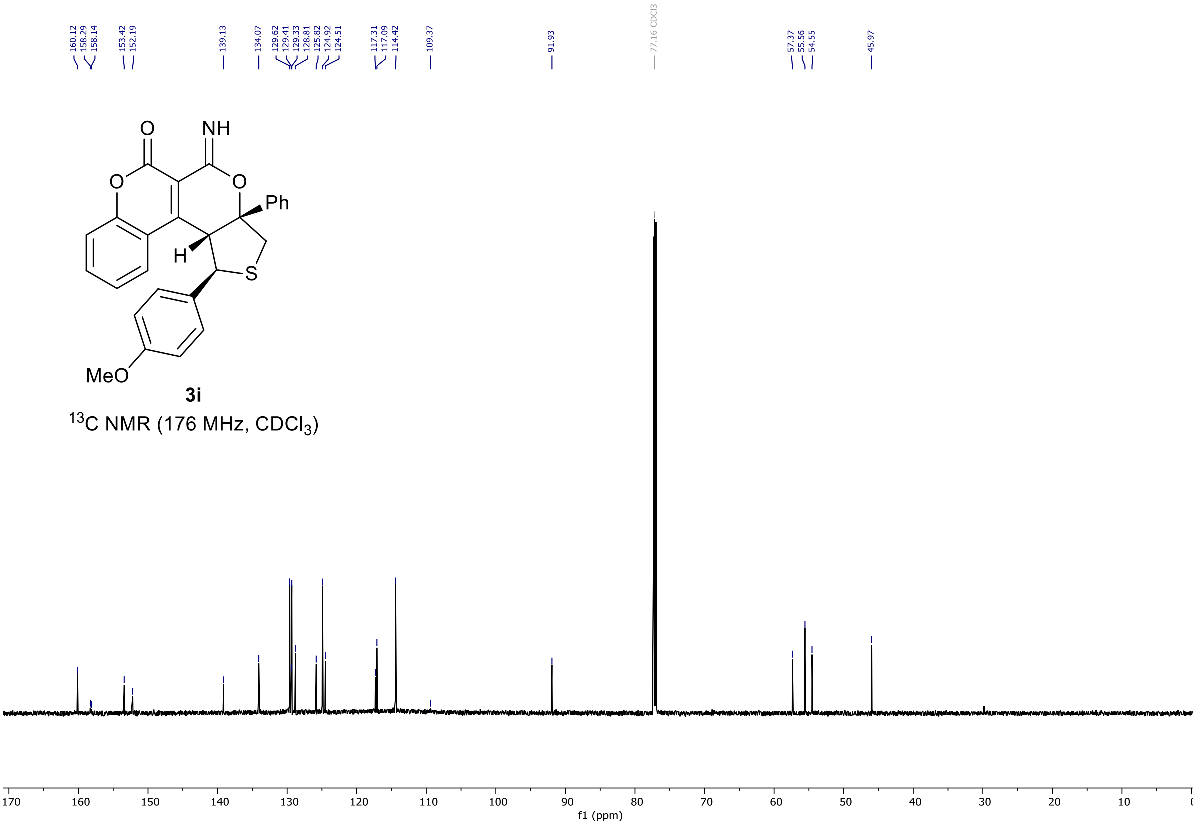

(1*R*,3a*R*,11c*R*)-5-Imino-3a-phenyl-1-(*m*-tolyl)-1,3,3a,11c-tetrahydrothieno[3',4':5,6]pyrano[3,4-c]chromen-6(5*H*)-one 3j

^{1}H NMR

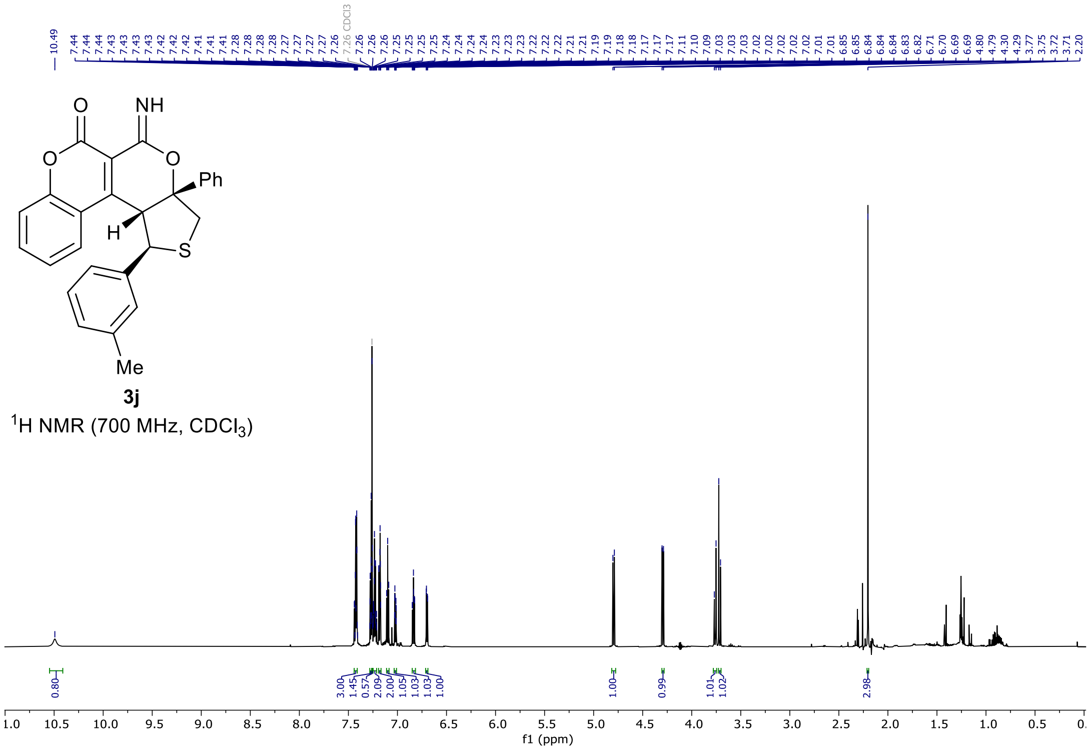

^{13}C NMR

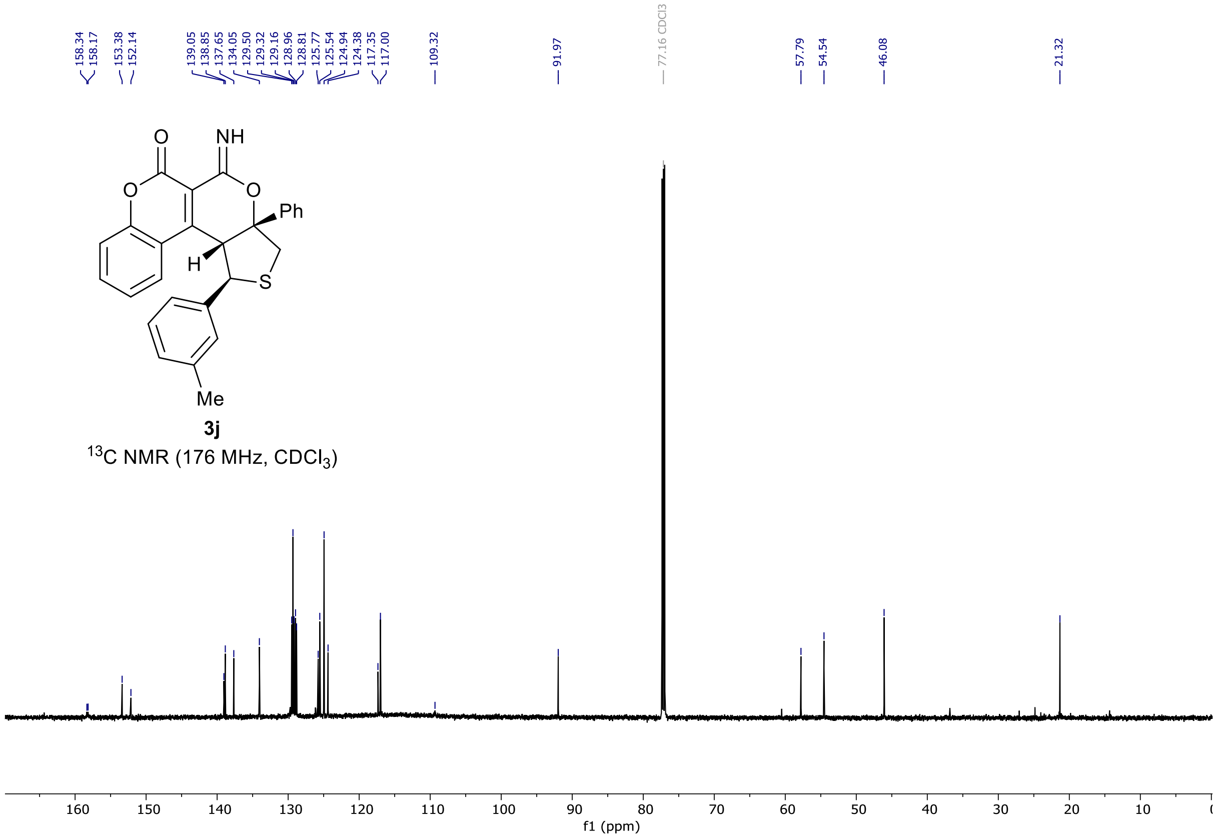

(1*R*,3a*R*,11c*R*)-5-Imino-3a-phenyl-1-(*p*-tolyl)-1,3,3a,11c-tetrahydrothieno[3',4':5,6]pyrano[3,4-c]chromen-6(5*H*)-one 3k

^{1}H NMR

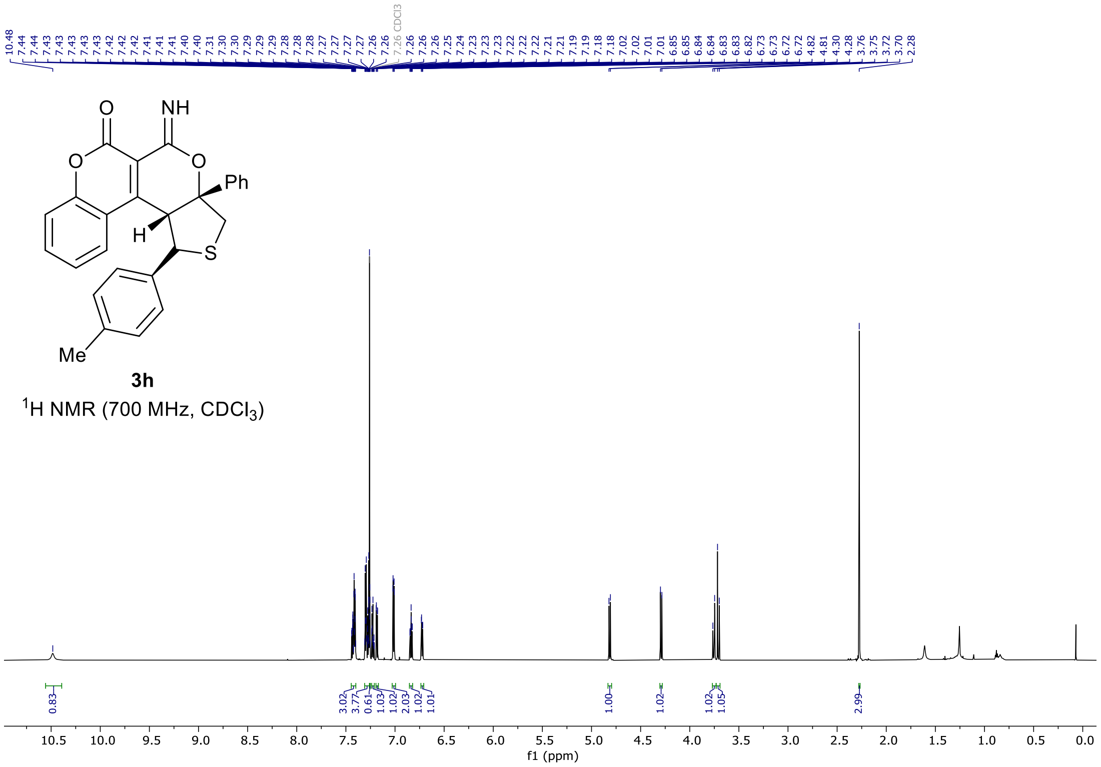

^{13}C NMR

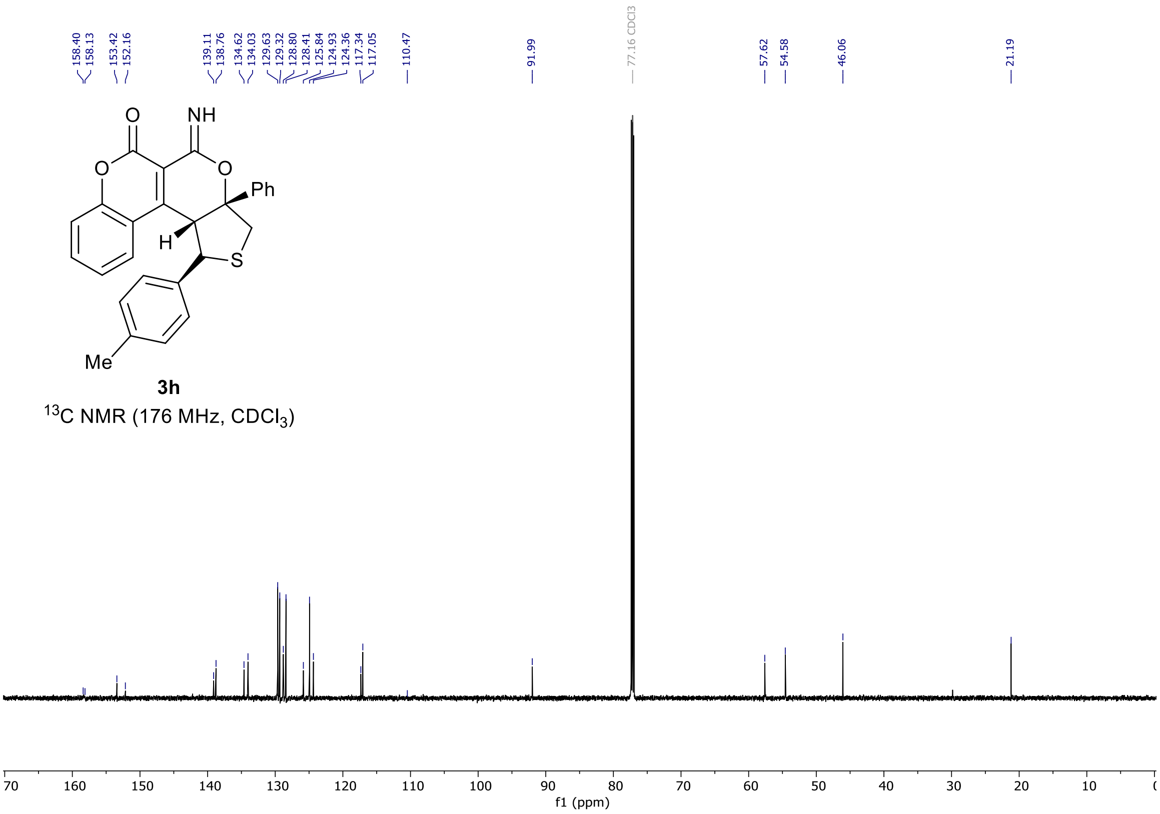

1*R*,3a*R*,11c*R*)-1-(3-Chlorophenyl)-5-imino-3a-phenyl-1,3,3a,11c-tetrahydrothieno[3',4':5,6]pyrano[3,4-c]chromen-6(5*H*)-one 3l

^{1}H NMR

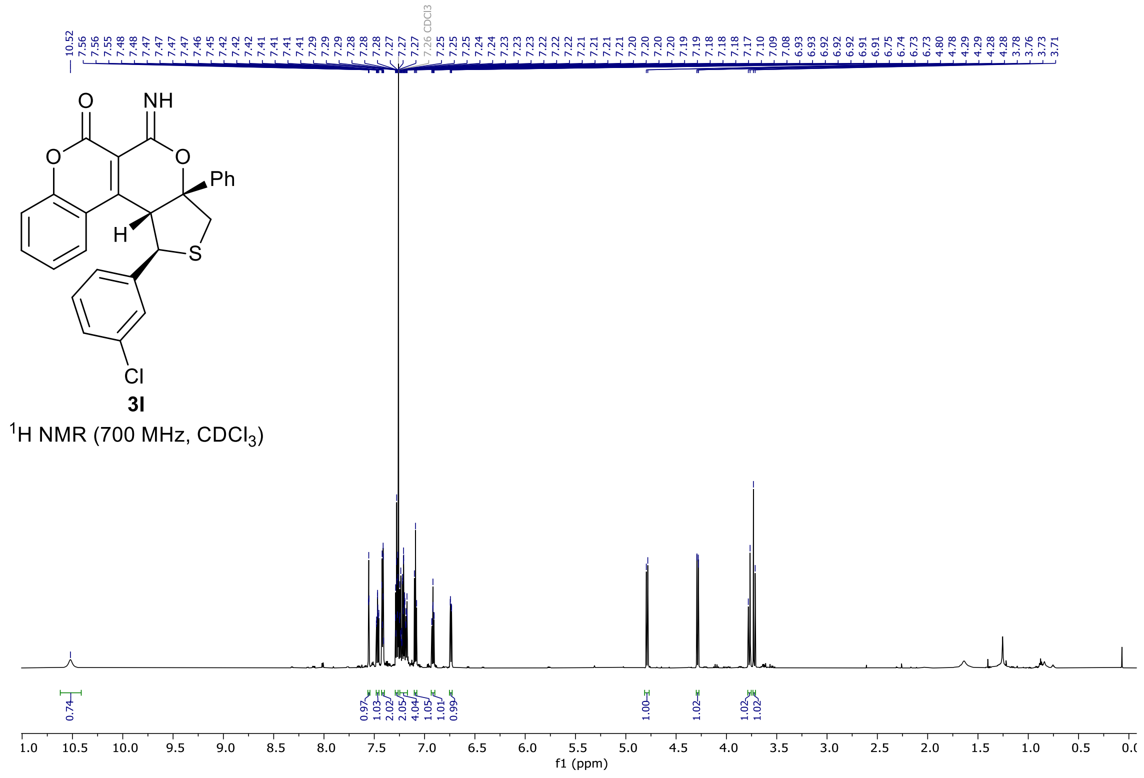

^{13}C NMR

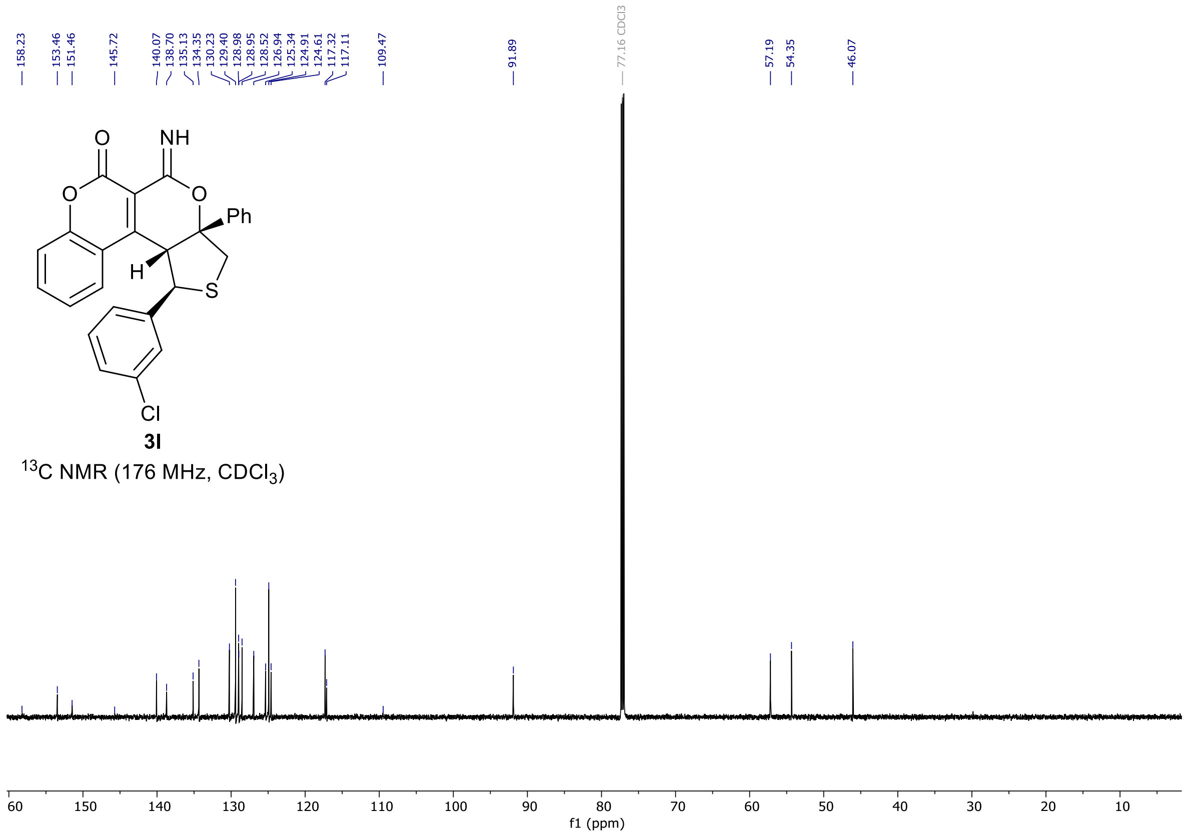

(1*R*,3a*R*,11c*R*)-1-(4-Chlorophenyl)-5-imino-3a-phenyl-1,3,3a,11c-tetrahydrothieno[3',4':5,6]pyrano[3,4-c]chromen-6(5*H*)-one 3m

^{1}H NMR

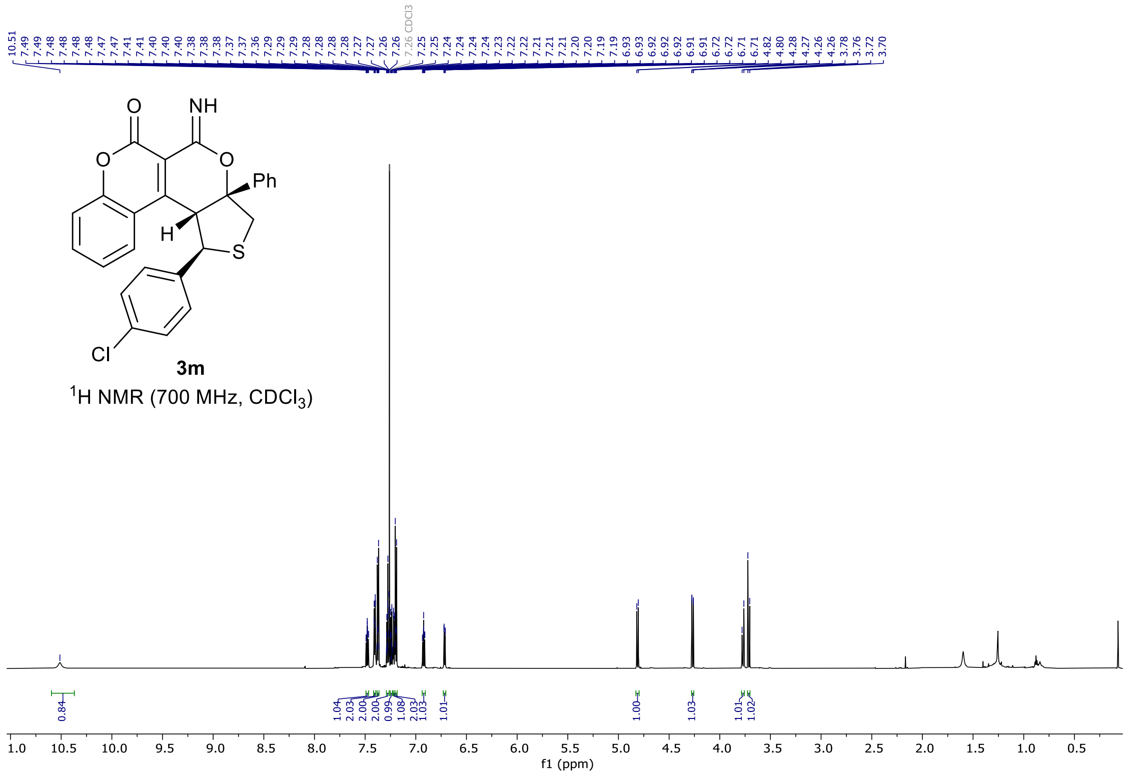

^{13}C NMR

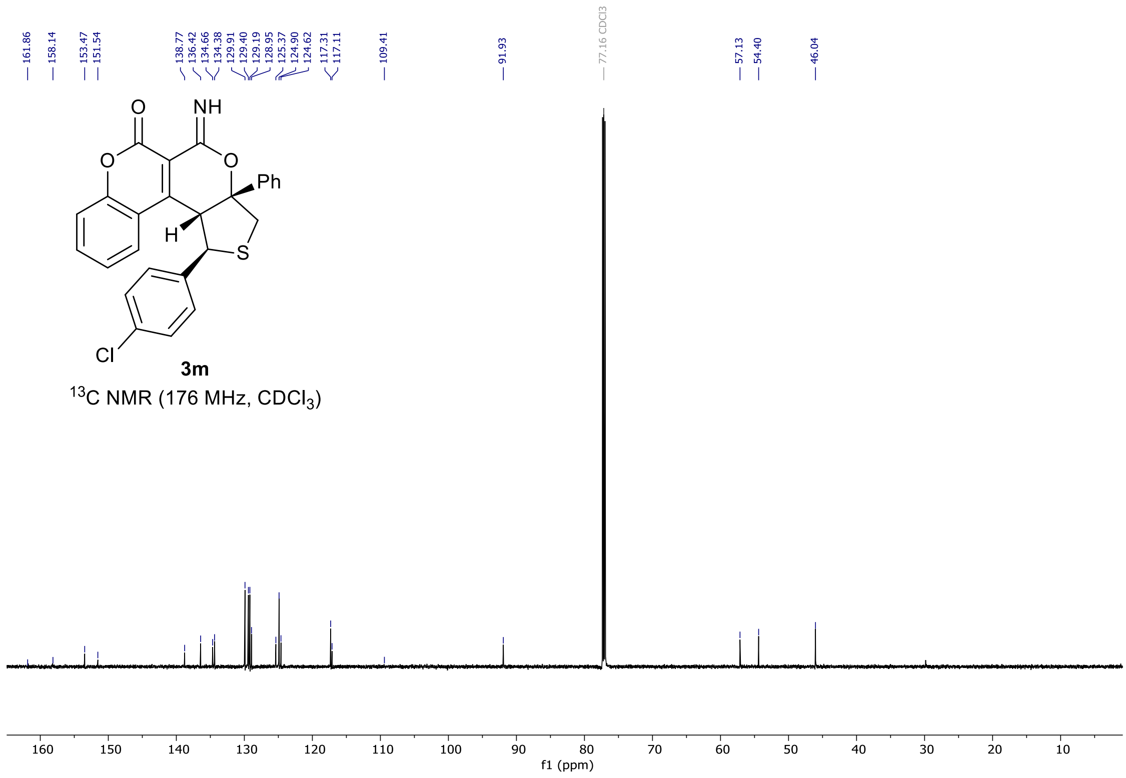

(1*R*,3a*R*,11c*R*)-5-Imino-1-(4-nitrophenyl)-3a-phenyl-1,3,3a,11c-tetrahydrothieno[3',4':5,6]pyrano[3,4-c]chromen-6(5*H*)-one 3n

^{1}H NMR

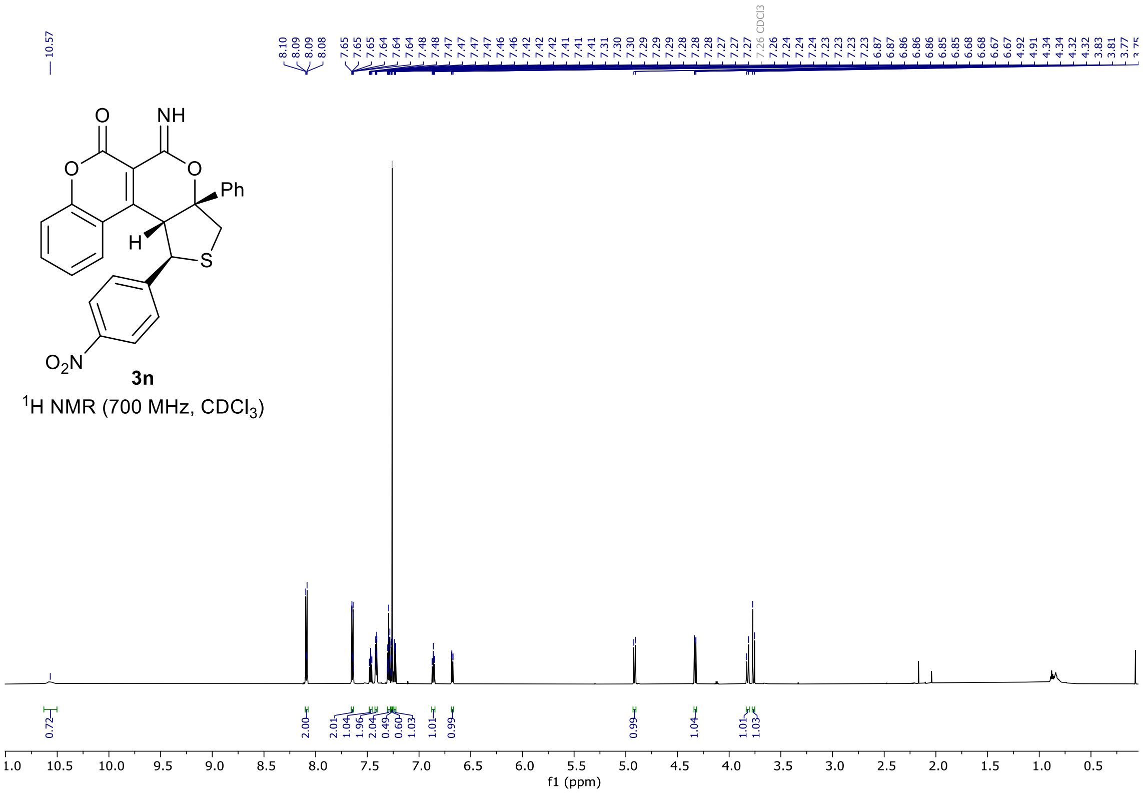

10.57
8.10 8.09 8.09 8.08
7.65 7.65 7.65 7.64 7.64 7.64 7.64 7.48 7.47 7.47 7.47 7.46 7.46 7.42 7.42 7.42 7.41 7.41 7.41 7.31 7.30 7.29 7.29 7.29 7.28 7.28 7.28 7.27 7.27 7.26 CDCl3 7.26 7.24 7.24 7.24 7.23 7.23 7.23 7.23 6.87 6.87 6.86 6.86 6.86 6.85 6.85 6.68 6.68 6.67 6.67 4.92 4.91 4.34 4.34 4.32 4.32 3.83 3.81 3.77
O
NH
O
O
Ph
H
S
O_2N
3n
^{1}H NMR (700 MHz, CDCl$_3$)
0.72 2.00 2.01 1.04 1.96 2.04 0.49 0.60 1.03 1.01 0.99 0.99 1.04 1.01 1.03
1.0 10.5 10.0 9.5 9.0 8.5 8.0 7.5 7.0 6.5 6.0 5.5 5.0 4.5 4.0 3.5 3.0 2.5 2.0 1.5 1.0 0.5
f1 (ppm)

^{13}C NMR

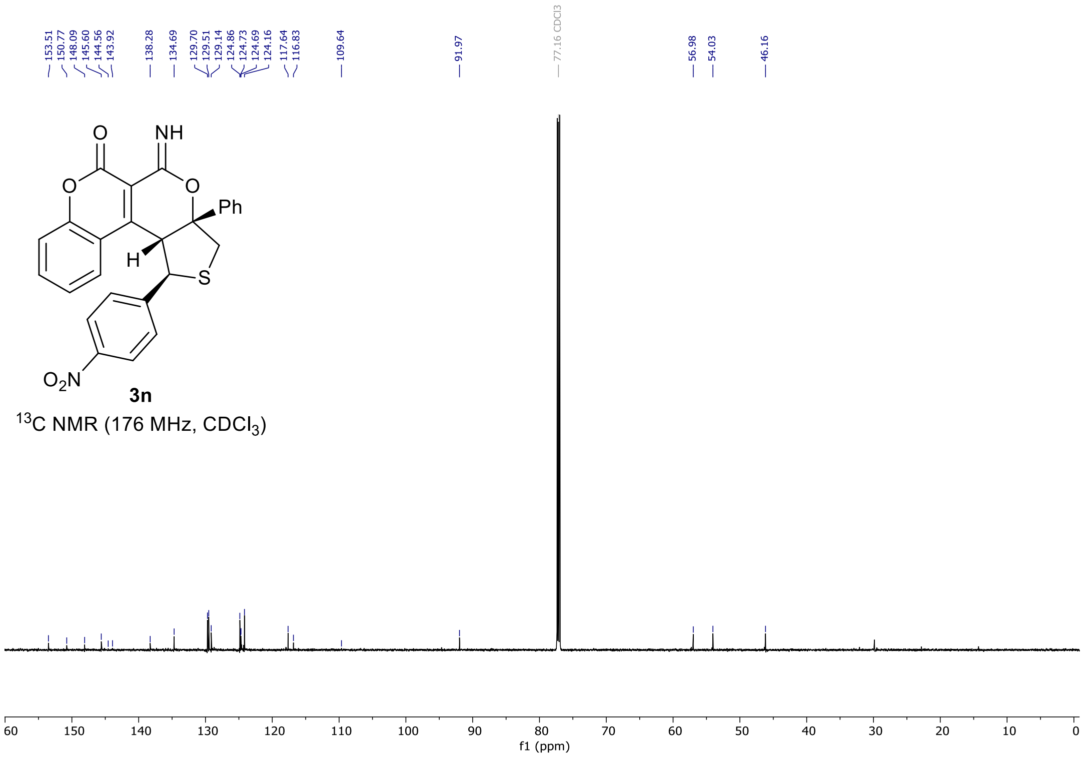

153.51 150.77 148.09 145.60 144.56 143.92 138.28 134.69 129.70 129.51 129.14 124.86 124.73 124.69 124.16 117.64 116.83 109.64 91.97 77.16 CDCl3 56.98 54.03 46.16
O
NH
O
O
Ph
H
S
O_2N
3n
^{13}C NMR (176 MHz, CDCl$_3$)
60 150 140 130 120 110 100 90 80 70 60 50 40 30 20 10 0
f1 (ppm)

(1*R*,3a*R*,11c*R*)-5-Imino-3a-phenyl-1-(4-(trifluoromethyl)phenyl)-1,3,3a,11c-tetrahydrothieno[3',4':5,6]pyrano[3,4-c]chromen-6(5*H*)-one 3o

^{1}H NMR

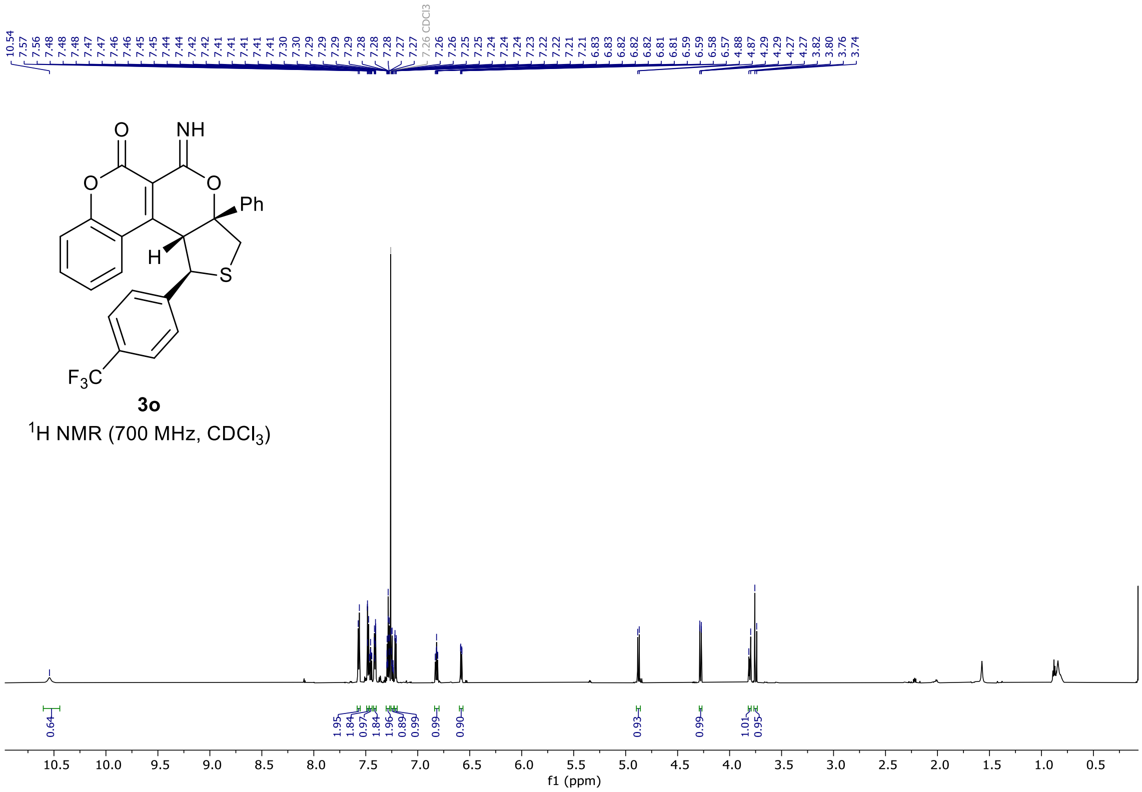

^{13}C NMR

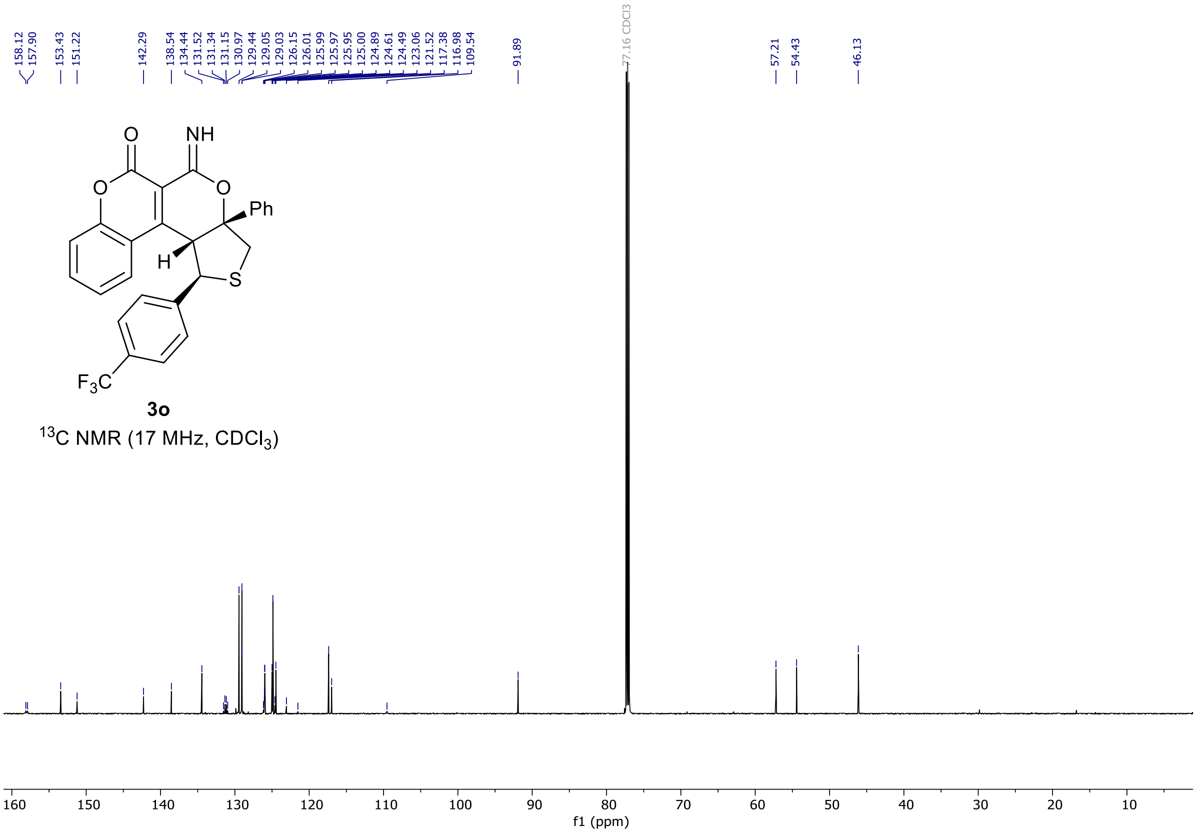

(1*R*,3a*R*,11c*R*)-5-Imino-9-methoxy-1,3a-diphenyl-1,3,3a,11c-tetrahydrothieno[3',4':5,6]pyrano[3,4-c]chromen-6(5*H*)-one 3p

^{1}H NMR

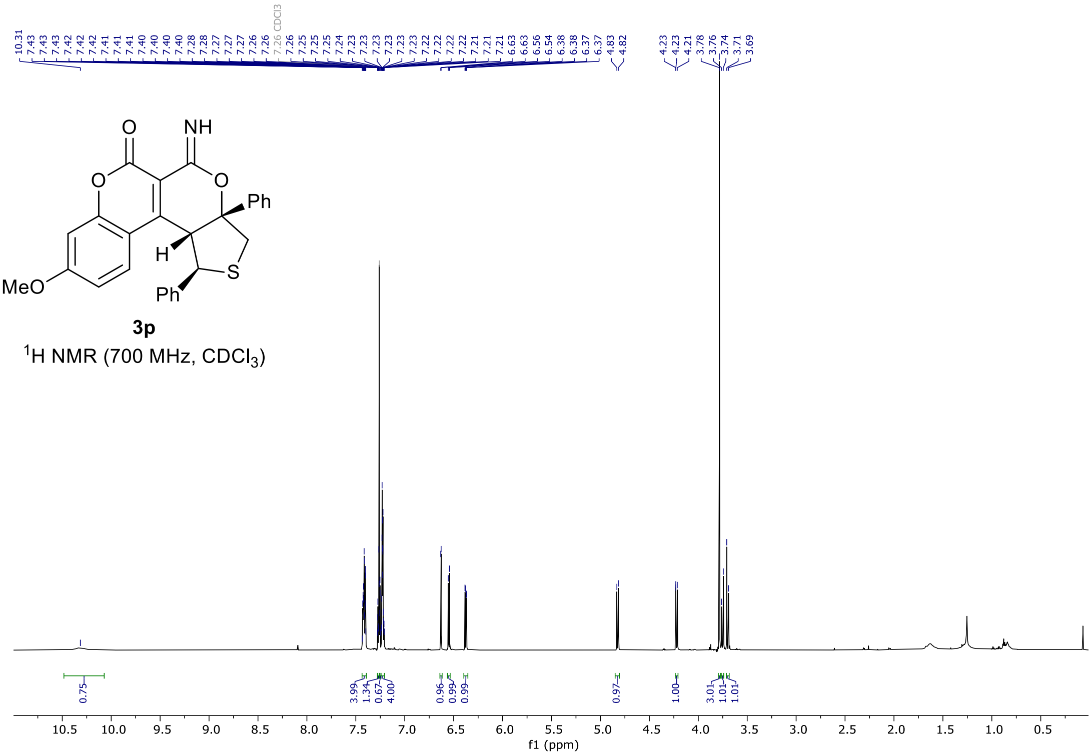

^{13}C NMR

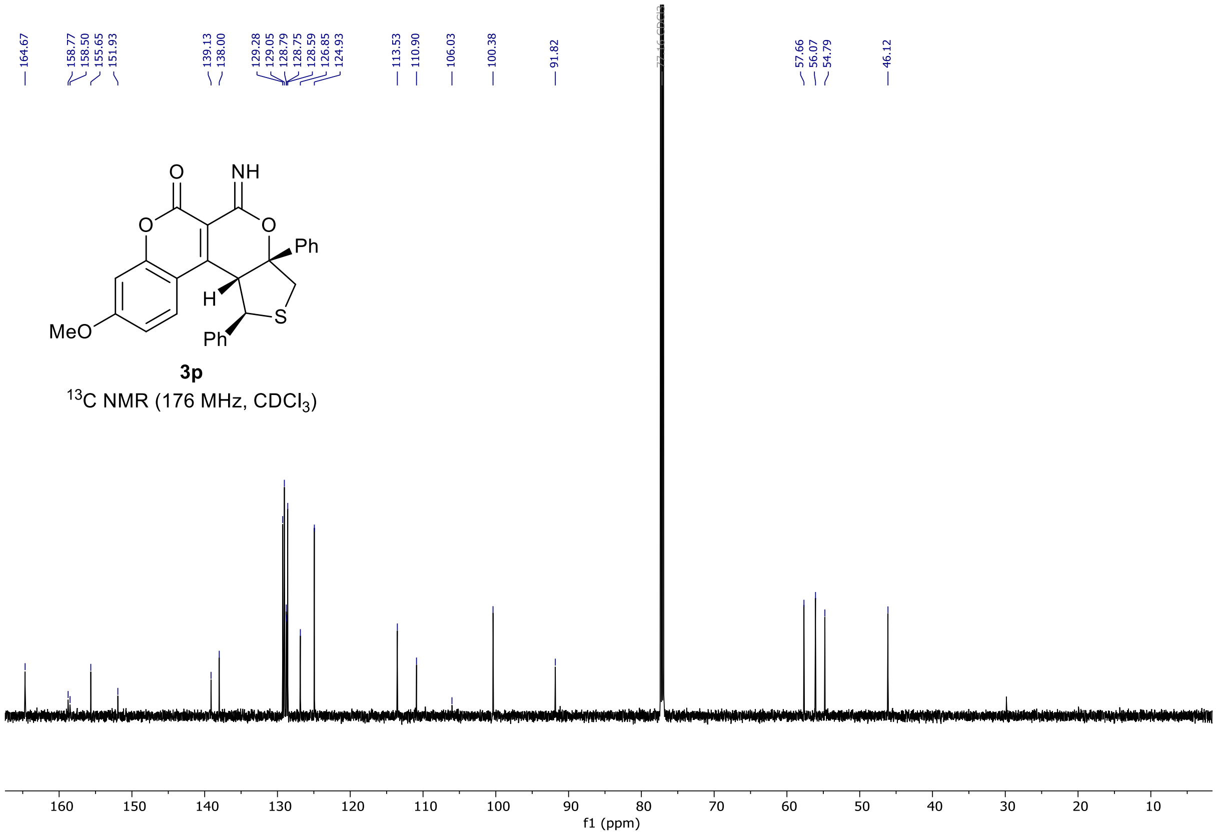

(1*R*,3a*R*,11c*R*)-10-Bromo-5-imino-1,3a-diphenyl-1,3,3a,11c-tetrahydrothieno[3',4':5,6]pyrano[3,4-c]chromen-6(5*H*)-one 3q

^{1}H NMR

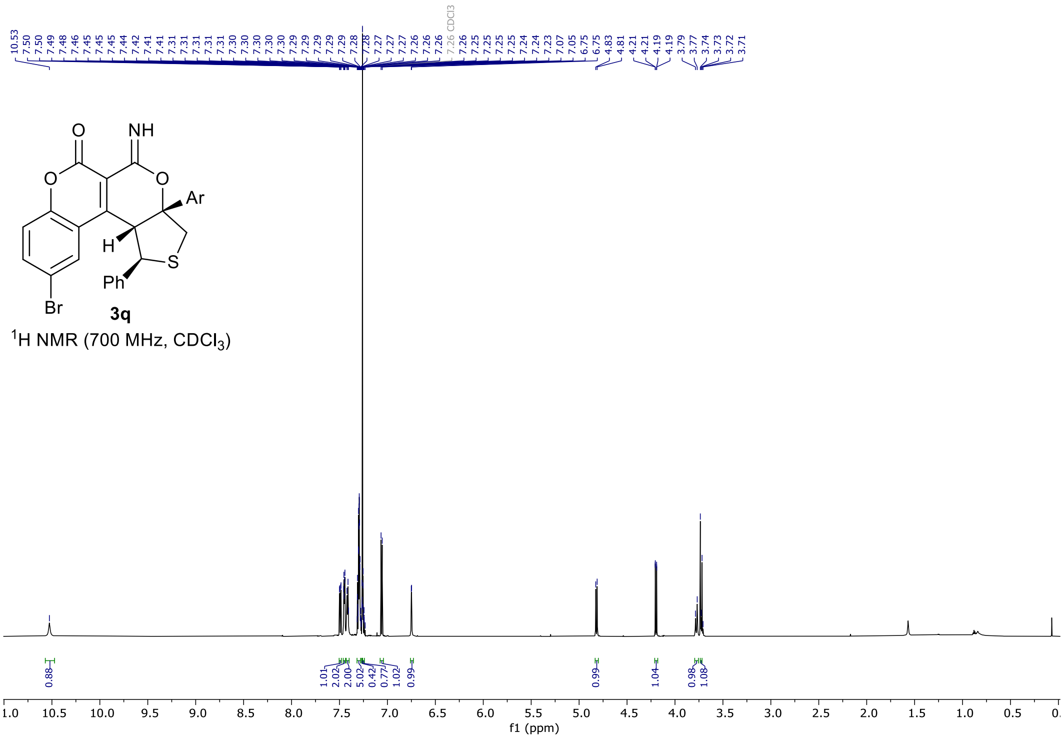

^{13}C NMR

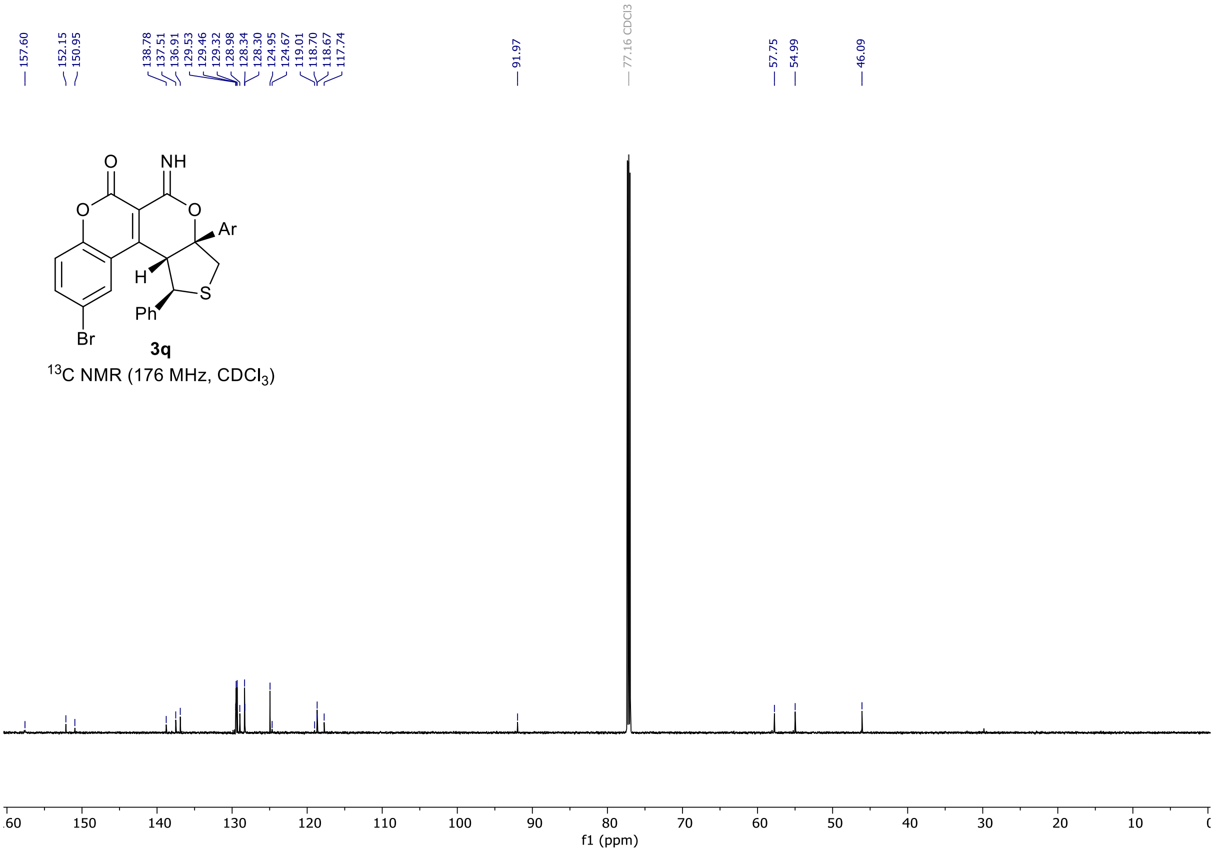

(1*R*,3a*R*,11c*R*)-1,3a-Diphenyl-1,3,3a,11c-tetrahydrothieno[3',4':5,6]pyrano[3,4-c]chromene-5,6-dione 4a

^{1}H NMR

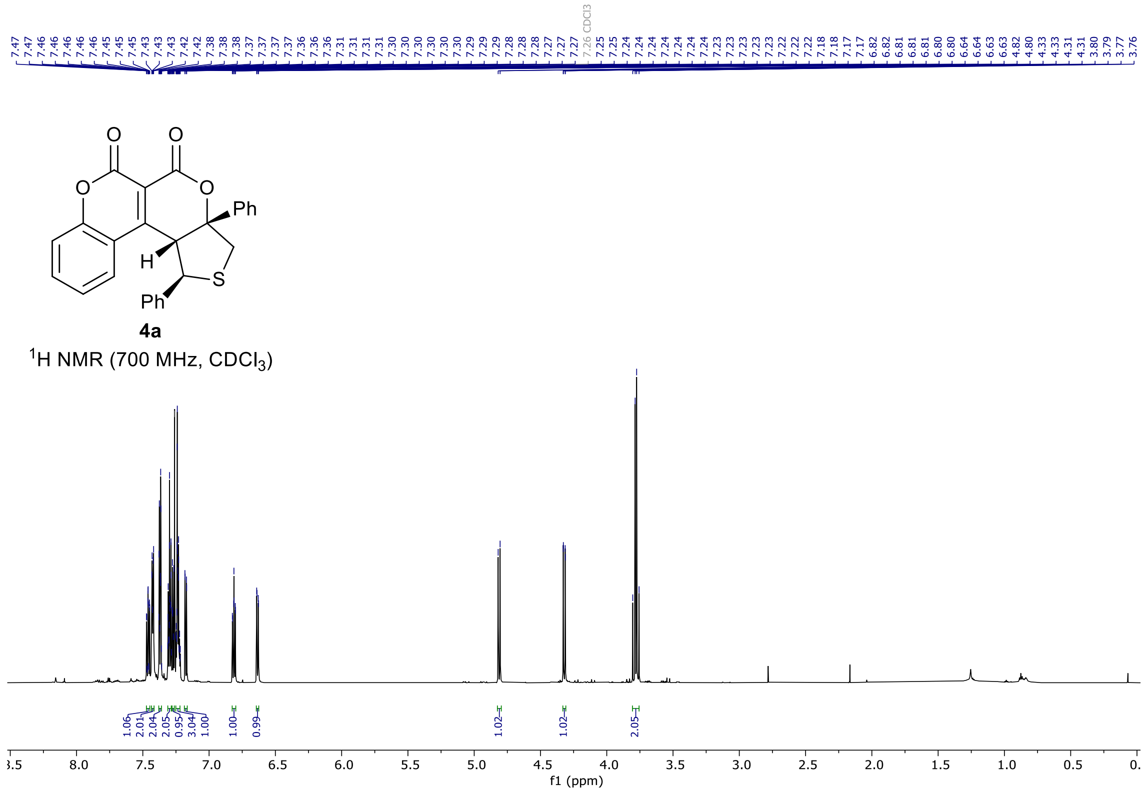

^{13}C NMR

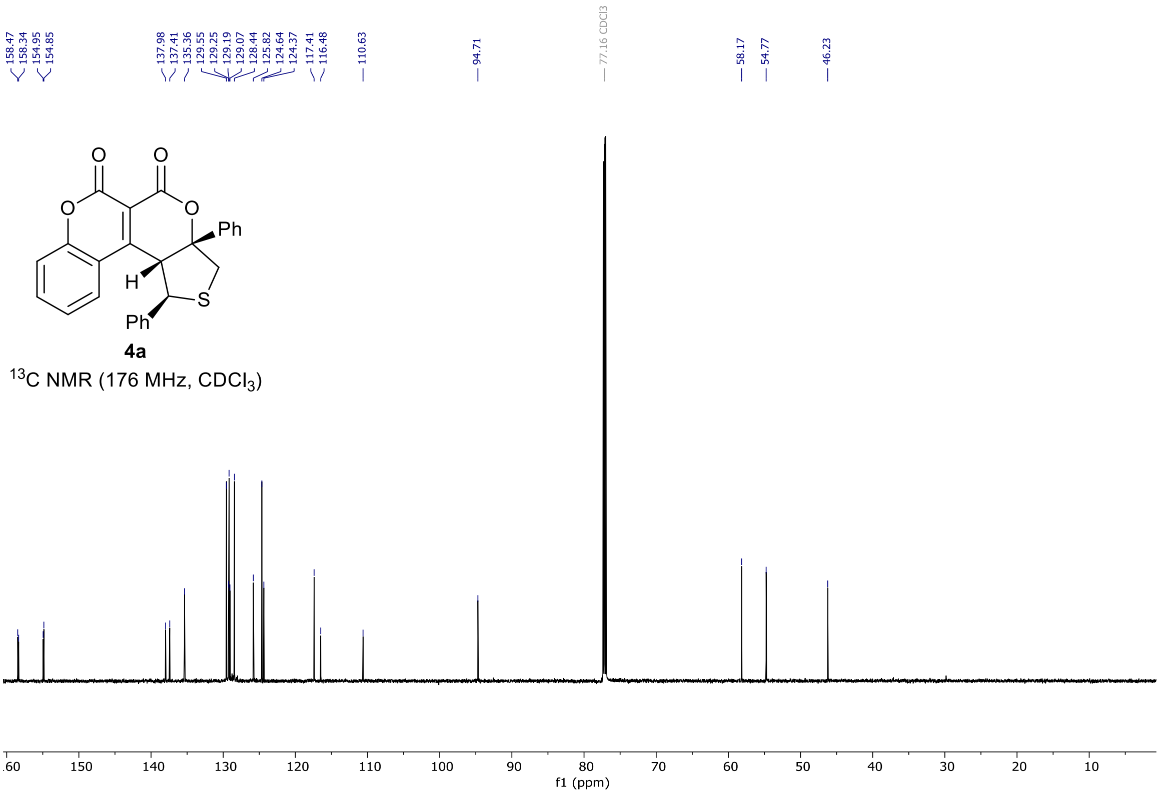

(1*R*,3a*R*,11c*R*)-3a-(2-Methoxyphenyl)-1-phenyl-1,3,3a,11c-tetrahydrothieno[3',4':5,6]pyrano[3,4-c]chromene-5,6-dione 4b

^{1}H NMR

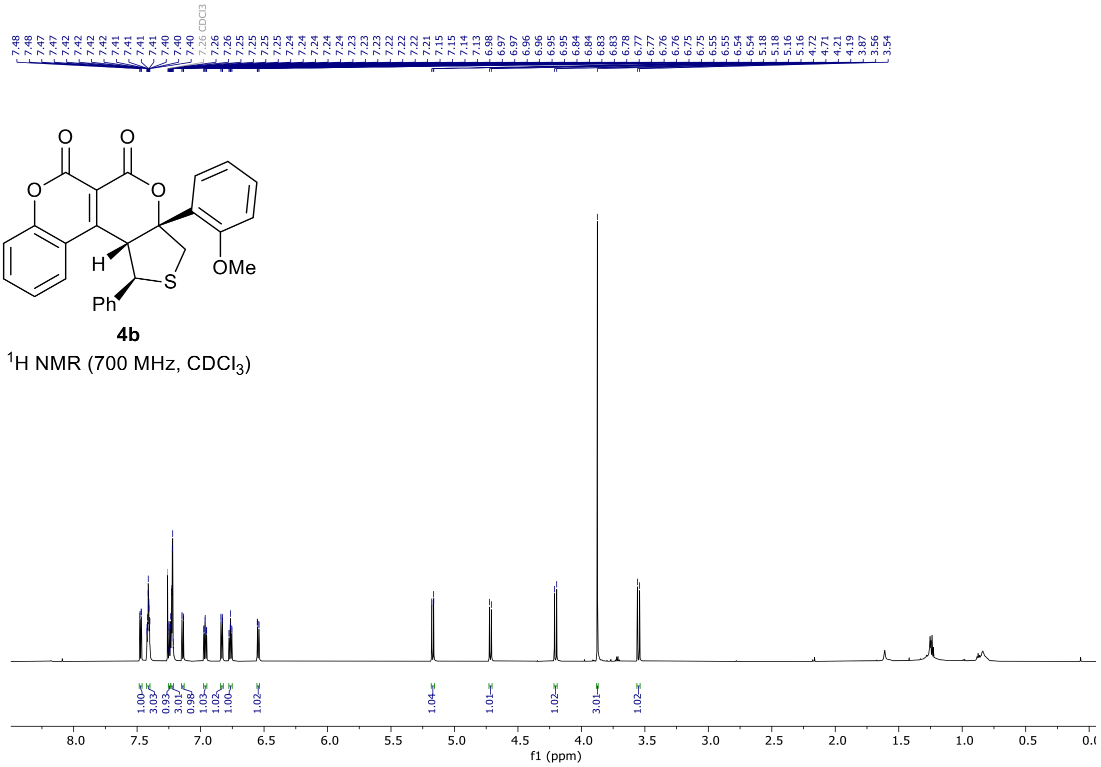

^{13}C NMR

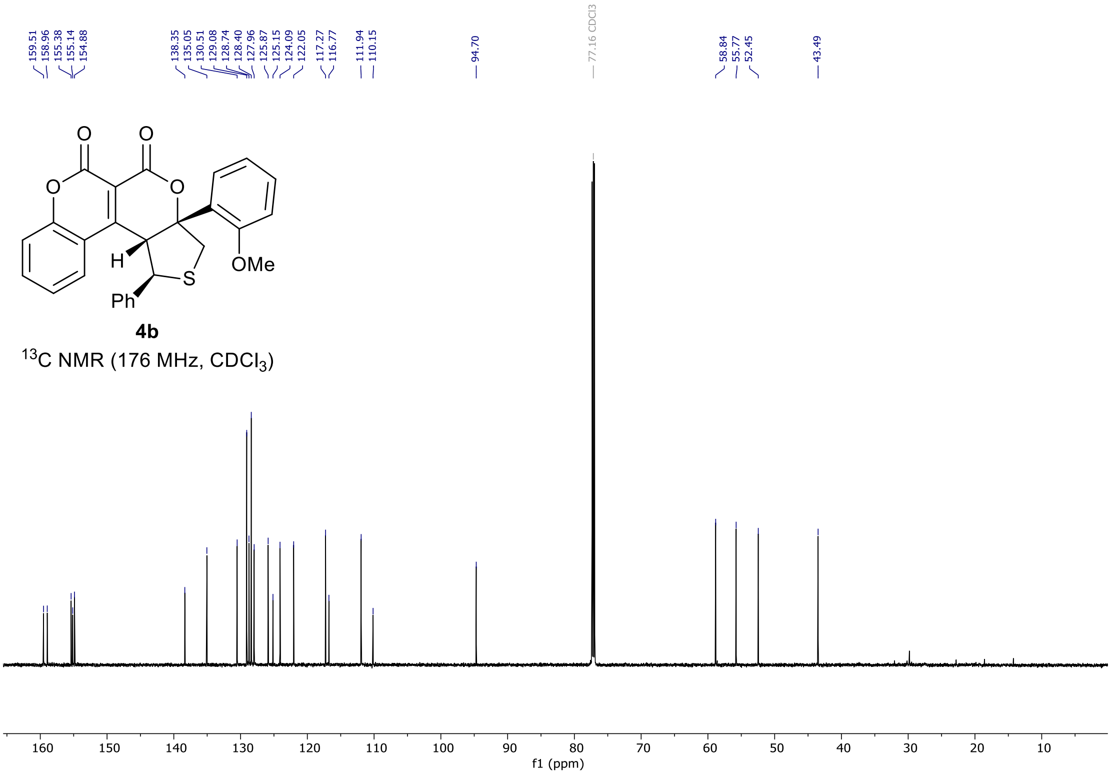

(1*R*,3a*R*,11c*R*)-3a-(3-Methoxyphenyl)-1-phenyl-1,3,3a,11c-tetrahydrothieno[3',4':5,6]pyrano[3,4-c]chromene-5,6-dione 4c

^{1}H NMR

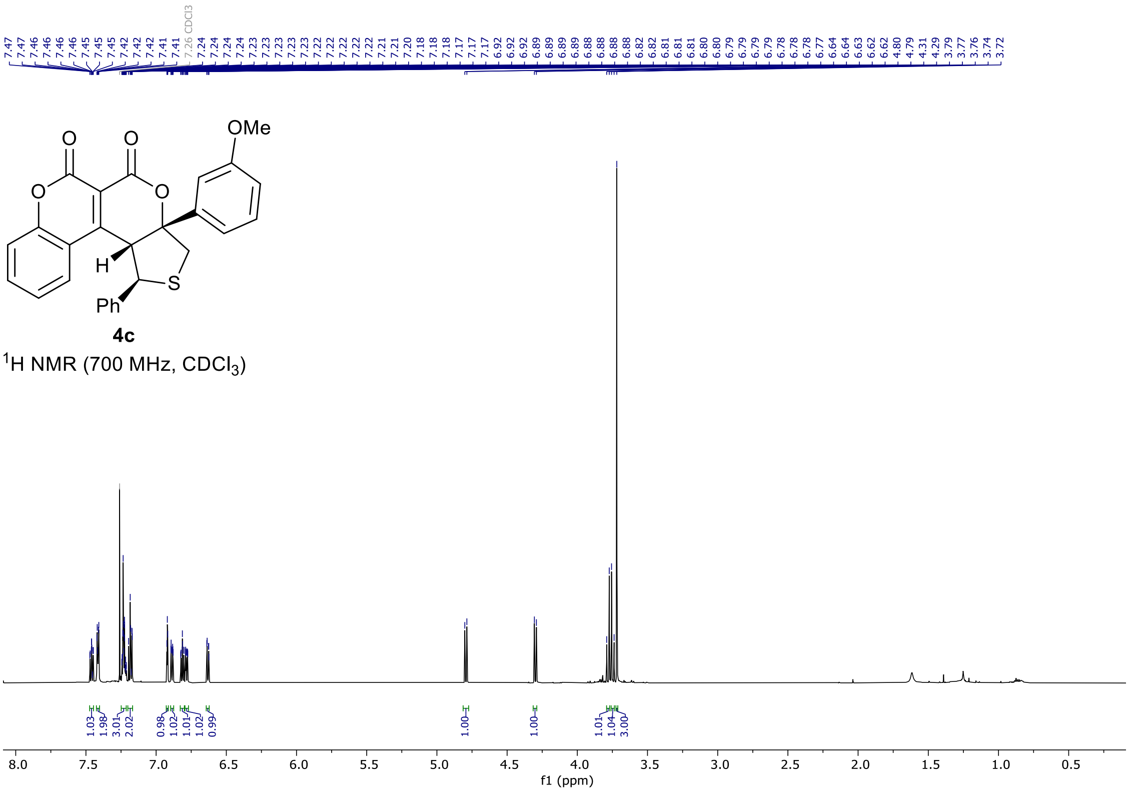

^{13}C NMR

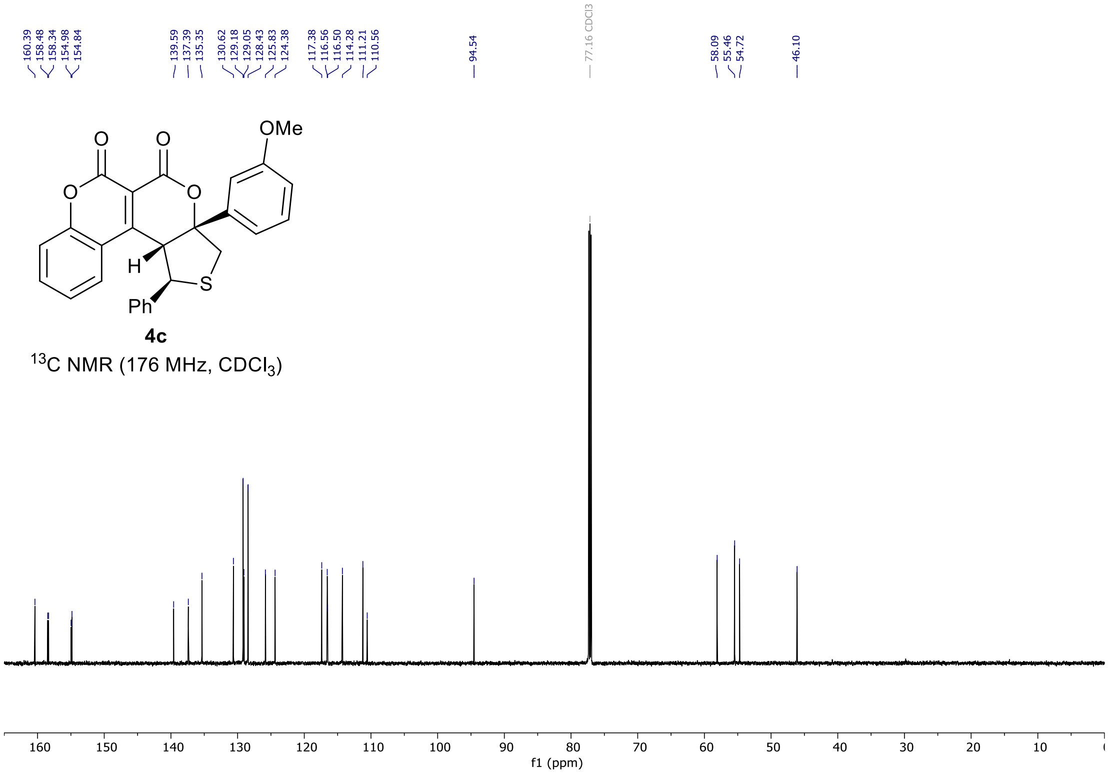

(1*R*,3a*R*,11c*R*)-3a-(2-Fluorophenyl)-1-phenyl-1,3,3a,11c-tetrahydrothieno[3',4':5,6]pyrano[3,4-c]chromene-5,6-dione 4d

^{1}H NMR

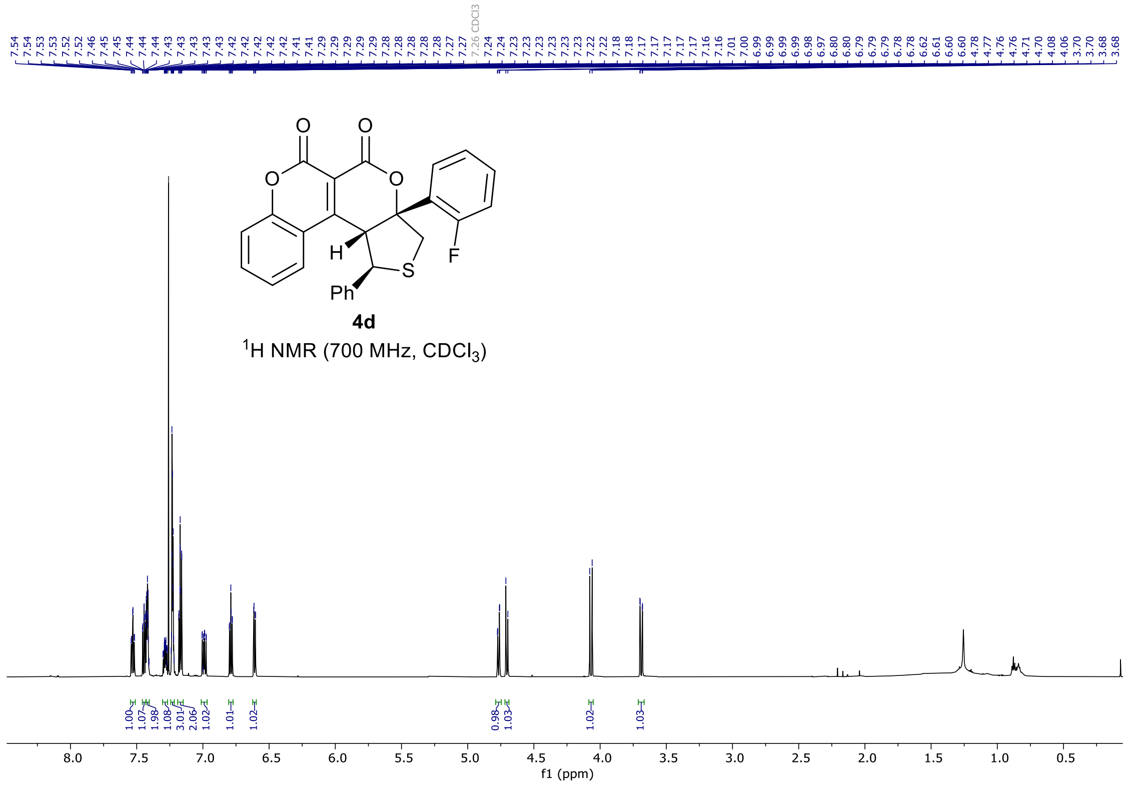

^{13}C NMR

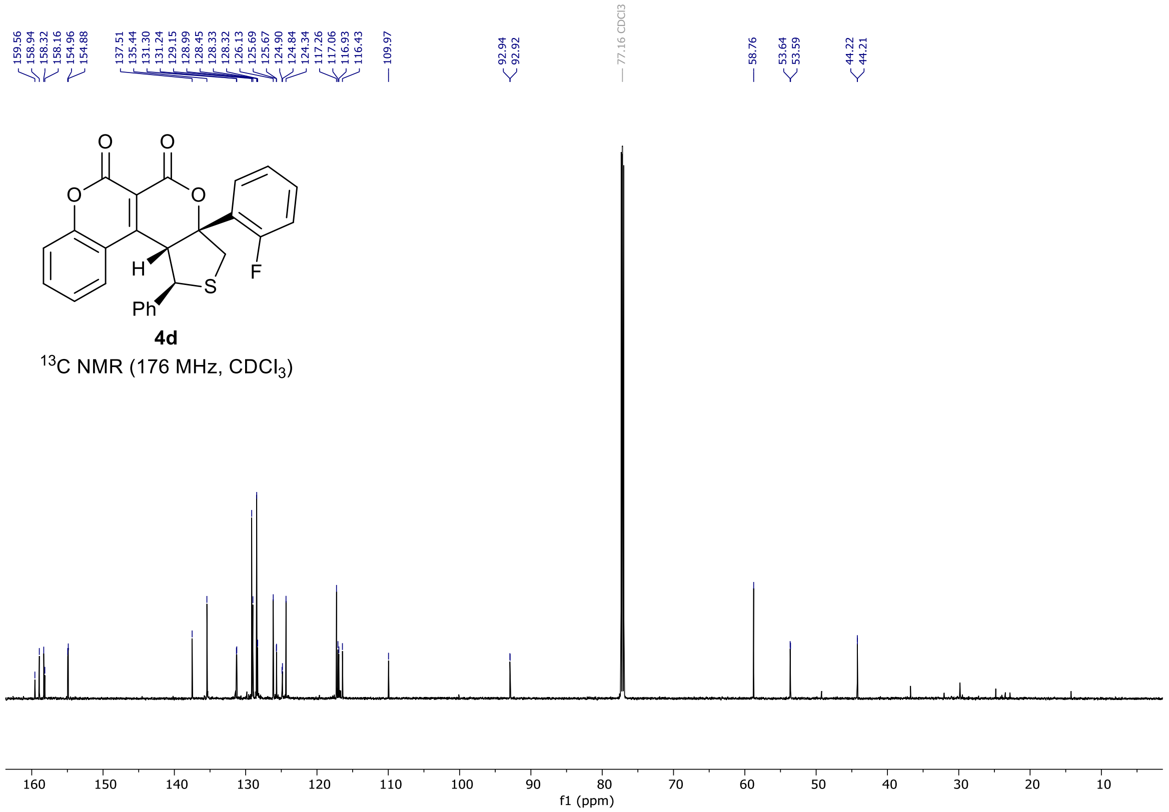

(1*R*,3a*R*,11c*R*)-1-Phenyl-3a-(4-(trifluoromethyl)phenyl)-1,3,3a,11c-tetrahydrothieno[3',4':5,6]pyrano[3,4-c]chromene-5,6-dione 4e

^{1}H NMR

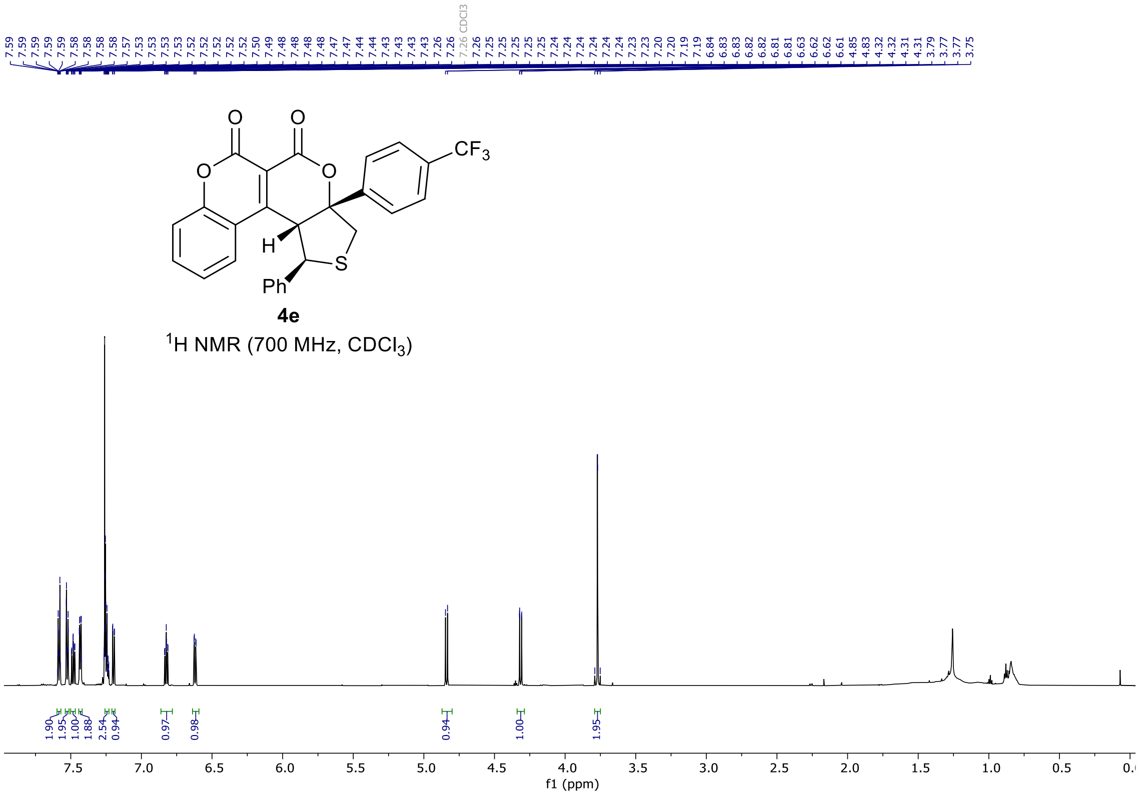

^{13}C NMR

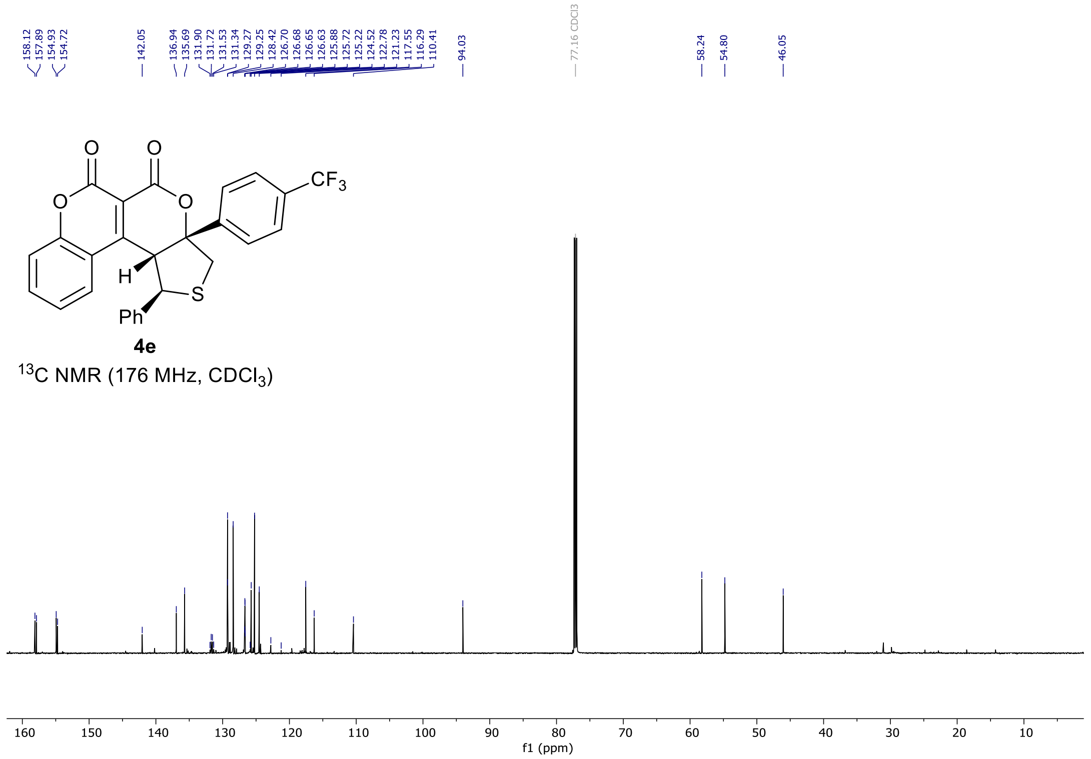

(1*R*,3a*R*,11c*R*)-3a-(Naphthalen-2-yl)-1-phenyl-1,3,3a,11c-tetrahydrothieno[3',4':5,6]pyrano[3,4-c]chromene-5,6-dione 4f

^{1}H NMR

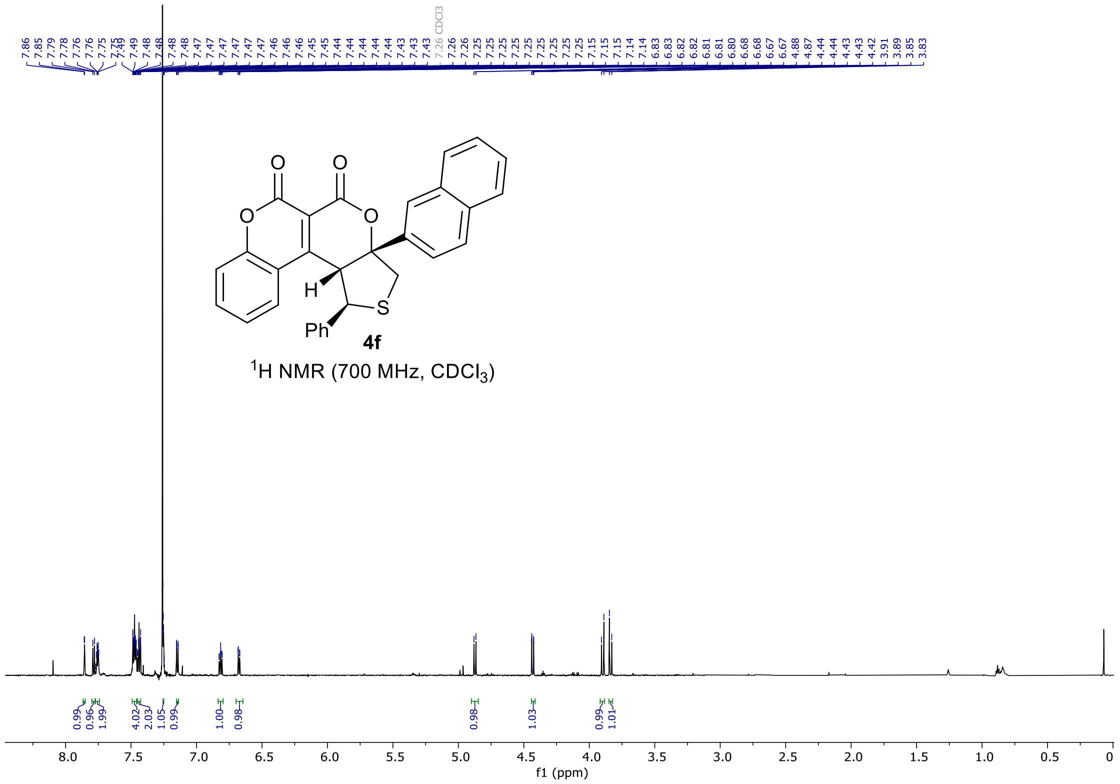

^{13}C NMR

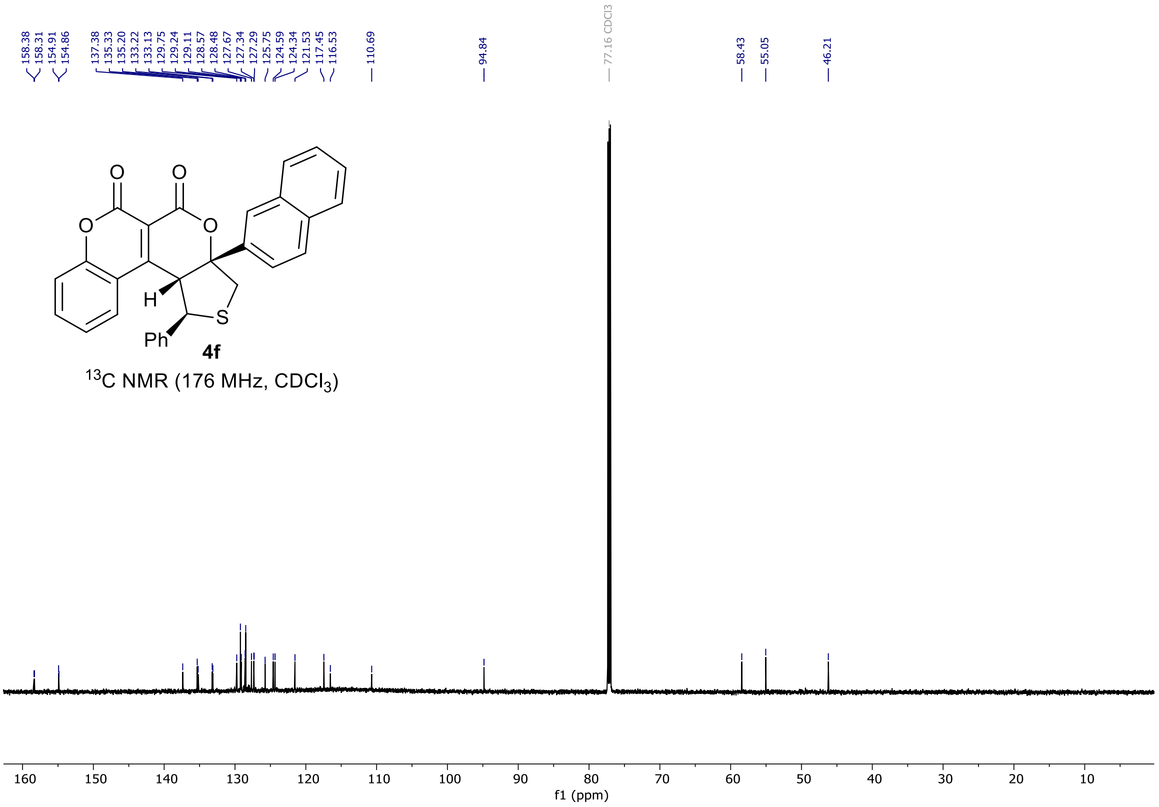

(1*R*,3a*R*,11c*R*)-1-(4-Methoxyphenyl)-3a-phenyl-1,3,3a,11c-tetrahydrothieno[3',4':5,6]pyrano[3,4-c]chromene-5,6-dione 4g

^{1}H NMR

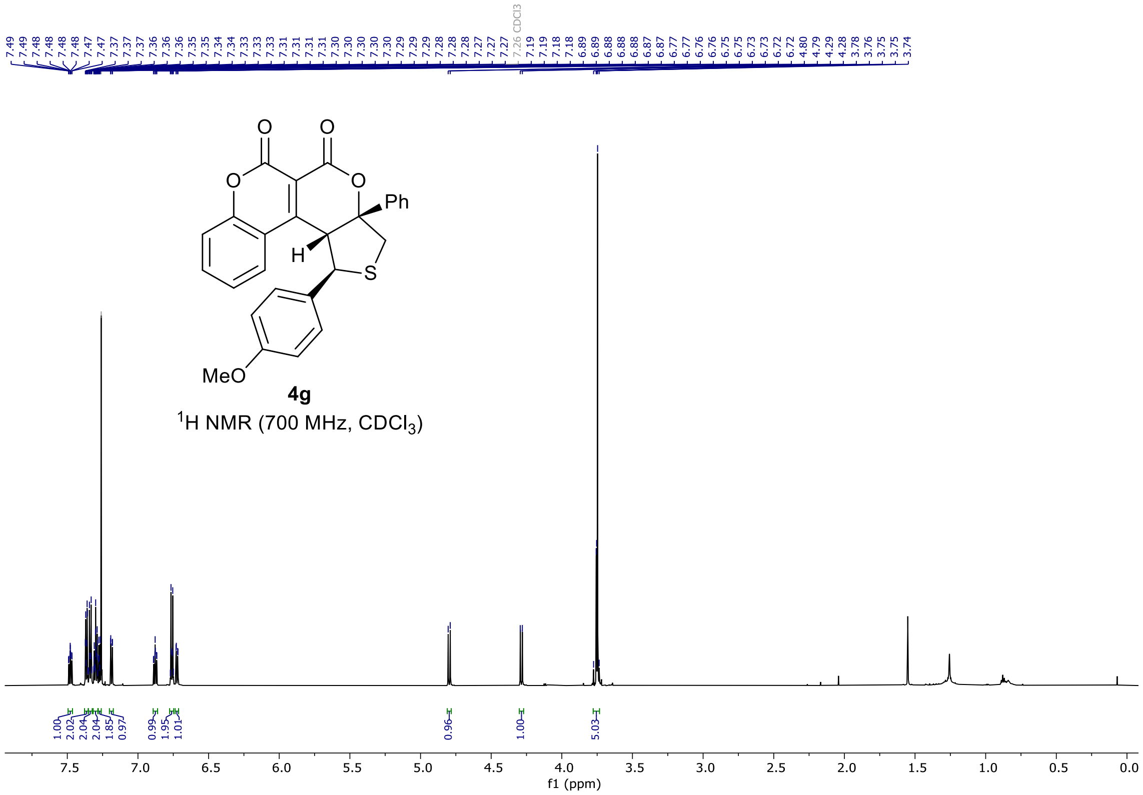

^{13}C NMR

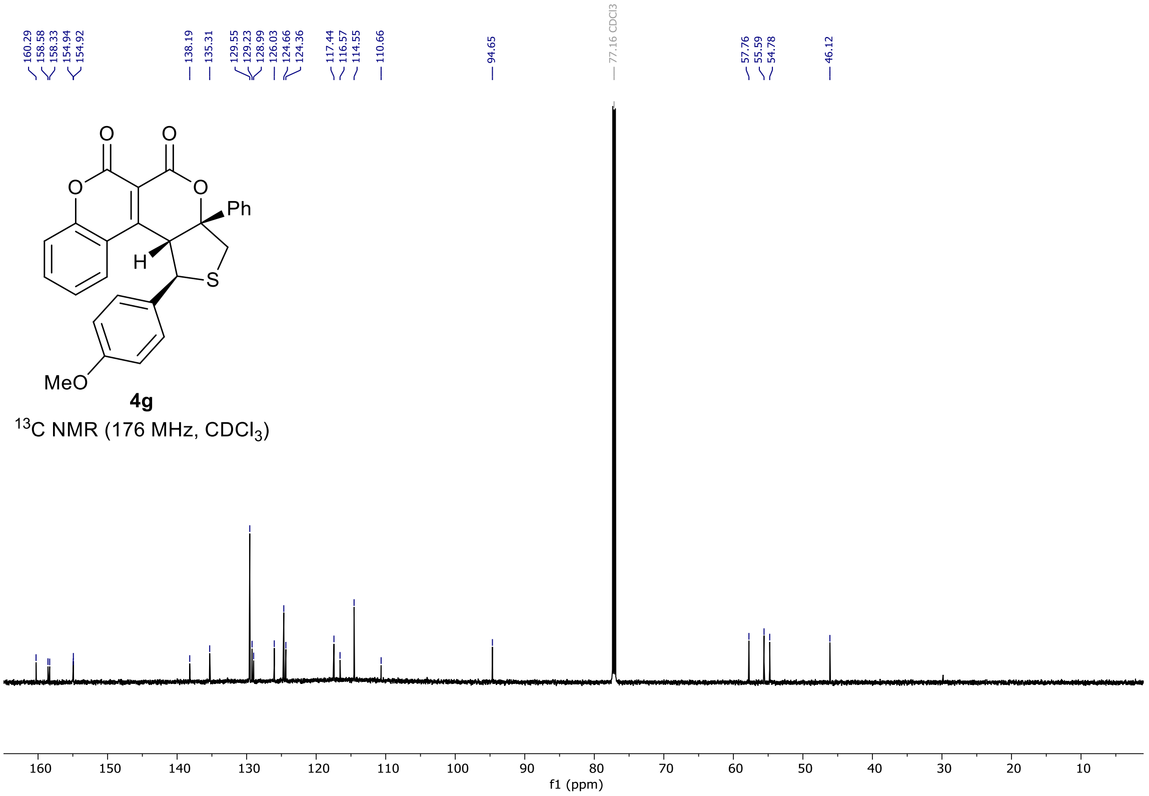

(1*R*,3a*R*,11c*R*)-3a-Phenyl-1-(*m*-tolyl)-1,3,3a,11c-tetrahydrothieno[3',4':5,6]pyrano[3,4-c]chromene-5,6-dione 4h

^{1}H NMR

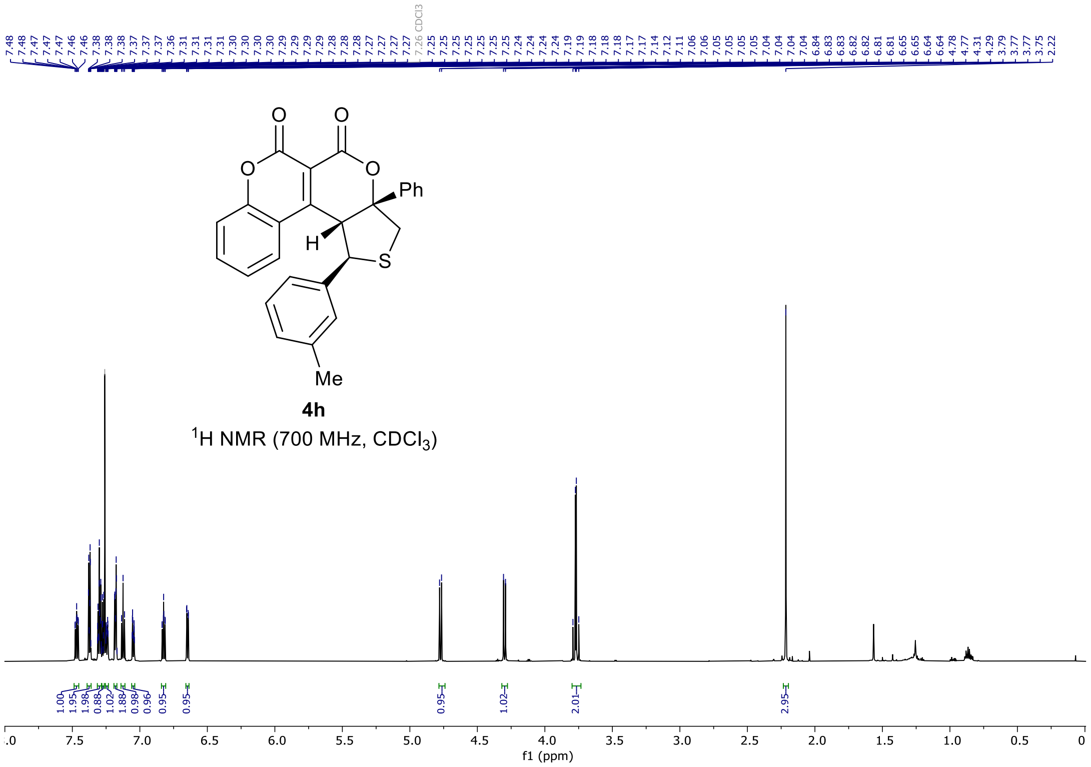

^{13}C NMR

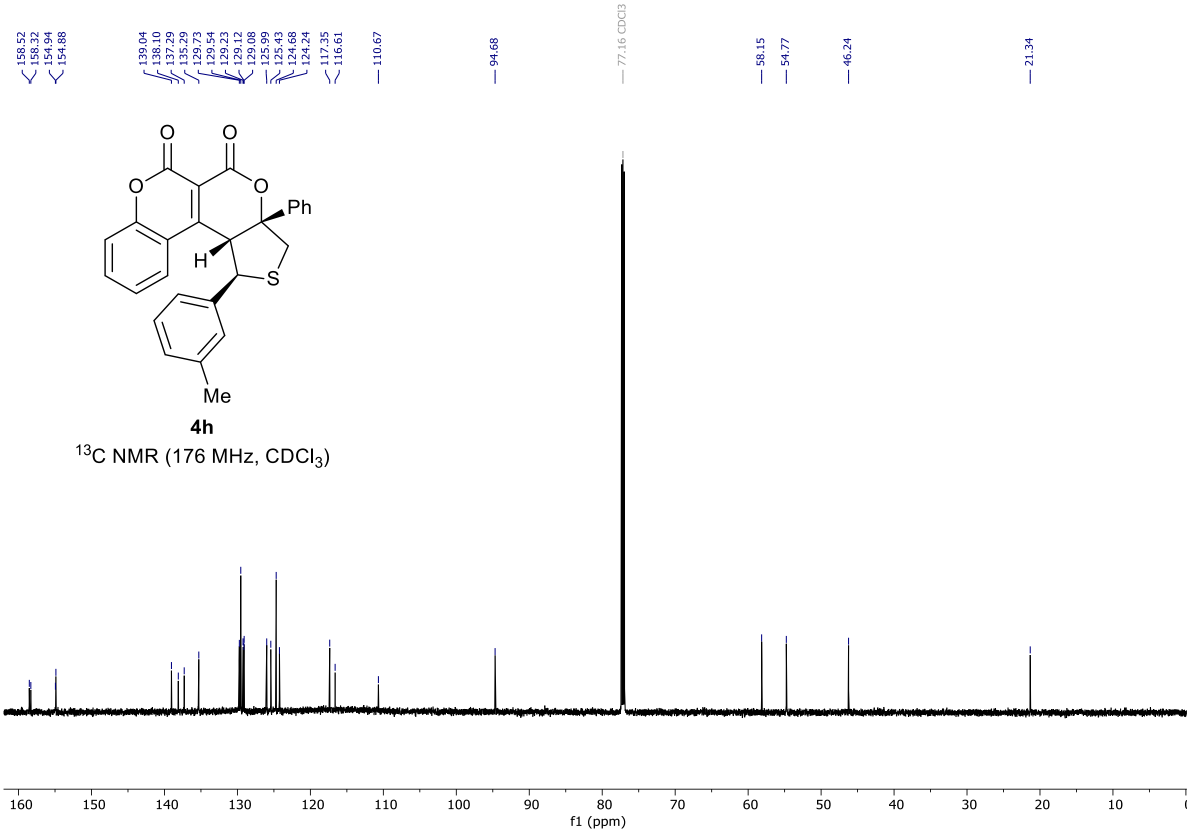

(*Z*)-*tert*-Butyl-((1*R*,3a*R*,11c*R*)-6-oxo-1,3a-diphenyl-1,3,3a,11c-tetrahydrothieno[3',4':5,6]pyrano[3,4-c]chromen-5(6*H*)-ylidene)carbamate 7

^{1}H NMR

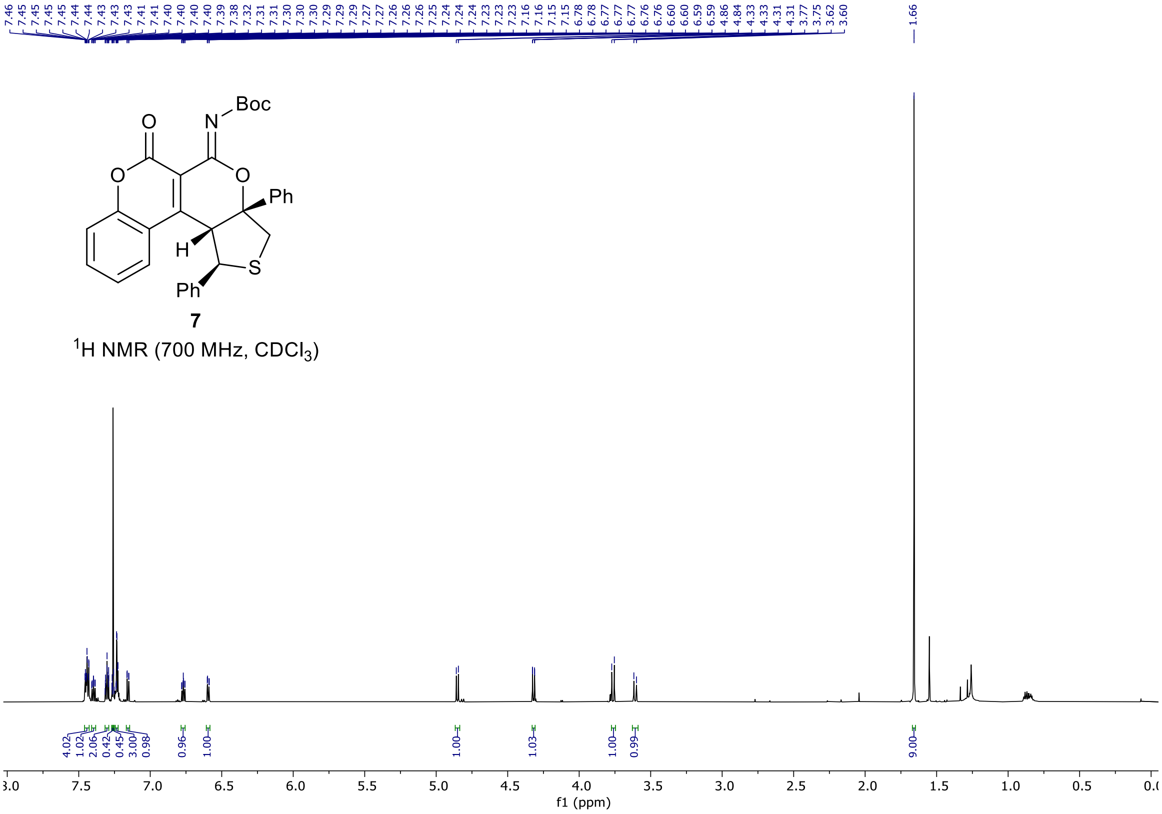

^{13}C NMR

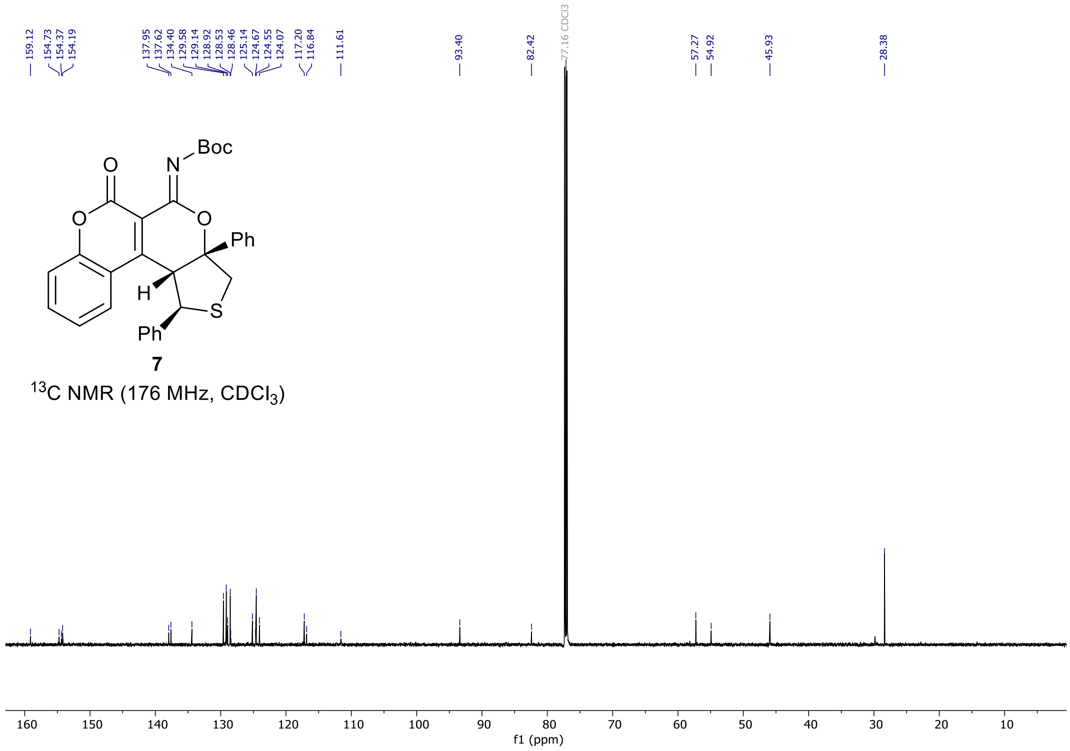

11. UPC^2 traces

(1*R*,3a*R*,11c*R*)-5-Imino-1,3a-diphenyl-1,3,3a,11c-tetrahydrothieno[3',4':5,6]pyrano[3,4-c]chromen-6(5*H*)-one 3a

Enantiomerically enriched sample

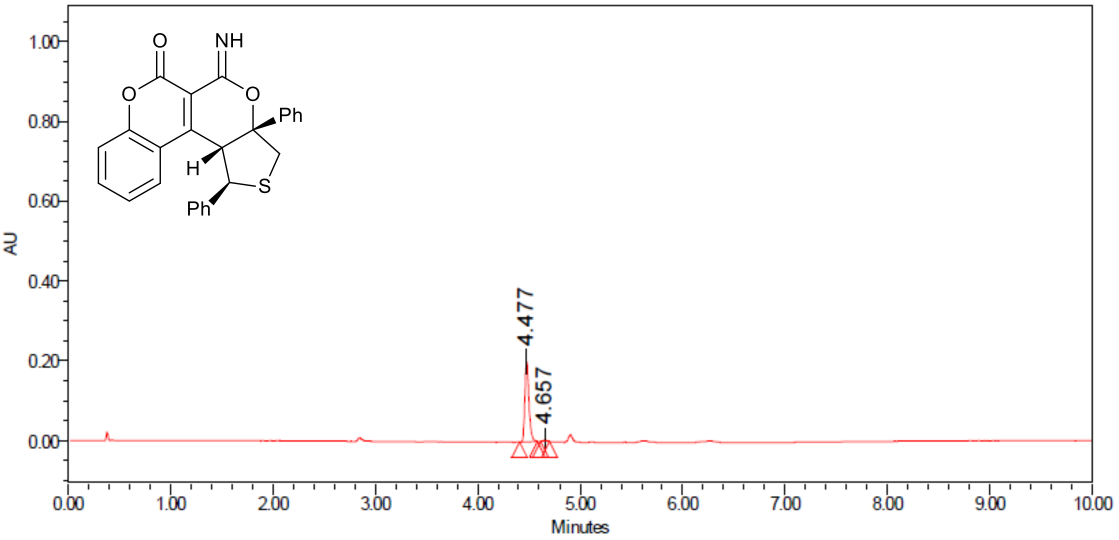

Peak Results

	RT	% Area
1	4.477	98.04
2	4.657	1.96

Racemic sample

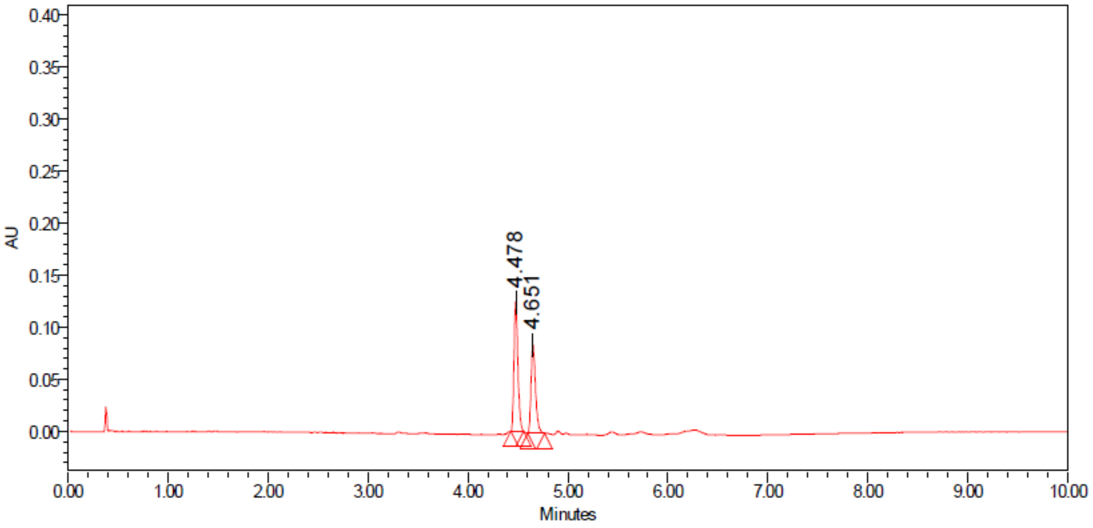

Peak Results

	RT	% Area
1	4.478	55.84
2	4.651	44.16

(1*R*,3a*R*,11c*R*)-5-Imino-3a-(2-methoxyphenyl)-1-phenyl-1,3,3a,11c-tetrahydrothieno[3',4':5,6]pyrano[3,4-c]chromen-6(5*H*)-one 3b

Enantiomerically enriched sample

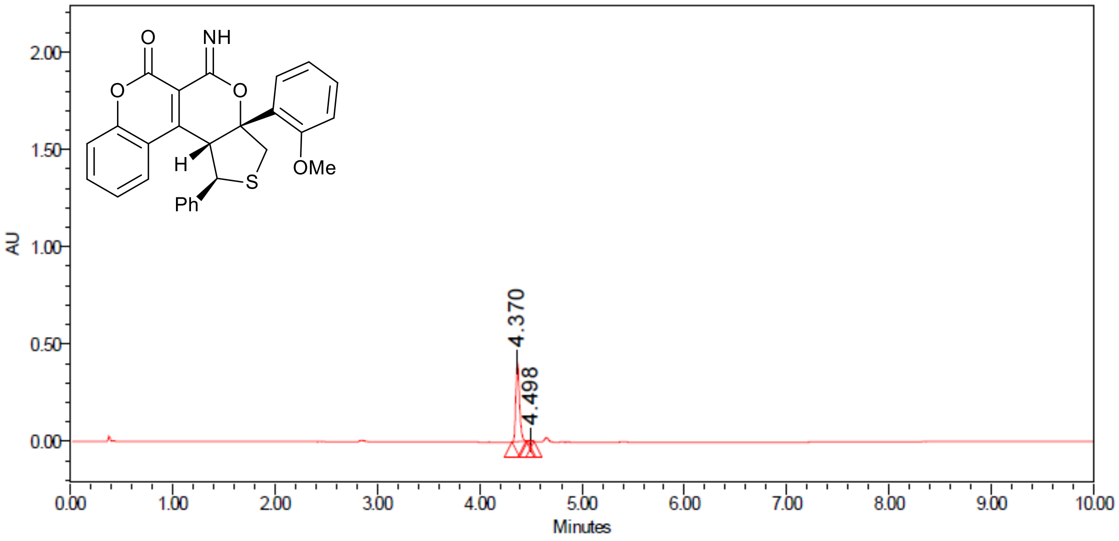

Peak Results

	RT	% Area
1	4.370	99.00
2	4.498	1.00

Racemic sample

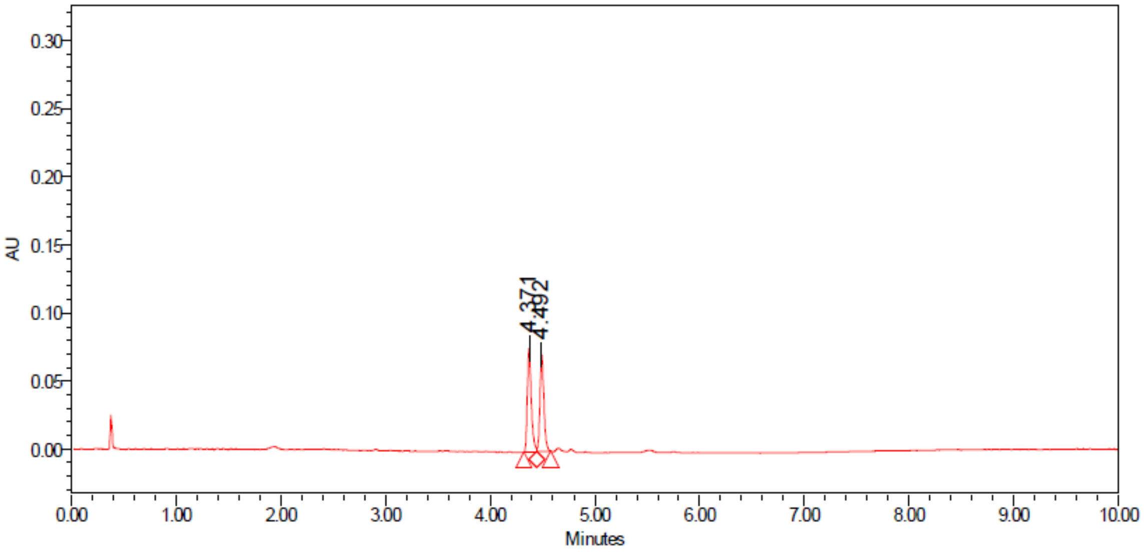

Peak Results

	RT	% Area
1	4.371	51.91
2	4.492	48.09

(1*R*,3a*R*,11c*R*)-5-Imino-3a-(3-methoxyphenyl)-1-phenyl-1,3,3a,11c-tetrahydrothieno[3',4':5,6]pyrano[3,4-c]chromen-6(5*H*)-one 3c

Enantiomerically enriched sample

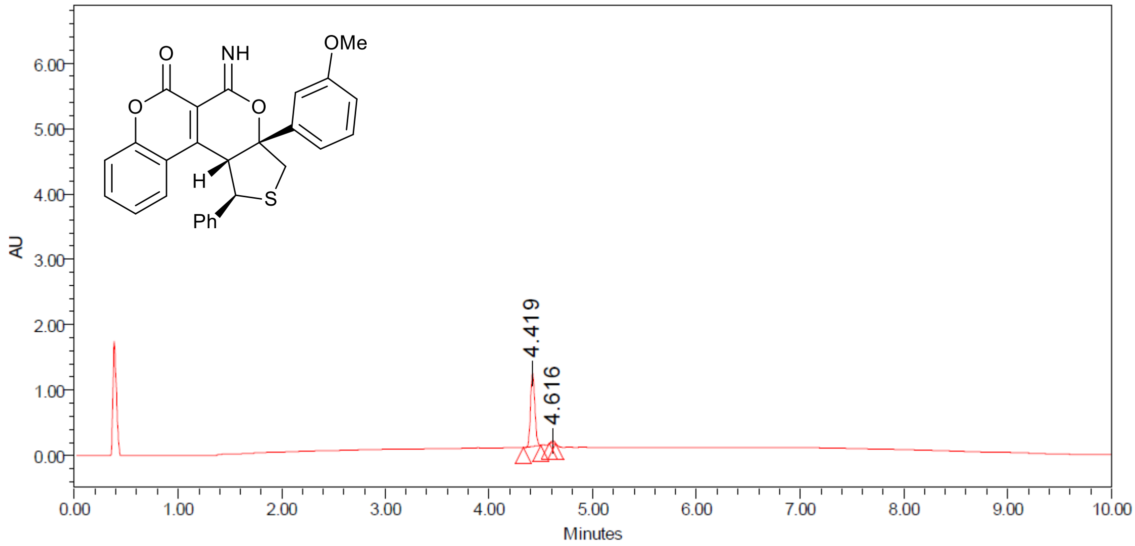

Peak Results

	RT	% Area
1	4.419	97.39
2	4.616	2.61

Racemic sample

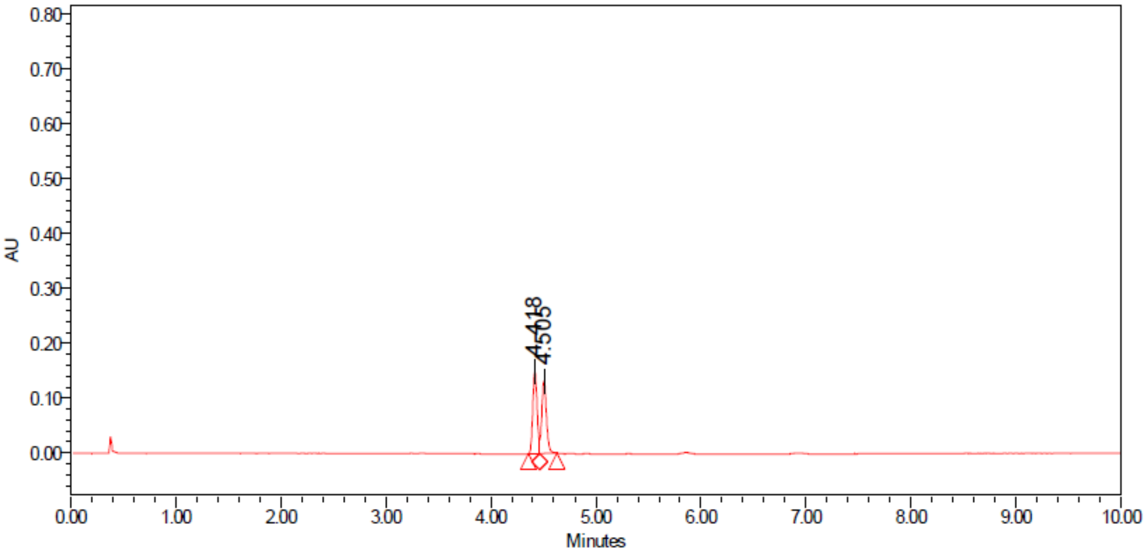

Peak R

	Name	RT	Area
1		4.418	425540
2		4.505	410021

(1*R*,3a*R*,11c*R*)-5-Imino-1-phenyl-3a-(*p*-tolyl)-1,3,3a,11c-tetrahydrothieno[3',4':5,6]pyrano[3,4-c]chromen-6(5*H*)-one 3d

Enantiomerically enriched sample

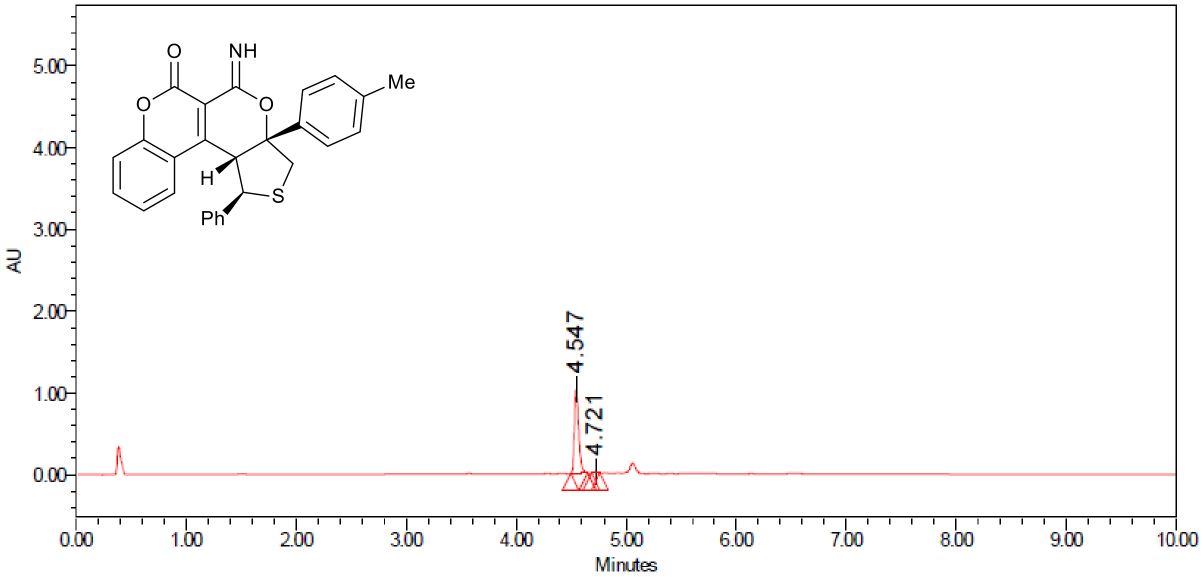

Peak Results

	RT	% Area
1	4.547	98.84
2	4.721	1.16

Racemic sample

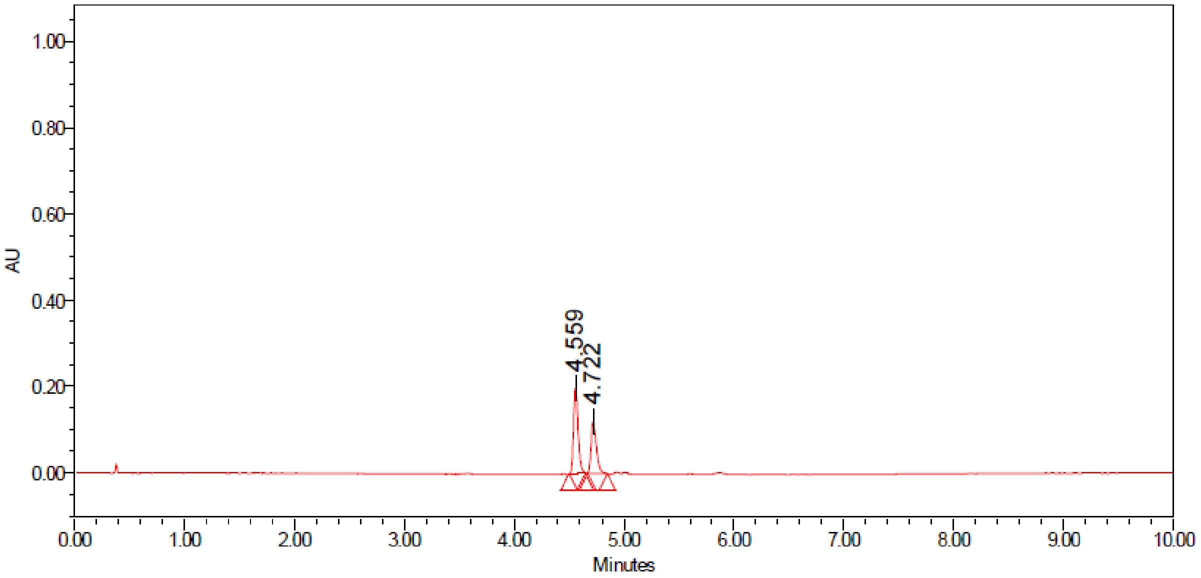

Peak Results

	RT	% Area
1	4.559	58.57
2	4.722	41.43

(1*R*,3a*R*,11c*R*)-3a-(2-Fluorophenyl)-5-imino-1-phenyl-1,3,3a,11c-tetrahydrothieno[3',4':5,6]pyrano[3,4-c]chromen-6(5*H*)-one 3e

Enantiomerically enriched sample

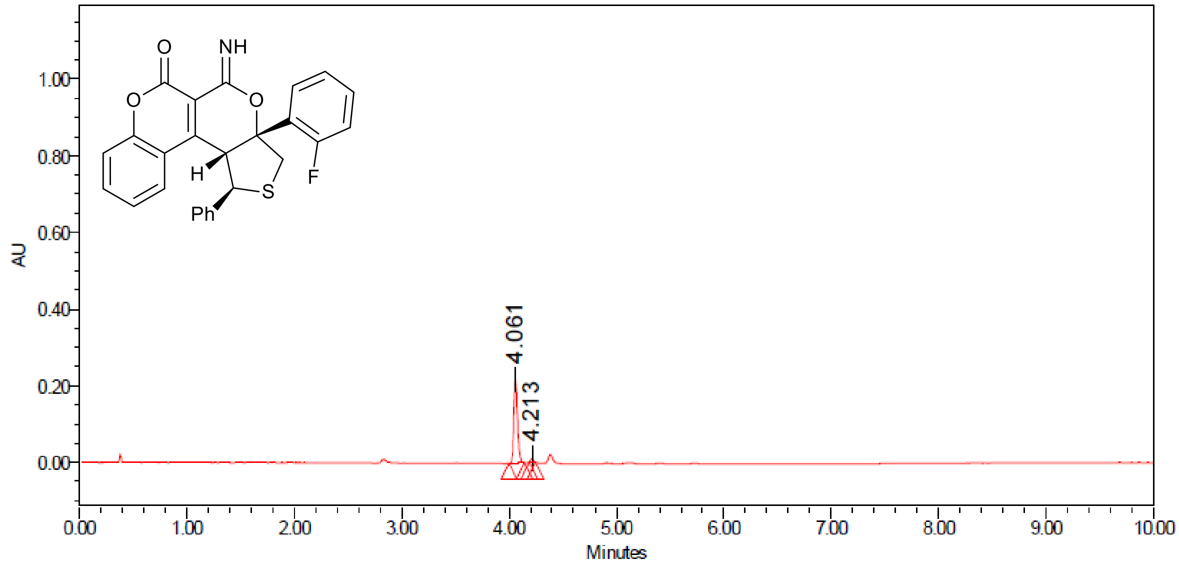

Peak Results

	RT	% Area
1	4.061	96.79
2	4.213	3.21

Racemic sample

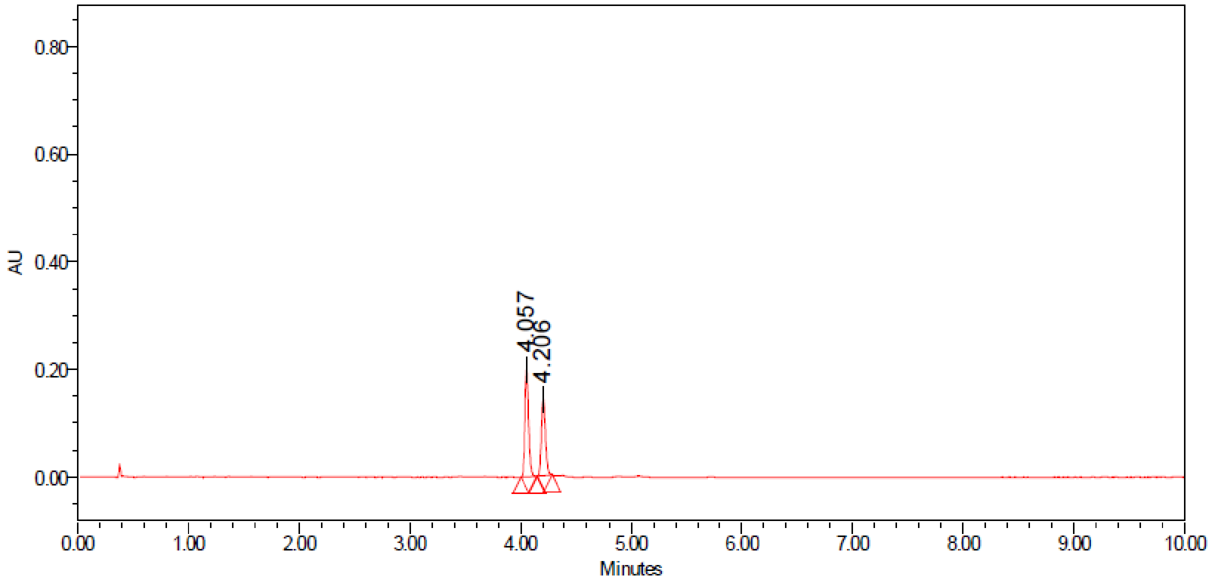

Peak Results

	RT	% Area
1	4.057	56.95
2	4.206	43.05

(1*R*,3a*R*,11c*R*)-3a-(4-Fluorophenyl)-5-imino-1-phenyl-1,3,3a,11c-tetrahydrothieno[3',4':5,6]pyrano[3,4-c]chromen-6(5*H*)-one 3f

Enantiomerically enriched sample

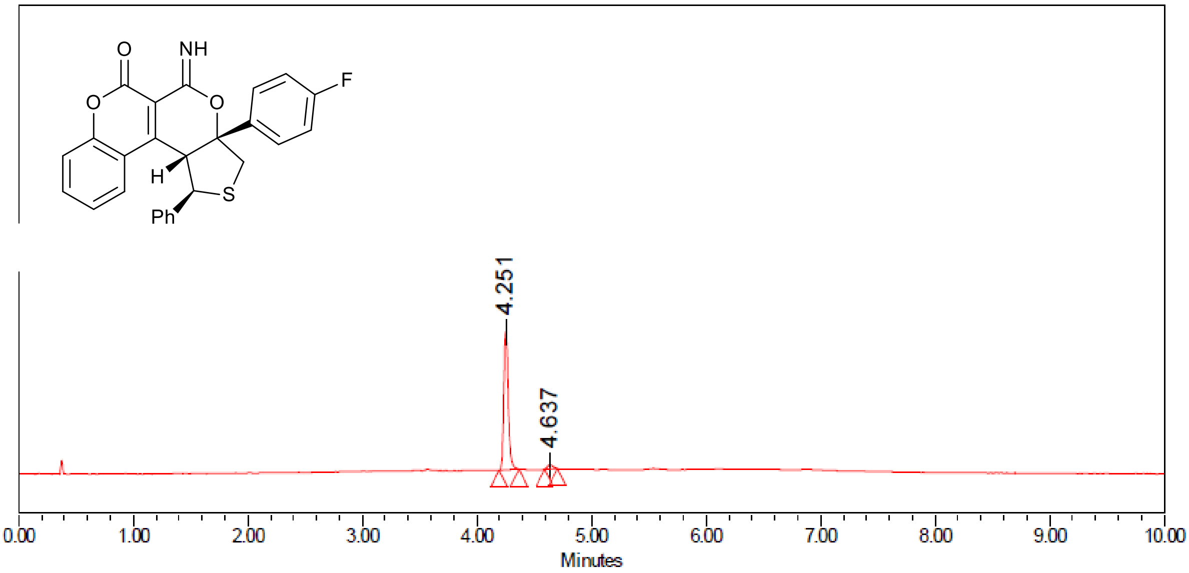

Peak Results

	RT	% Area
1	4.251	96.47
2	4.637	3.53

Racemic sample

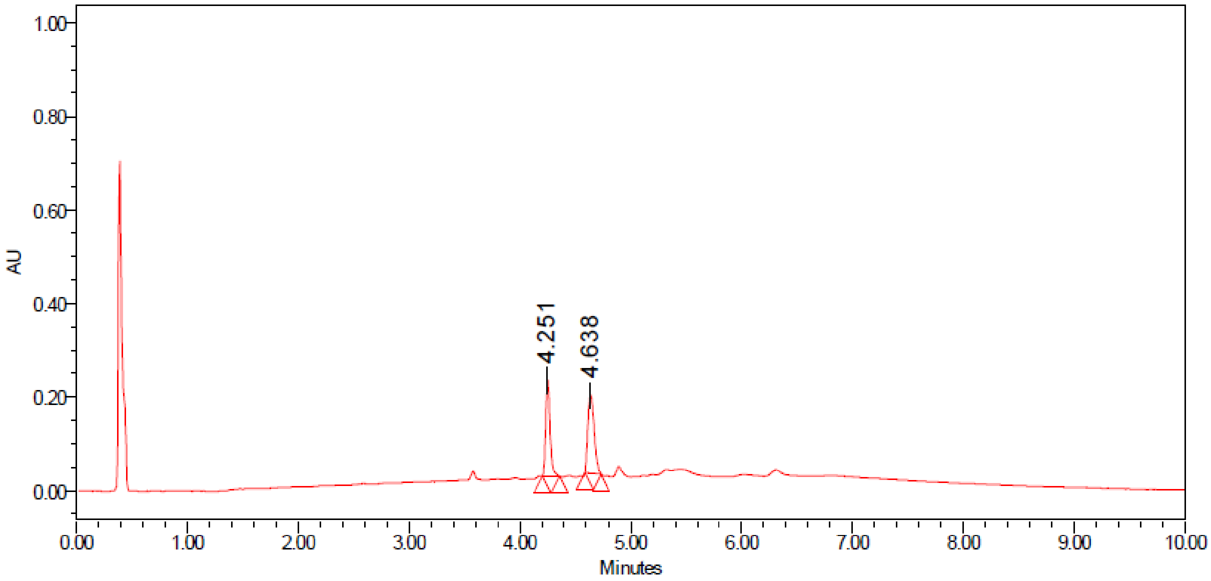

Peak Results

	RT	% Area
1	4.251	45.62
2	4.638	54.38

(1*R*,3a*R*,11c*R*)-5-Imino-1-phenyl-3a-(4-(trifluoromethyl)phenyl)-1,3,3a,11c-tetrahydrothieno[3',4':5,6]pyrano[3,4-c]chromen-6(5*H*)-one 3g

Enantiomerically enriched sample

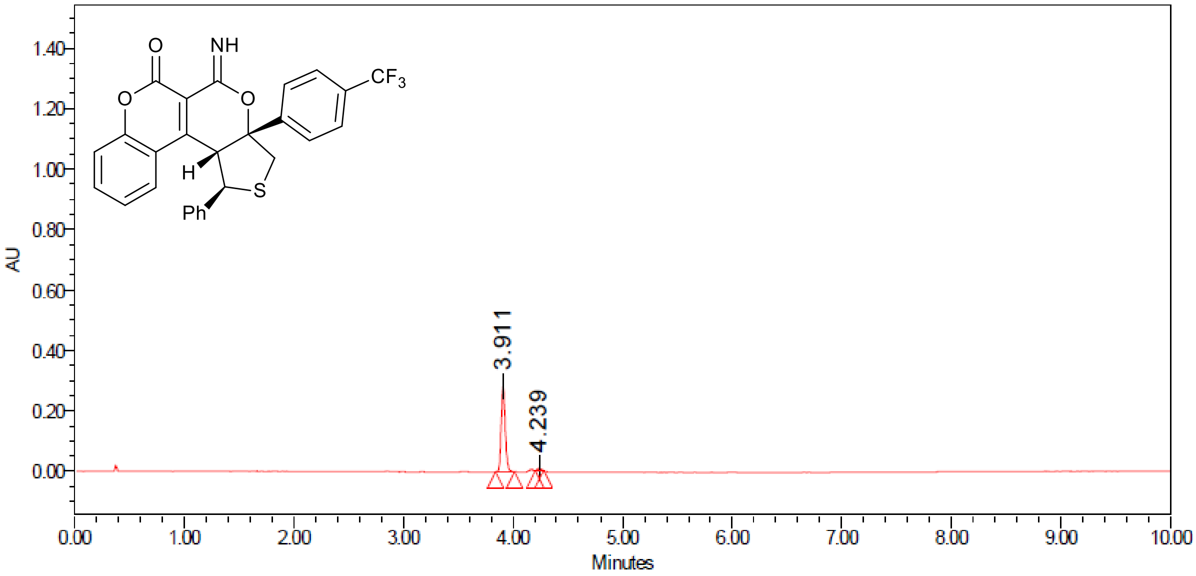

Peak Results

	RT	% Area
1	3.911	97.05
2	4.239	2.95

Racemic sample

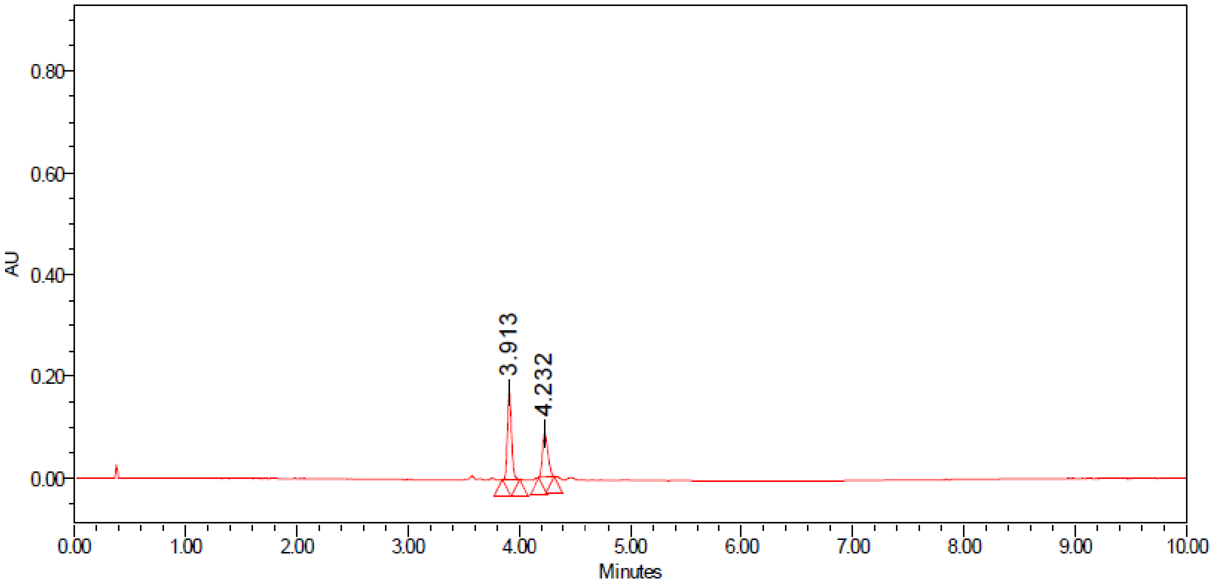

Peak Results

	RT	% Area
1	3.913	59.91
2	4.232	40.09

(1*R*,3a*R*,11c*R*)-5-Imino-3a-(naphthalen-2-yl)-1-phenyl-1,3,3a,11c-tetrahydrothieno[3',4':5,6]pyrano[3,4-c]chromen-6(5*H*)-one 3h

Enantiomerically enriched sample

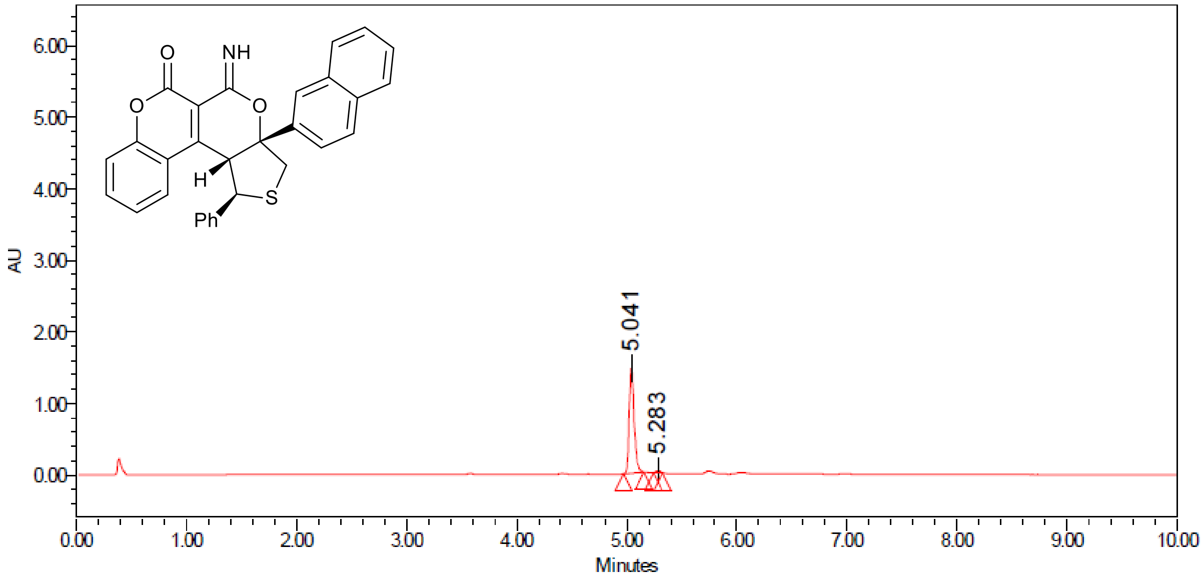

Peak Results

	RT	% Area
1	5.041	98.34
2	5.283	1.66

Racemic sample

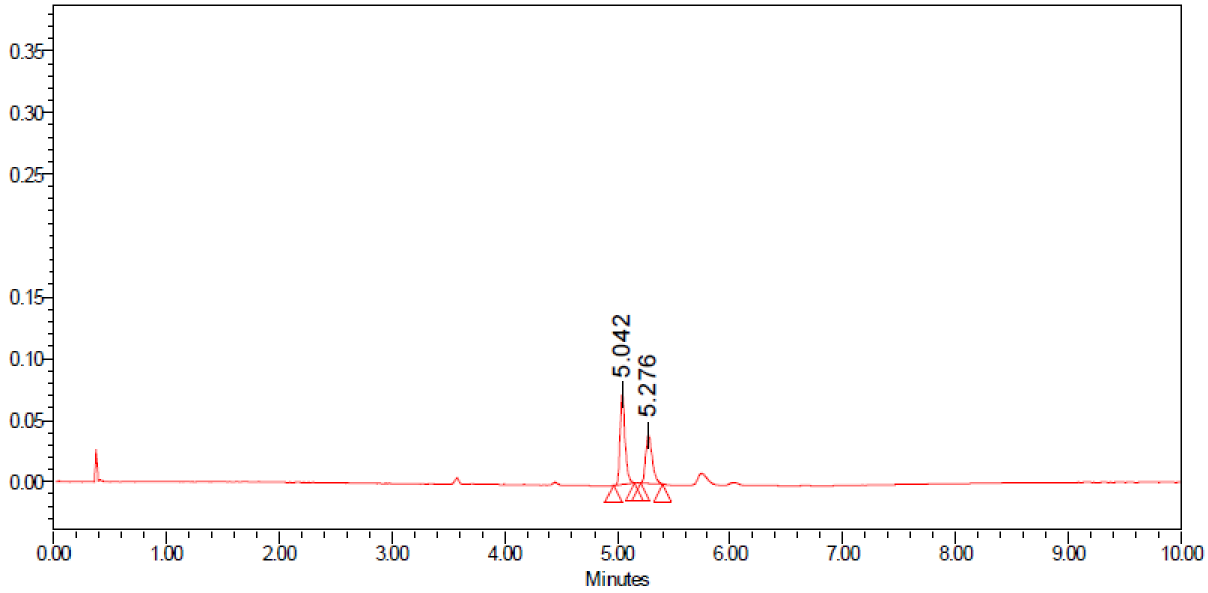

Peak Results

	RT	% Area
1	5.042	58.75
2	5.276	41.25

(1*R*,3a*R*,11c*R*)-5-Imino-1-(4-methoxyphenyl)-3a-phenyl-1,3,3a,11c-tetrahydrothieno[3',4':5,6]pyrano[3,4-c]chromen-6(5*H*)-one 3i

Enantiomerically enriched sample

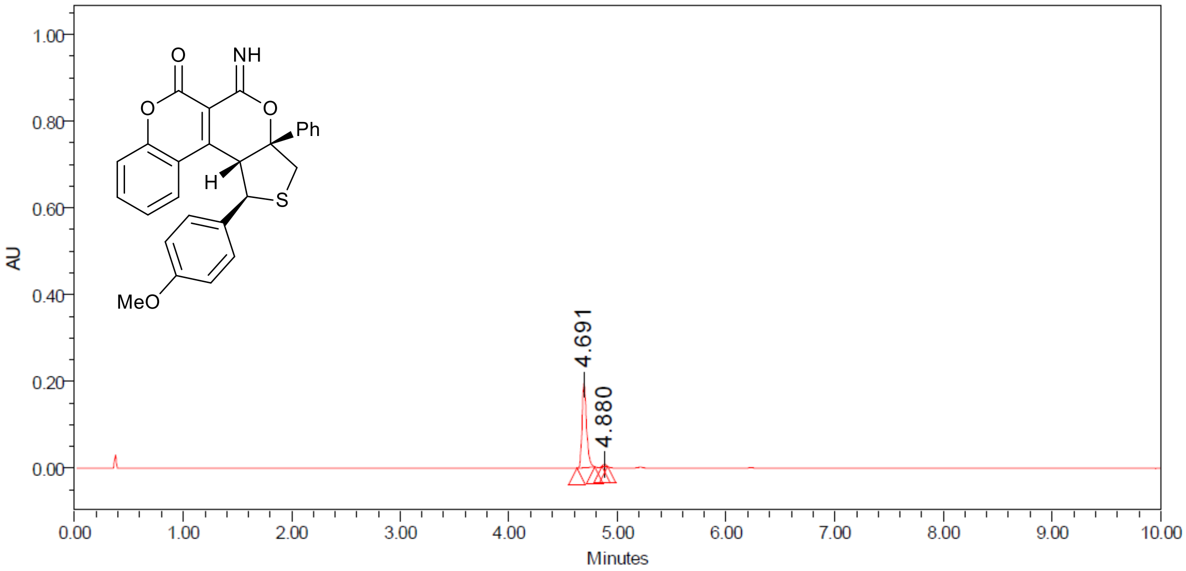

Peak Results

	RT	% Area
1	4.691	98.55
2	4.880	1.45

Racemic sample

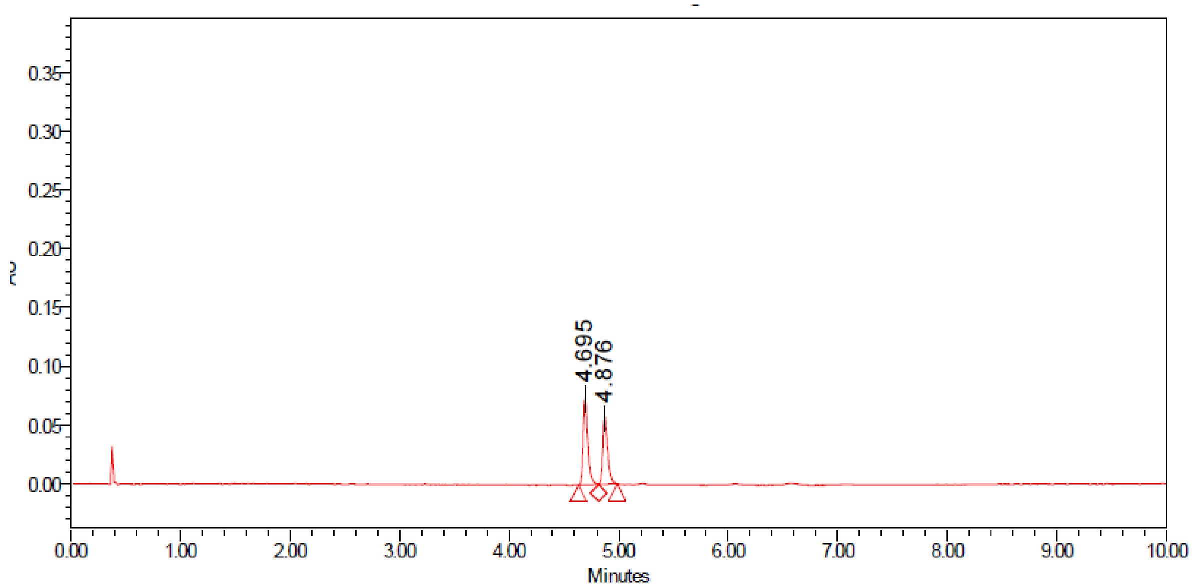

Peak Results

	RT	% Area
1	4.695	55.57
2	4.876	44.43

(1*R*,3a*R*,11c*R*)-5-Imino-3a-phenyl-1-(*m*-tolyl)-1,3,3a,11c-tetrahydrothieno[3',4':5,6]pyrano[3,4-c]chromen-6(5*H*)-one 3j

Enantiomerically enriched sample

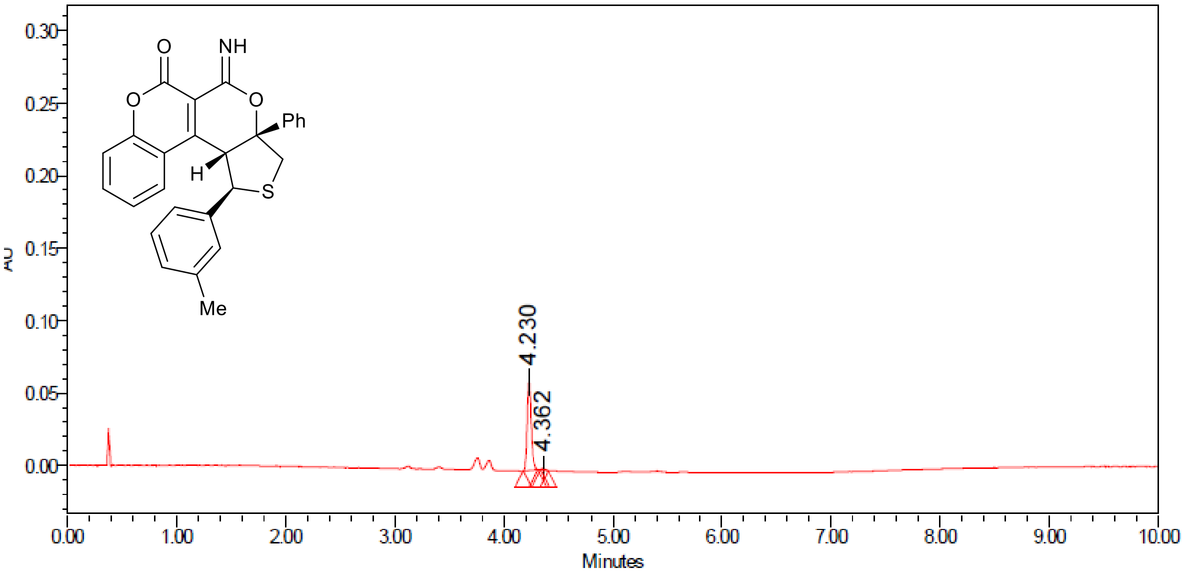

Peak Results

	RT	% Area
1	4.230	98.51
2	4.362	1.49

Racemic sample

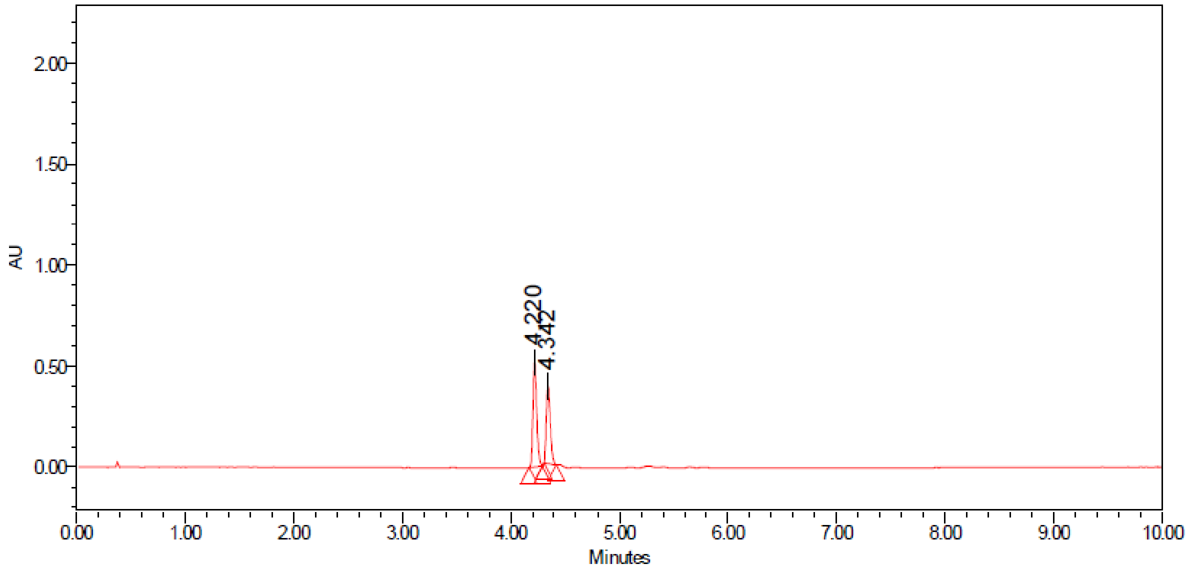

Peak Results

	RT	% Area
1	4.220	56.65
2	4.342	43.35

(1*R*,3a*R*,11c*R*)-5-Imino-3a-phenyl-1-(*p*-tolyl)-1,3,3a,11c-tetrahydrothieno[3',4':5,6]pyrano[3,4-c]chromen-6(5*H*)-one 3k

Enantiomerically enriched sample

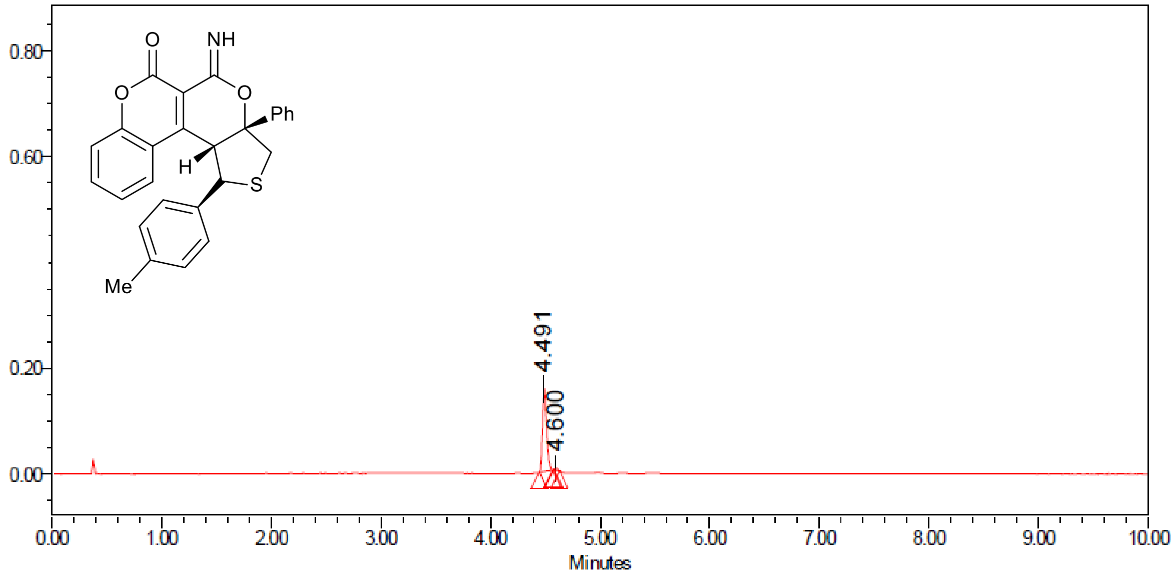

Peak Results

	RT	% Area
1	4.491	98.35
2	4.600	1.65

Racemic sample

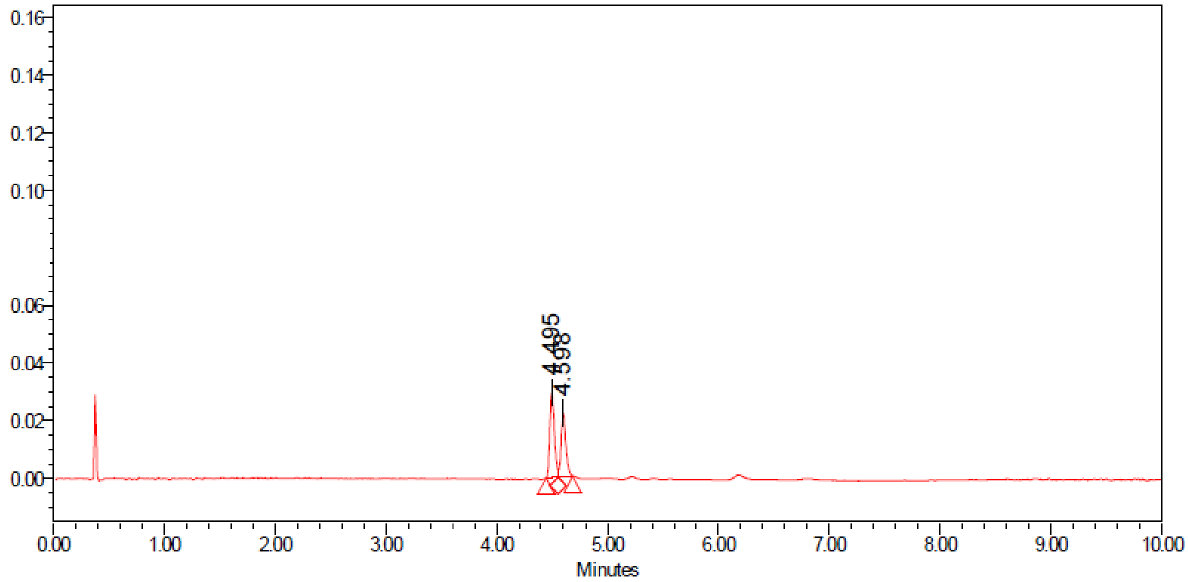

Peak Results

	RT	% Area
1	4.495	54.79
2	4.598	45.21

(1*R*,3a*R*,11c*R*)-1-(3-Chlorophenyl)-5-imino-3a-phenyl-1,3,3a,11c-tetrahydrothieno[3',4':5,6]pyrano[3,4-c]chromen-6(5*H*)-one 3l

Enantiomerically enriched sample

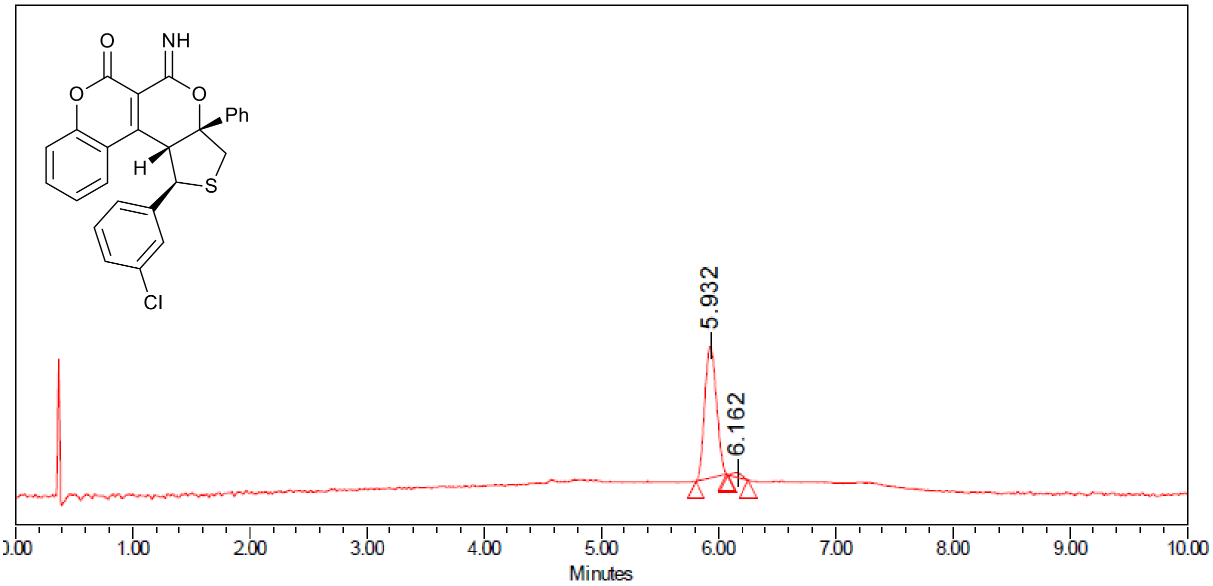

Peak Results

	RT	% Area
1	5.932	97.20
2	6.162	2.80

Racemic sample

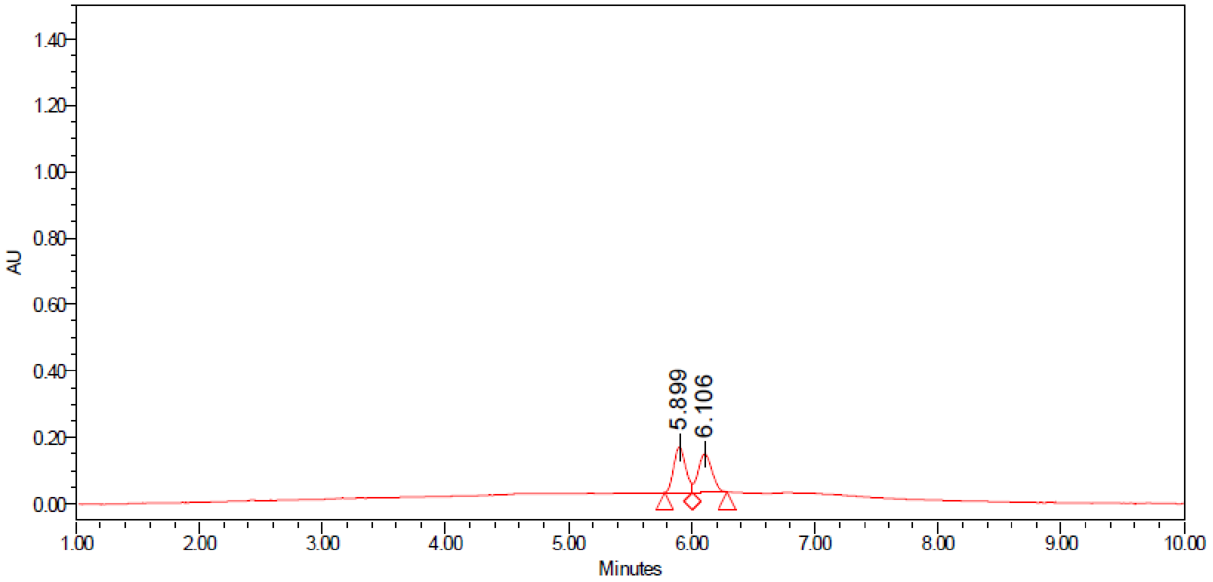

Peak Results

	RT	% Area
1	5.899	51.51
2	6.106	48.49

(1*R*,3a*R*,11c*R*)-1-(4-Chlorophenyl)-5-imino-3a-phenyl-1,3,3a,11c-tetrahydrothieno[3',4':5,6]pyrano[3,4-c]chromen-6(5*H*)-one 3m

Enantiomerically enriched sample

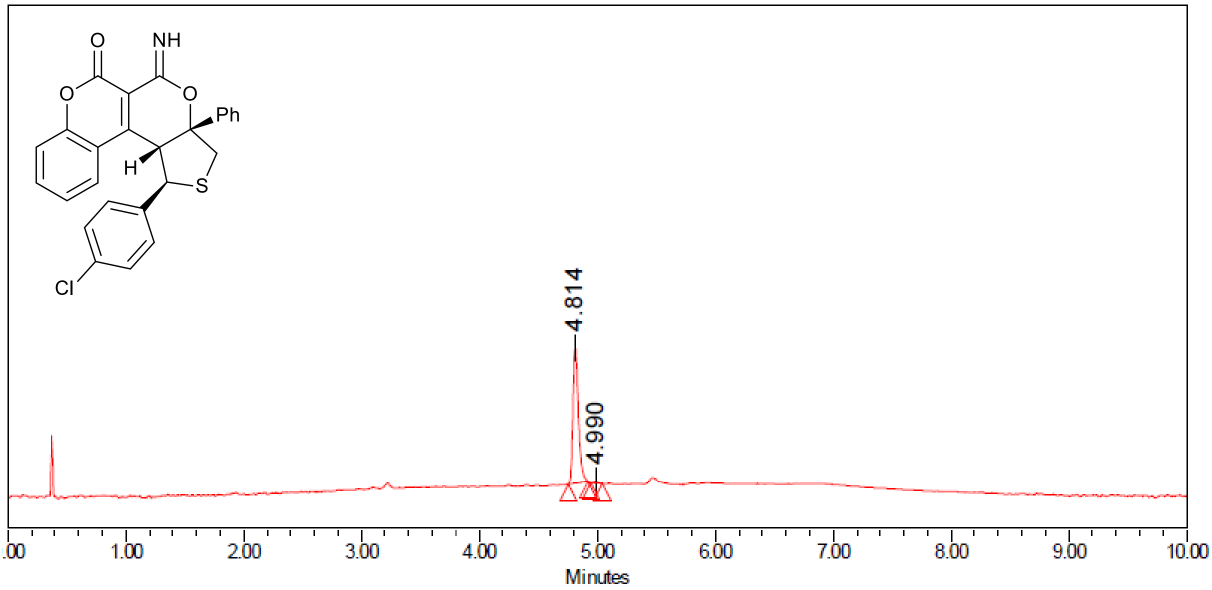

Peak Results

	RT	% Area
1	4.814	99.29
2	4.990	0.71

Racemic sample

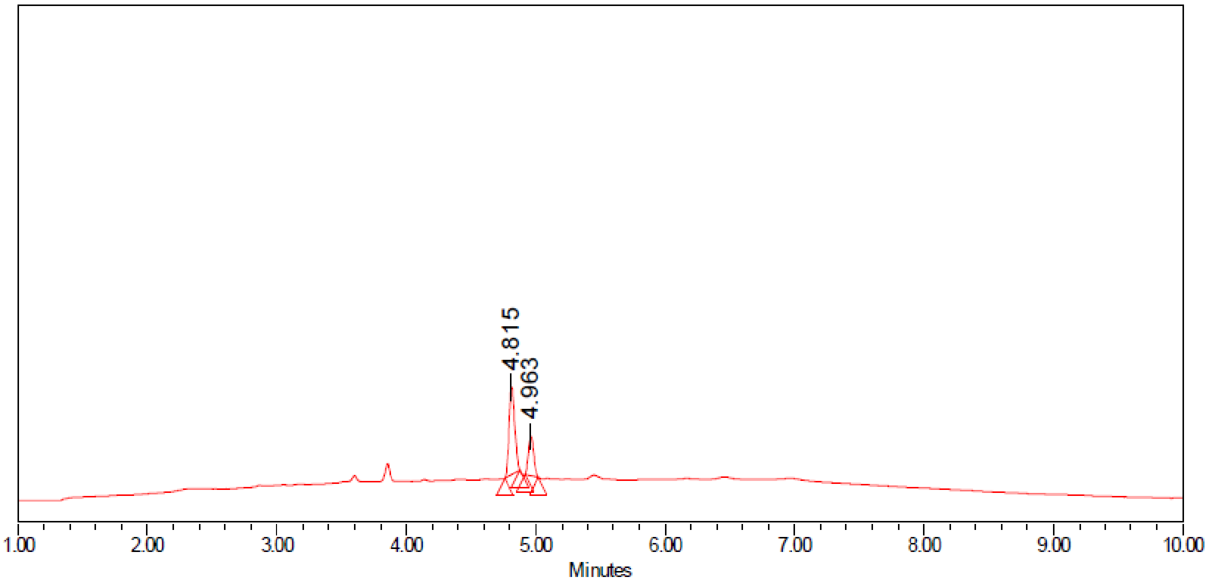

Peak Results

	RT	% Area
1	4.815	70.25
2	4.963	29.75

(1*R*,3a*R*,11c*R*)-5-Imino-1-(4-nitrophenyl)-3a-phenyl-1,3,3a,11c-tetrahydrothieno[3',4':5,6]pyrano[3,4-c]chromen-6(5*H*)-one 3n

Enantiomerically enriched sample

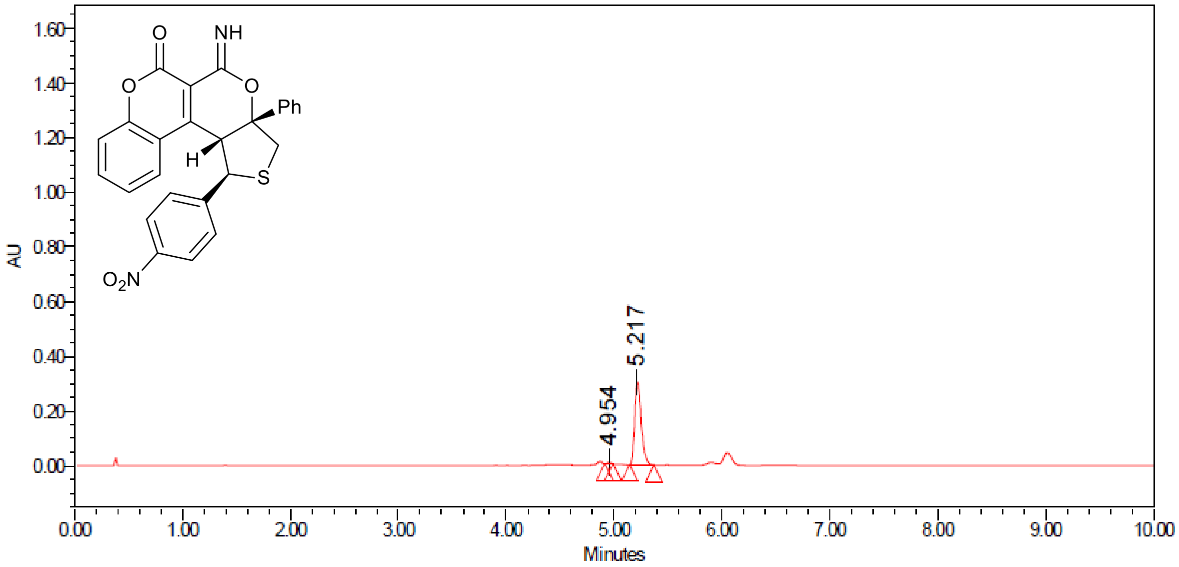

Peak Results

	RT	% Area
1	4.954	0.91
2	5.217	99.09

Racemic sample

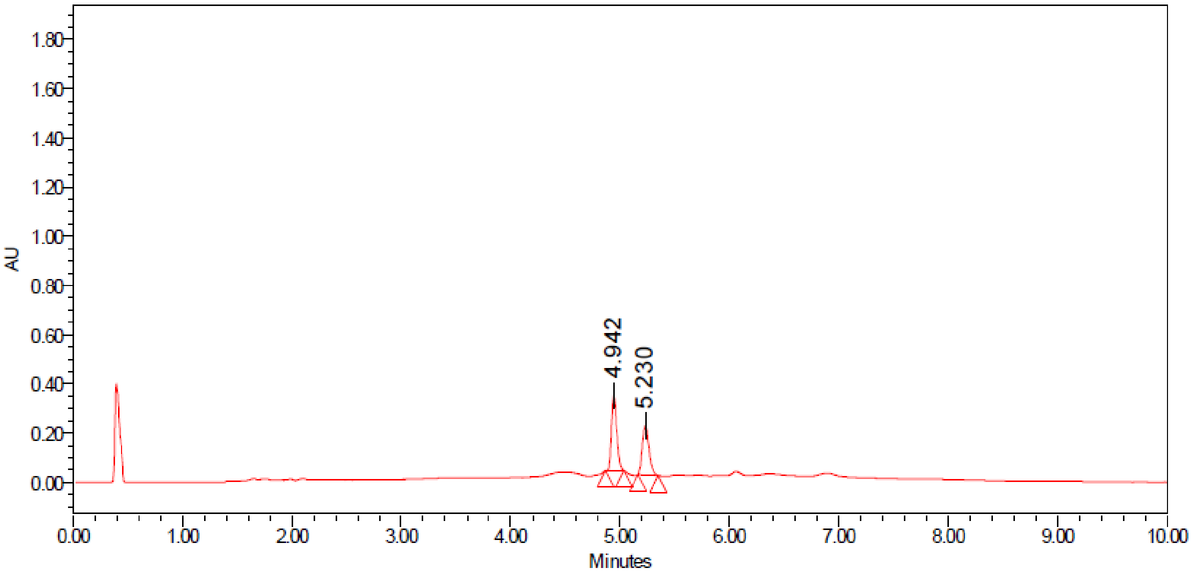

Peak Results

	RT	% Area
1	4.942	55.45
2	5.230	44.55

(1*R*,3a*R*,11c*R*)-5-Imino-3a-phenyl-1-(4-(trifluoromethyl)phenyl)-1,3,3a,11c-tetrahydrothieno[3',4':5,6]pyrano[3,4-c]chromen-6(5*H*)-one 3o

Enantiomerically enriched sample

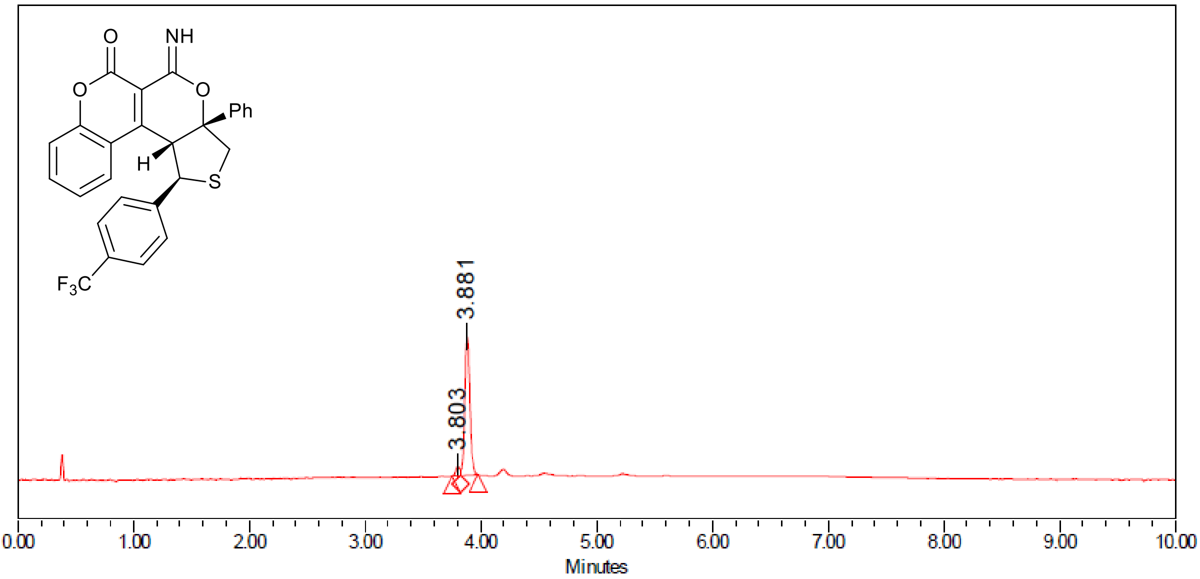

Peak Results

	RT	% Area
1	3.803	5.22
2	3.881	94.78

Racemic sample

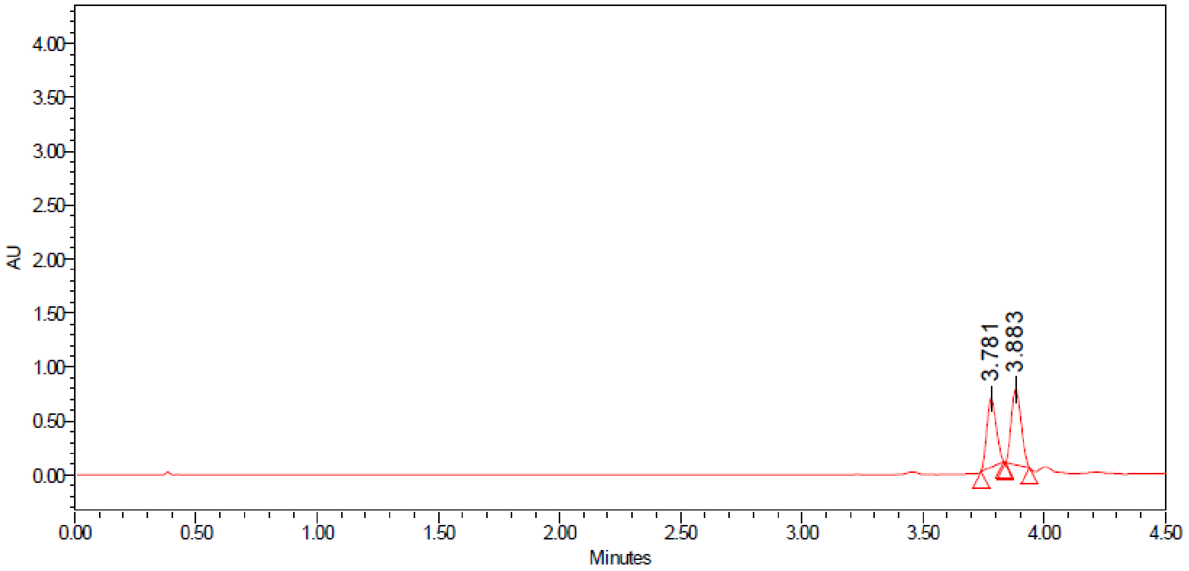

Peak Results

	RT	% Area
1	3.781	46.77
2	3.883	53.23

(1*R*,3a*R*,11c*R*)-5-Imino-9-methoxy-1,3a-diphenyl-1,3,3a,11c-tetrahydrothieno[3',4':5,6]pyrano[3,4-c]chromen-6(5*H*)-one 3p

Enantiomerically enriched sample

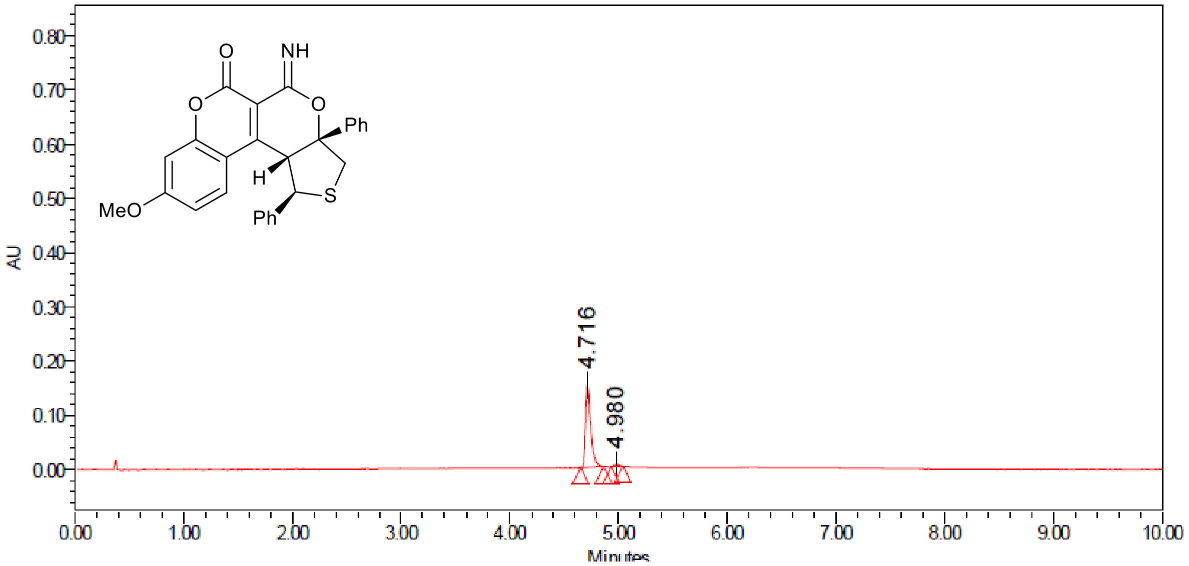

Peak Results

	RT	% Area
1	4.716	97.37
2	4.980	2.63

Racemic sample

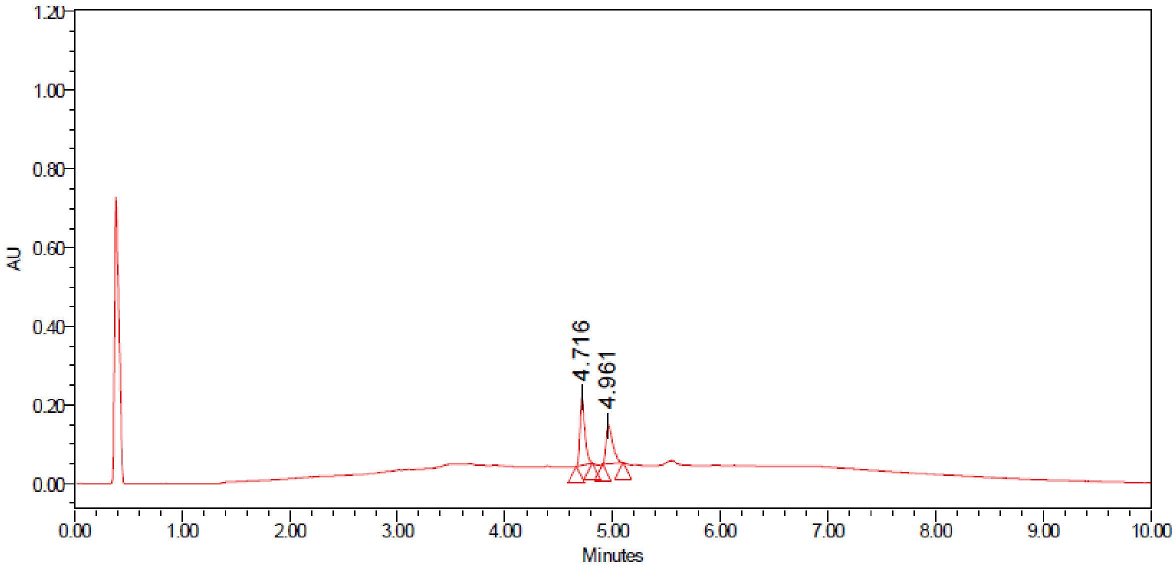

Peak Results

	RT	% Area
1	4.716	56.75
2	4.961	43.25

(1*R*,3a*R*,11c*R*)-10-Bromo-5-imino-1,3a-diphenyl-1,3,3a,11c-tetrahydrothieno[3',4':5,6]pyrano[3,4-c]chromen-6(5*H*)-one 3q

Enantiomerically enriched sample

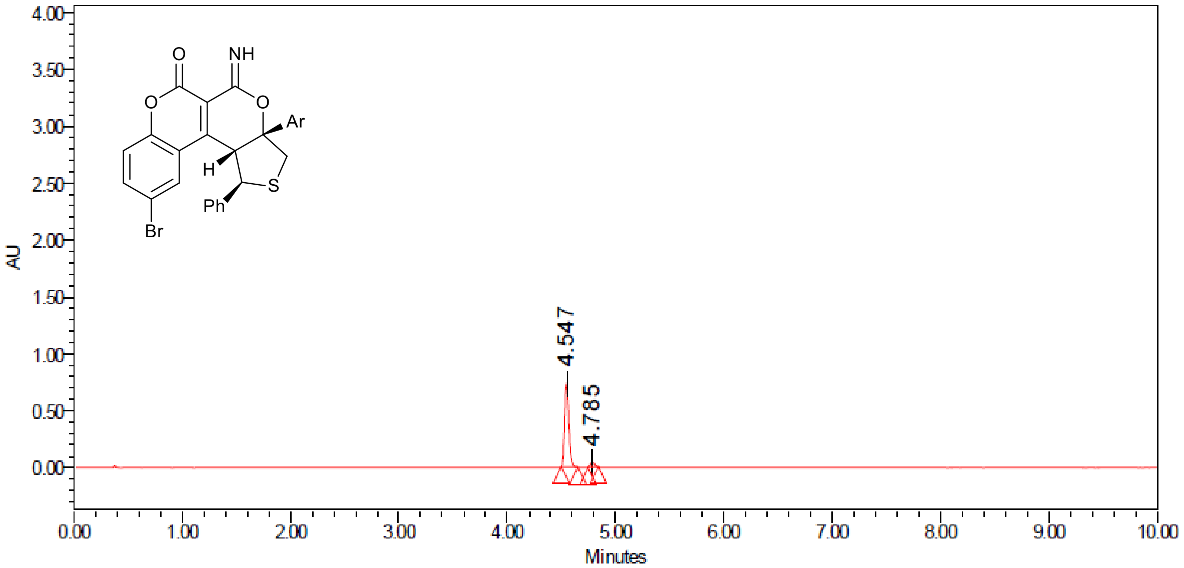

Peak Results

	RT	% Area
1	4.547	94.88
2	4.785	5.12

Racemic sample

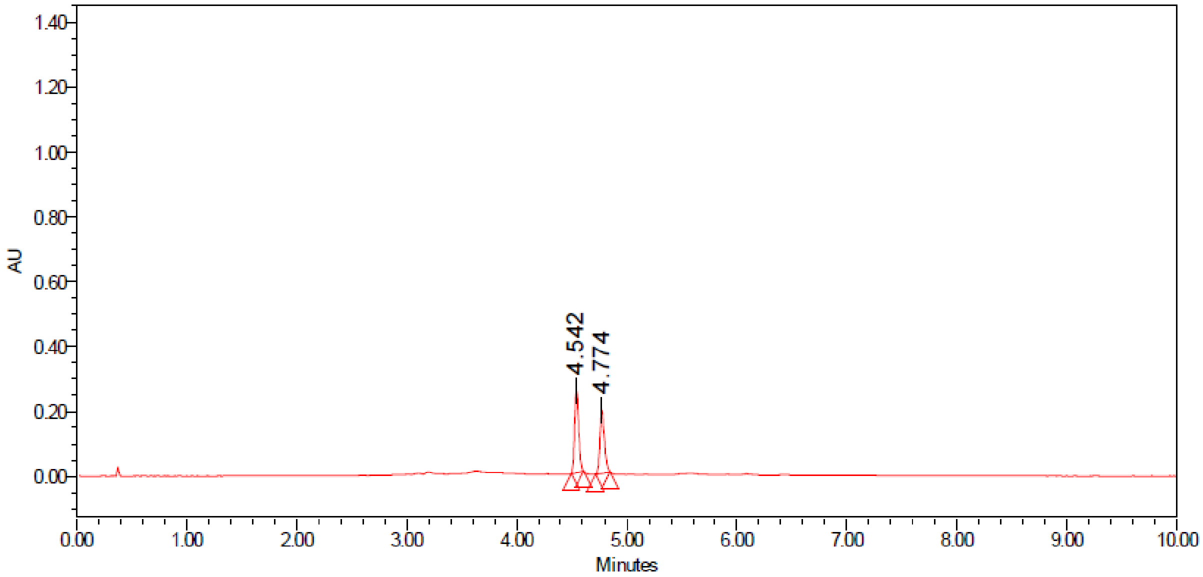

Peak Results

	RT	% Area
1	4.542	53.41
2	4.774	46.59

(1*R*,3a*R*,11c*R*)-1,3a-Diphenyl-1,3,3a,11c-tetrahydrothieno[3',4':5,6]pyrano[3,4-c]chromene-5,6-dione 4a

Enantiomerically enriched sample

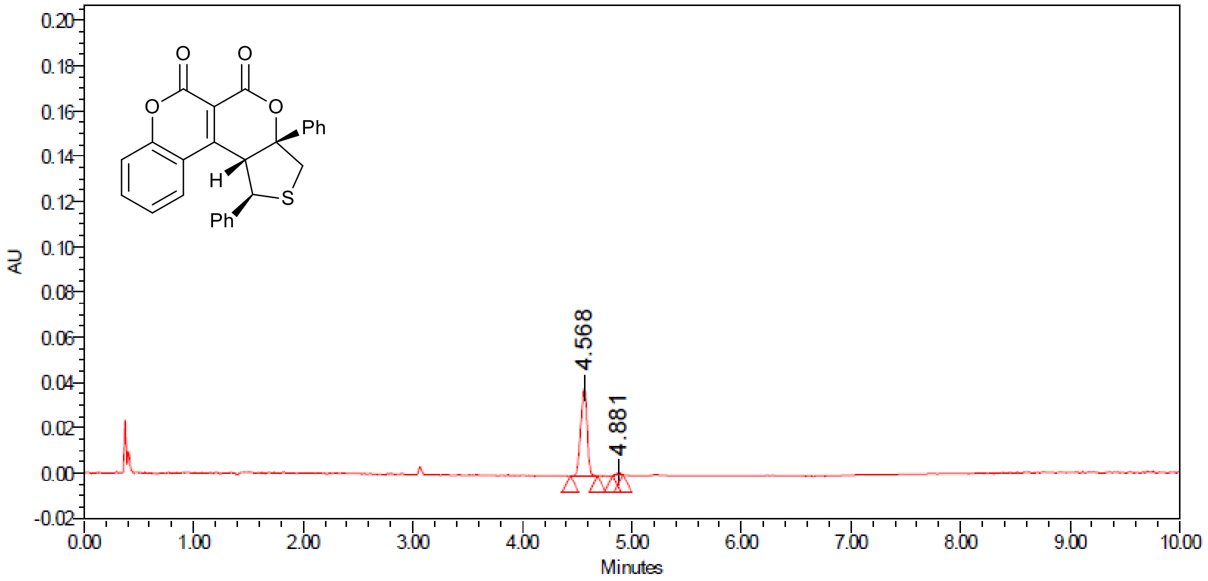

Peak Results

	RT	% Area
1	4.568	98.14
2	4.881	1.86

Racemic sample

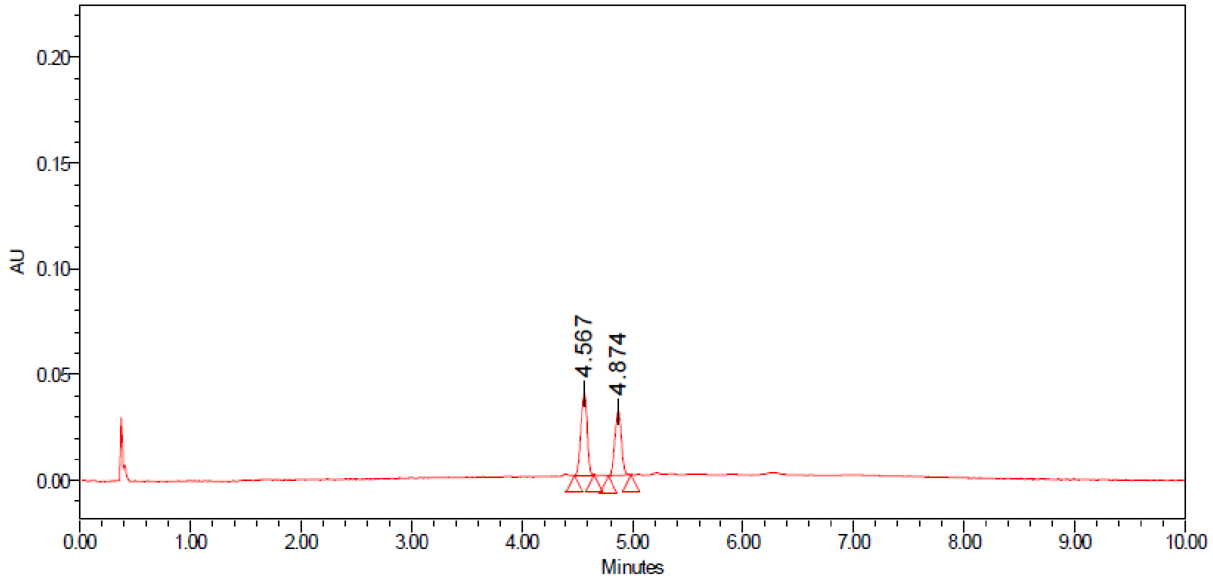

Peak Results

	RT	% Area
1	4.567	55.69
2	4.874	44.31

(1*R*,3a*R*,11c*R*)-3a-(2-Methoxyphenyl)-1-phenyl-1,3,3a,11c-tetrahydrothieno[3',4':5,6]pyrano[3,4-c]chromene-5,6-dione 4b

Enantiomerically enriched sample

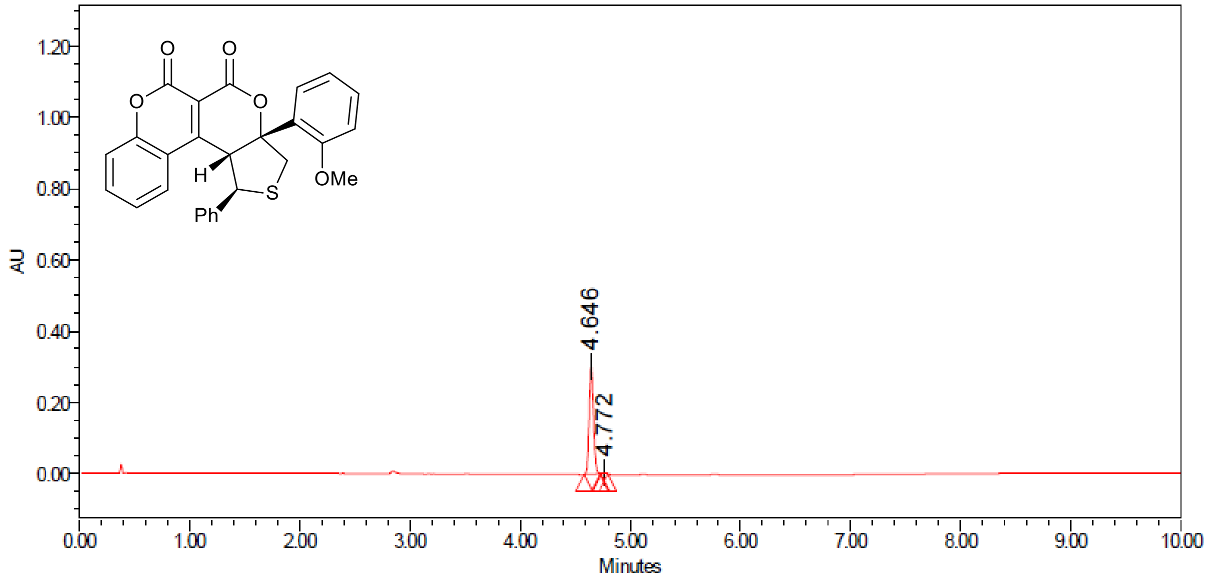

Peak Results

	RT	% Area
1	4.646	99.32
2	4.772	0.68

Racemic sample

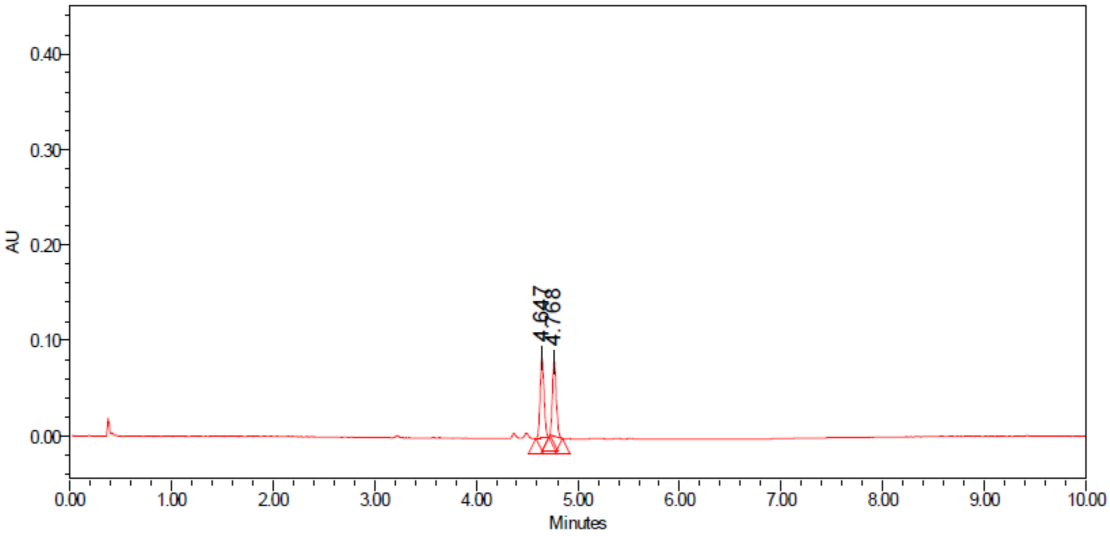

Peak Results

	RT	% Area
1	4.647	51.95
2	4.768	48.05

(1*R*,3a*R*,11c*R*)-3a-(3-Methoxyphenyl)-1-phenyl-1,3,3a,11c-tetrahydrothieno[3',4':5,6]pyrano[3,4-c]chromene-5,6-dione 4c

Enantiomerically enriched sample

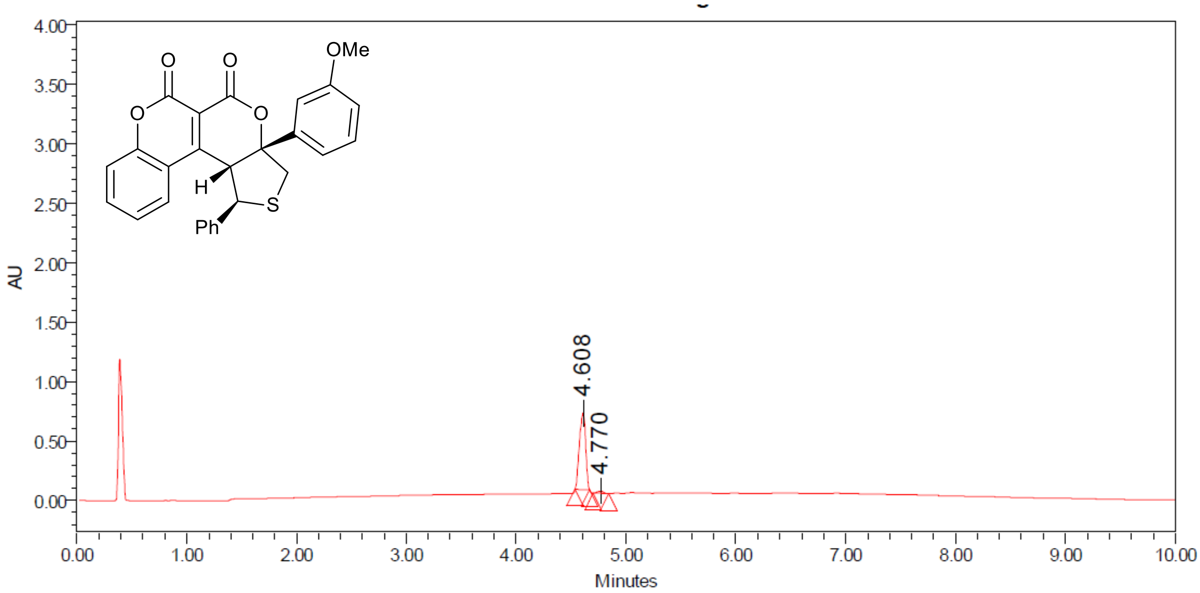

Peak Results

	RT	% Area
1	4.608	97.34
2	4.770	2.66

Racemic sample

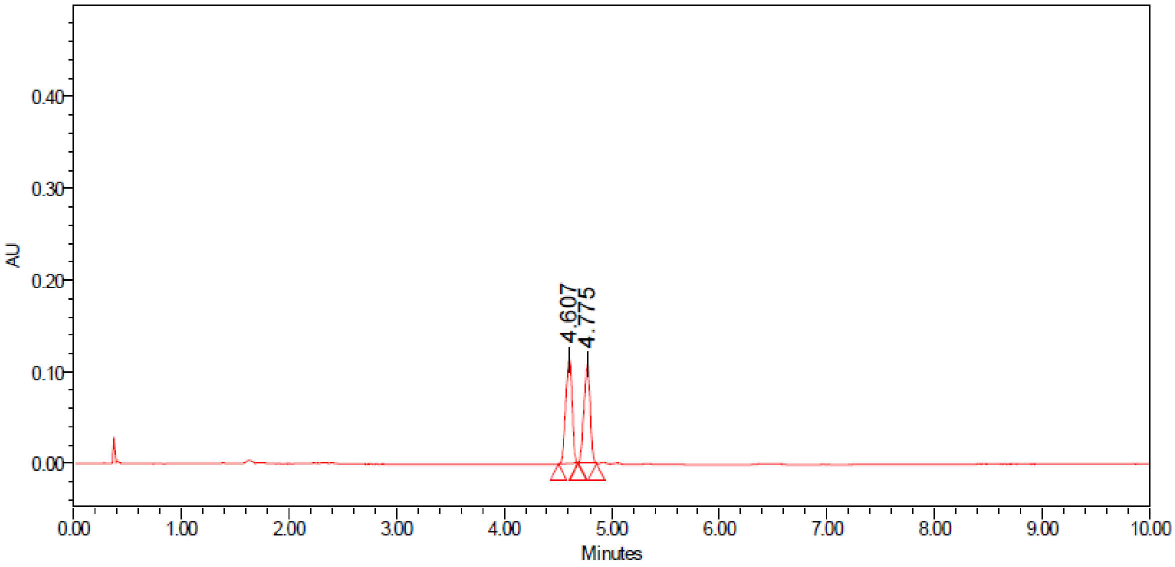

Peak Results

	RT	% Area
1	4.607	51.31
2	4.775	48.69

(1*R*,3a*R*,11c*R*)-3a-(2-Fluorophenyl)-1-phenyl-1,3,3a,11c-tetrahydrothieno[3',4':5,6]pyrano[3,4-c]chromene-5,6-dione 4d

Enantiomerically enriched sample

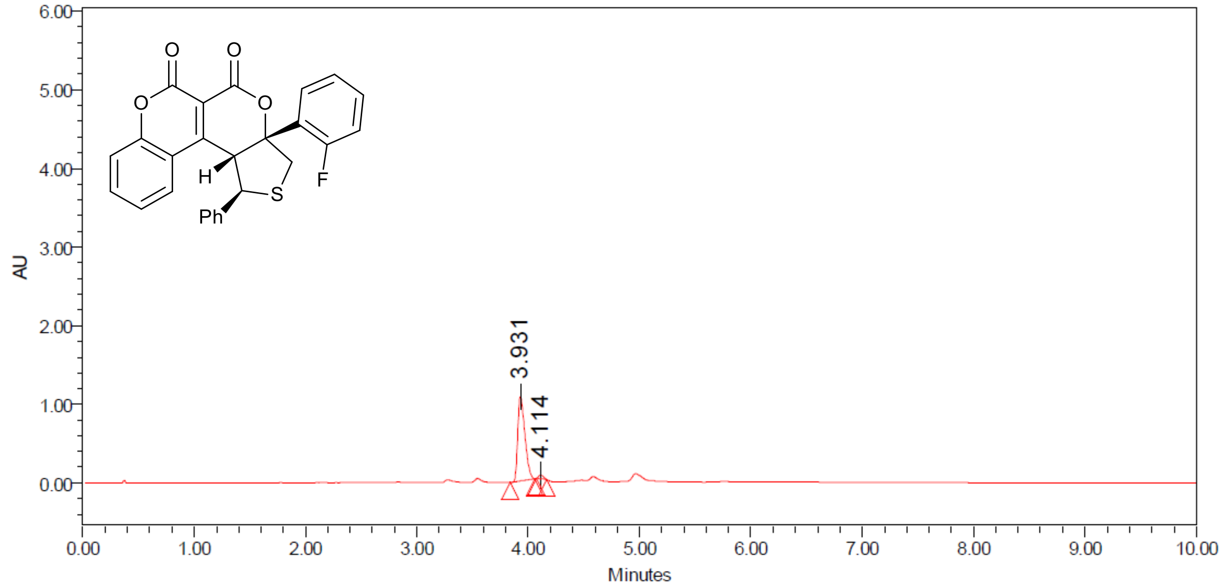

Peak Results

	RT	% Area
1	3.931	96.99
2	4.114	3.01

Racemic sample

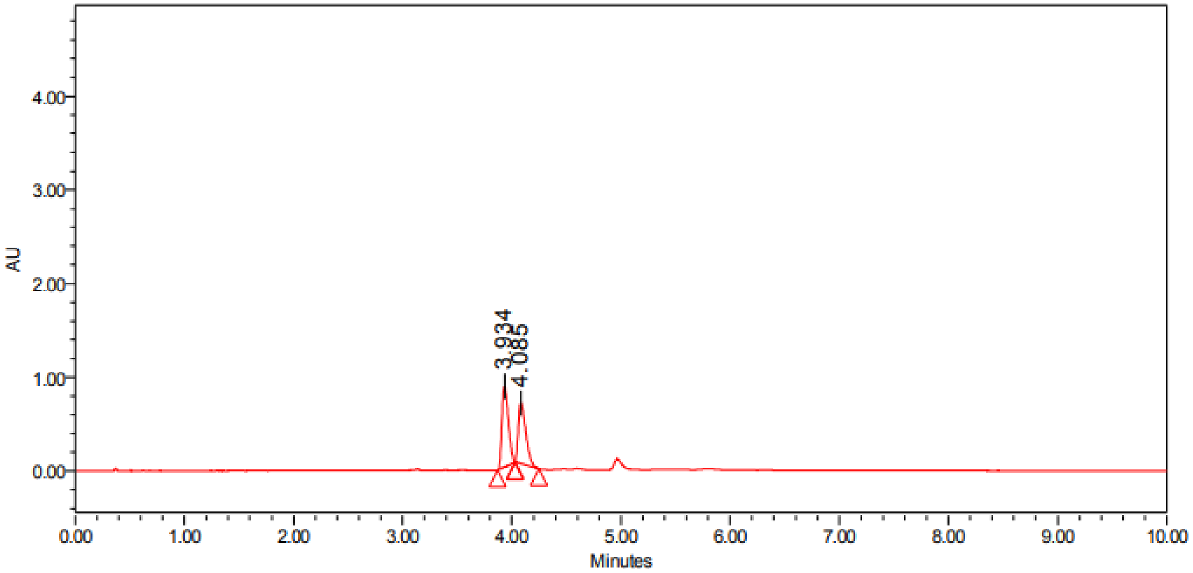

Peak Results

	RT	% Area
1	3.934	53.45
2	4.085	46.55

(1*R*,3a*R*,11c*R*)-1-Phenyl-3a-(4-(trifluoromethyl)phenyl)-1,3,3a,11c-tetrahydrothieno[3',4':5,6]pyrano[3,4-c]chromene-5,6-dione 4e

Enantiomerically enriched sample

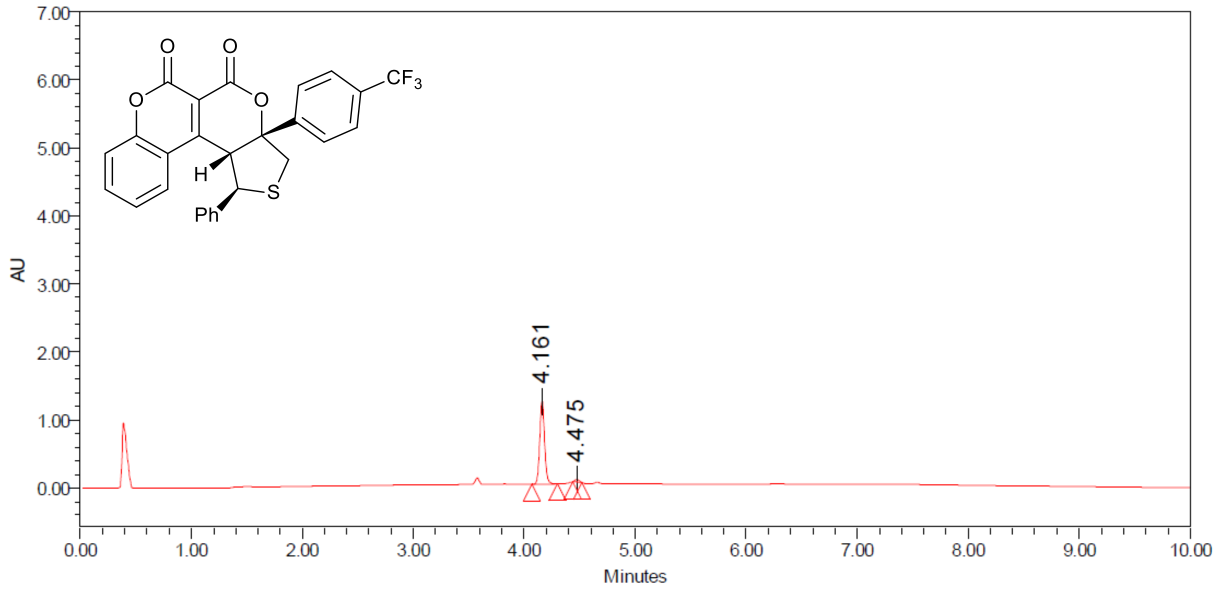

Peak Results

	RT	% Area
1	4.161	97.25
2	4.475	2.75

Racemic sample

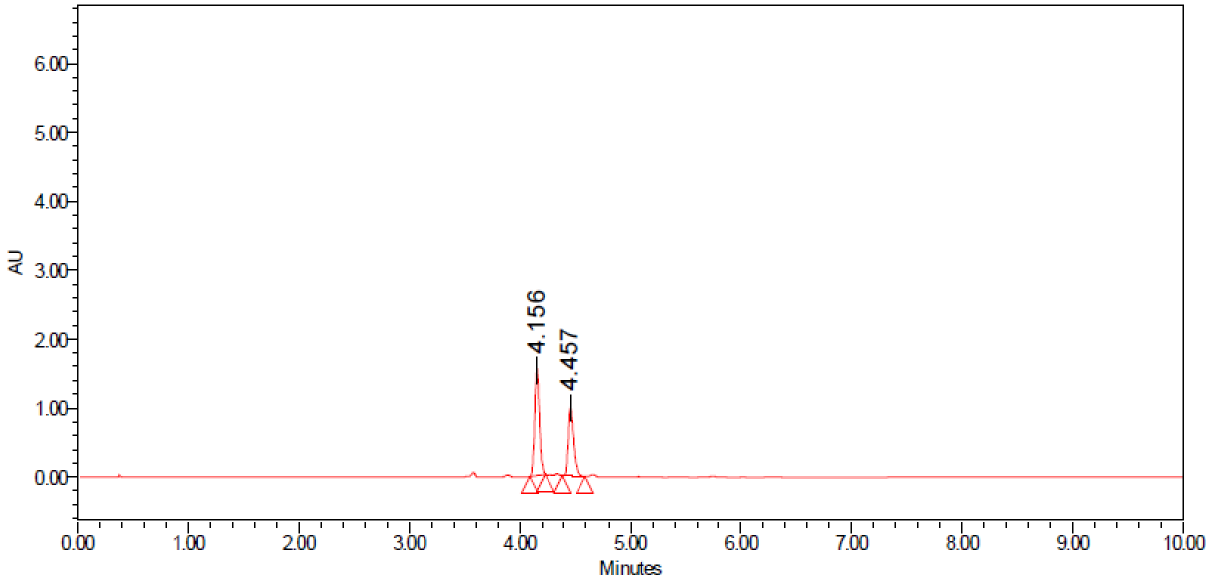

Peak Results

	RT	% Area
1	4.156	57.75
2	4.457	42.25

(1*R*,3a*R*,11c*R*)-3a-(Naphthalen-2-yl)-1-phenyl-1,3,3a,11c-tetrahydrothieno[3',4':5,6]pyrano[3,4-c]chromene-5,6-dione 4f

Enantiomerically enriched sample

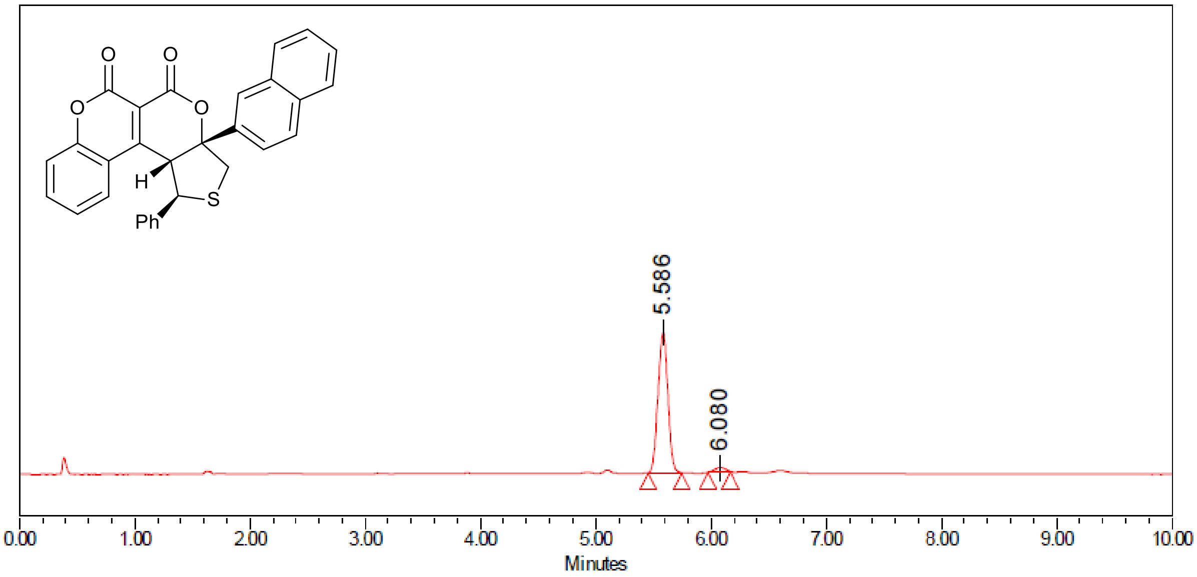

Peak Results

	RT	% Area
1	5.586	96.68
2	6.080	3.32

Racemic sample

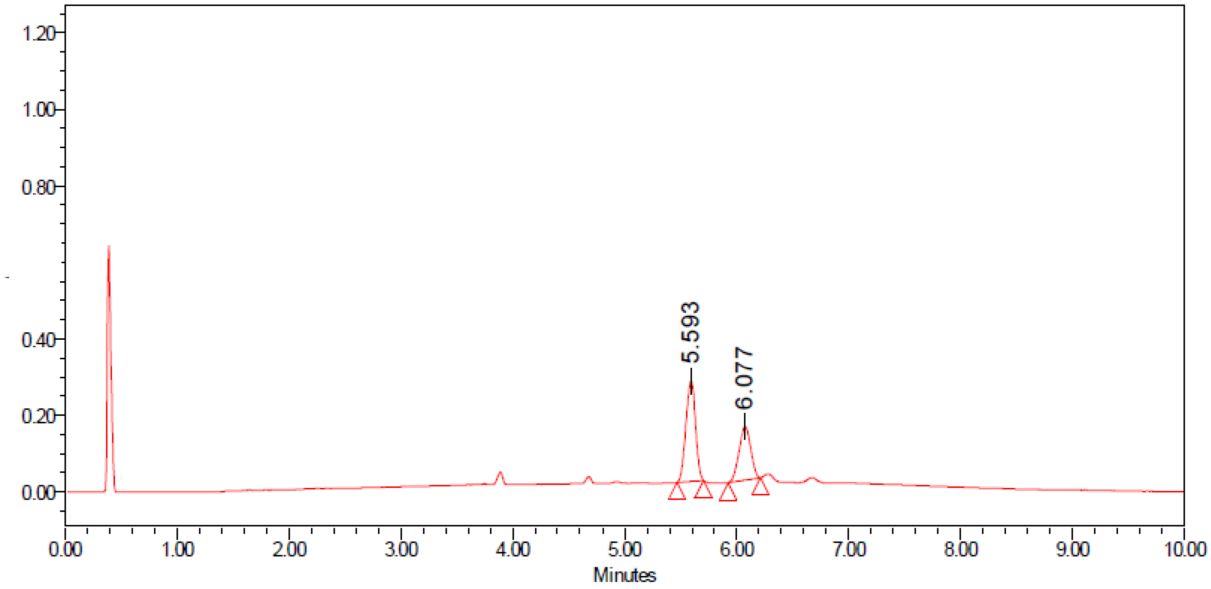

Peak Results

	RT	% Area
1	5.593	59.97
2	6.077	40.03

(1*R*,3a*R*,11c*R*)-1-(4-Methoxyphenyl)-3a-phenyl-1,3,3a,11c-tetrahydrothieno[3',4':5,6]pyrano[3,4-c]chromene-5,6-dione 4g

Enantiomerically enriched sample

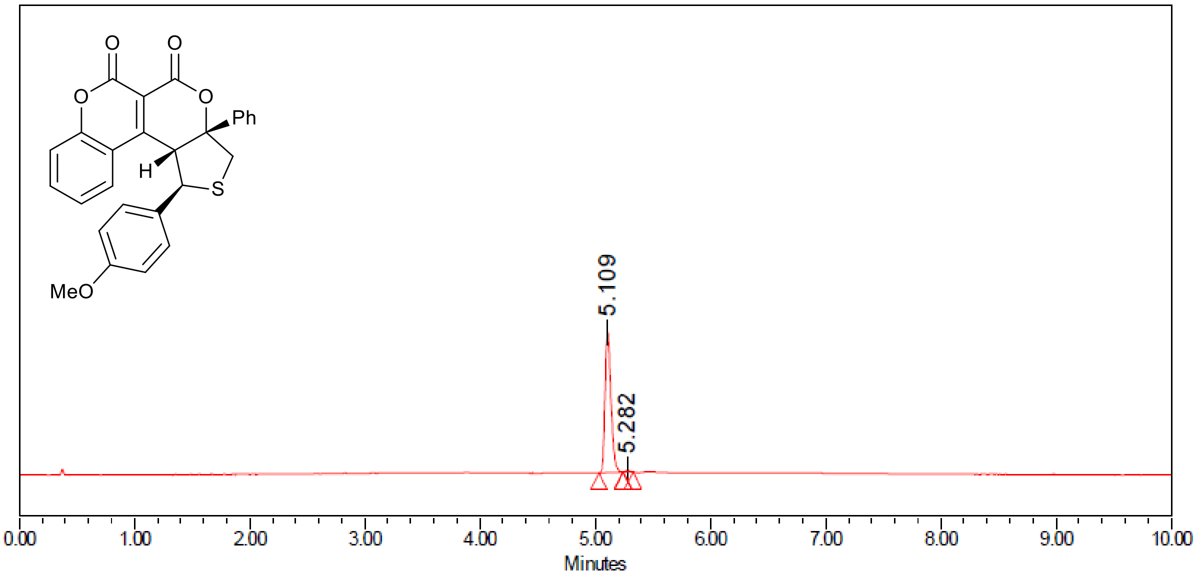

Peak Results

	RT	% Area
1	5.109	99.20
2	5.282	0.80

Racemic sample

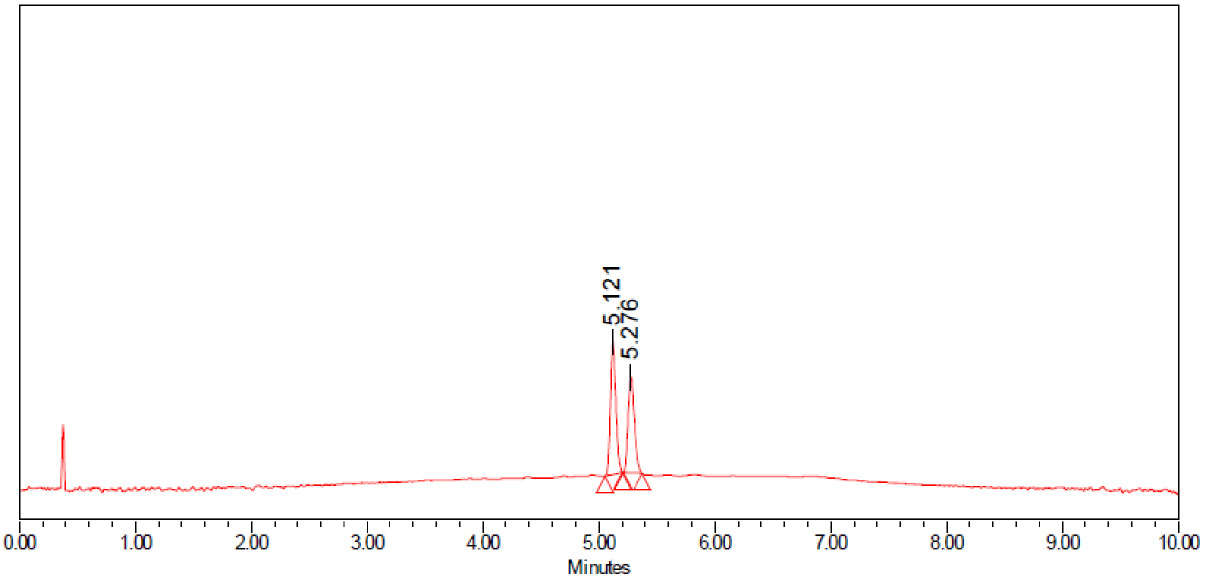

Peak Results

	RT	% Area
1	5.121	55.30
2	5.276	44.70

(1*R*,3a*R*,11c*R*)-3a-Phenyl-1-(*m*-tolyl)-1,3,3a,11c-tetrahydrothieno[3',4':5,6]pyrano[3,4-c]chromene-5,6-dione 4h

Enantiomerically enriched sample

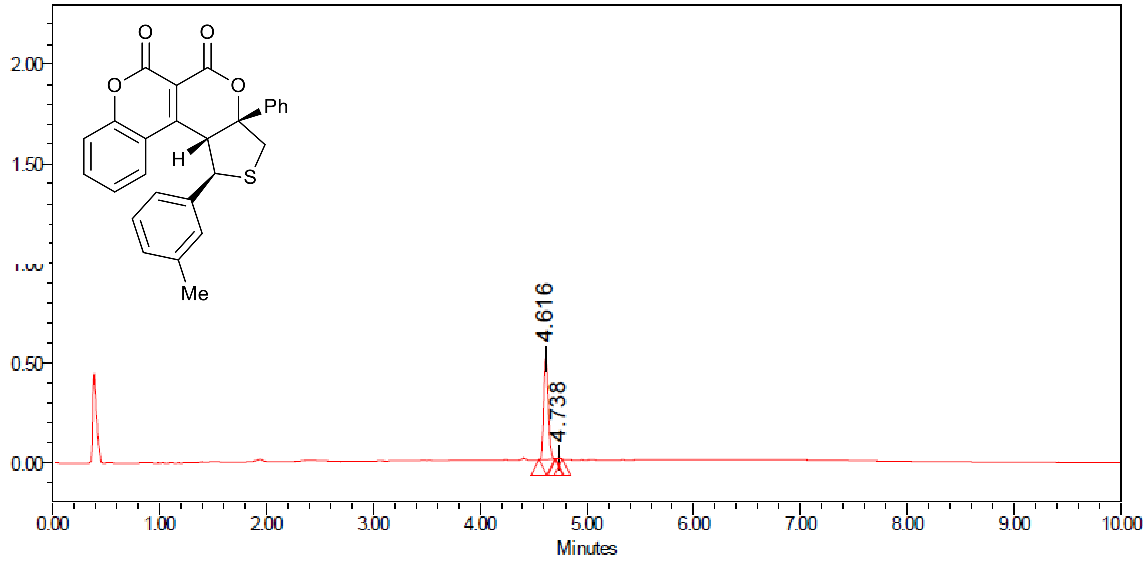

Peak Results

	RT	% Area
1	4.616	99.19
2	4.738	0.81

Racemic sample

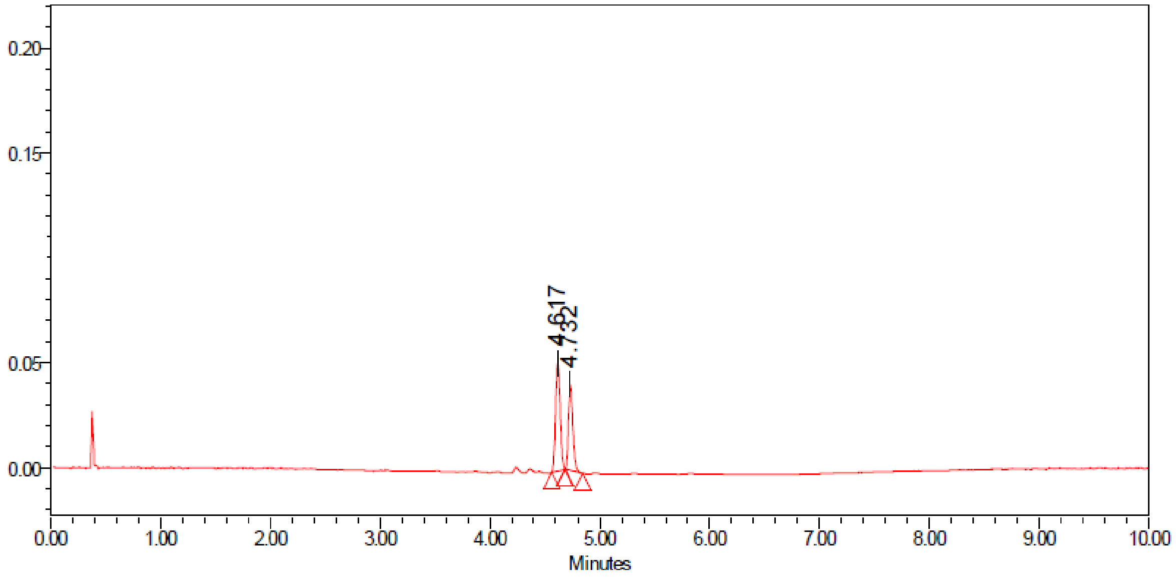

Peak Results

	RT	% Area
1	4.617	55.47
2	4.732	44.53
